# Public health utility of cause of death data: applying empirical algorithms to improve data quality

**DOI:** 10.1186/s12911-021-01501-1

**Published:** 2021-06-02

**Authors:** Sarah Charlotte Johnson, Matthew Cunningham, Ilse N. Dippenaar, Fablina Sharara, Eve E. Wool, Kareha M. Agesa, Chieh Han, Molly K. Miller-Petrie, Shadrach Wilson, John E. Fuller, Shelly Balassyano, Gregory J. Bertolacci, Nicole Davis Weaver, GBD Cause of Death Collaborators, Jalal Arabloo, Alaa Badawi, Akshaya Srikanth Bhagavathula, Katrin Burkart, Luis Alberto Cámera, Felix Carvalho, Carlos A. Castañeda-Orjuela, Jee-Young Jasmine Choi, Dinh-Toi Chu, Xiaochen Dai, Mostafa Dianatinasab, Sophia Emmons-Bell, Eduarda Fernandes, Florian Fischer, Ahmad Ghashghaee, Mahaveer Golechha, Simon I. Hay, Khezar Hayat, Nathaniel J. Henry, Ramesh Holla, Mowafa Househ, Segun Emmanuel Ibitoye, Maryam Keramati, Ejaz Ahmad Khan, Yun Jin Kim, Adnan Kisa, Hamidreza Komaki, Ai Koyanagi, Samantha Leigh Larson, Kate E. LeGrand, Xuefeng Liu, Azeem Majeed, Reza Malekzadeh, Bahram Mohajer, Abdollah Mohammadian-Hafshejani, Reza Mohammadpourhodki, Shafiu Mohammed, Farnam Mohebi, Ali H. Mokdad, Mariam Molokhia, Lorenzo Monasta, Mohammad Ali Moni, Muhammad Naveed, Huong Lan Thi Nguyen, Andrew T. Olagunju, Samuel M. Ostroff, Fatemeh Pashazadeh Kan, David M. Pereira, Hai Quang Pham, Salman Rawaf, David Laith Rawaf, Andre M. N. Renzaho, Luca Ronfani, Abdallah M. Samy, Subramanian Senthilkumaran, Sadaf G. Sepanlou, Masood Ali Shaikh, David H. Shaw, Kenji Shibuya, Jasvinder A. Singh, Valentin Yurievich Skryabin, Anna Aleksandrovna Skryabina, Emma Elizabeth Spurlock, Eyayou Girma Tadesse, Mohamad-Hani Temsah, Marcos Roberto Tovani-Palone, Bach Xuan Tran, Gebiyaw Wudie Tsegaye, Pascual R. Valdez, Prashant M. Vishwanath, Giang Thu Vu, Yasir Waheed, Naohiro Yonemoto, Rafael Lozano, Alan D. Lopez, Christopher J. L. Murray, Mohsen Naghavi

**Affiliations:** 1 Institute for Health Metrics and Evaluation, University of Washington, Seattle, WA USA; 2 Department of Environmental and Occupational Health Sciences, University of Washington, Seattle, WA USA; 3 Health Management and Economics Research Center, Iran University of Medical Sciences, Tehran, Iran; 4 Public Health Risk Sciences Division, Public Health Agency of Canada, Toronto, ON Canada; 5 Department of Nutritional Sciences, University of Toronto, Toronto, ON Canada; 6 Department of Social and Clinical Pharmacy, Charles University, Hradec Kralova, Czech Republic; 7 Institute of Public Health, United Arab Emirates University, Al Ain, United Arab Emirates; 8 Department of Health Metrics Sciences, School of Medicine, University of Washington, Seattle, WA USA; 9 Internal Medicine Department, Hospital Italiano de Buenos Aires, Buenos Aires, Argentina; 10 Board of Directors, Argentine Society of Medicine, Buenos Aires, Argentina; 11 Research Unit on Applied Molecular Biosciences (UCIBIO), University of Porto, Porto, Portugal; 12 Colombian National Health Observatory, National Institute of Health, Bogota, Colombia; 13 Epidemiology and Public Health Evaluation Group, National University of Colombia, Bogota, Colombia; 14 Biomedical Informatics, Seoul National University Hospital, Seoul, South Korea; 15 Faculty of Biology, Hanoi National University of Education, Hanoi, Vietnam; 16 Department of Epidemiology and Biostatistics, Shahroud University of Medical Sciences, Shahroud, Iran; 17 Department of Epidemiology, Shiraz University of Medical Sciences, Shiraz, Iran; 18 Associated Laboratory for Green Chemistry (LAQV), University of Porto, Porto, Portugal; 19 Institute of Gerontological Health Services and Nursing Research, Ravensburg-Weingarten University of Applied Sciences, Weingarten, Germany; 20 Student Research Committee, Iran University of Medical Sciences, Tehran, Iran; 21 Health Systems and Policy Research, Indian Institute of Public Health Gandhinagar, Gandhinagar, India; 22 Institute of Pharmaceutical Sciences, University of Veterinary and Animal Sciences, Lahore, Pakistan; 23 Department of Pharmacy Administration and Clinical Pharmacy, Xian Jiaotong University, Xian, China; 24 Big Data Institute, University of Oxford, Oxford, UK; 25 Kasturba Medical College, Mangalore,, Manipal Academy of Higher Education, Manipal, India; 26 College of Science and Engineering, Hamad Bin Khalifa University, Doha, Qatar; 27 Department of Health Promotion and Education, University of Ibadan, Ibadan, Nigeria; 28 Mashhad University of Medical Sciences, Mashhad, Iran; 29 Department of Epidemiology and Biostatistics, Health Services Academy, Islamabad, Pakistan; 30 School of Traditional Chinese Medicine, Xiamen University Malaysia, Sepang, Malaysia; 31 School of Health Sciences, Kristiania University College, Oslo, Norway; 32 Global Community Health and Behavioral Sciences, Tulane University, New Orleans, LA USA; 33 Neurophysiology Research Center, Hamadan University of Medical Sciences, Hamadan, Iran; 34 Brain Engineering Research Center, Institute for Research in Fundamental Sciences, Tehran, Iran; 35 CIBERSAM, San Juan de Dios Sanitary Park, Sant Boi de Llobregat, Spain; 36 Catalan Institution for Research and Advanced Studies (ICREA), Barcelona, Spain; 37 Department of Systems, Populations, and Leadership, University of Michigan, Ann Arbor, MI USA; 38 Department of Primary Care and Public Health, Imperial College London, London, UK; 39 Digestive Diseases Research Institute, Tehran University of Medical Sciences, Tehran, Iran; 40 Non-Communicable Disease Research Center, Shiraz University of Medical Sciences, Shiraz, Iran; 41 Non-Communicable Diseases Research Center, Tehran University of Medical Sciences, Tehran, Iran; 42 Department of Epidemiology and Biostatistics, Shahrekord University of Medical Sciences, Shahrekord, Iran; 43 Department of Nursing, Mashhad University of Medical Sciences, Mashhad, Iran; 44 Health Systems and Policy Research Unit, Ahmadu Bello University, Zaria, Nigeria; 45 Heidelberg Institute of Global Health (HIGH), Heidelberg University, Heidelberg, Germany; 46 National Institute of Health Research (NIHR), Tehran University of Medical Sciences, Tehran, Iran; 47 Faculty of Life Sciences and Medicine, King’s College London, London, UK; 48 Clinical Epidemiology and Public Health Research Unit, Burlo Garofolo Institute for Maternal and Child Health, Trieste, Italy; 49 World Health Organization (WHO) Centre on eHealth, University of New South Wales, Sydney, NSW Australia; 50 Department of Biotechnology, University of Central Punjab, Lahore, Pakistan; 51 Institute for Global Health Innovations, Duy Tan University, Hanoi, Vietnam; 52 Department of Psychiatry and Behavioural Neurosciences, McMaster University, Hamilton, ON Canada; 53 Department of Psychiatry, University of Lagos, Lagos, Nigeria; 54 Henry M Jackson School of International Studies, University of Washington, Seattle, WA USA; 55 Iran University of Medical Sciences, Tehran, Iran; 56 Associated Laboratory for Green Chemistry (LAQV), University of Porto, Oporto, Portugal; 57 Academic Public Health England, Public Health England, London, UK; 58 WHO Collaborating Centre for Public Health Education and Training, Imperial College London, London, UK; 59 University College London Hospitals, London, UK; 60 School of Social Sciences and Psychology, Western Sydney University, Penrith, NSW Australia; 61 Translational Health Research Institute, Western Sydney University, Penrith, NSW Australia; 62 Department of Entomology, Ain Shams University, Cairo, Egypt; 63 Emergency Department, Manian Medical Centre, Erode, India; 64 Independent Consultant, Karachi, Pakistan; 65 Institute for Population Health, King’s College London, London, UK; 66 School of Medicine, University of Alabama at Birmingham, Birmingham, AL USA; 67 Medicine Service, US Department of Veterans Affairs (VA), Birmingham, AL USA; 68 Department No.16, Moscow Research and Practical Centre ON Addictions, Moscow, Russia; 69 Therapeutic Department, Balashiha Central Hospital, Balashikha, Russia; 70 Department of Biomedical Sciences, Arba Minch University, Arba Minch, Ethiopia; 71 Pediatric Intensive Care Unit, King Saud University, Riyadh, Saudi Arabia; 72 Department of Pathology and Legal Medicine, University of São Paulo, Ribeirão Preto, Brazil; 73 Modestum LTD, London, UK; 74 Department of Health Economics, Hanoi Medical University, Hanoi, Vietnam; 75 College of Medicine and Health Sciences, Bahir Dar University, Bahir Dar, Ethiopia; 76 Argentine Society of Medicine, Buenos Aires, Argentina; 77 Velez Sarsfield Hospital, Buenos Aires, Argentina; 78 Department of Biochemistry, Jagadguru Sri Shivarathreeswara University, Mysore, India; 79 Center of Excellence in Behavioral Medicine, Nguyen Tat Thanh University, Ho Chi Minh City, Vietnam; 80 Foundation University Medical College, Foundation University Islamabad, Islamabad, Pakistan; 81 Department of Neuropsychopharmacology, National Center of Neurology and Psychiatry, Kodaira, Japan; 82 Department of Public Health, Juntendo University, Tokyo, Japan; 83 Melbourne School of Population and Global Health, University of Melbourne, Melbourne, VIC Australia; 84 Department of Health Metrics Sciences, Director of Subnational Burden of Disease Estimation, Institute for Health Metrics and Evaluation School of Medicine, University of Washington, 2301 5th Ave. Suite 600, Seattle, WA 98121 USA

**Keywords:** Redistribution, Garbage codes, Cause of death, Global Burden of Disease, Star ranking system, Vital registration

## Abstract

**Background:**

Accurate, comprehensive, cause-specific mortality estimates are crucial for informing public health decision making worldwide. Incorrectly or vaguely assigned deaths, defined as garbage-coded deaths, mask the true cause distribution. The Global Burden of Disease (GBD) study has developed methods to create comparable, timely, cause-specific mortality estimates; an impactful data processing method is the reallocation of garbage-coded deaths to a plausible underlying cause of death. We identify the pattern of garbage-coded deaths in the world and present the methods used to determine their redistribution to generate more plausible cause of death assignments.

**Methods:**

We describe the methods developed for the GBD 2019 study and subsequent iterations to redistribute garbage-coded deaths in vital registration data to plausible underlying causes. These methods include analysis of multiple cause data, negative correlation, impairment, and proportional redistribution. We classify garbage codes into classes according to the level of specificity of the reported cause of death (CoD) and capture trends in the global pattern of proportion of garbage-coded deaths, disaggregated by these classes, and the relationship between this proportion and the Socio-Demographic Index. We examine the relative importance of the top four garbage codes by age and sex and demonstrate the impact of redistribution on the annual GBD CoD rankings.

**Results:**

The proportion of least-specific (class 1 and 2) garbage-coded deaths ranged from 3.7% of all vital registration deaths to 67.3% in 2015, and the age-standardized proportion had an overall negative association with the Socio-Demographic Index. When broken down by age and sex, the category for unspecified lower respiratory infections was responsible for nearly 30% of garbage-coded deaths in those under 1 year of age for both sexes, representing the largest proportion of garbage codes for that age group. We show how the cause distribution by number of deaths changes before and after redistribution for four countries: Brazil, the United States, Japan, and France, highlighting the necessity of accounting for garbage-coded deaths in the GBD.

**Conclusions:**

We provide a detailed description of redistribution methods developed for CoD data in the GBD; these methods represent an overall improvement in empiricism compared to past reliance on a priori knowledge.

**Supplementary Information:**

The online version contains supplementary material available at 10.1186/s12911-021-01501-1.

## Background

Across humanity, we know two events to be inevitable: birth and death. In order to maximize the quality and quantity of time spent between these two events, we need accurate, timely, and cause-specific mortality estimates. Even though systematic cause of death (CoD) reporting has improved since the first such records measuring bubonic plague mortality [1], no country has yet created a perfectly accurate death registration system. The highest-quality CoD data are reported via vital registration (VR) systems, through which “the continuous, permanent, compulsory and universal” recording of vital demographic events occurs “in accordance with the legal requirements of a country” [2, 3]. The presence of VR systems is far from ubiquitous and remains especially inadequate in lower- and lower-middle-income countries [4–6]. Furthermore, the process of completing and accurately coding a death certificate according to the international standard established by the International Statistical Classification of Diseases and Related Health Problems (ICD) is challenging for all countries, regardless of income status [7].

According to the ICD, only one CoD is reported for statistical purposes: the underlying cause of death (UCoD), i.e., the disease or injury that initiated the chain of events leading to death [8]. Physicians often do not receive adequate training in the public health importance of ICD rules, however, and death certificates are regularly filled out incorrectly [9–11]. As a result, many deaths are ascribed to “garbage” codes, i.e. codes that are not specific enough, are an immediate or intermediate CoD, or impossible CoD [12, 13]. Sepsis, for example, is often listed as an UCoD, however, a number of conditions, including malaria, diabetes, or a road traffic injury [14] may be the underlying cause that leads to sepsis. Garbage codes mask the distribution of true underlying causes, and numerous country-specific data quality analyses that address garbage coding have revealed different mortality patterns than initially reported [15–20]. Furthermore, coding practices vary across age groups, sexes, space, and time, severely hindering intra- and inter-country comparability of cause-specific mortality over time and limiting the usability of CoD data for public health purposes [21–24].

The Global Burden of Disease (GBD) study, a tool for quantifying health loss from hundreds of diseases, injuries, and risk factors, is one response to the question of how to generate usable cause-specific mortality estimates from a collection of imperfect, heterogeneous data [2, 25, 26]. The GBD produces regular, timely estimates of cause-specific mortality that are comparable by age, sex, year, and location from 1980 onwards. Accounting for garbage-coded deaths is one of the key data processing steps in creating cause-specific mortality estimates and reveals a mortality distribution that countries can use to compare the mortality level and composition over time, across age groups and sexes. Here we present the methods developed to account for garbage-coded deaths in VR data by location, year, age, and sex in the GBD 2013 study through GBD 2020, in addition to describing the pattern of garbage-coded deaths in the world. Furthermore, we draw from previously established criteria, namely coverage and frequency of garbage-coded deaths, to evaluate the overall quality of CoD VR data in the world [16, 27].

## Methods

The GBD produces a continuously updated, comprehensive, comparable database of standardized CoD data by age, sex, location, and year from 1980 onwards. We aim to include as much CoD data as possible: rather than exclude data that do not fit the ideal, we have devised a number of methods to enhance the usability of a variety of CoD data sources. Existing CoD data sources differ based primarily on the method by which the data were collected (e.g., VR, verbal autopsy, sibling history) and the coding system and format used to report the CoD data (e.g., International Classification of Diseases [ICD]-9 and ICD-10) (Additional file 1: Figure 1). This variation creates a number of challenges in standardizing the data, including unknown age and/or sex, tabulated (aggregated) cause codes, misclassification of underlying causes to another cause or to garbage codes, and stochastic noise in deaths over time. An overview of the process for building the CoD database is summarized briefly below (Additional file 1: Figure 2), though an in-depth description of all methods is outside the scope of this paper and described elsewhere [2]. Specifically, we focus on the set of algorithms used to reallocate garbage-coded deaths to a most likely UCoD, collectively referred to as “redistribution” (the third box in Additional file 1: Figure 2). This study complies with the Guidelines for Accurate and Transparent Health Estimates Reporting (GATHER) statement [28]. The GBD study used de-identified data, and the waiver of informed consent was reviewed and approved by the University of Washington Institutional Review Board (application number 46665). Data preparation and analyses were carried out using R version 3.5.1 and Python 3 [29, 30].

**Fig. 1:**
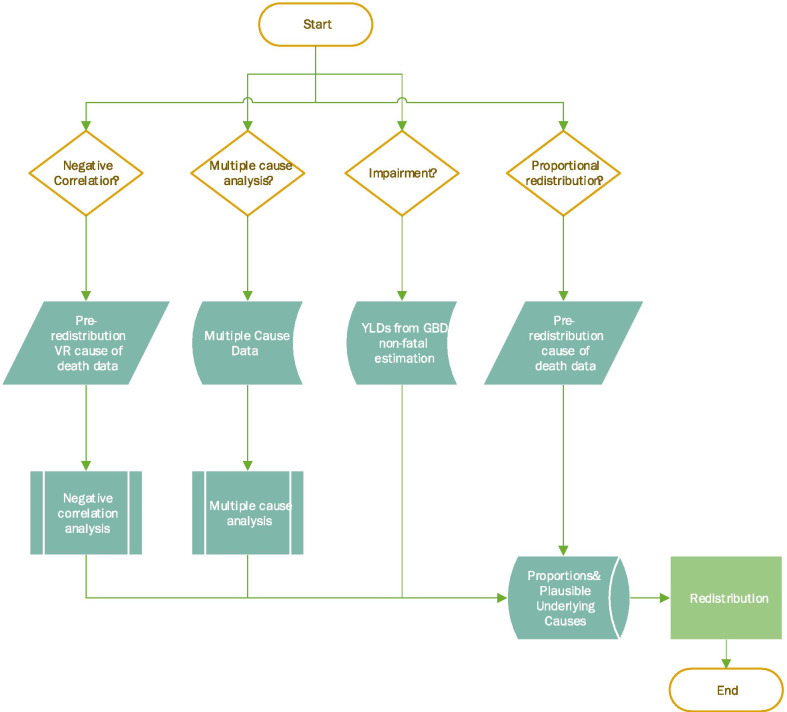
Flowchart for methods used to determine inputs into redistribution algorithm

We will first briefly cover the key steps in the data processing pipeline to contextualize how redistribution of garbage-coded deaths fits into creating the CoD database (Additional file 1: Figure 2). First, all causes of death are mapped from their original coding onto the GBD cause list [2]. Second, observations from some CoD data sources are not available by detailed age and sex, and must be split into detailed age and sex groups. This is achieved by using cause, age, and sex specific global mortality rates generated from CoD VR where complete age and sex detail is available. Alongside population, these mortality rates are used to estimate an expected number of deaths in each detailed age group and for both sexes, which are then scaled to total the deaths in the original non-detailed observation. Additional details on the age and sex splitting process can be found elsewhere [2]. Third, deaths where the cause has been misclassified to Alzheimer’s disease and other dementias are reassigned to the most plausible underlying cause [2]. Fourth, deaths assigned to a garbage code are redistributed (the focus of this manuscript) (Fig. 1). Fifth, misclassification of HIV-related deaths is corrected [2, 31, 32]. Finally, noisy data due to stochastic variation are smoothed and CoD data are uploaded to a central database for use in the GBD fatal estimation process [2]. This paper provides further detail on the most current methods developed to account for garbage-coded deaths in VR data using the detailed ICD-9 and ICD-10 nosological classification systems, as these data represent the vast majority of GBD’s mortality data (Additional file 1: Figure 1).

### Identification of garbage codes

In the first step of the cause of death database creation, every ICD code is mapped to a corresponding CoD in the mutually exclusive, collectively exhaustive GBD cause hierarchy (Additional file 1: Figure 3) [2]. Not every ICD code is a valid UCoD in the GBD hierarchy, however; garbage-coded deaths describe ICD codes that cannot or should not be considered the UCoD (Additional file 1: Figure 4) [33]. This includes impossible causes of death, e.g., senility; non-specific causes, e.g., ill-defined cancer site; causes that the GBD considers a symptom rather than a cause, e.g., back pain; and intermediate or immediate causes that result from other underlying conditions, e.g., heart failure, sepsis. We refer to these codes as “garbage codes”; garbage-coded deaths are not lost during analysis, but instead grouped based on diagnostic relatedness and collectively reassigned to the most probable UCoD during a process we refer to as redistribution, described in detail in the following sections.

### Categorization of garbage codes

While all garbage codes are alike in that they cannot (or should not) be considered the UCoD, not all garbage codes are the same, and vary in their level of specificity. For example, deaths that are garbage-coded as “sepsis” could be attributed to hundreds of underlying causes of deaths, whereas deaths garbage-coded to “unspecified stroke” have a short list of possible underlying causes. In GBD 2016, garbage levels, here termed “classes”, were created to categorize garbage codes into four classes of increasing specificity [34]. A more detailed explanation of these classes has been published previously [35]; and they are briefly described in Box 1 (a table of ICD codes by garbage class can be found in Additional file 1: Figure 4).

**Box 1:** Classes of garbage codes

**Class 1** includes garbage codes that are attributable to causes within all three Level 1 GBD causes in the GBD cause hierarchy (communicable, maternal, neonatal, and nutritional disease (CMNN); non-communicable disease (NCD); injury). For example, “sepsis” or “peritonitis” could be the result of any of the three Level 1 causes, and as such are the least specific class of garbage code and are redistributed across all three cause groupings
**Class 2** includes garbage codes that are attributable to causes within a single Level 1 GBD cause in the GBD cause hierarchy, e.g., “unintentional unspecified injuries” and such deaths are all redistributed onto injuries causes
**Class 3** includes garbage codes that are attributable to causes within a single Level 2 GBD cause, e.g., “ill-defined cancer site” deaths are all redistributed onto neoplasms
**Class 4** includes garbage codes that are attributable to causes within a single Level 3 GBD cause, e.g., “unspecified stroke” deaths will be redistributed to one of ischemic stroke, intracerebral hemorrhage, or subarachnoid hemorrhage

Classes one and two are collectively referred to as major garbage; correction of these classes has the most important policy implications, and the proportion of age-standardized major garbage out of all deaths in each location and year is a key component of the star rating data-quality metric produced by GBD [2], described in further detail below. In GBD 2020, 16.9% of ICD-9 and ICD-10 VR data across all years were major garbage-coded deaths, with the percent of major garbage staying relatively stable over time, ranging from a low of 13.5% to a high of 18.4% during the period from 1980 to 2019 (Additional file 1: Figure 5).

### Star rating

Fatal GBD estimation is most accurate when using data from complete VR systems that span consecutive years, with a low proportion of garbage-coded deaths. In GBD 2013, the Vital Statistics Performance Index (VSPI), a composite of six metrics, was created to empirically measure the performance of VR systems [27]. In GBD 2016, a simpler system was developed, using a star rating system from 0 to 5 to represent data quality for a location across a given time series [34]. For any given location-year, the two components that determine this star rating are the proportion of age-standardized major garbage and level of completeness. Completeness is a measure of how successfully the VR captures deaths that occur in a location-year (regardless of garbage coding). It is calculated as the fraction of total reported deaths in the VR over total GBD estimated all-cause mortality deaths. These components are then used to calculate a percent well certified (PWC) value between 0 and 1 (Eq. 1).

1 $$PWC = Percent_{Completeness} \times \left( {1 - Percent_{MajorGarbage} } \right)$$

Star values are then assigned based on the calculated PWC value. A mapping of PWC values to star ratings can be found in the Additional file 1 (Additional file 1: Figure 6). This method for assigning a star rating to a specific location-year of data and then summarizing that metric across a time series is described in detail elsewhere [34]. A location can increase its number of stars by decreasing the proportion of major garbage-coded deaths, increasing the total number of deaths captured, and increasing the number of available years of data. Data quality, as measured via the star ranking system, ranges substantially across GBD locations and within countries with subnational detail available (Additional file 1: Figure 7).

### Redistribution

Redistribution is the process of reallocating garbage-coded deaths to plausible underlying causes [12]. For each group of diagnostically related garbage codes, we define a set of probable underlying causes of death and the proportion of garbage-coded deaths that are redistributed to each underlying cause, separately by GBD age group, sex, location, and year. We want to note that while uncertainty intervals for these proportions are calculated, they are used only to aid in the modelling of data that have completed all steps of the data processing pipeline (Additional file 1: Figure 2). They are not used to inform redistribution of the garbage coded deaths. Thus, specific details regarding calculation of redistribution uncertainty have been omitted from this paper but are described in detail elsewhere [2].

There are four main methods used to determine a set of plausible underlying causes and proportions for a given group of garbage codes, explained in detail in subsequent paragraphs: (1) multiple cause analysis, (2) negative correlation, (3) impairment, and (4) proportional redistribution (Table 1, Fig. 1). Garbage codes are first grouped based on diagnostic relatedness (Additional file 1: Figure 4), afterwards one of these four methods is chosen. The appropriate method is determined on a case-by-case basis, as will be explained in more detail below. Each of these methods independently produces the necessary inputs to redistribution, where garbage-coded deaths are reallocated. Although the underlying algorithm for redistribution, the final step shown in bright green in Fig. 1, has not changed significantly since GBD 2013 [36], substantial improvements were made during GBD 2019 and 2020 to the methods for the steps feeding into redistribution, shown in teal boxes in Fig. 1.

**Table 1:** Number of garbage-coded deaths (and percentage of all garbage-coded deaths) by ICD revision and method of determining redistribution parameters for cause of death data from 1980 to 2019

Method	ICD-9	ICD-10	Total
Multiple cause	18,266,079 (35.1%)	35,096,700 (30.8%)	53,362,779 (32.2%)
Negative correlation	11,711,386 (22.5%)	34,410,369 (30.2%)	46,121,755 (27.8%)
Impairment	209,513 (0.4%)	449,294 (0.4%)	658,807 (0.4%)
Proportional redistribution	21,796,259 (41.9%)	43,851,463 (38.5%)	65,647,722 (39.6%)

### Multiple cause analysis

**Fig. 2:**
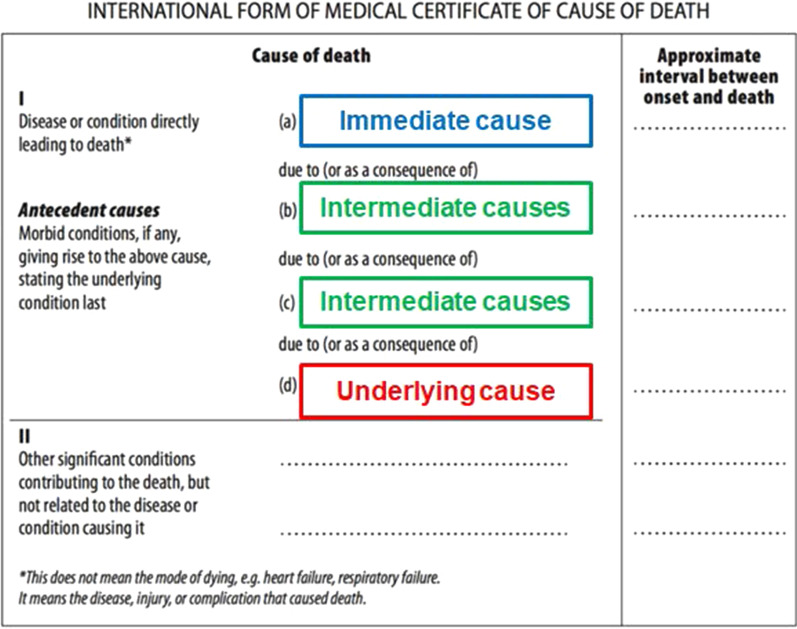
International medical death certificate for cause of death [50]

Death is not a single event, but rather a chain of causal events ultimately leading to death. Multiple cause data, individual-level records listing all causes from the death certificate, include the chain of events leading to death (Part I, Fig. 2) and other significant conditions contributing to mortality, but are not part of the sequence directly leading to death (Part II, Fig. 2) [37].

The chain of events leading to death includes underlying (disease or injury that initiated the events resulting in death), intermediate (events initiated by the underlying cause), and immediate (the terminal event) causes (Fig. 2) [37]. Multiple cause data rarely distinguish intermediate from immediate causes, and therefore we refer to all causes in the chain (i.e., non-underlying causes) on a death certificate as intermediate causes. For example, if a child gets pneumonia, is unable to receive adequate medical attention and then dies of sepsis, we would say the underlying cause of death is pneumonia and sepsis is an intermediate cause. These data are particularly useful to analyze causes that would not otherwise be captured by the underlying cause alone [38], but such data are difficult to obtain due to data privacy issues; Table 2 shows the number of deaths and location-years available for analysis in GBD 2020. As the list of location-years of multiple cause data availability increases, so does our preference for this method over the others presented in this manuscript.

**Table 2:** Data availability for multiple cause analyses

Country	Years	Data source	Deaths	Location years
Austria	2001–2014	Austria Hospital Inpatient Discharges	461,538	14
Brazil	1999–2017	Brazil Mortality Information System	17,398,531	512
Brazil	2015–2016	Brazil Hospital Information System	294,461	52
Canada	1994–2009	Canada Discharge Abstract Database	38,405	16
Colombia	1998–2017	Colombia Vital Statistics	3,676,540	20
Georgia	2014–2014	Georgia Hospital Data	1,066	1
Italy	2003–2015	Italy Civil Registration Multiple Causes of Death	7,640,383	13
Italy	2003–2018	Italy—Friuli-Venezia Giulia Multiple Causes of Death Data	112,555	16
Italy	2005–2016	Italy Hospital Inpatient Discharges	2,385,430	12
Mexico	2003–2005	Mexico Ministry of Health Hospital Discharges	59,597	64
Mexico	2007–2009	Mexico Secretariat of Health Hospital Discharges	108,985	96
Mexico	2009–2016	Mexico Vital Registration—Multiple Causes of Death	4,473,427	256
New Zealand	2000–2015	New Zealand National Minimum Dataset	152,725	32
South Africa	1997–2016	South Africa Vital Registration—Causes of Death	4,696,348	180
Taiwan (Province of China)	2008–2017	Taiwan Vital Registration—Multiple Causes of Death	1,237,304	10
United States of America	1980–2010	United States National Hospital Discharge Survey	180,802	31
United States of America	1980–2016	United States NVSS Custom Mortality Data	68,133,196	1,887
United States of America	2003–2008	United States State Inpatient Databases	1,847,569	70

Multiple cause data available by source and the total number of deaths available for each country and year range. Brazil, Mexico, South Africa, and the United States were analyzed by first administrative level, and New Zealand data were analyzed by Maori and Non-Maori ethnicities

#### Intermediate causes of death

A variety of methods have been previously used to account for intermediate causes incorrectly listed as the UCoD, including multinomial regression, Bayesian regression, and coarsened exact matching [39–42]. We have built on these analyses, and the methods presented here include two key innovations introduced in GBD 2019 and further developed in GBD 2020: (1) determining a set of plausible underlying causes from multiple cause data, rather than relying on literature reviews or expert opinion, and (2) increasing generalizability across all GBD-estimated locations. In GBD 2019, the analysis described in the following paragraphs was introduced to inform the redistribution of deaths incorrectly coded to the following intermediate causes: sepsis; embolism (pulmonary and arterial); heart failure (left, right, and unspecified); acute kidney injury; hepatic failure; acute respiratory failure; pneumonitis; and unspecified central nervous system disorders. In GBD 2020, this list was expanded to include gastrointestinal bleeding; chronic respiratory failure; peritonitis; fluid, electrolyte, and acid–base disorders; arrhythmia; pneumothorax; alcoholic hepatic failure; amyloidosis; cachexia; osteomyelitis; plegia; atherosclerosis; empyema; hypertension; shock, cardiac arrest, and coma; and renal failure.

First, death certificates with a non-garbage UCoD were mapped to corresponding GBD causes and tagged indicating presence of the intermediate cause of interest by ICD code (ICD codes for each intermediate cause can be found in Additional file 1: Figure 8). For all aforementioned intermediate causes except sepsis, Part I and Part II of the death certificate were included for analysis. Records were then aggregated by UCoD, age group, sex, year, location, and intermediate cause presence. The proportion of intermediate cause-related deaths was calculated by dividing the number of intermediate cause-related deaths by total deaths for each demographic group.

Second, we determined the set of the most plausible underlying causes, separately for each intermediate cause. A key feature of redistribution is the selection of the most likely underlying causes of death. Our first approach used all underlying causes appearing in the multiple cause data; however, this resulted in arbitrarily small proportions, e.g., 0.00068% of pulmonary embolism-related deaths due to diphtheria in high-income countries among males between the ages of 15 and 29. To avoid artificial redistribution results, we performed a two-step process to trim the list of underlying causes that will serve as redistribution targets for the garbage coded deaths. First, we keep the underlying causes comprising 80% of deaths in the multiple cause data. Then, a least absolute shrinkage and selection operator (LASSO) regression is used on only the response variable (proportion of intermediate-cause-related deaths) and the underlying causes comprising the bottom 20% of deaths [43]. LASSO adds a penalty, tuned by adjusting the lambda parameter, equal to the absolute value of the magnitude of the coefficients, such that the coefficients on many of the underlying causes were reduced to zero and could be empirically excluded. Related dimension reduction techniques, such as ridge and elastic net regressions, may reduce coefficients, but do not push them to zero, and were therefore not used. The lambda parameter was chosen based on minimization of the cross-validated sum of squared residuals, with 10 folds. The R package “glmnet” was used [44].

After determining the most plausible set of underlying causes for each intermediate cause, we then constructed a predictive model. The proportion of deaths related to the intermediate cause of interest was estimated using a generalized linear model with binomial response and link logit (Eq. 2) using the R package “lme4” [45]

2 $$\begin{aligned} & {\text{Y}}_{i} \sim B\left( {n_{i} , \pi_{i} } \right) \\ & logit\left( {\pi_{i} } \right) = \beta_{0} + \beta_{1} \cdot X + \beta_{age} + \beta_{sex} + \gamma_{underlying\,cause} \\ \end{aligned}$$

where: $Y_{i}$ = the proportion of deaths related to the intermediate cause of interest.

Where the distribution of random variable ${\text{Y}}_{i}$ is binomial, with $n_{i}$ number of observations and probability of an intermediate cause-related death $\pi_{i}$ for each age, sex, location, year, and underlying cause group $i$. $\beta_{0}$ is the global intercept, $\beta_{1}$ is the effect of $X$ covariates, $\beta_{age}$ and $\beta_{sex}$ are the categorical covariates for age group and sex, and $\gamma_{underlying\,cause}$ is the random effect on UCoD. Separate models were run for each intermediate cause of interest, and each set of covariates is listed in the Additional file 1 (Additional file 1: Figure 9), with the most common being Healthcare Access and Quality Index (HAQ Index). The HAQ Index is a measure of amenable mortality informed by mortality rates for a set of 32 causes which should not be fatal given adequate medical treatment [46].

A step-by-step example is given below for sepsis (Eq. 3). Referenced below as “sepsis fraction,” proportions were extrapolated for all GBD locations using the above Eq. 2 and multiplied by GBD 2019 estimated cause-specific deaths to calculate the number of intermediate cause-related deaths for each age, sex, location, year, and underlying cause (step 1 in Eq. 3, below). Intermediate cause-related deaths were then summed to calculate the total intermediate cause-related deaths across all underlying causes (Eq. 3, step 2). Lastly, we calculated the cause fraction for each intermediate cause, with total intermediate‐cause-related deaths as the denominator, by age, sex, location, year, and GBD cause (Eq. 3, step 3).

3 $$\begin{aligned} & 1.\quad sepsis\,deaths_{a,s,l,y,c = } sepsis\,fraction_{a,s,l,y,c} *GBD\,deaths_{a,s,l,y,c } \\ & 2.\quad total\,sepsis\,deaths_{a,s,l,y} = \mathop \sum \limits_{c} sepsis\,deaths_{a,s,l,y,c} \\ & 3.\quad proportion\,of\,sepsis\, to\, redistribute_{a,s,l,y,c} = \frac{{sepsis\, deaths_{a,s,l,y,c} }}{{total\,sepsis\,deaths_{a,s,l,y} }} \\ \end{aligned}$$

where “sepsis fraction” was estimated from the model shown in Eq. 2 and a, s, l, y, c denote a given age group, sex, location, year, and UCoD, respectively.

The resulting proportions from Eq. 3, step 3 were used as inputs to redistribution (Fig. 1). Results from the multiple cause analysis for pulmonary embolism (Additional file 1: Figure 10) and unspecified heart failure (Additional file 1: Figure 11) are shown in the Additional file 1.

#### Unspecified injuries: X59 and Y34

Deaths due to injury are described in the ICD by codes specific to the external cause (e.g., motor vehicle crash) and for the injury diagnosis (e.g., injury to head), also referred to as nature of injury codes [47]. Though it is often easier to identify the nature of injury of a deceased person than the factor that caused the injury, a detailed external injury code is required for correctly assigning the UCoD [48]. Two common non-specific codes for external causes of mortality are exposure to unspecified factors (X59 in ICD-10) and unspecified event of undetermined intent (Y34 in ICD-10) [49]. These codes comprise 2.5% of all garbage-coded deaths and 8.1% of total injuries deaths in ICD-10 VR. To identify proportions and plausible underlying causes for these deaths, we employed a multi-step approach that uses the combination of nature of injury codes in the causal chain in multiple cause data Fig. 2.

First, death certificates in multiple cause data with the garbage code of interest or a GBD injuries cause as the UCoD were selected. The detailed nature of injury codes in the causal chain of these death certificates were collapsed to 37 custom groups of diagnostically related ICD Codes (Additional file 1: Figure 12). For each death, we then identified combinations of nature of injury codes appearing in the chain according to these custom groups. The top 95% of combinations were then used to derive preliminary cause, age, sex, year, and location-specific redistribution proportions. These proportions were derived based on the probability of a given combination being coded to an X59/Y34-related garbage code or a GBD injuries cause and then summed for all combinations. An example is given below for X59 (Eq. 4):

4 $$\begin{aligned} 1.\quad & P_{{(combination_{j} |UCoD\,X59)}} = \frac{{\# of\, combination_{j} \, deaths | UCoD\,X59}}{{\mathop \sum \nolimits_{j = 0}^{m} {(}\# of\, combination_{j} \,deaths {|}UCoD\,X59)}} \\ 2.\quad & P_{{(GBD\,injuries\,cause_{i} |combination_{j} )}} = \\ & \quad \frac{{\# \,of\, UCoD\,GBD\,injuries\,cause_{i} \,deaths| combination_{j} }}{{\sum\nolimits_{i = 0}^{n} {\left( {\# \,of\, UCoD\, GBD\, injuries\, cause_{i} \, deaths {|}combination_{j} } \right)} }} \\ 3.\quad & redistribution\,proportion_{{GBD\,injuries\,cause_{i} }} = \\ & \quad \mathop \sum \limits_{j = 0}^{m} \left( {P{(}combination_{j} {|}UCoDX59} \right)* \\ & \quad P{(}GBD\, injuries\,cause_{i} {|}combination_{j} )) \\ \end{aligned}$$

where: combination_j_ = a given nature of injury code combination in the causal chain; UCoD X59 = a death with X59 coded as the UCoD; UCoD GBD injuries cause_i_ = a death with a given GBD injuries cause *i* coded as the UCoD.

These proportions are based on the specific pattern of injuries in country-years with multiple cause data; they are preliminary and can only be applied to multiple cause data to estimate the fraction of each injury cause that are coded to X59 or Y34. We applied these cause-, age-, sex-, year-, and location-specific redistribution proportions on the data where X59 or Y34 was the UCoD to get the number of unspecified injuries deaths “attributable” to each GBD injuries cause. Then, for each GBD injuries cause in the multiple cause data, we calculated the fraction of redistributed garbage-coded injuries deaths over the fraction of total injuries deaths for that cause and modeled this intermediate cause fraction using a mixed effects linear regression (Eq. 2), same as that used for intermediate causes. As described in detail above for analyzing intermediate causes, unspecified injuries fractions were multiplied by CoD results from the previous GBD round, summed across all GBD injuries causes, and final redistribution proportions were calculated separately for X59 (Additional file 1: Figure 13) and Y34 (Additional file 1: Figure 14) by age, sex, location, year, and GBD injuries cause for use in all CoD data. Results from this analysis are shown in the Additional file 1. An additional, separate example of using multiple cause data to redistribute misclassification of accidental poisoning can be found in the Additional file 1 (Additional file 1: Figure 15).

### Negative correlation

While multiple cause analysis is the preferred method of determining underlying cause targets and proportions for garbage codes, this method is not possible for class 4, the most specific garbage-coded deaths (e.g., malignant neoplasm of ill-defined digestive organs). This is because a death certificate would never include a more detailed ICD code nested within a less detailed code; for example “malignant neoplasm of ill-defined digestive organs” and “liver cancer”. In these instances of class 4 garbage, there is a noticeable inverse relationship between the garbage-coded death and its plausible underlying causes of death, i.e., as the number of garbage-coded deaths increases, the number of deaths due to plausible underlying causes decreases. Thus, we use a negative correlation method to determine how to redistribute these deaths (Fig. 1). First described by Ahern et al. [50] for the redistribution of unspecified heart failure, this method assumes that with improvements in coding practices, more deaths are assigned to the plausible underlying cause(s) and fewer to the corresponding garbage codes. The detailed methods for negative correlation redistribution have been described elsewhere [2]. In GBD 2019, the core methods for negative correlation redistribution were revisited, and a slightly different approach was adopted to redistribute deaths attributed to unspecified diabetes, unspecified stroke, and malignant neoplasm without specification of site. Using unspecified stroke as an example, these methods are summarized in brief here.

The corresponding plausible underlying causes of death for unspecified stroke are assumed a priori to be the subtypes ischemic stroke, intracerebral stroke, and subarachnoid stroke. Shown in Eq. 5 below, we assume the logit-transformed proportion of each stroke subtype (out of all non-garbage-coded stroke deaths), $\mu_{i} ,$ can be modeled linearly as a function of covariates predictive of stroke mortality, $\beta_{1} X_{i}$, with intercept $\beta_{0}$ for each age, sex, location, and year group $i$.

5 $$\begin{aligned} & logit {\text{ X}}_{i} \sim {\text{ N}}\left( {\mu_{i} , \sigma^{2} } \right) \\ & \mu_{i} = \beta_{0} + \beta_{1} X_{i} \\ \end{aligned}$$

In an ideal world, the method would conclude after the aforementioned regression (Eq. 5). In practice, however, we noticed bias in the residuals with respect to the proportion of unspecified stroke in all stroke related deaths. To account for these biases, we apply an adjustment, which is made in two steps. First, residuals from the regression (Eq. 5) are calculated and regressed against the logit-transformed proportion of deaths coded to unspecified stroke in order to identify any trend present between the residuals and the proportion of deaths garbage-coded to unspecified stroke. Second, the adjustment is calculated using the slope of this regression line, and the difference between the value of the residuals when no deaths are garbage-coded to unspecified stroke and at the observed proportion of deaths coded to unspecified stroke (Eq. 6). Ideally, the proportion of unspecified stroke would not influence the model and regressing the residuals against the proportion of unspecified stroke would show little correlation with a slope near 0. The adjustment would then be quite small. However, stronger correlation between the residuals and the proportion of unspecified stroke results in a larger adjustment being necessary.

6 $$\begin{aligned} & 1.\quad residuals_{i} = \beta_{0} + \beta_{1} *logit\left( {GC} \right)_{i} \\ & 2.\quad adjustment_{i} = \beta_{1} *logit\left( {No GC} \right) - \beta_{1} *logit\left( {GC} \right)_{i} \\ \end{aligned}$$

where: $i$ = each age, sex, location, year group; $residuals_{i} =$ difference in observed and predicted values from model fit in Eq. 5 (the regression line); $\beta_{1}$ = slope of the relationship between $residuals_{i}$ and $logit\left( {GC} \right)$; $\beta_{0}$ = y-intercept; $logit\left( {GC} \right)_{i}$ = logit-transformed proportion of all stroke-related deaths coded to unspecified stroke; $logit\left( {No GC} \right)$ = y-intercept (in logit space) where all deaths are coded to specific stroke subtype (i.e., no deaths are coded to unspecified stroke).

This adjustment is added to the initially estimated proportion of a given stroke subtype generated by Eq. 5, bringing it closer to the true proportion of a world without garbage coding. Proportions are normalized by age, sex, location, and year.

Since the residuals are modeled on logit(GC%), it is not possible to calculate the adjustment for GC% = 0%. Instead, we used GC% = 1% to represent the counterfactual of “no garbage.” The same methods are applied to the redistribution of unspecified diabetes and malignant neoplasm without specification of site. We therefore combine two approaches—descriptive linear modeling with covariates explanatory of mortality and an adjustment for coding practices—to produce improved estimates as compared to previous GBD cycles.

### Impairments

The GBD defines impairments as domains of health loss that are a consequence of multiple underlying causes, rather than underlying causes of death themselves [2]. Anaemia, for example, can occur as the result of chronic kidney disease or malaria, but is not considered the UCoD. Due to the difficulty in identifying a single underlying cause for impairments, neither a multiple cause analysis nor the negative correlation method is possible, and instead we rely on the non-fatal burden estimation process of GBD [2]. The resulting years lived with disability (YLDs) [2] are used to calculate redistribution proportions and to determine a plausible set of underlying causes of death for impairments (Fig. 1).

Plausible underlying causes are restricted to causes that have years of life lost (YLLs) attributed to them rather than exclusively YLDs, i.e. causes from which a person can conceivably die. Proportions are calculated by dividing the number of cause-specific YLDs for a given impairment by the sum of YLDs across all causes for each age group, sex, location, and year. Locations with a star rating > 3 have country-specific proportions, while countries with a star rating ≤ 3 are assigned region-level proportions. GBD 2020 redistribution of anemia and pelvic inflammatory disease relied on the results of the non-fatal GBD 2019 estimation process. Proportions and underlying causes are then used as inputs to redistribute garbage-coded CoD data (Fig. 1). In the GBD 2020 study, 0.4% of garbage-coded deaths across all years were incorrectly assigned to impairments, rather than to the appropriate UCoD, prior to redistribution (Table 1).

### Proportional redistribution

**Fig. 3:**
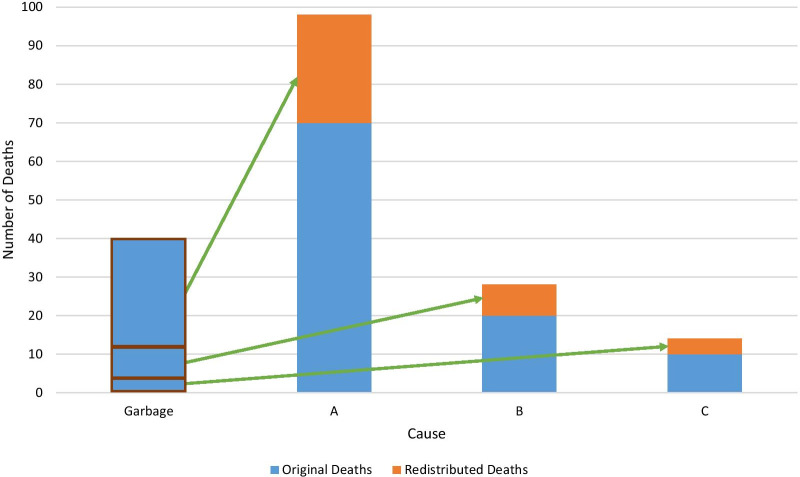
Conceptual diagram of garbage-coded deaths being redistributed proportionally onto plausible underlying causes A, B, and C. Deaths are reallocated separately for each age, sex, location, and year of cause of death data

Unlike the other processes outlined above, where we use external data sources to define a set of proportions for redistribution, proportional redistribution reallocates garbage-coded deaths to be directly proportional to the distribution of plausible underlying causes of death in the non-garbage-coded deaths in the CoD data, separately by age group, sex, location, and year, as shown in Fig. 3.

The key assumption of proportional redistribution is that garbage coding is independent of underlying cause: every underlying cause targeted by proportional redistribution for a given garbage code is equally likely to be miscoded. We use this method when the distribution of non-garbage-coded deaths is plausible and there are enough non-garbage-coded deaths to inform the post-redistribution cause pattern (Fig. 1). Proportional redistribution is only used for the least specific class 1 garbage-coded deaths, e.g., “all ill-defined,” the set of plausible causes includes all non-garbage-coded deaths in the data. Whereas for more detailed class 3 garbage codes, e.g., unspecified upper respiratory infections, the set of underlying causes is determined a priori based on clinical knowledge. Proportional redistribution was used for 11.6% of all ICD-9 and -10 coded VR deaths and 39.6% of garbage-coded deaths in CoD data from 1980 to 2019 (Table 1).

### Role of the funding source

The funders of the study had no role in study design, data collection, data analysis, data interpretation, or writing of the report. The corresponding author had full access to all the data in the study and final responsibility for the decision to submit for publication.

## Results

**Fig. 4:**
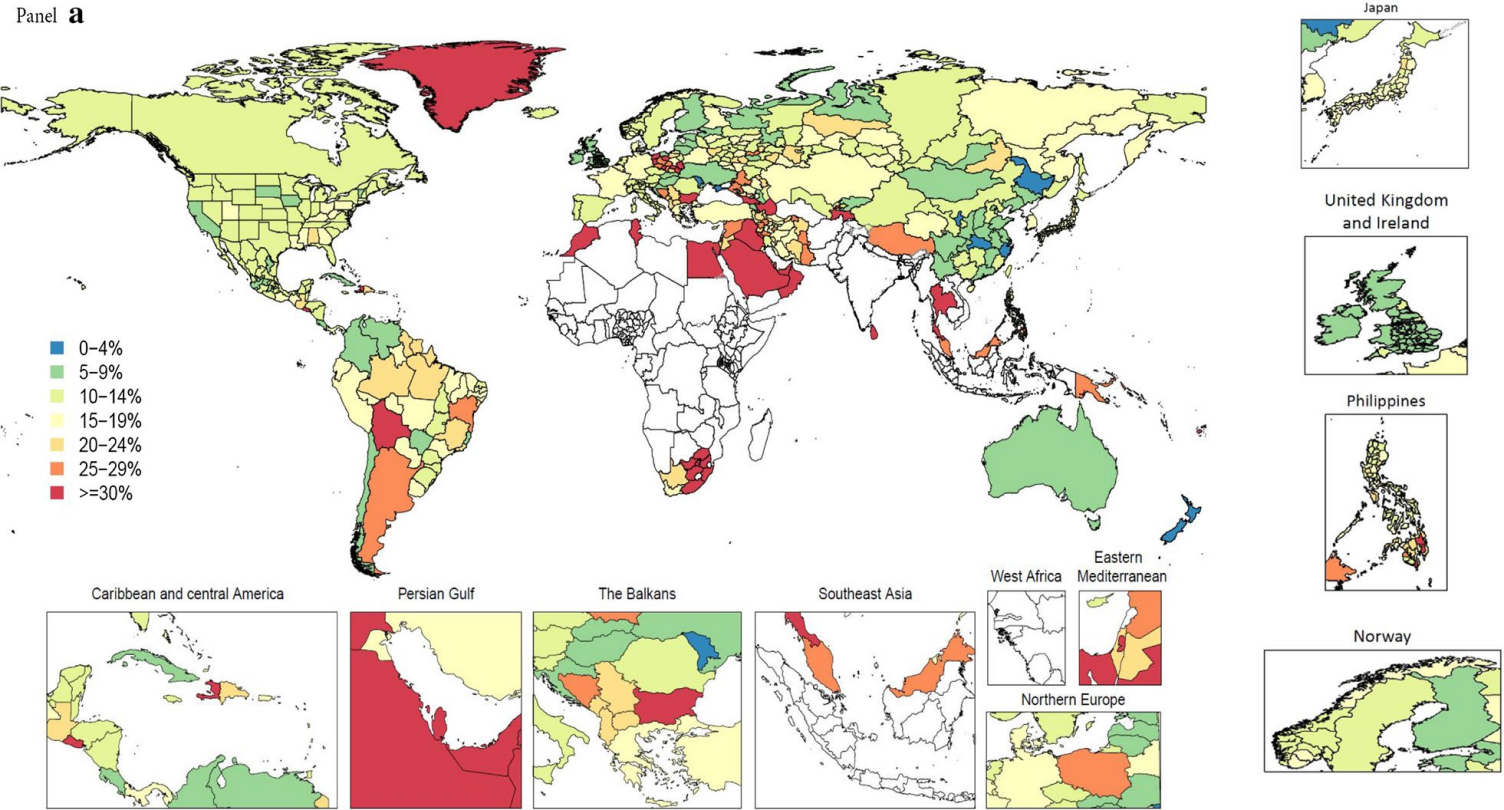

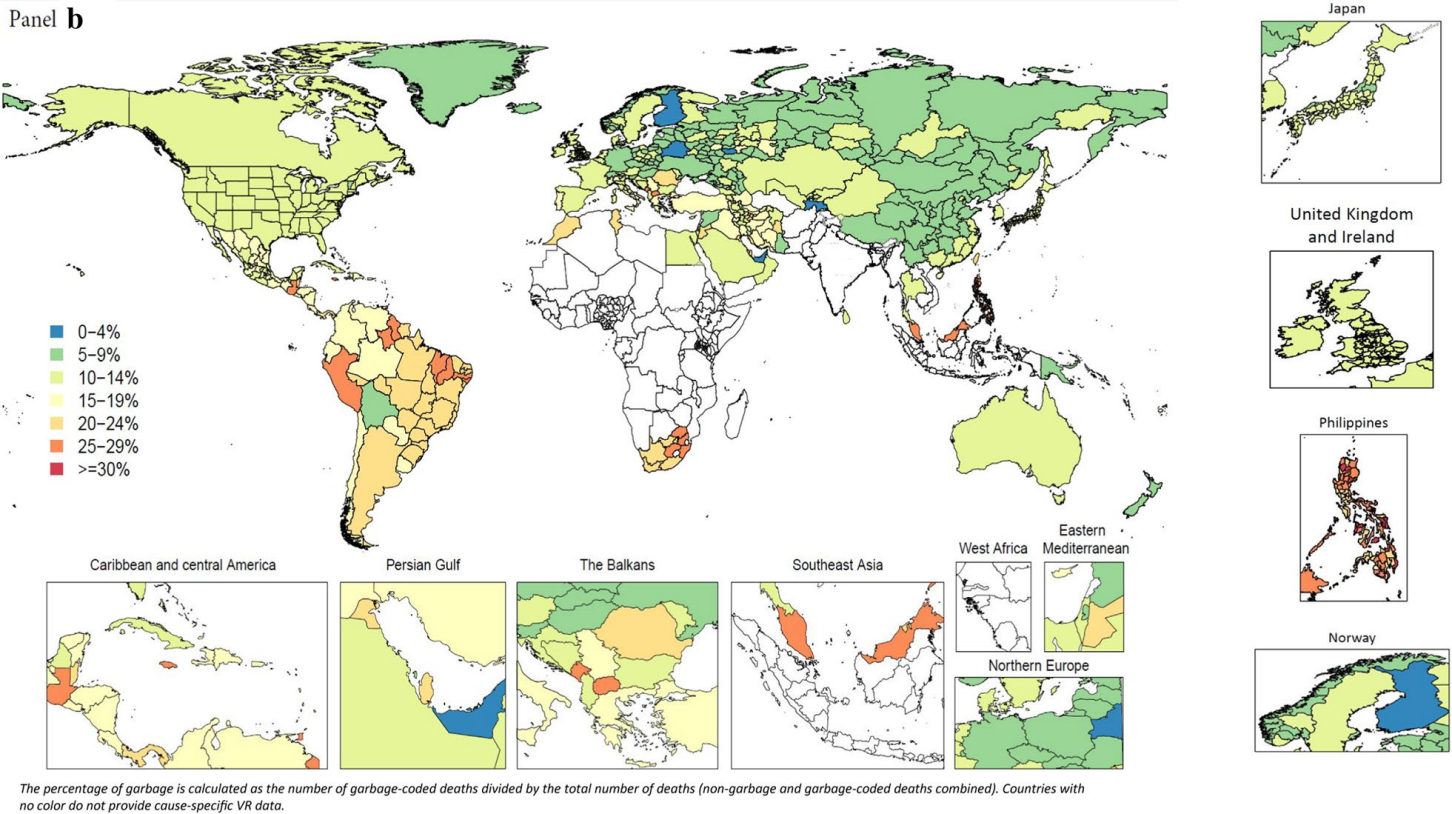
Percentage of major, class 1 and 2 (**a**), and class 3 and 4 garbage (**b**) in VR data in 2015 or closest available year, all ages, both sexes

The percentage of garbage-coded deaths out of all deaths in VR data varied widely across locations and by garbage code class. In VR data for the year 2015 (or the most recent year available by location), for example, deaths coded to major (class 1 or 2) garbage codes spanned a wider range across locations (from a low of 3.7% to a high of 67.3%) compared to the percentage of deaths coded to more detailed (class 3 and 4) garbage codes, which ranged from 2.4% to 34.6% (Fig. 4). Additional stratification of the percentage of garbage-coded deaths for each class is presented in the Additional file 1 (Additional file 1: Figure 16). Results in Fig. 4 are shown for the year 2015 in order to maximize the data availability across locations because the overall level of garbage coding does not change substantially over time (Additional file 1: Figure 5). There is also substantial subnational variation in the proportion of deaths coded to class 1 or 2 garbage codes. In 2015, subnational variation was largest in Russia, from 5.1% in Jewish autonomous oblast to 27.7% in Rostov oblast, and in Brazil, ranging from 8.5% in Espírito Santo to 29.5% in Bahia. Some countries, such as Japan, Norway, and the UK, had very little variation in proportion of deaths coded to class 1 or 2 garbage codes, compared to countries with relatively more variation, such as the Philippines.

**Fig. 5:**
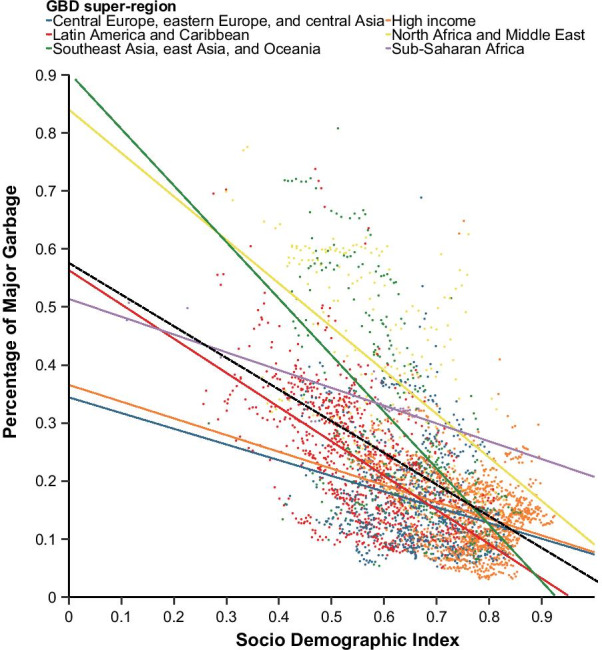
Age-standardised proportion of major garbage vs. SDI by location and year, 1980–2019. The dashed black line represents the global trend

The portion of age-standardized deaths coded to major garbage, out of all deaths, decreases as a location’s Socio-Demographic Index (SDI) increases (Fig. 5). The SDI value serves as an indicator of development status and is a value between 0 and 1 calculated from three components: fertility rate, income per capita, and average educational attainment. More information on the SDI and how it is calculated is described elsewhere [33]. This relationship between SDI and age-standardized major garbage is true at the global level and in each GBD super-region, although it is less pronounced in some regions, such as sub-Saharan Africa. Using the age-standardized, rather than all-age, proportion of major garbage as a metric is more useful for inter-country comparisons because the percentage of garbage-coded deaths is often higher in locations with larger elderly populations.

**Fig. 6:**
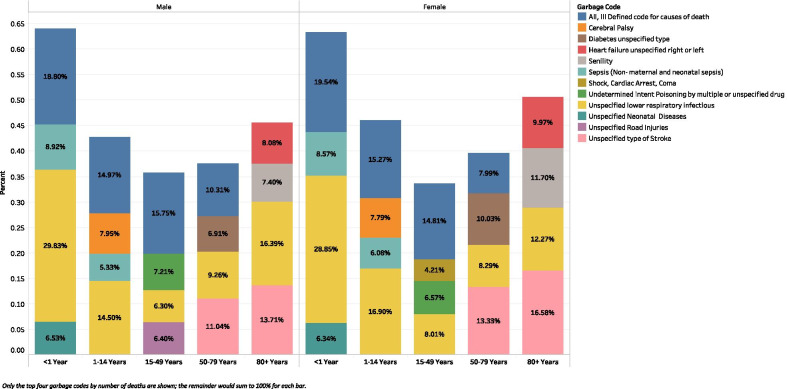
Stacked bar chart of the top four garbage codes, by percentage of all garbage-coded deaths, for ICD-10 VR data in 2015 by age and sex

**Fig. 7:**
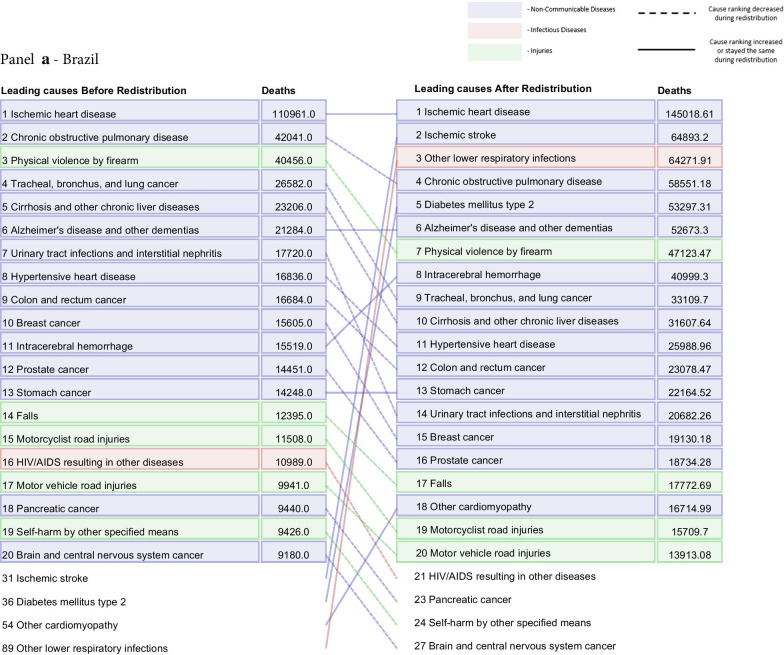

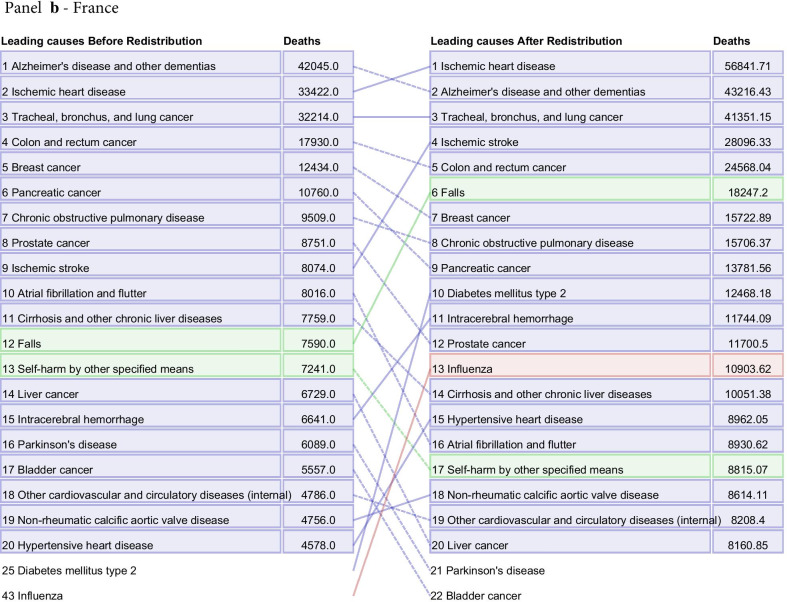

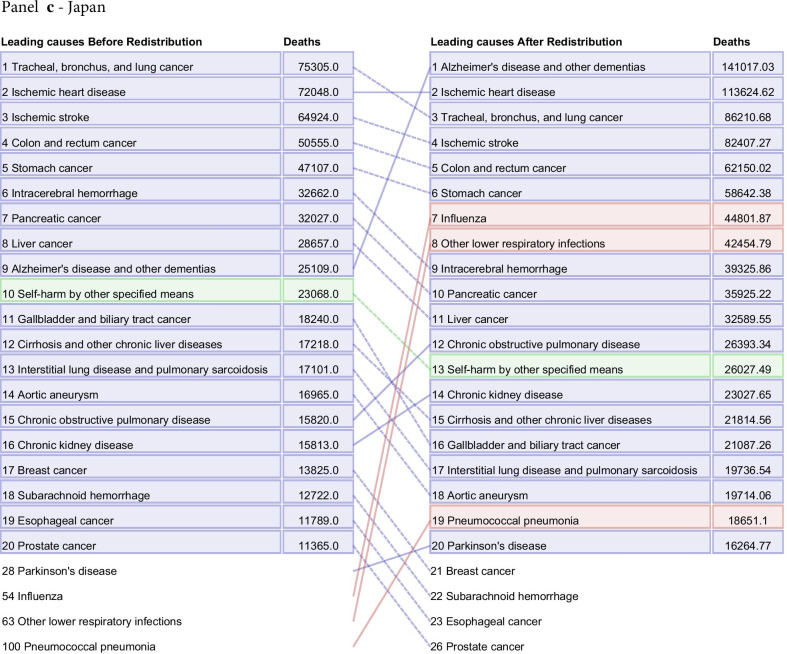

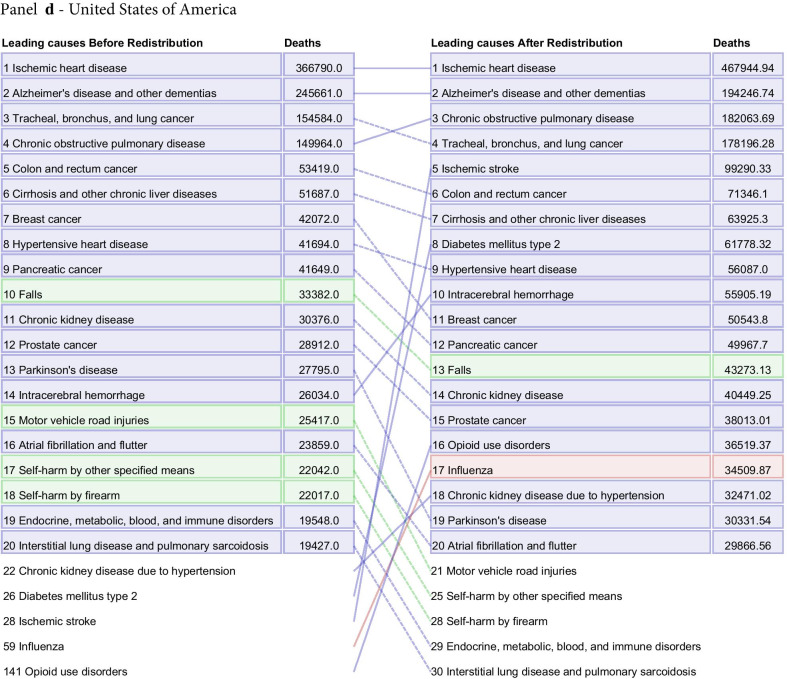
Leading 20 causes of death for Brazil (**a**), France (**b**), Japan (**c**), and the United States (**d**) in 2015 for all ages and both sexes combined. The left-hand column is data before redistribution compared to data after redistribution in the right-hand column. Causes are connected by arrows before and after redistribution. Infectious diseases are shown in red, non-communicable diseases in blue, and injuries in green. This figure also captures additional corrections applied prior to redistribution, namely adjustments made for the misdiagnosis of Parkinson’s, atrial fibrillation, and Alzheimer’s disease and other dementias not discussed in detail in this paper (Additional file 1: Figure 1). Additionally, only real underlying causes are included in this figure. For that reason, one will not see "Garbage Code" listed in the deaths prior to redistribution

In addition to geographic variation, the garbage codes that comprise the most deaths vary across age groups. In those under 1 year of age in 2015, unspecified lower respiratory infections accounted for the largest proportion of garbage-coded deaths, out of all garbage-coded deaths, for both males and females (Fig. 6), compared to unspecified stroke for both sexes in the 50 to 79 age range. There was also some variation by sex and age: in those aged 80 and over, most garbage-coded deaths were attributable to unspecified lower respiratory infections in males, compared to unspecified stroke in females. While Fig. 6 depicts the most frequent garbage codes at the global level, there is notable variation in garbage code prevalence by location. More information on country-specific leading garbage codes can be found in the Additional file 1 (Additional file 1: Figure 17). Similar to Fig. 4, results in both Fig. 6 and the following Fig. 7 are shown for the year 2015 in order to maximize the data availability across locations.

The process of redistribution affects the number of deaths assigned to different causes, the cause fraction, and the corresponding mortality rates. The effect of redistribution can be large, and results in changes in the rankings of the top causes of death by location, age, and sex. At the national level, redistribution of garbage codes can substantially change the rankings of the top 10, 20, and 50 causes of death. Figure 7 highlights the change in ranking by total deaths of the top 20 underlying causes before and after redistribution for Brazil, France, Japan, and the US in 2015, combined for all age groups and both sexes. These four countries were selected for illustrative purposes, and underlying cause rankings for all other countries and territories estimated by GBD can be found in Additional file 1: Figure 18. In Brazil, France, and the US, there were large increases in the rank of ischemic stroke after redistribution, from 31st to second, ninth to fourth, and 28th to fifth, respectively. Deaths due to diabetes mellitus type 2 increased 4.0-fold in the US and 10.6-fold in Brazil after redistribution. Notably, in Japan, Alzheimer’s disease and other dementias rose from the ninth-ranked UCoD to the first in terms of number of deaths. In Japan, large increases in the rank of deaths due to influenza, pneumococcal pneumonia, and other lower respiratory infections occurred. Of our exemplars, the US is the only country shown where redistribution resulted in a large increase in the rank of drug use disorders, with opioid use disorders jumping in rank from 141^st^ to 16th following redistribution. France was the only country of the four to have an injuries-related cause move into the top 10 after redistribution, with deaths due to falls ranked sixth, increasing from 7,590 assigned deaths to 18,247 assigned deaths in 2015.

## Discussion

We have described the four methods for redistributing garbage-coded VR deaths in the GBD: (1) multiple cause analysis, (2) negative correlation, (3) impairments, and (4) proportional redistribution (Fig. 1). Overall, the methods introduced here reflect an improvement in empiricism of redistribution methods; for less-detailed garbage, rather than relying on a priori selection of plausible underlying causes and proportions, we have sought out alternative methods and data sources. Notably, this study provides the first in-depth explanation of the incorporation of multiple cause data to inform redistribution for 32.2% of garbage-coded deaths in GBD 2020 (Table 1).

The change in ranking among the top 20 underlying causes of death by number of deaths before and after redistribution highlights the necessity of redistribution of garbage-coded deaths to understand a country’s actual cause-specific mortality pattern (Fig. 7). This figure also captures the effects of misdiagnosis corrections, a process outside the scope of this paper that has been described previously [3]. Redistribution is not the ideal solution for the problem of garbage-coded deaths, however: ultimately, higher-quality CoD data in all locations is needed to provide accurate information on mortality patterns and inform public health decision making.

Interventions to increase the quality of cause of death coding must be context-specific. We have shown that the proportion of major garbage has not varied dramatically over time (Additional file 1: Figure 5), but rather by SDI, with countries with lower SDI having higher proportions of age-standardized garbage (Fig. 5). When contrasting the proportion of major garbage versus more detailed garbage codes, however, there is substantial intra- and inter-country variation (Fig. 4). These deaths coded to classes 1 and 2 have the most substantial health policy implications, as they can mislead policy makers on the overall mortality composition in a population, as well as on the importance of various leading causes of death within a disease category [35]. Deaths coded to classes 3 and 4 can hamper prevention and treatment efforts because they do not distinguish between subtypes of a disease. National-level policy interventions have been shown to increase death registration (including ascertainment of a CoD) [51]. Specifically, enhanced training efforts led by the Bloomberg Data for Health Initiative, where physicians and instructors leading ICD-compliant certification courses received targeted training, has dramatically improved the number of correctly filled out death certificates in locations including the Philippines, Sri Lanka, and Peru [52]. Such interventions have decreased the number of deaths coded to class 1, 2, or 3 garbage; however, reducing deaths coded to the most specific, class 4 garbage often requires more expensive medical technology. Diagnosis of ischemic versus hemorrhagic stroke, for example, requires computed tomography scanners, which are often unavailable in low-resource settings [53].

The methods described here have a number of limitations. First, the scope of this paper has been limited to countries sharing VR data for use in the GBD; countries without color in Fig. 4 are therefore excluded from the methods presented here. Second, for the multiple cause analysis, the primary explanatory variable in the majority of the models which is used to predict the proportion of intermediate-cause-related deaths for all GBD-estimated locations is the HAQ Index. The inclusion of additional explanatory covariates, additional sources of multiple cause data to support these covariates, and empirical covariate selection is crucial for strengthening the predictive validity of estimates. Third, the multiple cause analysis has circular dependencies, as the proportions used to redistribute garbage-coded deaths rely on GBD cause-specific mortality estimates. If, for example, our redistribution proportions for unspecified heart failure overestimate mortality due to a given CoD, then the overall level of estimated mortality will increase for that cause, and this effect will continue to be perpetuated in subsequent GBD rounds. Solutions to reduce the circularity in generation of results are being explored. Fourth, in the case of proportional redistribution, we make a strong assumption that the assignment of garbage is independent from the underlying cause. We hope to improve this method in the future, with the incorporation of data where death certificates are linked with hospital admissions. Lastly, we want to acknowledge that the term “garbage” codes may be viewed as punitive; renaming has been discussed within the GBD; however, for this manuscript we have opted to maintain it to be consistent with other publications on this topic.

In addition to continually seeking out additional multiple cause of death data, we are currently working to improve the methods used to redistribute unspecified injuries X59 and Y34 garbage codes. We are in the process of implementing machine-learning algorithms to improve upon the algebra-based method described above for generating cause-, age-, sex-, and year-specific redistribution proportions for X59 and Y34. Furthermore, we would like to align our measure of data quality with the more comprehensive Vital Statistics Performance Index (VSPI). While VSPI and the current star ranking of data quality both incorporate measures of completeness and proportion of garbage-coded deaths, VSPI includes additional measures such as proportion of deaths without age or sex detail and timeliness of data reporting. Producing VSPI as a data quality indicator would also align the GBD with other efforts to produce comparable metrics of data quality [54]. Lastly, we welcome future collaborations to analyze country-specific explanations behind many of the descriptive analyses produced here.

## Conclusions

In an ideal world, CoD certification and coding practices would be consistent and accurate across space and time, and there would be no need for garbage code redistribution. In the absence of such standardized practices, the GBD uses redistribution methods on garbage-coded deaths in order to provide the most comprehensive set of cause of death-specific mortality estimates and enable precision in public health decision making. These methods continue to be updated and improved as new strategies and data sources become available.

## Supplementary Information

**Additional file 1**. Supplementary figures and tables.

## Data Availability

The datasets supporting the conclusions of this article are available from the corresponding author upon request.

## Abbreviations

CMNN: Communicable, maternal, neonatal, and nutritional
CoD: Cause of death
GATHER: Guidelines for Accurate and Transparent Health Estimates Reporting
GBD: Global Burden of Disease
HAQ: Healthcare Access and Quality
ICD: International Classification of Diseases
LASSO: Least absolute shrinkage and selection operator
NCD: Non-communicable disease
PWC: Percent well certified
SDI: Socio-Demographic Index
UCoD: Underlying cause of death
VR: Vital registration
VSPI: Vital Statistics Performance Index
YLD: Years lived with disability
YLL: Years of life lost

## References

[1] Alter GC, Carmichael AG (1999). Classifying the dead: toward a history of the registration of causes of death. J Hist Med Allied Sci.

[2] GBD 2019 Diseases, Injuries, and Impairments Collaborators. Global burden of 369 diseases and injuries in 204 countries and territories, 1990–2019: a systematic analysis for the Global Burden of Disease Study 2019. The Lancet. in press 10.1016/S0140-6736(20)30925-9 PMC7567026 33069326

[3] Principles and Recommendations for a Vital Statistics System Revision 2 [Internet]. United Nations; 2001 [cited 2020 May 29]. https://unstats.un.org/unsd/publication/SeriesM/SeriesM_19rev2E.pdf

[4] Sibai AM (2004). Mortality certification and cause-of-death reporting in developing countries. Bull World Health Organ.

[5] Jha P (2014). Reliable direct measurement of causes of death in low- and middle-income countries. BMC Med.

[6] AbouZahr C, Boerma T (2005). Health information systems: the foundations of public health. Bull World Health Organ.

[7] Ruzicka LT, Lopez AD (1990). The use of cause-of-death statistics for health situation assessment: national and international experiences. World Health Stat Q Rapp Trimest Stat Sanit Mond.

[8] World Health Organization, editor. International statistical classification of diseases and related health problems. 10th revision, 2nd edition. Geneva: World Health Organization; 2004

[9] Barber JB (1992). Improving accuracy of death certificates. J Natl Med Assoc.

[10] Campos-Outcalt D (2005). Cause-of-death certification: not as easy as it seems. J Fam Pract.

[11] Lakkireddy DR, Basarakodu KR, Vacek JL, Kondur AK, Ramachandruni SK, Esterbrooks DJ (2007). Improving death certificate completion: a trial of two training interventions. J Gen Intern Med.

[12] Naghavi M, Makela S, Foreman K, O’Brien J, Pourmalek F, Lozano R (2010). Algorithms for enhancing public health utility of national causes-of-death data. Popul Health Metr.

[13] Mathers CD, Fat DM, Inoue M, Rao C, Lopez AD (2005). Counting the dead and what they died from: an assessment of the global status of cause of death data. Bull World Health Organ.

[14] Rudd K, Johnson S, Agesa K, Shackelford K, Tsoi D, Kievlan D (2020). Global, regional, and national sepsis incidence and mortality, 1990–2017. The Lancet.

[15] Hernández B, Ramírez-Villalobos D, Romero M, Gómez S, Atkinson C, Lozano R (2011). Assessing quality of medical death certification: Concordance between gold standard diagnosis and underlying cause of death in selected Mexican hospitals. Popul Health Metr.

[16] Rao C, Lopez AD, Yang G, Begg S, Ma J (2005). Evaluating national cause-of-death statistics: principles and application to the case of China. Bull World Health Organ.

[17] Lu TH, Lee MC, Chou MC (2000). Accuracy of cause-of-death coding in Taiwan: types of miscoding and effects on mortality statistics. Int J Epidemiol.

[18] de Lima RB, Frederes A, Marinho MF, da Cunha CC, Adair T, França EB (2019). Investigation of garbage code deaths to improve the quality of cause-of-death in Brazil: results from a pilot study. Rev Bras Epidemiol.

[19] Ellingsen CL, Ebbing M, Alfsen GC, Vollset SE (2018). Injury death certificates without specification of the circumstances leading to the fatal injury—the Norwegian Cause of Death Registry 2005–2014. Popul Health Metr.

[20] Metcalf P, Meyer M, Suchindran C, Heiss G (2016). Assessment of a regression method to reclassify deaths attributable to heart failure. Glob J Health Sci.

[21] Danilova I, Shkolnikov VM, Jdanov DA, Meslé F, Vallin J (2016). Identifying potential differences in cause-of-death coding practices across Russian regions. Popul Health Metr.

[22] Qaddumi JAS, Nazzal Z, Yacoub A, Mansour M (2018). Physicians’ knowledge and practice on death certification in the North West Bank, Palestine: across sectional study. BMC Health Serv Res.

[23] Madadin M, Alhumam AS, Bushulaybi NA, Alotaibi AR, Aldakhil HA, Alghamdi AY (2019). Common errors in writing the cause of death certificate in the Middle East. J Forensic Leg Med.

[24] Teixeira RA, Naghavi M, Guimarães MDC, Ishitani LH, França EB, Teixeira RA (2019). Quality of cause-of-death data in Brazil: garbage codes among registered deaths in 2000 and 2015. Rev Bras Epidemiol.

[25] GBD 2019 Risk Factors Collaborators. The unfulfilled promise of prevention: the global burden of 87 risk factors, 1990–2019; a systematic analysis for the Global Burden of Disease Study 2019. Lancet Press.

[26] GBD 2019 Demographics Collaborators. Global, regional, and national age-sex-specific fertility, mortality, and population estimates, 1950–2019: a comprehensive demographic analysis for the Global Burden of Disease Study 2019. Lancet. 2020;in press. 10.1016/S0140-6736(20)30977-6 PMC7566045 33069325

[27] Phillips DE, Lozano R, Naghavi M, Atkinson C, Gonzalez-Medina D, Mikkelsen L (2014). A composite metric for assessing data on mortality and causes of death: the vital statistics performance index. Popul Health Metr.

[28] Stevens GA, Alkema L, Black RE, Boerma JT, Collins GS, Ezzati M (2016). Guidelines for accurate and transparent health estimates reporting: the GATHER statement. PLOS Med.

[29] R Core Team. R: A language and environment for statistical computing. [Internet]. Vienna, Austria: R Foundation for Statistical Computing; 2019. http://www.R-project.org/

[30] Python Software Foundation. Python Language Reference, version 3.0. [Internet]. Python.org. [cited 2021 Mar 3]. https://www.python.org/

[31] WHO|Exposing misclassified HIV/AIDS deaths in South Africa [Internet]. WHO. World Health Organization; 2020 [cited 2020 May 29]. http://www.who.int/bulletin/volumes/89/4/11-086280/en/10.2471/BLT.11.086280 PMC3066530 21479092

[32] Groenewald P, Nannan N, Bourne D, Laubscher R, Bradshaw D (2005). Identifying deaths from AIDS in South Africa. AIDS.

[33] GBD 2017 Causes of Death Collaborators (2018). Global, regional, and national age-sex-specific mortality for 282 causes of death in 195 countries and territories, 1980–2017: a systematic analysis for the Global Burden of Disease Study 2017—The Lancet. Lancet.

[34] Naghavi M, Abajobir AA, Abbafati C, Abbas KM, Abd-Allah F, Abera SF (2017). Global, regional, and national age-sex specific mortality for 264 causes of death, 1980–2016: a systematic analysis for the Global Burden of Disease Study 2016. The Lancet.

[35] Naghavi M, Richards N, Chowdhury H, Eynstone-Hinkins J, Franca E, Hegnauer M (2020). Improving the quality of cause of death data for public health policy: are all ‘garbage’ codes equally problematic?. BMC Med.

[36] Global, regional, and national age–sex specific all-cause and cause-specific mortality for 240 causes of death, 1990–2013: a systematic analysis for the Global Burden of Disease Study 2013. The Lancet. 2015;385(9963):117–71. 10.1016/S0140-6736(14)61682-2 PMC4340604 25530442

[37] Kircher T, Anderson RE (1987). Cause of death. Proper completion of the death certificate. JAMA.

[38] Puffer RR (1989). New approaches for epidemiologic studies of mortality statistics. Bull Pan Am Health Organ.

[39] Foreman KJ, Naghavi M, Ezzati M (2016). Improving the usefulness of US mortality data: new methods for reclassification of underlying cause of death. Popul Health Metr.

[40] Snyder ML, Love S-A, Sorlie PD, Rosamond WD, Antini C, Metcalf PA (2014). Redistribution of heart failure as the cause of death: the Atherosclerosis Risk in Communities Study. Popul Health Metr.

[41] Stevens GA, King G, Shibuya K (2010). Deaths from heart failure: using coarsened exact matching to correct cause-of-death statistics. Popul Health Metr.

[42] Murray CJL, Dias RH, Kulkarni SC, Lozano R, Stevens GA, Ezzati M (2008). Improving the comparability of diabetes mortality statistics in the U.S. and Mexico. Diabetes Care.

[43] Tibshirani R (1996). Regression shrinkage and selection via the Lasso. J R Stat Soc Ser B Methodol.

[44] Friedman JH, Hastie T, Tibshirani R (2010). Regularization paths for generalized linear models via coordinate descent. J Stat Softw.

[45] Bates D, Mächler M, Bolker B, Walker S (2015). Fitting linear mixed-effects models using lme4. J Stat Softw.

[46] Fullman N, Yearwood J, Abay SM, Abbafati C, Abd-Allah F, Abdela J (2018). Measuring performance on the Healthcare Access and Quality Index for 195 countries and territories and selected subnational locations: a systematic analysis from the Global Burden of Disease Study 2016. The Lancet.

[47] Injury Data and Resources - ICD Injury Matrices [Internet]. 2019 [cited 2020 Jun 18]. https://www.cdc.gov/nchs/injury/injury_matrices.htm

[48] World Health Organization. Injury surveillance guidelines. 2001;(WHO/NMH/VIP/01.02). https://apps.who.int/iris/handle/10665/42451

[49] ICD-10 Version:2019 [Internet]. 2020 [cited 2020 Jun 18]. https://icd.who.int/browse10/2019/en#/Y34

[50] Ahern RM, Lozano R, Naghavi M, Foreman K, Gakidou E, Murray CJ (2011). Improving the public health utility of global cardiovascular mortality data: the rise of ischemic heart disease. Popul Health Metr.

[51] Suthar AB, Khalifa A, Yin S, Wenz K, Fat DM, Mills SL (2019). Evaluation of approaches to strengthen civil registration and vital statistics systems: A systematic review and synthesis of policies in 25 countries. PLOS Med.

[52] Hart JD, Sorchik R, Bo KS, Chowdhury HR, Gamage S, Joshi R (2020). Improving medical certification of cause of death: effective strategies and approaches based on experiences from the Data for Health Initiative. BMC Med.

[53] WHO|Stroke: a global response is needed [Internet]. WHO. World Health Organization; 2020 [cited 2020 Jun 29]. http://www.who.int/bulletin/volumes/94/9/16-181636/en/

[54] Mikkelsen L, Moesgaard K, Hegnauer M, Lopez AD (2020). ANACONDA: a new tool to improve mortality and cause of death data. BMC Med.

