# Supplementary material for: Public health utility of cause of death data: applying empirical algorithms to improve data quality
Source: BMC Med Inform Decis Mak. 2021 Jun 2;21:175. doi: 10.1186/s12911-021-01501-1 (PMC8170729; doi:10.1186/s12911-021-01501-1)
Supplement: Supplementary file 1 — Additional file 1. Supplementary figures and tables. [file 12911_2021_1501_MOESM1_ESM.pdf]

Supplemental Appendix: Public health utility of cause of death data: applying empirical algorithms to improve data quality

Table of Contents

Supplemental Figures…………………………………………………………………….…. 2

Appendix Figure 1: Yearly VR deaths by coding system…………………………... 2

Appendix Figure 2: Causes of death data process overview………………………... 3

Appendix Figure 3: ICD code to GBD cause mapping (ICD9 and ICD10)………... 4

Appendix Figure 4: ICD Codes by garbage package and class…………………… 35

Appendix Figure 5: Major garbage totals by year (ICD9 and ICD10 VR)………. 41

Appendix Figure 6: PWC to star mappings……………………………………….. 42

Appendix Figure 7: VR data quality star ratings 2010-2019 (ICD9 and ICD10)... 43

Appendix Figure 8: Intermediate cause ICD mappings (ICD9 and ICD10)……... 44

Appendix Figure 9: Covariates used in MCOD analysis……………………….…. 46

Appendix Figure 10: Redistribution proportions, Pulmonary Embolism………... 49

Appendix Figure 11: Redistribution proportions, Unspecified Heart Failure…... 52

Appendix Figure 12: N-code ICD mappings (ICD9 and ICD10)……………….... 55

Appendix Figure 13: Redistribution proportions, Unspecified Factor X59……... 59

Appendix Figure 14: Redistribution proportions, External Causes UDI Y34…... 62

Appendix Figure 15: Redistribution proportions, Accidental Poisoning……….... 65

Appendix Figure 16: Garbage class specific percentage of garbage……………... 66

Appendix Figure 17: Country specific leading garbage codes…………………… 70

Appendix Figure 18: Country specific leading causes of death, pre and post garbage code redistribution………………………………………………. 87

Appendix Figure 1

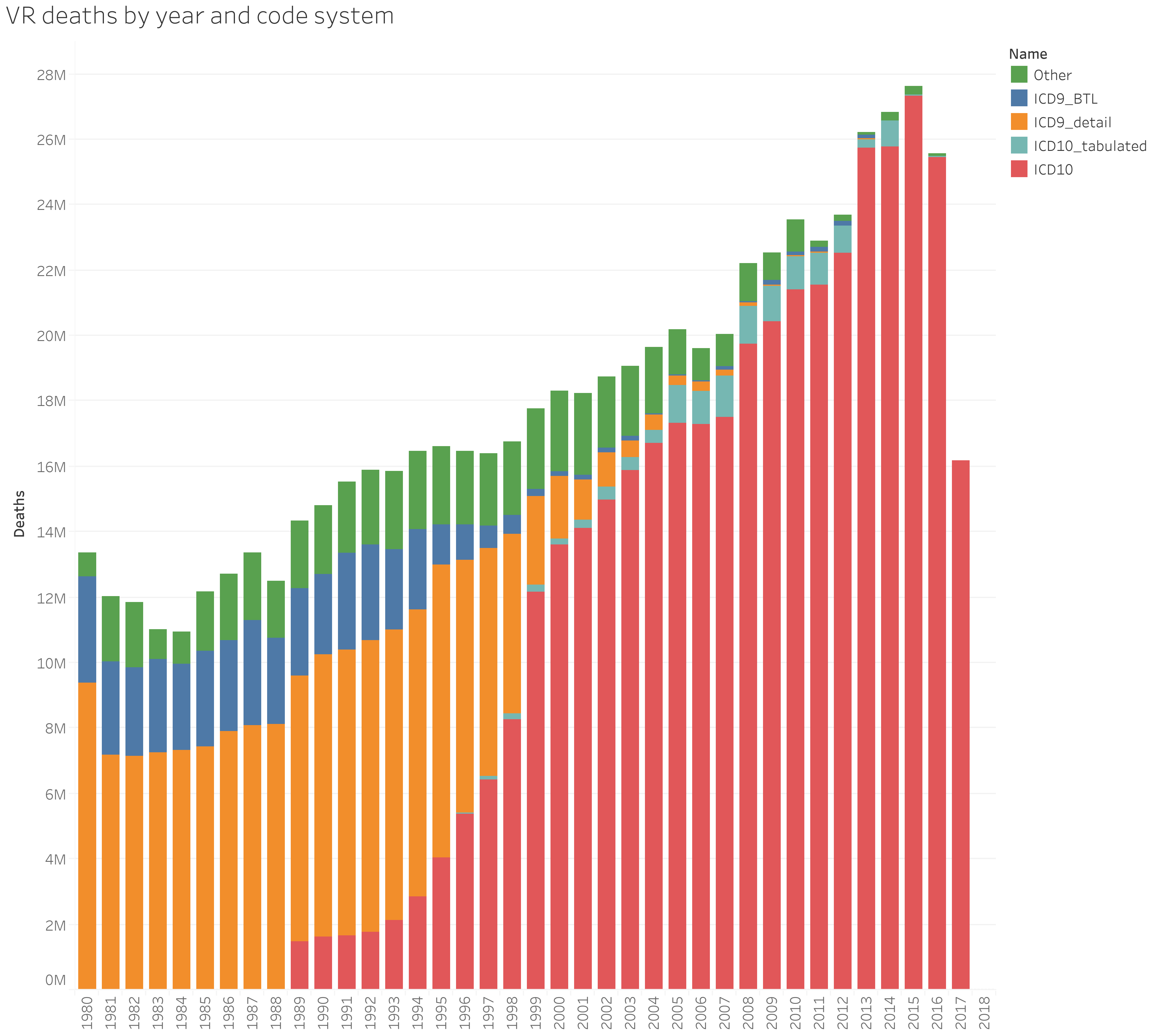

Appendix Figure 2

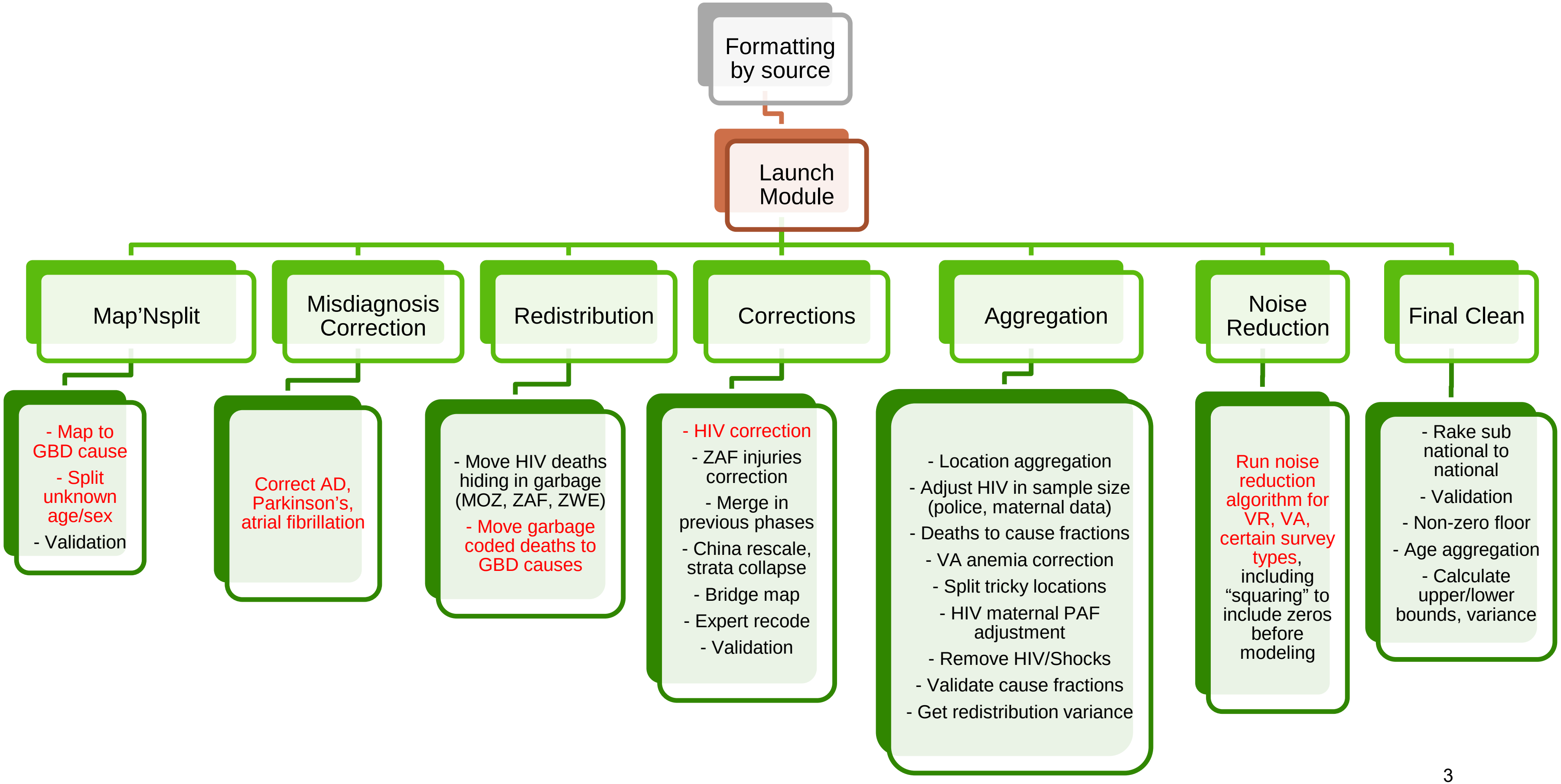

Appendix Figure 3

Map of ICD10 codes to GBD cause	
**GBD cause name**	**ICD codes**
Cholera	A00-A00.9
Garbage Code	A01, A14.9, A29-A31.9, A40-A45.9, A47-A48.0, A48.3, A48.8-A49.9, A59-A59.9, A61-A62, A64-A64.0, A71-A73, A74.0, A76, A97, A99-A99.0, B07-B09, B11-B14, B16.9-B17, B17.1, B17.8-B17.9, B19-B19.0, B19.2-B19.9, B28-B29, B30-B32.4, B34-B34.1, B34.3-B46.9, B49-B49.9, B54-B55, B55.1-B55.9, B58-B59.9, B61-B62, B64, B68-B68.9, B73-B74.2, B76-B76.9, B78-B82.9, B83.9-B85.4, B87-B89, B92-B94.0, B94.2-B94.9, B95.6-B97.1, B97.3, B97.7-B99.9, C14-C14.9, C22.9, C26-C29, C35-C36, C39-C39.9, C42, C46-C46.9, C55-C55.9, C57.9, C59-C6, C63.9, C68, C68.9-C69, C69.9, C74-C74.9, C75.9-C80.9, C83, C83.9, C85.1, C85.9, C87, C91.1, C91.4-C91.5, C91.7-C91.9, C92.7-C92.9, C93.2, C93.5-C93.7, C93.9, C94.6, C97-D00.0, D01, D01.4-D02, D02.4-D02.9, D07, D07.3, D07.6-D09, D09.1, D09.7, D09.9-D10, D10.9, D13, D13.9-D14, D14.4, D17-D21.9, D28, D28.9-D29, D29.9-D30, D30.9, D36.0, D36.9-D37.0, D37.6-D38, D38.6-D39.0, D39.7, D39.9-D40, D40.9-D41, D41.9, D44, D44.9, D48, D48.7-D49.1, D49.5, D49.7-D50.0, D50.9, D54, D59, D59.4, D59.8-D59.9, D62-D63.0, D63.8-D64, D64.1-D65.9, D68, D69.9, D75.9, D79-D85, D87-D88, D89.8-D99, E07.8-E08.9, E12-E16, E17-E19, E34.0, E34.9-E35.8, E37-E39, E47-E50.9, E62, E64.1, E69, E85.3-E87.9, E90-E998, F04-F07.0, F07.2-F09.9, F17-F17.9, F19-F23.9, F25-F50, F50.8-G00, G00.9-G02.8, G03.9, G06-G09.9, G15-G19, G21, G21.2, G21.4-G22.0, G27-G29, G32-G34, G38-G39., G42-G44.8, G47-G47.2, G47.4-G60.9, G62-G62.0, G62.2-G69, G74-G89.4, G91-G93.6, G93.8-G94.8, G96-G96.9, G98-H05, H05.2-H69.9, H71-H99, I00.0, I03-I04., I10-I10.9, I14-I19, I26-I27, I27.8-I27.9, I29-I29.9, I31.2-I31.4, I37.9, I42-I42.0, I42.9, I44-I46.9, I49-I51, I51.5-I59, I64-I64.9, I67, I67.4, I67.8-I68, I68.8-I69, I69.4-I70.1, I70.9, I74-I76, I90-I95.1, I95.8-I96.9, I98.4-I98.8, I99-ID5.9, J02.9, J03.9, J04.3, J06, J06.9-J08, J15.9, J17-J19.6, J22-J29, J40-J40.9, J47-J59, J64-J65.0, J69-J69.9, J71-J81.9, J83, J85-J90.9, J93-J94.9, J96-K19, K21-K21.9, K22.7, K30, K31.9-K34, K39, K47-K49, K53-K54, K63-K63.4, K63.8-K63.9, K65-K66.1, K66.9, K68.1-K69, K70.4-K71.6, K71.8-K72.9, K75.0, K78-K79, K84, K87-K89, K92-K92.2, K92.9-K93, K96-K99, L06-L07, L09, L15-L50.9, L52-L54.8, L56-L56.2, L56.4-L56.5, L57-L57.9, L59-L87.9, L90-L92.9, L94-L96, L98.5-L99.8, M04, M10-M12.0, M12.2-M29, M37-M39, M43.2-M49, M49.2-M64, M65.1-M71, M71.2-M72.4, M72.8-M73, M73.8-M79.9, M83-M86.2, M86.5-M86.9, M87.2-M87.9, M89.1-M89.4, M90-M99.9, N09, N13-N13.5, N13.7-N13.9, N17-N17.9, N19-N19.9, N24, N28.8-N28.9, N32.1-N32.2, N32.8-N33.8, N35-N35.9, N37-N38, N39.3-N40.9, N42-N43.4, N44.1-N44.8, N46-N48.9, N50-N59, N61-N64.9, N66-N71.9, N73-N74.0, N74.2-N74.8, N78-N79, N82-N82.9, N84, N84.2-N86, N88-N95.9, N97-N97.9, O08-O08.9, O17-O19, O27, O37-O39, O49-O59, O78-O79, O93-O95.9, P06, P16-P18, P23, P23.5-P23.9, P30-P34.2, P37.3-P37.4, P40-P49, P62-P69, P73, P79, P82, P85-P89, P96.9-P99.9, Q08-Q10.3, Q19, Q29-Q29., Q36.0-Q36.9, Q46-Q49, Q88, Q89.9, Q94, Q99.9-R19.6, R19.8-R50.1, R50.8-R78, R78.6-R94.8, R96-TO7., U05, U08-U81, U89.9-U99, V87-V87.1, V87.4-V88.1, V88.4-V89.9, V99-V99.0, W47-W48, W63, W71-W72, W76-W76.9, W82, W95-W97, W98, X07, X40-X44.9, X46-X46.9, X49-X49.9, X55-X56, X59-X59.9, X84-X84.9, Y09-Y14.9, Y16-Y34.9, Y85-Y87, Y87.2, Y89, Y89.9-Z15.8, Z17-ZZZ
Typhoid fever	A01.0
Paratyphoid fever	A01.1-A01.4
Other salmonella infections	A02-A02.0, A02.8-A02.9
Invasive Non-typhoidal Salmonella (iNTS)	A02.1-A02.2
Shigellosis	A03-A03.9
Other diarrheal diseases	A04, A04.6, A04.8-A04.9, A07, A07.3-A07.4, A08, A08.3-A09.9, K52.1-K52.3, R19.7
Enteropathogenic E coli infection	A04.0
Enterotoxigenic E coli infection	A04.1-A04.4
Campylobacter enteritis	A04.5
Clostridium difficile	A04.7
Other bacterial foodborne diarrhea	A05-A05.9
Amoebiasis	A06-A06.9
Other intestinal infectious diseases	A07.0-A07.1, A07.8-A07.9

Map of ICD10 codes to GBD cause

GBD cause name	ICD codes
Cryptosporidiosis	A07.2
Rotaviral enteritis	A08.0
Norovirus	A08.1
Adenovirus	A08.2
Respiratory tuberculosis	A10-A14, A15-A16.9
Tuberculosis of nervous system	A17-A17.9
Tuberculosis of other organs	A18, A18.4-A19.9, K67.3
Tuberculosis of bones and joints	A18.0
Tuberculosis of genitourinary system	A18.1
Tuberculous peripheral lymphadenopathy	A18.2
Tuberculosis of intestines, peritoneum and mesenteric glands	A18.3
Zoonotic bacterial diseases	A20-A28.9, M49.1
Other unspecified infectious diseases	A32-A32.9, A38-A38.9, A48.2, A48.4-A48.5, A65-A65.0, A69-A69.1, A74, A74.8-A74.9, A81-A81.9, A88-A89.9, B03-B04, B25-B25.9, B27-B27.9, B29.4, B33, B33.3-B33.8, B47-B48.8, B91, B95-B95.5, D70.3, D89.3, F02.1, G14-G14.6, I00, I02, I02.9, I98.1, K67.8, K75.3, K76.3, K77.0, M89.6, P35, P35.1-P35.2, P35.9, P37, P37.2, P37.5-P37.9
Tetanus	A33-A35.0
Diphtheria	A36-A36.9
Pertussis	A37-A37.9
Meningococcal meningitis	A39-A39.9
Pyoderma	A46-A46.0, A66-A67.9, H05.1, I89.1-I89.8, L00-L02.9, L04-L05.9, L08-L08.9, L88, L97-L98.4
Other lower respiratory infections	A48.1, A70, B34.2, B97.2, J12-J12.0, J12.2-J12.9, J15-J15.2, J15.5, J15.7-J15.8, J16-J16.9, J20-J21.9, J91.0, P23.0-P23.4
Syphilis	A50-A53.9, I98.0, K67.2, M03.1, M73.1

Map of ICD10 codes to GBD cause

GBD cause name	ICD codes
Gonococcal infection	A54-A54.9, K67.1, M73.0
Chlamydial infection	A55-A56.8, K67.0
Other sexually transmitted infections	A57-A58, A63-A63.8, B63
Genital herpes	A60-A60.9
Other neglected tropical diseases	A68-A68.9, A69.2-A69.9, A75-A75.9, A77-A79.9, A92-A94.0, A96-A96.9, A98-A98.3, A98.5-A98.8, B33.0-B33.1, B60-B60.8, B67.5-B67.7, B70-B71.9, B74.3-B75, B83-B83.8, P37.1
Poliomyelitis	A80-A80.9
Rabies	A82-A82.9
Encephalitis	A83-A86.4, B94.1, F07.1, G04-G05.8, G21.3
Viral meningitis	A87-A87.9
Dengue	A90-A91.9
Yellow fever	A95-A95.9
Ebola	A98.4
Non-genital herpes infection	B00-B00.9, B10-B10.8
Varicella and herpes zoster	B01-B02.9, P35.8
Measles	B05-B05.9
Rubella	B06-B06.9, P35.0
Acute hepatitis A	B15-B15.9
Acute hepatitis B	B16-B16.2, B17.0, B19.1, P35.3
Acute hepatitis E	B17.2
Cirrhosis and other chronic liver diseases	B18-B18.9, I85-I85.9, I98.2, K70-K70.3, K71.7, K73-K75, K75.2, K75.4-K76.2, K76.4-K76.9, K77.8

Map of ICD10 codes to GBD cause

GBD cause name	ICD codes
HIV/AIDS resulting in other diseases	B20, B20.1-B24.9, F02.4
HIV/AIDS - Drug-susceptible Tuberculosis	B20.0
Mumps	B26-B26.9
Myocarditis	B33.2, I40-I41.9, I51.4
Malaria	B50-B53.8
Visceral leishmaniasis	B55.0
African trypanosomiasis	B56-B56.9
Chagas disease	B57-B57.5, K93.1
Schistosomiasis	B65-B65.9
Food-borne trematodiases	B66-B66.9, B72.0
Cystic echinococcosis	B67-B67.4, B67.8-B67.9
Cysticercosis	B69-B69.9
Guinea worm disease	B72
Ascariasis	B77-B77.9
Scabies	B86
Drug-susceptible tuberculosis	B90-B90.9, K93.0, M49.0, N74.1, P37.0
Respiratory syncytial virus pneumonia	B97.4-B97.6, J12.1
Lip and oral cavity cancer	C0-C08.9, D10.0-D10.5, D11-D11.9
Other pharynx cancer	C09-C10.9, C12-C13.9, D10.7
Nasopharynx cancer	C11-C11.9, D10.6

Map of ICD10 codes to GBD cause	
**GBD cause name**	**ICD codes**
Esophageal cancer	C15-C15.9, D00.1, D13.0
Stomach cancer	C16-C16.9, D00.2, D13.1, D37.1
Other malignant neoplasms (internal)	C17-C17.9, C3-C31.9, C37-C38.8, C4, C48-C48.9, C4A-C5, C51-C52.9, C57-C57.8, C60-C60.9, C63-C63.8, C66-C66.9, C68.0-C68.8, C7, C75-C75.0, C75.4-C75.8, D07.4, D09.2, D13.2-D13.3, D14.0, D15-D16.9, D28.0-D28.1, D28.7, D29.0, D30.2, D30.4-D30.8, D31-D31.9, D35-D35.2, D35.5-D36, D36.1-D36.7, D37.2, D38.2-D38.5, D39.2, D39.8, D41.2-D41.3, D44.1-D44.8, D48.0-D48.4
Colon and rectum cancer	C18-C21.9, D01.0-D01.3, D12-D12.9, D37.3-D37.5
Liver cancer	C22-C22.1, C22.3-C22.8, D13.4
Hepatoblastoma	C22.2
Gallbladder and biliary tract cancer	C23-C24.9, D13.5
Pancreatic cancer	C25-C25.9, D13.6-D13.7
Larynx cancer	C32-C32.9, D02.0, D14.1, D38.0
Tracheal, bronchus, and lung cancer	C33-C34.9, D02.1-D02.3, D14.2-D14.3, D38.1
Malignant neoplasm of bone and articular cartilage	C40-C41.9
Malignant skin melanoma	C43-C43.9, D03-D03.9, D22-D23.9, D48.5
Non-melanoma skin cancer (squamous-cell carcinoma)	C44-C44.9, D04-D04.9, D49.2
Mesothelioma	C45-C45.9
Neuroblastoma and other peripheral nervous cell tumors	C47-C47.9
Soft tissue and other extraosseous sarcomas	C49-C49.9
Breast cancer	C50-C50.9, D05-D05.9, D24-D24.9, D48.6, D49.3
Cervical cancer	C53-C53.9, D06-D06.9, D26.0
Uterine cancer	C54-C54.9, D07.0-D07.2, D26.1-D26.9
Ovarian cancer	C56-C56.9, D27-D27.9, D39.1

Map of ICD10 codes to GBD cause

GBD cause name	ICD codes
Other direct maternal disorders	C58-C58.0, N98-N98.9, O09-O09.9, O21-O22.9, O26-O26.9, O28-O31.8, O34-O36.9, O40-O43.1, O43.8-O43.9, O47-O48.1, O60-O61.9, O63-O63.9, O68-O69.9, O70.0-O70.9, O73-O77.9, O80-O84.9, O87-O90.9, O92-O92.7
Prostate cancer	C61-C61.9, D07.5, D29.1, D40.0
Testicular cancer	C62-C62.9, D29.2-D29.8, D40.1-D40.8
Kidney cancer	C64-C65.9, D30.0-D30.1, D41.0-D41.1
Bladder cancer	C67-C67.9, D09.0, D30.3, D41.4-D41.8, D49.4
Other eye cancers	C69.0-C69.1, C69.3-C69.8
Retinoblastoma	C69.2
Brain and central nervous system cancer	C70-C72.9, C75.1-C75.3
Thyroid cancer	C73-C73.9, D09.3, D09.8, D34-D34.9, D44.0
Hodgkin lymphoma	C81-C81.9
Other non-Hodgkin lymphoma	C82-C82.9, C83.0-C83.6, C83.8, C84-C85.0, C85.2-C85.8, C86-C86.6, C96-C96.9
Burkitt lymphoma	C83.7
Multiple myeloma	C88-C90.9
Leukemia	C91, C92, C93, C94, C95-C95.9
Acute lymphoid leukemia	C91.0, C91.2-C91.3, C91.6
Acute myeloid leukemia	C92.0, C92.3-C92.6, C93.0, C94.0, C94.2, C94.4-C94.5
Chronic myeloid leukemia	C92.1-C92.2
Other leukemia	C93.1, C93.3, C93.8, C94.1, C94.3, C94.7-C94.8
Uterine fibroids	D25-D26, D28.2
Benign and in situ CNS neoplasms	D32-D33.9, D35.3-D35.4, D42-D43.9, D49.6

Map of ICD10 codes to GBD cause	
**GBD cause name**	**ICD codes**
Myelodysplastic, myeloproliferative, and other hematopoietic neoplasms	D45-D47.9
Dietary iron deficiency	D50.1-D50.8
Other nutritional deficiencies	D51-D52.0, D52.8-D53.9, E00-E02, E51-E61.9, E63-E64, E64.2-E64.9, M12.1
Post procedural or drug treatment disorders	D52.1, D59.0, D59.2, D59.6, D69.5, D70.1-D70.2, D78-D78.8, E03.2, E06.4, E09-E09.9, E16.0, E23.1, E24.2, E27.3, E36-E36.8, E66.1, E88.3, E89-E89.9, G21.0-G21.1, G24.0, G25.1, G25.4, G25.6-G25.7, G72.0, G93.7, G97-G97.9, I95.2-I95.3, I97-I97.9, I98.9, J70.0-J70.5, J95-J95.9, K43-K43.9, K52.0, K62.7, K91-K91.9, K94-K95.8, M87.1, N14-N14.4, N65-N65.1, N99-N99.9, P96.2, P96.5, R50.2
G6PD deficiency	D55-D55.2
Other hemoglobinopathies and hemolytic anemias	D55.3-D55.9, D58-D58.9, D59.1, D59.3, D59.5, D60-D60.9, D64.0
Thalassemias	D56-D56.9
Sickle cell disorders	D57-D57.8
Aplastic anemias	D61-D61.9
Chronic kidney disease	D63.1, N18-N18.9
Other endocrine, metabolic, blood, and immune disorders	D66-D67, D68.0-D69.4, D69.6-D69.8, D70-D70.0, D70.4-D75.8, D76-D77, D86.8, D89-D89.2, E07.0, E16.1-E16.9, E20-E23.0, E23.2-E24.1, E24.3, E24.8-E27.2, E27.4-E28.1, E28.3-E34, E34.1-E34.8, E67-E68, E70-E77.9, E79-E83.9, E85-E85.2, E88-E88.2, E88.4-E88.9
Interstitial lung disease and pulmonary sarcoidosis	D86-D86.2, D86.9, J84-J84.9
Other skin and subcutaneous diseases	D86.3, L10-L14.0, L51-L51.9
Hypothyroidism	E03-E03.1, E03.3-E04.9, E06-E06.3, E06.5-E07, E07.1
Hyperthyroidism	E05-E05.9
Diabetes mellitus type 1	E10-E10.1, E10.3-E10.9, P70.2
Chronic kidney disease due to diabetes mellitus type 1	E10.2
Diabetes mellitus type 2	E11-E11.1, E11.3-E11.9
Chronic kidney disease due to diabetes mellitus type 2	E11.2
Alcohol use disorders	E24.4, F10-F10.9, G31.2, G62.1, G72.1, P04.3, Q86.0, R78.0, X45-X45.9, X65-X65.9, Y15-Y15.9

Map of ICD10 codes to GBD cause

GBD cause name	ICD codes
Polycystic ovarian syndrome	E28.2
Protein-energy malnutrition	E40-E46.9, E64.0
Obesity	E65-E66.0, E66.2-E66.9
Lipoprotein metabolism and other lipidaemias disorders	E78-E78.9
Cystic fibrosis	E84-E84.9
Alzheimer's disease and other dementias	F00-F02.0, F02.8-F03.9, G30-G31.1, G31.8-G31.9
Other neurological disorders	F02.2, G10-G12.1, G13-G13.8, G23-G24, G24.1-G25.0, G25.2-G25.3, G25.5, G25.8-G26.0, G36-G37.9, G61-G61.9, G70-G71.1, G71.3-G71.9, G73-G73.7, G90-G90.9, G95-G95.9
Parkinson's disease	F02.3, G20-G20.9
Opioid use disorders	F11-F11.9, P96.1, R78.1
Cannabis use disorders	F12-F12.9
Other drug use disorders	F13-F13.9, F16-F16.9, F18-F18.9, P04.4, R78.3-R78.5
Cocaine use disorders	F14-F14.9, R78.2
Amphetamine use disorders	F15-F15.9
Other mental disorders	F24
Anorexia nervosa	F50.0-F50.5
H influenzae type B meningitis	G00.0
Pneumococcal meningitis	G00.1
Other meningitis	G00.2-G00.8, G03-G03.8
Motor neuron disease	G12.2-G12.9
Multiple sclerosis	G35-G35.9

Map of ICD10 codes to GBD cause

GBD cause name	ICD codes
Idiopathic epilepsy	G40-G41.9
Ischemic stroke	G45-G46.8, I63-I63.9, I65-I66.9, I67.2-I67.3, I67.5-I67.6, I69.3
Sleep apnea	G47.3
Other congenital birth defects	G71.2, Q02-Q04.9, Q06-Q07.9, Q10.4-Q18.9, Q30-Q34.9, Q80-Q86, Q86.1-Q86.8, Q89-Q89.8
Fibromyalgia and other neuropathies	G72, G72.2-G72.9, M33-M33.9
Cellulitis	H05.0, L03-L03.9, M72.5-M72.6
Otitis media	H70-H70.9
Rheumatic heart disease	I01-I01.9, I02.0, I05-I09.9
Hypertensive heart disease	I11-I11.9
Chronic kidney disease due to hypertension	I12-I13.9
Angina	I20-I20.9
Acute myocardial infarction	I21-I24.9
Chronic ischemic heart disease	I25-I25.9
Pulmonary Arterial Hypertension	I27.0
Other musculoskeletal disorders	I27.1, I67.7, M07-M08, M30-M31.9, M34-M36.8, M40-M43.1, M87-M87.0, M88-M89.0, M89.5, M89.7-M89.9
Other cardiovascular and circulatory diseases (internal)	I27.2, I28-I28.9, I30-I31.1, I31.8-I32.8, I47-I47.9, I51.0-I51.3, I68.0, I72-I72.9, I77-I79.8, I86-I86.0, I86.4-I87, I87.1-I89.0, I89.9, I98
Endocarditis	I33-I33.9, I38-I39.9
Non-rheumatic degenerative mitral valve disease	I34-I34.9
Non-rheumatic calcific aortic valve disease	I35-I35.9
Other non-rheumatic valve diseases	I36-I37.8

Map of ICD10 codes to GBD cause

GBD cause name	ICD codes
Other cardiomyopathy	I42.1-I42.5, I42.7-I42.8, I43-I43.9
Alcoholic cardiomyopathy	I42.6
Atrial fibrillation and flutter	I48-I48.9
Subarachnoid hemorrhage	I60-I60.9, I62.0, I67.0-I67.1, I69.0
Intracerebral hemorrhage	I61-I62, I62.1-I62.9, I68.1-I68.2, I69.1-I69.2
Lower extremity peripheral arterial disease	I70.2-I70.8, I73-I73.9
Aortic aneurysm	I71-I71.9
Phlebitis and thrombophlebitis	I80-I82.9, I87.0, K75.1
Varicose veins	I83-I83.9, I86.1-I86.3
Hemorrhoids, anal fissure, anal abscess, and fistula	I84-I84.9, K60-K62, K62.2-K62.6, K62.8-K62.9, K64-K64.9
Upper respiratory infections	J00-J02.8, J03-J03.8, J04-J04.2, J05-J05.1, J06.0-J06.8, J36-J36.0
Influenza	J09-J11.8, U04-U04.9
Pneumococcal pneumonia	J13-J13.9, J15.3-J15.4, J15.6
H influenzae type B pneumonia	J14-J14.0
Allergic disorders of upper respiratory system	J30-J30.9
Chronic diseases of upper respiratory system	J31-J34.0, J35-J35.9, J37-J37.1, J39.0-J39.1
Other chronic respiratory diseases	J34.1-J34.9, J38-J39, J39.2-J39.9, J66-J68.9, J70, J70.8-J70.9, J82, J91, J91.8-J92, J92.9
Chronic obstructive pulmonary disease	J41-J44.9
Asthma	J45-J46.9
Coal workers pneumoconiosis	J60-J60.0

Map of ICD10 codes to GBD cause

GBD cause name	ICD codes
Asbestosis	J61-J61.0, J92.0
Silicosis	J62-J62.9
Other pneumoconiosis	J63-J63.8
Esophageal diseases	K20-K20.9, K22-K22.6, K22.8-K24
Peptic ulcer disease	K25-K28.9
Gastritis and duodenitis	K29-K29.9
Other digestive diseases	K31-K31.8, K38-K38.2, K58-K58.0, K59, K59.1-K59.2, K59.4-K59.9, K66.8, K67, K68, K77, K90, K90.1-K90.9, K92.8, K93.8
Appendicitis	K35-K37.9, K38.3-K38.9
Inguinal hernia	K40-K40.9
Femoral hernia	K41-K41.9
Abdominal and other hernia	K42-K42.9, K45-K46.9
Diaphragmatic hernia	K44-K44.9
Crohn's disease	K50-K50.9, M09.1
Ulcerative colitis	K51-K52, K52.8-K52.9
Vascular intestinal disorders	K55-K55.9
Paralytic ileus and intestinal obstruction	K56-K56.9
Diverticular disease of intestines	K57-K57.9
Constipation	K58.9, K59.0, K59.3
Benign and in situ intestinal neoplasms	K62.0-K62.1, K63.5
Gallbladder and biliary diseases	K80-K83.9

Map of ICD10 codes to GBD cause

GBD cause name	ICD codes
Pancreatitis	K85-K86.9
Celiac disease	K90.0
Environmental heat and cold exposure	L55-L55.9, L56.3, L56.8-L56.9, L58-L58.9, W88-W94.9, W97.9, W99-W99.9, X30-X32.9, X39-X39.9
Decubitus ulcer	L89-L89.9
Systemic and discoid lupus erythematous	L93-L93.2, M32-M32.9
Pyogenic and reactive arthritis	M00-M03.0, M03.2-M03.6, M08.9-M09.0, M09.2-M09.8, M65-M65.0, M71.0-M71.1
Rheumatoid arthritis	M05-M06.9, M08.0-M08.8
Osteoporosis and related pathological fracture	M80-M82.8
Osteomyelitis non-traumatic	M86.3-M86.4
Acute glomerulonephritis	N00-N01.9
Chronic kidney disease due to other and unspecified causes	N02-N02.9, N07-N08.8, N15.0, Q61-Q62.8
Chronic kidney disease due to glomerulonephritis	N03-N06.9
Urinary tract infections and interstitial nephritis	N10-N12.9, N13.6, N15, N15.1-N16.8, N30-N30.3, N30.8-N30.9, N34-N34.3, N39.0-N39.2
Urolithiasis	N20-N23.0
Other urinary diseases	N25-N28.1, N29-N29.8, N31-N32.0, N32.3-N32.4, N36-N36.9, N39, N44-N44.0
Adverse effects of medical treatment	N30.4, P93-P93.8, Y40-Y84.9, Y88-Y88.3
Prostatitis and epididymitis	N41-N41.9, N45-N45.9, N49-N49.9
Other benign and in situ neoplasms	N60-N60.9
Inflammatory diseases of cervix, vagina, and vulva	N72-N72.0, N75-N77.8
Endometriosis	N80-N80.9

Map of ICD10 codes to GBD cause

GBD cause name	ICD codes
Genital prolapse	N81-N81.9
Other gynecological diseases	N83-N83.9
Benign and in situ cervical and uterine neoplasms	N84.0-N84.1, N87-N87.9
Spontaneous abortion	N96, O01-O03.9
Ectopic pregnancy	O00-O00.9
Induced abortion	O04-O07.9
Maternal hypertensive disorders	O10-O16.9
Maternal hemorrhage	O20-O20.9, O43.2, O44-O46.9, O62-O62.9, O67-O67.9, O70, O72-O72.3
Maternal sepsis and other maternal infections	O23-O23.9, O85-O86.8, O91-O91.2
Indirect maternal deaths	O24-O25.3, O98-O98.6, O98.8-O99.9
Maternal obstructed labor and uterine rupture	O32-O33.9, O64-O66.9, O71-O71.9
Late maternal deaths	O96-O97.9
CC code	O98.7
Other neonatal disorders	P00-P01, P01.2-P01.6, P01.8-P01.9, P04-P04.2, P04.5-P04.9, P08-P09, P19-P19.9, P29-P29.9, P50-P51.9, P53-P54.9, P60-P61.1, P61.3-P61.9, P70-P70.1, P70.3-P72.9, P74-P76.9, P78, P80-P81.9, P83-P84, P92-P92.9, P94-P94.9, P96, P96.3-P96.4, P96.8
Neonatal preterm birth	P01.0-P01.1, P05-P05.9, P07-P07.3, P22-P22.9, P25-P28.9, P52-P52.9, P61.2, P77-P77.9, P78.0-P78.9
Neonatal encephalopathy due to birth asphyxia and trauma	P01.7, P02-P03.9, P10-P15.9, P20-P21.9, P24-P24.9, P90-P91.9
Neonatal sepsis and other neonatal infections	P36-P36.9, P38-P39.9
Hemolytic disease and other neonatal jaundice	P55-P59.9
Still Born	P95-P95.9
Urogenital congenital anomalies	P96.0, Q50-Q60.6, Q63-Q64.9

Map of ICD10 codes to GBD cause

GBD cause name	ICD codes
Neural tube defects	Q00-Q01.9, Q05-Q05.9
Congenital heart anomalies	Q20-Q28.9
Orofacial clefts	Q35-Q36, Q37-Q37.9
Digestive congenital anomalies	Q38-Q45.9, Q79.0-Q79.5
Congenital musculoskeletal and limb anomalies	Q65-Q79, Q79.6-Q79.9
Other chromosomal abnormalities	Q87-Q87.8, Q91-Q93.9, Q95-Q95.9, Q97-Q97.9, Q99-Q99.8
Down syndrome	Q90-Q90.9
Turner syndrome	Q96-Q96.9
Klinefelter syndrome	Q98-Q98.9
Sudden infant death syndrome	R95-R95.9
Conflict and terrorism	U00-U03, Y38-Y38.9
Zika virus	U06-U06.9
COVID-19	U07-U07.2
Other drug-resistant infectious diseases	U82-U84, U85-U89, Z16-Z16.3
Other transport injuries	V00-V00.8, V05-V05.9, V81-V81.9, V83-V86.9, V88.2-V88.3, V90-V98.8
Pedestrian road injuries	V01-V04.9, V06-V09.9
Cyclist road injuries	V10-V19.9
Motorcyclist road injuries	V20-V29.9
Motor vehicle road injuries	V30-V79.9, V87.2-V87.3
Other road injuries	V80-V80.9, V82-V82.9

Map of ICD10 codes to GBD cause

GBD cause name	ICD codes
Falls	W00-W19.9
Other exposure to mechanical forces	W20-W31.9, W35-W38.9, W40-W43.9, W45.0-W45.2, W46-W46.2, W49-W52
Unintentional firearm injuries	W32-W34.9
Other unintentional injuries	W39-W39.9, W77-W77.9, W81-W81.9, X50-X54.9, X57-X58.9
Foreign body in other body part	W44-W45, W45.3-W45.9
Non-venomous animal contact	W52.0-W62.9, W64-W64.9
Drowning	W65-W70.9, W73-W74.9
Pulmonary aspiration and foreign body in airway	W75-W75.9, W78-W80.9, W83-W84.9
Electrocution	W85-W87.9
Fire, heat, and hot substances	X00-X06.9, X08-X19.9
Contact with snakes and lizards	X20-X20.9
Contact with spiders	X21-X21.9
Contact with scorpions	X22-X22.9
Contact with hornets, wasps and bees	X23-X23.9
Contact with other venomous	X24-X29.9
Victim of lightning	X33-X33.9
Earthquake	X34-X34.9
Volcanic eruption	X35-X35.9
Avalanche, landslide and other earth movements	X36-X36.9
Cataclysmic storm	X37-X37.9

Map of ICD10 codes to GBD cause

GBD cause name	ICD codes
Flood	X38-X38.9
Poisoning by carbon monoxide	X47-X47.9
Poisoning by pesticides	X48-X48.9
Self-harm by other specified means	X60-X64.9, X66-X67.9, X69-X71.9, X75-X75.9, X77-X83.9, Y87.0
Self-harm by poisoning pesticides	X68-X68.9
Self-harm by firearm	X72-X74.9
Self-harm by fire, heat, and hot substances	X76-X76.9
Physical violence by other means	X85-X92.9, X96-X98.9, Y00-Y04.9, Y06-Y08.9, Y87.1
Physical violence by firearm	X93-X95.9
Physical violence by sharp object	X99-X99.9
Sexual violence	Y05-Y05.9
Police conflict and executions	Y35-Y35.9, Y89.0
Military operations	Y36-Y37.9, Y89.1
Multidrug-resistant tuberculosis without extensive drug resistance	U84.3

Map of ICD9 codes to GBD cause

GBD cause name	ICD codes
Garbage Code	000-000.9, 002, 030-031.9, 038-039.9, 040.0, 041.1-041.9, 067-070, 070.3-070.9, 076-078.2, 078.8-078.9, 079.8-079.9, 085, 085.1-085.9, 088.0-088.7, 089-089.9, 105-119, 125-125.3, 126-126.9, 127.2-127.9, 130-132.9, 133.8-134.9, 136.3-136.9, 139.1-139.9, 149-149.9, 155.2, 159-159.9, 165-169, 176-179.9, 183.9-184, 184.5, 184.9, 187, 187.9, 189, 189.9, 190.9, 194-194.0, 194.9-199.9, 202.9, 204.1, 204.5-204.9, 205.8-205.9, 206.2-206.9, 209, 209.2-209.3, 209.6-210, 211, 211.9-212, 212.9, 214-216.9, 221, 221.9-222, 222.9-223, 223.9, 229, 229.1, 229.9-230.0, 230.9-231, 231.8-231.9, 233, 233.3, 233.6, 233.9-234, 234.9-235, 235.1-235.3, 235.5, 235.9-236, 236.3, 236.6, 236.9, 237.4, 238, 239-239.1, 239.5, 239.7-239.9, 244, 244.9, 247-250.9, 259.2, 264-264.9, 274-274.9, 276.0-276.9, 277.3, 278, 279-281, 285-285.9, 286.6, 289.1-289.3, 289.8-289.9, 293-294.0, 295-302.9, 304, 304.9-305, 305.1, 305.9-307.0, 307.2-320, 320.9, 324-327.1, 328-329, 331.3-331.4, 332.1-332.9, 338-339.8, 342-344.9, 346-348.9, 349.9-353.6, 354-355.9, 357, 357.2, 357.8-357.9, 360-376, 376.2-380.9, 384-389.9, 399-401.9, 405-409.4, 415-416, 416.2-416.9, 418-419.9, 423.0, 424, 424.4-424.5, 424.9-425, 425.9-427, 427.4-427.5, 427.9-429, 429.1-429.9, 436-437, 437.3, 437.9-440.1, 440.3, 440.8-440.9, 444-445.8, 458-458.9, 459.0, 459.5-459.9, 464.5, 465, 465.9, 482.9-483, 484, 484.8-486.9, 490-490.9, 494-494.9, 505-505.9, 507-507.9, 510-514.9, 515.0-515.9, 518-518.5, 518.8, 519, 519.8-529.9, 530.1, 530.7-530.9, 536.2-536.3, 536.8-536.9, 537.7, 537.9, 544-549, 559-559.0, 560.4-560.7, 561, 562.2-563, 564.8-564.9, 567-569, 569.8-570.9, 572-572.1, 573.1-573.3, 578-578.9, 584-584.9, 586-587.9, 591-591.9, 593.9, 599.7, 599.9-600.9, 603-603.9, 605-608.1, 608.3-609, 611-616.9, 619-619.9, 621-621.3, 622-622.0, 622.8-628.9, 629.9, 637-637.9, 639-639.9, 690-693.9, 695.8-706.9, 708-709.9, 712-713.8, 714.4, 715-716, 716.1-730.0, 730.2-730.3, 730.7-731.9, 733, 733.2-739.9, 749.1, 759, 759.9, 770.0, 779.9-788, 788.1-790.2, 790.4-797.9, 798.1-E80, E800.8-E800.9, E801.8-E801.9, E802.8-E802.9, E803.8-E803.9, E804.8-E804.9, E805.8-E805.9, E806.8-E806.9, E807.8-E810, E810.8-E811, E811.8-E812, E812.8-E813, E813.8-E814, E814.8-E815, E815.8-E816, E816.8-E817, E817.8-E818, E818.8-E819, E819.8-E820, E820.8-E821, E821.8-E822, E822.8-E823, E823.8-E824, E824.8-E825, E825.8-E826, E826.8-E827, E827.8-E828, E828.8-E829, E829.8-E83, E839, E85, E850.3-E855.9, E858-E859, E866-E866.9, E87, E877, E88, E887-E887.0, E928.9-E929.0, E929.8-E929.9, E980-E989, ZZZ
Cholera	001-001.9
Typhoid fever	002.0
Paratyphoid fever	002.1-002.9
Invasive Non-typhoidal Salmonella (iNTS)	003-003.7
Other salmonella infections	003.8-003.9
Shigellosis	004-004.9
Other bacterial foodborne diarrhea	005-005.9
Amoebiasis	006-006.9
Other intestinal infectious diseases	007-007.3, 007.9-008.1
Cryptosporidiosis	007.4-007.7
Other diarrheal diseases	007.8, 008.3-009.9
Aeromonas	008.2
Respiratory tuberculosis	010-012.9
Tuberculosis of nervous system	013-013.9, 320.4
Tuberculosis of intestines, peritoneum and mesenteric glands	014-014.9
Tuberculosis of bones and joints	015-015.9

Map of ICD9 codes to GBD cause

GBD cause name	ICD codes
Tuberculosis of genitourinary system	016-016.9
Tuberculosis of other organs	017-017.1, 017.3-019.9
Tuberculous peripheral lymphadenopathy	017.2
Zoonotic bacterial diseases	020-029, 073-073.9, 078.3, 100-100.9, 484.4-484.5
Diphtheria	032-032.9
Pertussis	033-033.9, 484.3
Other unspecified infectious diseases	034, 034.1-034.9, 040, 040.1-041.0, 046-046.9, 050-051.9, 057-059.9, 074-075.9, 078.4-078.7, 079-079.5, 079.7, 101-101.6, 104-104.9, 136-136.2, 139, 323.0-323.3, 390-390.9, 392, 392.9, 771.0-771.2
Upper respiratory infections	034.0, 460-464.4, 464.8-464.9, 465.0-465.8, 475-475.9, 476.9
Pyoderma	035-035.9, 102-103.9, 376.0-376.1, 457.2-457.3, 680-680.9, 683-689
Meningococcal meningitis	036-036.9
Tetanus	037-037.9, 771.3
HIV/AIDS resulting in other diseases	042-044.9
Poliomyelitis	045-045.9, 138
Viral meningitis	047-049.9
Varicella and herpes zoster	052-053.9
Non-genital herpes infection	054-054.0, 054.2-054.9
Genital herpes	054.1
Measles	055-055.9, 484.0
Rubella	056-056.9
Yellow fever	060-060.9

Map of ICD9 codes to GBD cause

GBD cause name	ICD codes
Dengue	061-061.8
Encephalitis	062-064.9, 139.0, 323, 323.4-323.9
Other neglected tropical diseases	065-066.9, 080-083.9, 087-088, 088.8-088.9, 122.5-122.7, 123-123.0, 123.2-124.9, 125.4-125.6, 125.9, 127, 127.1, 128-129.0
Acute hepatitis A	070.0-070.1
Acute hepatitis B	070.2
Rabies	071-071.9
Mumps	072-072.9
Respiratory syncytial virus pneumonia	079.6, 480.1
Malaria	084-084.9
Visceral leishmaniasis	085.0
Chagas disease	086-086.2, 086.9, 425.6
African trypanosomiasis	086.3-086.5
Syphilis	090-097.9
Gonococcal infection	098-098.9
Other sexually transmitted infections	099-099.9
Schistosomiasis	120-120.9
Food-borne trematodiases	121-121.9
Cystic echinococcosis	122-122.4, 122.8-122.9
Cysticercosis	123.1
Guinea worm disease	125.7

Map of ICD9 codes to GBD cause

GBD cause name	ICD codes
Ascariasis	127.0
Scabies	133-133.6
Interstitial lung disease and pulmonary sarcoidosis	135-135.9, 515, 516-516.9
Drug-susceptible tuberculosis	137-137.9, 138.0-138.9, 730.4-730.6
Lip and oral cavity cancer	140-145.9, 210.0-210.6, 235.0
Other pharynx cancer	146-146.9, 148-148.9
Nasopharynx cancer	147-147.9, 210.7-210.9
Esophageal cancer	150-150.9, 211.0, 230.1
Stomach cancer	151-151.9, 211.1, 230.2
Other malignant neoplasms (internal)	152-152.9, 158-158.9, 160-160.9, 163-164.9, 183.2-183.8, 184.0-184.4, 184.8, 187.1-187.8, 189.2-189.4, 189.8, 194.1, 194.5-194.8, 209.0, 209.4, 211.2, 211.8, 212.0, 212.4-212.8, 213-213.9, 221.0-221.8, 222.1, 222.8, 223.2, 223.8, 224-224.9, 227-228.9, 229.0, 229.8, 230.7-230.8, 233.4-233.5, 234.0-234.8, 235.4, 235.8, 236.1, 238.0-238.1, 239.2
Colon and rectum cancer	153-154.9, 209.1, 209.5, 211.3-211.4, 230.3-230.6, 569.0
Liver cancer	155-155.1, 155.3-155.9, 211.5
Gallbladder and biliary tract cancer	156-156.9
Pancreatic cancer	157-157.9, 211.6-211.7
Larynx cancer	161-161.9, 212.1, 231.0, 235.6
Tracheal, bronchus, and lung cancer	162-162.9, 212.2-212.3, 231.1-231.2, 235.7
Malignant neoplasm of bone and articular cartilage	170-170.9
Soft tissue and other extraosseous sarcomas	171-171.9
Malignant skin melanoma	172-172.9
Non-melanoma skin cancer (squamous-cell carcinoma)	173-173.9, 222.4, 232-232.9, 238.2

Map of ICD9 codes to GBD cause	
**GBD cause name**	**ICD codes**
Breast cancer	174-175.9, 217-217.8, 233.0, 238.3, 239.3, 610-610.9
Cervical cancer	180-180.9, 219.0, 233.1, 622.1-622.2, 622.7
Other direct maternal disorders	181-181.9, 643-645.2, 650-651.9, 654-659.2, 659.4-659.9, 662-664.9, 665.4-665.9, 667-669.9, 671-679.1
Uterine cancer	182-182.9, 233.2
Ovarian cancer	183-183.0, 220-220.9, 236.2
Prostate cancer	185-185.9, 222.2, 236.5
Testicular cancer	186-186.9, 222.0, 222.3, 236.4
Bladder cancer	188-188.9, 223.3, 233.7, 236.7, 239.4
Kidney cancer	189.0-189.1, 189.5-189.6, 223.0-223.1
Other eye cancers	190-190.4, 190.6-190.8
Retinoblastoma	190.5
Brain and central nervous system cancer	191-192.9, 194.3-194.4
Thyroid cancer	193-193.9, 226-226.9
Other non-Hodgkin lymphoma	200-200.1, 200.3-200.9, 202-202.8
Burkitt lymphoma	200.2
Hodgkin lymphoma	201-201.9
Multiple myeloma	203-203.9
Leukemia	204, 205, 206, 207, 208-208.9
Acute lymphoid leukemia	204.0, 204.2
Acute myeloid leukemia	205.0, 205.2-205.3, 206.0, 207.0, 207.2-207.8

Map of ICD9 codes to GBD cause

GBD cause name	ICD codes
Chronic myeloid leukemia	205.1
Other leukemia	206.1, 207.1, 207.9
Uterine fibroids	218-219, 219.1-219.9, 236.0
Benign and in situ CNS neoplasms	225-225.9, 237-237.3, 237.5-237.9, 239.6
Myelodysplastic, myeloproliferative, and other hematopoietic neoplasms	238.4-238.9
Hypothyroidism	240-241.9, 243-243.9, 245-245.9
Hyperthyroidism	242-242.9, 775.3
Post procedural or drug treatment disorders	244.0-244.1, 244.3-244.8, 251.3, 253.7, 357.6, 518.7, 519.0, 536.4, 539-539.9, 551.2, 552.2, 553.2, 558.1, 564.2-564.4, 569.6, 579.3, 598.2, 779.4-779.5
Other nutritional deficiencies	244.2, 265-269.9, 281.0-281.9, 716.0
Other endocrine, metabolic, blood, and immune disorders	246-246.9, 251-251.2, 251.4-253.6, 253.8-256.3, 256.8-259.1, 259.3-259.9, 270-271.9, 273-273.9, 275-276, 277, 277.1-277.2, 277.4-277.9, 278.2-278.8, 286-286.5, 286.7-289.0, 289.4-289.7
Polycystic ovarian syndrome	256.4
Protein-energy malnutrition	260-263.9
Lipoprotein metabolism and other lipidaemias disorders	272-272.9
Cystic fibrosis	277.0
Obesity	278.0-278.1
Other hemoglobinopathies and hemolytic anemias	282-282.1, 282.7-283.9
G6PD deficiency	282.2-282.3
Thalassemias	282.4-282.5
Sickle cell disorders	282.6
Aplastic anemias	284-284.9

Map of ICD9 codes to GBD cause

GBD cause name	ICD codes
Alzheimer's disease and other dementias	290-290.9, 294.1-294.9, 331-331.2
Alcohol use disorders	291-291.9, 303-303.9, 305.0, 357.5, 790.3, E860-E860.1
Other drug use disorders	292-292.9, 304.1, 304.5-304.8, 305.3-305.4, 305.8, 760.7
Opioid use disorders	304.0, 305.5, E850.0-E850.2
Cocaine use disorders	304.2, 305.6
Cannabis use disorders	304.3, 305.2
Amphetamine use disorders	304.4, 305.7
Anorexia nervosa	307.1
H influenzae type B meningitis	320.0
Pneumococcal meningitis	320.1
Other meningitis	320.2-320.3, 320.5-320.8, 321-322.9
Sleep apnea	327.2-327.8
Other neurological disorders	330-330.9, 331.5-331.9, 333-334.9, 335.3, 336-337.9, 341-341.9, 349, 349.2-349.8, 353.8-353.9, 356-356.9, 357.0-357.1, 357.3-357.4, 357.7, 358-359.9, 775.2
Parkinson's disease	332-332.0
Motor neuron disease	335-335.2, 335.8-335.9
Multiple sclerosis	340-340.9
Idiopathic epilepsy	345-345.9
Adverse effects of medical treatment	349.0-349.1, 457.0, E870-E876.9, E878-E879.9, E930-E949.9
Otitis media	381-383.9
Rheumatic heart disease	391-391.9, 392.0, 393-398.9

Map of ICD9 codes to GBD cause

GBD cause name	ICD codes
Hypertensive heart disease	402-402.9
Chronic kidney disease due to hypertension	403-404.9
Acute myocardial infarction	410-411.9
Chronic ischemic heart disease	412-412.9, 414-414.9
Angina	413-413.9
Pulmonary Arterial Hypertension	416.0
Other musculoskeletal disorders	416.1, 437.4, 446-446.9, 710, 710.1-710.9, 732-732.9
Other cardiovascular and circulatory diseases (internal)	417-417.9, 420-420.9, 423, 423.1-423.9, 427.0-427.2, 427.6-427.8, 442-443, 447-450, 456, 456.3, 456.8-457, 457.1, 457.8-457.9, 459, 459.2-459.3
Endocarditis	421-421.9
Myocarditis	422-422.9
Non-rheumatic degenerative mitral valve disease	424.0
Non-rheumatic calcific aortic valve disease	424.1
Other non-rheumatic valve diseases	424.2-424.3, 424.8
Other cardiomyopathy	425.0-425.4, 425.7-425.8, 429.0
Alcoholic cardiomyopathy	425.5
Atrial fibrillation and flutter	427.3
Subarachnoid hemorrhage	430-430.9
Intracerebral hemorrhage	431-432.9, 437.2
Ischemic stroke	433-435.9, 437.0-437.1, 437.5-437.8
Lower extremity peripheral arterial disease	440.2, 440.4, 443.0-443.9

Map of ICD9 codes to GBD cause

GBD cause name	ICD codes
Aortic aneurysm	441-441.9
Phlebitis and thrombophlebitis	451-453.9, 459.1
Varicose veins	454-454.9, 456.4-456.6
Hemorrhoids, anal fissure, anal abscess, and fistula	455-455.9, 565-566.9, 569.1-569.4
Cirrhosis and other chronic liver diseases	456.0-456.2, 571-571.9, 572.2-573.0, 573.4-573.9
Other lower respiratory infections	466-469, 470.0, 480-480.0, 480.2-480.9, 482-482.1, 482.3-482.8, 483.0-483.9, 484.1-484.2, 484.6-484.7
Other chronic respiratory diseases	470, 470.9, 478.3-479, 495-495.9, 506-506.9, 508-509, 517-517.8, 518.6, 518.9, 519.1-519.4
Chronic diseases of upper respiratory system	471-474.9, 476-476.1, 478-478.2
Allergic disorders of upper respiratory system	477-477.9
Pneumococcal pneumonia	481-481.9
H influenzae type B pneumonia	482.2
Influenza	487-489
Chronic obstructive pulmonary disease	491-492.9, 496-499
Asthma	493-493.9
Coal workers pneumoconiosis	500-500.9, 501.0-501.9
Asbestosis	501
Silicosis	502-502.9, 503.0, 503.9
Other pneumoconiosis	503, 503.1, 504-504.9
Esophageal diseases	530-530.0, 530.2-530.6
Peptic ulcer disease	531-534.9

Map of ICD9 codes to GBD cause

GBD cause name	ICD codes
Gastritis and duodenitis	535-535.9
Other digestive diseases	536-536.1, 537-537.6, 537.8, 538, 543-543.9, 564.1, 564.5, 569.7, 579, 579.1-579.2, 579.8-579.9
Appendicitis	540-542.9
Inguinal hernia	550-550.9
Femoral hernia	551-551.0, 552.0, 553.0, 553.6
Abdominal and other hernia	551.1, 552.1, 553.1
Diaphragmatic hernia	551.3-552, 552.3, 553.3
Inguinal, femoral, and abdominal hernia	552.4-553, 553.8-553.9
Crohn's disease	555-555.9
Ulcerative colitis	556-556.9, 558.0
Vascular intestinal disorders	557-557.9
Inflammatory bowel disease	558, 569.5
Diarrheal diseases	558.2-558.9
Paralytic ileus and intestinal obstruction	560-560.3, 560.8-560.9
Diverticular disease of intestines	562-562.1
Constipation	564-564.0, 564.6-564.7
Gallbladder and biliary diseases	574-576.9
Pancreatitis	577-577.9, 579.4
Celiac disease	579.0
Acute glomerulonephritis	580-580.9

Map of ICD9 codes to GBD cause

GBD cause name	ICD codes
Chronic kidney disease due to glomerulonephritis	581-583.9
Chronic kidney disease	585-585.9
Other urinary diseases	588-588.9, 593-593.8, 596-596.9, 599.8, 608.2
Chronic kidney disease due to other and unspecified causes	589-589.9, 753-753.3
Urinary tract infections and interstitial nephritis	590-590.9, 595-595.9, 597-597.9, 599.0
Urolithiasis	592-592.9, 594-594.9, 788.0
Urethral stricture, phimosis, and paraphimosis	598-598.1, 598.8-599, 599.1-599.6
Prostatitis and epididymitis	601-602.9, 604-604.9
Endometriosis	617-617.9
Genital prolapse	618-618.9
Other gynecological diseases	620-620.9, 621.4-621.9, 622.3-622.6, 629-629.8
Spontaneous abortion	630-632.9, 634-634.9, 646.3
Ectopic pregnancy	633-633.9
Induced abortion	635-636.9, 638-638.9
Maternal hemorrhage	640-641.9, 661-661.9, 665, 666-666.9
Maternal hypertensive disorders	642-642.9
Indirect maternal deaths	646-646.2, 646.4-649.9
Maternal obstructed labor and uterine rupture	652-653.9, 660-660.9, 665.0-665.3
Maternal sepsis and other maternal infections	659.3, 670-670.9
Cellulitis	681-682.9

Map of ICD9 codes to GBD cause

GBD cause name	ICD codes
Other skin and subcutaneous diseases	694-695.3
Systemic and discoid lupus erythematous	695.4-695.5, 710.0
Decubitus ulcer	707-707.9
Pyogenic and reactive arthritis	711-711.9
Rheumatoid arthritis	714-714.3, 714.8-714.9
Osteomyelitis non-traumatic	730.1
Osteoporosis and related pathological fracture	733.0-733.1
Neural tube defects	740-741.9, 742.0
Other congenital birth defects	742, 742.1-742.4, 742.8-744.9, 748-748.9, 757-757.9, 759.0-759.8
Congenital musculoskeletal and limb anomalies	742.5, 754-756.5, 756.8-756.9
Congenital heart anomalies	745-747.9
Orofacial clefts	749-749.0, 749.2-749.9
Digestive congenital anomalies	750-751.9, 756.6-756.7
Urogenital congenital anomalies	752-752.9, 753.4-753.9
Other chromosomal abnormalities	758, 758.1-758.6, 758.8-758.9
Down syndrome	758.0
Klinefelter syndrome	758.7
Other neonatal disorders	760-760.6, 760.8-761, 761.2-761.6, 766-766.9, 770, 771, 772-772.0, 775-775.0, 775.4-776.5, 776.7-777, 778-779, 779.3, 779.6-779.8
Neonatal preterm birth	761.0-761.1, 764-765.9, 769-769.9, 770.2-770.9, 772.1-772.9, 776.6, 777.0-777.9
Neonatal encephalopathy due to birth asphyxia and trauma	761.7-763.9, 767-768, 768.2-768.9, 770.1, 779.0-779.2

Map of ICD9 codes to GBD cause

GBD cause name	ICD codes
Still Born	768.0-768.1
Neonatal sepsis and other neonatal infections	771.4-771.9
Hemolytic disease and other neonatal jaundice	773-774.9
Diabetes mellitus type 1	775.1
Sudden infant death syndrome	798-798.0
Other transport injuries	E800-E800.2, E801-E801.2, E802-E802.2, E803-E803.2, E804-E804.2, E805-E805.2, E806-E806.2, E807-E807.2, E820.7, E821.7, E826.2, E827.2, E828.2, E830-E838.9, E840-E849.9, E929.1
Cyclist road injuries	E800.3, E801.3, E802.3, E803.3, E804.3, E805.3, E806.3, E807.3, E810.6, E811.6, E812.6, E813.6, E814.6, E815.6, E816.6, E817.6, E818.6, E819.6, E820.6, E821.6, E822.6, E823.6, E824.6, E825.6, E826.1
Motor vehicle road injuries	E810.0-E810.1, E811.0-E811.1, E812.0-E812.1, E813.0-E813.1, E814.0-E814.1, E815.0-E815.1, E816.0-E816.1, E817.0-E817.1, E818.0-E818.1, E819.0-E819.1, E820.0-E820.1, E821.0-E821.1, E822.0-E822.1, E823.0-E823.1, E824.0-E824.1, E825.0-E825.1
Motorcyclist road injuries	E810.2-E810.3, E811.2-E811.3, E812.2-E812.3, E813.2-E813.3, E814.2-E814.3, E815.2-E815.3, E816.2-E816.3, E817.2-E817.3, E818.2-E818.3, E819.2-E819.3, E820.2-E820.3, E821.2-E821.3, E822.2-E822.3, E823.2-E823.3, E824.2-E824.3, E825.2-E825.3
Other road injuries	E810.4-E810.5, E811.4-E811.5, E812.4-E812.5, E813.4-E813.5, E814.4-E814.5, E815.4-E815.5, E816.4-E816.5, E817.4-E817.5, E818.4-E818.5, E819.4-E819.5, E820.4-E820.5, E821.4-E821.5, E822.4-E822.5, E823.4-E823.5, E824.4-E824.5, E825.4-E825.5, E826.3-E826.4, E827.3-E827.4, E828.4, E829.4
Pedestrian road injuries	E810.7, E811.7, E812.7, E813.7, E814.7, E815.7, E816.7, E817.7, E818.7, E819.7, E822.7, E823.7, E824.7, E825.7, E826.0, E827.0, E828.0, E829.0
Drug use disorders	E850
Poisoning by other means	E856-E857.0, E860.2-E861.9, E864-E865.9, E867-E867.0, E869.0-E869.8, E929.2
Poisoning by carbon monoxide	E862-E862.9, E868-E869, E869.9
Poisoning by pesticides	E863-E863.9
Falls	E880-E886.9, E888-E888.9, E929.3
Fire, heat, and hot substances	E890-E899.0, E924-E924.9, E929.4
Environmental heat and cold exposure	E900-E902.9, E926-E926.9, E929.5
Other unintentional injuries	E903-E904.9, E913.2-E913.3, E923-E923.9, E927-E928.0, E928.8
Venomous animal contact	E905

Map of ICD9 codes to GBD cause

GBD cause name	ICD codes
Contact with snakes and lizards	E905.0
Contact with spiders	E905.1
Contact with scorpions	E905.2
Contact with hornets, wasps and bees	E905.3
Contact with other venomous	E905.4-E905.9
Non-venomous animal contact	E906-E906.9
Victim of lightning	E907-E907.0
Cataclysmic storm	E908-E908.1, E908.3-E908.9
Flood	E908.2
Earthquake	E909-E909.0
Volcanic eruption	E909.1
Avalanche, landslide and other earth movements	E909.2
Other forms of forces of nature	E909.3-E909.9
Drowning	E910-E910.9
Pulmonary aspiration and foreign body in airway	E911-E913.1, E913.8-E913.9
Foreign body in other body part	E914-E915.0
Other exposure to mechanical forces	E916-E921.9, E928.1-E928.6
Unintentional firearm injuries	E922-E922.9, E928.7
Electrocution	E925-E925.9
Self-harm by other specified means	E950-E952.9, E954, E956-E958.0, E958.2-E959

Map of ICD9 codes to GBD cause

GBD cause name	ICD codes
Self-harm by hanging, strangulation, and suffocation	E953-E953.9
Self-harm by firearm	E955-E955.9
Self-harm by fire, heat, and hot substances	E958.1
Sexual violence	E960-E960.1
Physical violence by other means	E961-E964, E965.5-E965.9, E967-E969
Physical violence by firearm	E965-E965.4
Physical violence by sharp object	E966
Police conflict and executions	E970-E978
Terrorism	E979-E979.9
Military operations	E990-E999.0
Conflict and terrorism	E999.1

Appendix Figure 4

ICD10 Codes by Garbage Package and Class		
**Garbage Package**	**Garbage Class**	**ICD Codes**
All, Ill Defined code for causes of death	1	A59-A59.9, A71-A71.9, A74-A74.0, B07-B07.9, B30-B30.9, B35-B36.9, B85-B85.4, B87-B88.9, B94-B94.0, D68, E15-E16, E50-E50.9, E64, E64.1, F06, F06.3-F06.4, F07, F07.2, F09-F09.9, F30-F49, F51-F99.0, G32-G32.8, G43-G44.2, G44.4-G44.8, G47-G47.2, G47.4-G47.9, G50-G60.9, G62-G62.0, G62.2-G65.2, G89-G89.4, G99-H05, H05.2-H69.9, H71-H99, K00-K19, K30, L20-L30.9, L40-L50.9, L52-L54.8, L56-L56.2, L56.4-L56.5, L57-L57.9, L59-L68.9, L70-L76.8, L80-L87.9, L90-L92.9, L94-L96, L98, L98.5-L99.8, M04, M10-M12.0, M12.2-M29, M37-M39, M43, M43.2-M49, M49.2-M65, M65.1-M71, M71.2-M72.4, M72.8-M73, M73.8-M79.9, M83-M85.9, M87, M87.2-M87.9, M89, M89.1-M89.4, M90-M99.9, N32, N32.8-N33.8, N35-N35.9, N37-N37.8, N39, N39.3-N39.8, N42-N44, N44.1-N44.8, N46-N48.9, N50-N53.9, N61-N64.9, N91-N91.5, N95, N95.1-N95.9, N97-N97.9, R07-R07.0, R08-R09, R09.3, R12-R12.0, R14-R15.9, R19-R19.6, R19.8-R23, R23.1-R30.9, R32-R39.9, R41-R49.9, R51-R53.8, R55-R55.0, R58-R63.3, R63.5, R63.8-R65.1, R66-R72.9, R74-R78, R78.6-R94.8, R96-R99.9, U05, U08-U81, U89-U99, Z00-Z15.8, Z17-ZB0
Senility	1	R54-R54.9
Unspecified Infectious Diseases	2	A14-A14.9, A29-A30.9, A45-A45.9, A47, A61-A62, A72-A73, A76, A97, B11-B14, B28-B29, B31-B32.4, B61-B62, B68-B68.9, B73-B74.2, B76-B76.9, B78-B81.8, B84, B92-B94, B94.8-B95, B95.6-B97.1, B97.3, B97.7-B99.9
Unspecified Bacterial Diseases	2	A48, A48.8-A49, A49.3-A49.9
Unspecified STD	3	A64-A64.0
Unspecified Hemorrhagic Fever	3	A99-A99.0
Unspecified Viral Diseases	2	B08-B09, B34-B34.1, B34.3-B34.9, G93, G93.3
Unspecified Protozoal Diseases	4	B64
Unspecified Intestinal Parasite	4	B82-B83, B83.9
Unspecified Parasitic Diseases	3	B89
Typhoid or paratyphoid fevers	3	A01
Haemophilus influenza infection, unspecified site	3	A49, A49.2
Unspecified Malaria	4	B54-B54.0, P37, P37.3-P37.4
Unspecified Oropharynx Cancer	3	C1, C14-C14.9, D0-D00.0, D10, D10.9, D37-D37.0
Unspecified GI Cancer	3	C2, C26-C3, C35-C36, D0-D00, D01, D01.4-D01.9, D13, D13.9, D37, D37.6-D37.9, D4, D49-D49.0
Unspecified Respiratory Cancer	3	C3, C39-C39.9, D0, D02, D02.4-D02.9, D14, D14.4, D38, D38.6, D4, D49, D49.1
Unspecified Uterus Cancer	3	C5, C55-C55.9
Unspecified Female Genital Cancer	3	C5, C57, C57.9, C59-C6, D0, D07, D07.3, D28, D28.9, D39-D39.0, D39.7, D39.9, N84, N84.2-N84.8
Unspecified Male Genital Cancer	3	C6, C63, C63.9, D0, D07, D07.6, D29, D29.9, D4-D40, D40.9
Unspecified Urinary Cancer	3	C6, C68, C68.9, D0, D09, D09.1, D30, D30.9, D4, D41, D41.9
Unspecified Endocrine Cancer	3	C7, C75, C75.9, D4, D44, D44.9, D49, D49.7, E34-E34.0
Unspecified Site Cancer	3	C4, C42, C7, C76, C76.7-C77, C77.3-C77.4, C77.8-C78, C79, C79.2-C80.9, C87, C97-D0, D08-D09, D09.7, D09.9, D36-D36.0, D36.9, D4, D48, D48.7-D49, D49.8-D49.9, D54
Head and Neck Cancer	3	C7, C76-C76.1, C77-C77.1, C78-C78.3, D17-D21.9
Abdomen and Pelvis Cancer	3	C7, C76, C76.2-C76.3, C77, C77.2, C77.5, C78, C78.4-C79.1
Unspecified genital Cancer	3	D0, D07, D4, D49, D49.5
Acquired hemolytic anemia	2	D59, D59.4, D59.8-D59.9
Unspecified Blood Diseases	3	D75, D75.9, D79, D85, D87-D88, D90-D99
Unspecified Thyroid Diseases	3	E07, E07.8-E07.9
Unspecified Diabetes Related cause	3	E08-E08.9
Unspecified Endo/Metabolic Diseases	3	E17-E19, E35, E37-E39, E47-E49, E62, E69, E90-E998
Unspecified Endocrine Diseases	3	E34, E34.9-E35.8
Unspecified Mental/Brain Disorders	3	F06-F06.1, F06.5-F07.0, F07.8-F08
Unspecified Eating Disorders	3	F50, F50.8-F50.9
Unspecified Meningitis	4	G00, G00.9-G03, G03.9
Unspecified CNS Infection	3	G09-G09.9
Unspecified CNS Diseases	3	G15-G19, G21, G21.2, G21.4-G22.0, G27-G29, G33-G34, G38-G39, G42, G48-G49, G66-G69, G74-G79, G84-G88
External Causes UDI, type unspecified	2	G44, G44.3, G91, G91.3, R58, TO7., W47-W47., Y24, Y24.5-Y24.7, Y25, Y25.2, Y26, Y26.3, Y27, Y27.4-Y27.5, Y28, Y28.3, Y28.5, Y29, Y29.3, Y33-Y34.9, Y86-Y87, Y87.2, Y89, Y89.9-Y99.9
Unspecified Brain Diseases	3	G93, G93.8-G94, G96-G96.9, G98-G98.9
CNS Fluid Diseases	3	E87, E87.7
Unspecified CNS sign and symptom	3	F04-F05.9
CNS Abscess	1	G06-G08.0
Unspecified cardiovascular diseases	3	I00-I00.0, I03, I04, I14, I16-I18, I19, I29-I29.9, I51, I51.6, I52-I59, I90-I94, I96-I96.9, I98, I98.4-I98.8, I99-ID5.9
Cerebral Cysts	2	G93-G93.0

ICD10 Codes by Garbage Package and Class

Garbage Package	Garbage Class	ICD Codes
Hypertension	2	I10-I10.9, I15-I15.9, I67, I67.4, R03-R03.0, R04-R04.0
Pulmonary Embolism	1	I26-I26.9
right heart failure and pulmonary heart disease	2	I27, I27.8-I27.9
Sepsis (Non- maternal and neonatal sepsis)	1	A40-A41.9, A48-A48.0, A48.3, A49-A49.1, D65-D65.9, I76, R02-R02.9, R50-R50.1, R50.8-R50.9, R56-R56.0, R65, R65.2
Amyloidosis	1	E85, E85.3-E85.9
Cerebral Palsy	1	G80-G80.9, G82, G82.1, G82.4, G83-G83.0, G83.8
Left heart failure	1	I50, I50.2-I50.4
Unspecified Heart Diseases	3	I51, I51.7-I51.9
Unspecified type of Stroke	4	I64-I64.9, I67, I67.8-I68, I68.8-I69, I69.4-I69.9
Atherosclerosis	2	I70-I70.0
Arterial Embolism	2	I74-I75.8
Unspecified upper respiratory infectious	3	J02, J02.9-J03, J03.9-J04, J04.3, J06, J06.9
Unspecified acute respiratory infectious	4	J07-J08, J23-J29
Unspecified lower respiratory infectious	4	J15, J15.9, J17-J19.6, J22-J22.9, P23, P23.5-P23.9
Unspecified chronic respiratory diseases	3	J48-J59, J71-J79, J81, J81.9, J83, J85, J85.9, J87-J90, J90.9, J93, J93.6, J97-J98.0, J98.4-J99.8
Unspecified Pneumoconiosis	4	J64-J64.9
Pneumonitis	1	J69-J69.9, J85-J85.3
Chronic respiratory failure	1	J96, J96.1-J96.8
Unspecified Digestive Diseases	3	K31, K31.9-K34, K39, K47, K48-K49, K53-K54, K69, K78-K79, K84, K87-K89, K92, K92.9-K93, K96-K99
Unspecified Intestine Diseases	3	K21-K22, K22.7, K63-K63.4, K63.8-K63.9
Alcoholic hepatic failure	3	K70, K70.4-K70.9
Gastrointestinal Bleeding	2	K92-K92.2
Unspecified Skin Diseases	3	L06-L07, L09, L15-L19, L31-L39, L69, L77-L79
Osteomyelitis	1	M86-M86.2, M86.5-M86.9
Unspecified Urinary Diseases	3	N09, N24, N28, N28.8-N28.9, N38-N39, N39.9-N40.9, N54-N59, N66-N69, N78-N79, N84, N84.9-N86, N88-N90.9
Urinary Obstruction Diseases	3	N13-N13.5, N13.7-N13.9, R31-R31.9
Acute kidney failure	1	N17, N19-N19.9
Fistula	1	N32, N32.1-N32.2, N82-N82.9
Unspecified Gynecologic Diseases	3	N92-N95.0
Maternal Complication	3	O08-O08.9
Unspecified Maternal Diseases	3	O17-O19, O27, O37-O39, O49-O59, O78-O79, O93-O95.9
Unspecified Neonatal Diseases	3	P06, P16-P17, P18, P30-P34.2, P40-P49, P62-P69, P73, P79, P82, P85-P89, P96, P96.9-P99.9
Unspecified Congenital Diseases	3	Q08-Q10.3, Q19, Q29, Q36-Q36.9, Q46-Q49, Q88-Q89, Q89.9, Q94
Unspecified Chromosomal Diseases	3	Q99, Q99.9
Unspecified sign and symptom for Heart diseases	3	R00-R01.2, R07, R07.1-R07.9
Unspecified sign and symptom for Respiratory diseases	2	R05-R06.9, R23-R23.0
Unspecified sign and symptom for GI diseases	2	R13-R13.9
Nausea and Vomiting	1	R11-R11.9
Exposure to unspecified factor X59	2	W47, W48, W63, W71-W72, W82, W95-W97, W98, X07, X55, X56, X59-X59.9
Unspecified Transport Injuries	4	V99-V99.0, Y85-Y85.9
Unspecified Road Injuries	4	V89-V89.9
Unspecified Traffic Injuries	4	V87-V87.1, V87.4-V88.1, V88.4-V88.9
Self-harm by unspecified means	4	X84-X84.9
Assault by unspecified means	4	Y09-Y09.9
Undetermined intent Poisoning by no opioid analgesics	1	Y10-Y10.9
Undetermined intent Poisoning by antiepileptic and psychotropic drugs	1	Y11-Y11.9
Undetermined intent Poisoning by narcotics and psychodysleptics drugs	1	Y12-Y12.9
Undetermined intent Poisoning by autonomic nervous system drugs	1	Y13-Y13.9
Undetermined intent Poisoning by unspecified drugs and biological drugs	1	Y14-Y14.9
Undetermined intent Poisoning by solvents and halogenated hydrocarbons	1	Y16-Y16.9
Undetermined intent Poisoning by other gases and vapors	1	Y17-Y17.9
Undetermined intent Poisoning by pesticides	1	Y18-Y18.9
Undetermined intent Poisoning by unspecified chemicals and noxious substances	1	Y19-Y19.9
Undetermined intent Strangulation	2	W76-W76.9, Y20-Y20.9
Undetermined intent Drowning	2	Y21-Y21.9
Undetermined intent shooting by Handgun Firearm	2	Y22-Y22.9
Undetermined intent shooting by rifle and larger firearm	2	Y23-Y23.2, Y23.4-Y23.7

ICD10 Codes by Garbage Package and Class		
**Garbage Package**	**Garbage Class**	**ICD Codes**
Undetermined intent shooting by unspecified firearm	2	Y23, Y23.3, Y23.8-Y24.4, Y24.8-Y24.9
Undetermined intent of Explosion	2	Y25-Y25.1, Y25.4-Y25.9
Undetermined intent of fire and flames	2	Y26-Y26.2, Y26.4-Y26.9
Undetermined intent of Hot Objects	2	Y27-Y27.3, Y27.6-Y27.9
Undetermined intent of Sharp Objects	2	Y28-Y28.2, Y28.4, Y28.6-Y28.9
Undetermined intent of Blunt Objects	2	Y29-Y29.0
Undetermined intent of Moving Objects	2	Y31-Y31.9
Undetermined intent of fall	2	Y29, Y29.1-Y29.2, Y29.4-Y30.9
Undetermined intent Poisoning by multiple or unspecified drug	1	F19-F19.9, X40-X44.9, X46-X46.9, X49-X49.9, X55-X55.
Undetermined intent of Crashing	2	Y32-Y32.9
Anemia in neoplastic Diseases	1	D63-D63.0
Anemia Unspecified	1	D50-D50.0, D50.9, D62-D63, D63.8-D64, D64.1-D64.9, D69, D69.9
Unspecified Cardiomyopathy	4	I42-I42.0, I42.9
Myocardial Degeneration	4	I51, I51.5
Female pelvic inflammatory diseases	2	N70-N71.9, N73-N74.0, N74.2-N74.8
Schizophrenia	1	F06, F06.2, F20-F23.9, F25-F29.9
Diabetes unspecified type	4	E12-E12.1, E12.3-E13.1, E13.3-E14.1, E14.3-E14.9, R73-R73.9
CKD due to diabetes Unspecified type	4	E12, E12.2, E13, E13.2, E14, E14.2
Unspecified Bronchitis and Bronchiectasis	3	J40-J40.9, J47-J47.9
Valve Disorder and Endocarditis	4	I37, I37.9
Primary or secondary Liver Cancer Unspecified	3	C2, C22, C22.9
Eye Unspecified Site Cancer	4	C6, C69, C69.9
Adrenal Unspecified Site Cancer-parent cause	3	C7, C74-C74.0
Chronic lymphocytic leukemia by age	4	C91, C91.1
Lymphoid leukemia unspecified by age	4	C91, C91.4-C91.5, C91.7-C91.9
myeloid leukemia by age	4	C92, C92.7-C93, C93.2, C93.5-C93.7, C93.9
Adrenal Unspecified Site Cancer in medulla or cortex	3	C7, C74, C74.1
Adrenal Site Cancer unspecified part of adrenal gland	3	C7, C74, C74.9
Heart failure unspecified right or left	2	I50, I50.8-I50.9, J81, J81.1
Hepatitis Unspecified	3	B17, B17.1, B17.8-B17.9, B19-B19.0, B19.2-B19.9, B94, B94.2
Hepatic Failure	1	K71-K71.6, K71.8-K72.9, R16-R18.9
Fluid, Electrolyte, Acid Base Disorders	1	E86-E87.6, E87.8-E87.9
Cardiac rhythm disorders	3	I44-I45.9, I49-I49.9
Assigned death to tobacco	2	F17-F17.9
Intermediate cause for CNS	1	G91-G91.2, G91.4-G93, G93.1-G93.2, G93.4-G93.6, G94-G94.8
Cachexia	1	R63, R63.4, R63.6, R64
Peritonitis & Acute Abdomen	1	K65-K66.1, K66.9, K68-K68.9, R10-R10.9
Acute Respiratory Failure	1	J80-J81.0, J96-J96.0, J96.9, J98, J98.1-J98.3
Shock, Cardiac Arrest, Coma	1	I46-I46.9, I95-I95.1, I95.8-I95.9, R03, R03.1, R09-R09.0, R09.2, R09.8, R40-R40.4, R55, R56, R56.1-R57.9
hepatitis B unspecified	4	B16, B16.9
Plegia	1	G81-G82.0, G82.2-G82.3, G82.5-G83, G83.1-G83.5, G83.9
Pleurisy, Pyothorax	1	J86-J86.9, J90-J90.0, J94-J94.1, J94.8-J94.9, R09, R09.1
Pneumothorax	1	I31, I31.2-I31.4, J93-J93.1, J93.8-J94, J94.2, R04, R04.1-R04.9
Non-follicular lymphoma, unspecified	3	C8, C83, C83.9, C85, C85.1, C85.9
Pneumoconiosis associated with tuberculosis	3	J65-J65.0
upper and lower limb cancer	3	C7, C76, C76.4-C76.5
MDS not classified	3	C94, C94.6

ICD9 Codes by Garbage Package and Class		
**Garbage Package**	**Garbage Class**	**ICD Codes**
All, Ill Defined code for causes of death	1	076-078.2, 110-111.9, 125-125.3, 126-127, 127.2-127.9, 131-133, 133.8-134.9, 139, 139.1, 139.9, 247-248, 264-264.9, 274-274.9, 289, 289.1-289.3, 293, 294-294.0, 296-302.9, 306-307.0, 307.2-307.4, 307.6-319.9, 327-327.1, 328-329, 338-339.1, 339.3-339.8, 346-346.9, 350-353.6, 354-355.9, 360-362, 362.1-376, 376.2-380.9, 384-389.9, 520-529.9, 536, 536.3, 536.8-537, 537.7, 537.9, 564, 564.8-564.9, 603-603.9, 605-608.1, 608.3-609, 611-612.1, 615-616.9, 621-621.3, 622-622.0, 622.8-623.6, 623.8-624.5, 624.8-629, 629.9, 690-693.9, 695, 695.8-706.9, 708-709.9, 712-713.8, 715-716, 716.2-721.6, 721.8-730, 730.3, 731-731.9, 733, 733.2-734.2, 737-738, 738.2-739.9, 780, 780.1, 780.4-780.5, 780.7-782.3, 782.6-783.1, 783.3-784.6, 784.9, 786, 786.6, 786.8, 787, 787.3-788, 788.3-789, 790-790.1, 790.4-796.1, 796.3-796.9, 798, 798.1-799, 799.2-799.3, 799.5-799.9
Senility	1	797-797.9
Unspecified Bacterial Diseases	2	041, 041.2-041.9
Unspecified Leishmaniasis	3	085, 085.9
Unspecified Infectious Diseases	2	000-000.9, 030-030.9, 067-069, 078, 078.8-079, 079.8-079.9, 089-089.9, 105-109.9, 119, 136, 136.8-136.9, 139, 139.8
Sepsis (Non- maternal and neonatal sepsis)	1	038-038.9, 040-040.0, 041, 041.1, 286, 286.6, 780, 780.6, 785, 785.4
Unspecified enteric Infection	3	002
Unspecified Oropharynx Cancer	3	149-149.9, 210, 230-230.0, 235, 235.1
Unspecified GI Cancer	3	159-159.9, 211, 211.9, 230, 230.9, 235, 235.2-235.3, 235.5, 239-239.0
Unspecified Respiratory Cancer	3	165-165.9, 212, 212.9, 231, 231.8-231.9, 235, 235.9, 239, 239.1
Unspecified Uterus Cancer	3	179, 179.1-179.6
Unspecified Female Genital Cancer	3	183, 183.9-184, 184.5, 184.9, 221, 221.9, 233, 233.3, 236, 236.3, 623, 623.7, 624, 624.6
Unspecified Urinary Cancer	3	189, 189.9, 223, 223.9, 233, 233.9, 236, 236.9-237, 237.4
Unspecified Male Genital Cancer	3	187, 187.9, 222, 222.9, 233, 233.6, 236, 236.6
Unspecified genital Cancer	3	233, 236, 239, 239.5
Unspecified Endocrine Cancer	3	239, 239.7
Unspecified Site Cancer	3	166-169, 177-179.0, 179.9, 195, 195.8-196, 196.3-197, 198, 198.2-199.9, 209, 209.2-209.3, 209.6-209.7, 214, 214.1, 214.4-215, 215.2-215.3, 215.7-216, 216.5-216.9, 229, 229.1, 229.9, 234, 234.9, 239, 239.8-239.9, 259, 259.2
Head and Neck Cancer	3	195-195.0, 196-196.0, 214-214.0, 215-215.0, 216-216.4
Thorax Cancer	3	195, 195.1, 196, 196.1, 197-197.3, 214, 214.2, 215, 215.4
Abdomen and Pelvis Cancer	3	195, 195.2-195.3, 196, 196.2, 197, 197.4-198.1, 214, 214.3, 215, 215.5-215.6
Unspecified Thyroid Diseases	4	244, 244.9
Unspecified Diabetes Related cause	3	249-249.9
Amyloidosis	1	277, 277.3
Unspecified Endo/Metabolic Diseases	3	278
Unspecified Meningitis	4	320, 320.9
Unspecified CNS Infection	3	357, 357.8-357.9
Unspecified Heart Diseases	3	399-400.0, 406-409.4, 418-419.9, 429, 429.2-429.9
left heart failure	1	428-428.4
Pneumonitis	1	507-507.9, 513-513.1
Unspecified cardiovascular diseases	3	459, 459.5-459.9, 519, 519.8-519.9
Unspecified Neonatal Diseases	3	779, 779.9
Exposure to unspecified factor X59	2	839, 859, 877, 887-887.0, 928, 928.9-929, 929.9, E83, E839, E85, E859, E87, E877, E88, E887-E887.0, E928, E928.9-E929, E929.8-E929.9
Undetermined intent of fall	3	987-987.2, 987.9, E987-E987.9
Intermediate cause for CNS	3	293-293.9, 331, 331.3-331.4, 332, 332.1-332.9, 347-349, 349.9
Unspecified Mental/Brain Disorders	2	304, 304.9
CNS Abscess	1	324-326.9, 437, 437.3
Cerebral Palsy	1	343-343.9
Hypertension	2	401-401.9, 405-405.9, 784, 784.7, 796, 796.2
Atherosclerosis	2	440-440.1, 440.3, 440.8-440.9
Pulmonary Embolism	1	415-415.9
right heart failure and pulmonary heart disease	2	416, 416.2-416.9
Unspecified type of Stroke	4	436-437, 437.9-439.6
Arterial Embolism	2	444-445.8
Unspecified Hemorrhage	1	459-459.0
Unspecified upper respiratory infectious	3	464, 464.5, 465, 465.9
Unspecified lower respiratory infectious	4	482, 482.9-483, 484, 484.8-486.9, 770-770.0
Unspecified Pneumoconiosis	3	505-505.9

ICD9 Codes by Garbage Package and Class		
**Garbage Package**	**Garbage Class**	**ICD Codes**
Acute Respiratory Failure	2	514-515.9, 518-518.0, 518.4-518.5
Interstitial Emphysema	1	518, 518.1-518.2
Chronic respiratory failure	2	518, 518.8
Unspecified sign and symptom for GI diseases	2	536, 536.2, 787-787.2
Unspecified Intestine Diseases	3	530, 530.1, 530.7-530.9, 544-549, 559-560, 560.4-560.7, 561-562, 562.2-563, 569, 569.8-569.9
Liver Abscess	1	572-572.1
Gastrointestinal Bleeding	2	578-578.9
Urinary Obstruction Diseases	3	591-591.9, 600-600.9, 788, 788.1-788.2
Fistula	1	619-619.9
Unspecified Urinary Diseases	3	593, 593.9, 599, 599.9
Ur Bleeding	2	599, 599.7
Maternal Complication	3	637-637.9, 639-639.9
Osteomyelitis	1	730-730.0, 730.2, 730.7-730.9
Unspecified Congenital D	3	749, 749.1, 759, 759.9
Unspecified sign and symptom for Heart diseases	3	785-785.3, 786, 786.5
Respiratory Bleeding	2	784, 784.8, 786, 786.3
Unspecified sign and symptom for Respiratory diseases	3	782, 782.5, 786-786.2, 786.4, 786.7, 786.9
Cachexia	1	783, 783.2, 799, 799.4
Motor vehicle collision with train	4	810, E810, E810.8-E810.9
Motor vehicle with another motor vehicle	4	811, E811, E811.8-E811.9
Other motor vehicle another motor vehicle	4	812, E812, E812.8-E812.9
Motor vehicle other vehicle	4	813, 813.8-813.9, E813, E813.8-E813.9
Motor vehicle with pedestrian	4	814, E814, E814.8-E814.9
Other motor vehicle on the highway	4	815, E815, E815.8-E815.9
Motor vehicle without collision on the highway	4	816, E816, E816.8-E816.9
Non-collision motor vehicle while boarding or alighting	4	817, E817, E817.8-E817.9
Other Non-collision motor vehicle traffic accident	4	818, E818, E818.8-E818.9
Motor vehicle accident of unspecified nature	4	819, E819, E819.8-E819.9
Non-traffic accident involving motor driven snow vehicle	4	820, 820.8-820.9, E820, E820.8-E820.9
Non-traffic accident other off-road motor vehicle	4	821, E821, E821.8-E821.9
Other motor vehicle Non-traffic accident with moving object	4	822, E822, E822.8-E822.9
Other motor vehicle Non-traffic accident with stationary object	4	823, 823.8-823.9, E823, E823.8-E823.9
Other motor vehicle Non-traffic while boarding and alighting	4	824, 824.8-824.9, E824, E824.8-E824.9
Other motor vehicle Non-traffic accident of other and unspecified nature	4	825, E825, E825.8-E825.9
Pedal cycle accident	4	826, E826, E826.8-E826.9
Animal-drawn vehicle accident	4	827, E827, E827.8-E827.9
Accident involving animal being ridden	4	828, E828, E828.8-E828.9
Other road vehicle accidents	4	829, E829, E829.8-E829.9
Other specified person with railway accident with rolling stock	4	800, 800.8-800.9, E80-E800, E800.8-E800.9
Other specified person -Railway accident without antecedent collision	4	802, 802.8-802.9, E80, E802, E802.8-E802.9
Unspecified person in railway accident other object	4	801, 801.8-801.9, E80, E801, E801.8-E801.9
Unspecified person in railway accident involving explosion fire or burning	4	803, 803.8-803.9, E80, E803, E803.8-E803.9
Unspecified person in fall in on or from railway train	4	804, 804.8-804.9, E80, E804, E804.8-E804.9
Unspecified person in Hit by rolling stock	4	805, 805.8-805.9, E80, E805, E805.8-E805.9
Unspecified person in Other specified railway accident	4	806, 806.8-806.9, E80, E806, E806.8-E806.9
Unspecified person in Railway accident of unspecified nature	4	807, E80, E807, E807.8-E807.9
Unspecified person in Unspecified Road Injuries	4	808, 809, 929-929.0, E80, E808-E809, E929-E929.0
Undetermined intent Poisoning by chemicals and substances	1	980-980.9, E980-E980.9
Undetermined intent Poisoning by gases and oil	1	981, 982-982.1, 982.8, E981-E982.9
Undetermined intent Strangulation	2	983-983.1, 983.9, E983-E983.9
Undetermined intent Drowning	2	984, E984
Undetermined intent shooting by unspecified firearm	2	985-985.6, E985-E985.7
Undetermined intent of Sharp Objects	3	986, E986
Undetermined intent of fire and flames	2	988, 988.1, E988, E988.1
Undetermined intent of Crashing	2	988, 988.6, E988, E988.5-E988.6
External Causes UDI, type unspecified	2	000-001.1, 002-003.2, 003.9-004.3, 004.9-005.4, 005.9-006.6, 006.9-008.4, 008.9-009.5, 009.9-010.3, 010.9-011.1, 011.9-012.2, 012.9-013.5, 013.8-014.0, 014.9-015.2, 015.9-016.2, 016.9-017.0, 017.9-018.3, 019, 019.9, 029-030, 339, 339.2, 714, 714.4, 716, 716.1, 721, 721.7, 735-736.9, 738-738.1, 988-988.0, 988.2, 988.8-989, E000-E80, E83, E85, E87, E88, E988-E988.0, E988.2-E988.4, E988.7-E989

ICD9 Codes by Garbage Package and Class		
Garbage Package	Garbage Class	ICD Codes
Undetermined intent Poisoning by multiple or unspecified drug	1	305, 305.9, 850, 850.3-851.0, 852-854.1, E85-E850, E850.3-E854.8
Unspecified blood neoplasm	4	238, 289, 289.8-289.9
Anemia Unspecified	1	280-281, 285-285.9
Unspecified Cardiomyopathy	4	425, 425.4, 425.9, 429, 429.1
Female pelvic inflammatory diseases	2	613-614.9
Schizophrenia	1	295-295.9
Diabetes unspecified type	4	250-250.9, 357, 357.2, 362-362.0, 790, 790.2
Unspecified Bronchitis and Bronchiectasis	2	490-490.9, 494-494.9
Primary or secondary Liver Cancer Unspecified	3	155, 155.2
Eye Unspecified Site Cancer	3	190, 190.9
Adrenal Unspecified Site Cancer	4	194-194.0, 194.9
Chronic lymphocytic leukemia by age	4	204, 204.1
Lymphoid leukemia unspecified by age	4	204, 204.5-204.9
myeloid leukemia by age	4	205, 205.8-206, 206.2-206.9
Heart failure unspecified right or left	1	428, 428.6-428.9
Hepatitis Unspecified	3	070, 070.4-070.9
Hepatic Failure	1	570-570.9, 573, 573.1-573.3, 782, 782.4, 789, 789.1-789.2, 789.5
Acute kidney failure	1	584-584.9, 586-587.9
Unspecified Eating Disorders	4	307, 307.5
Non-rheumatic valvar unspecified	1	424, 424.4-424.5, 424.9
hepatitis B unspecified	4	070, 070.3
Tobacco use disorder	2	305, 305.1
Peritonitis & Acute Abdomen	1	567-568.9, 789-789.0, 789.3-789.4, 789.6-789.9
Pneumothorax	1	423-423.0, 512-513, 513.9
Pleurisy, Pyothorax	1	510-511.9, 518, 518.3
upper and lower limb cancer	3	195, 195.4-195.5
Fluid, Electrolyte, Acid Base Disorders	1	276-276.9
Plegia	1	342-342.9, 344-344.9
Shock, Cardiac Arrest, Coma	1	427, 427.5, 427.9, 458-458.9, 780-780.0, 780.2-780.3, 785, 785.5-785.9, 799-799.1
Cardiac rhythm disorders	3	426-427, 427.4
Non-follicular lymphoma, unspecified	3	202, 202.9

Appendix Figure 5

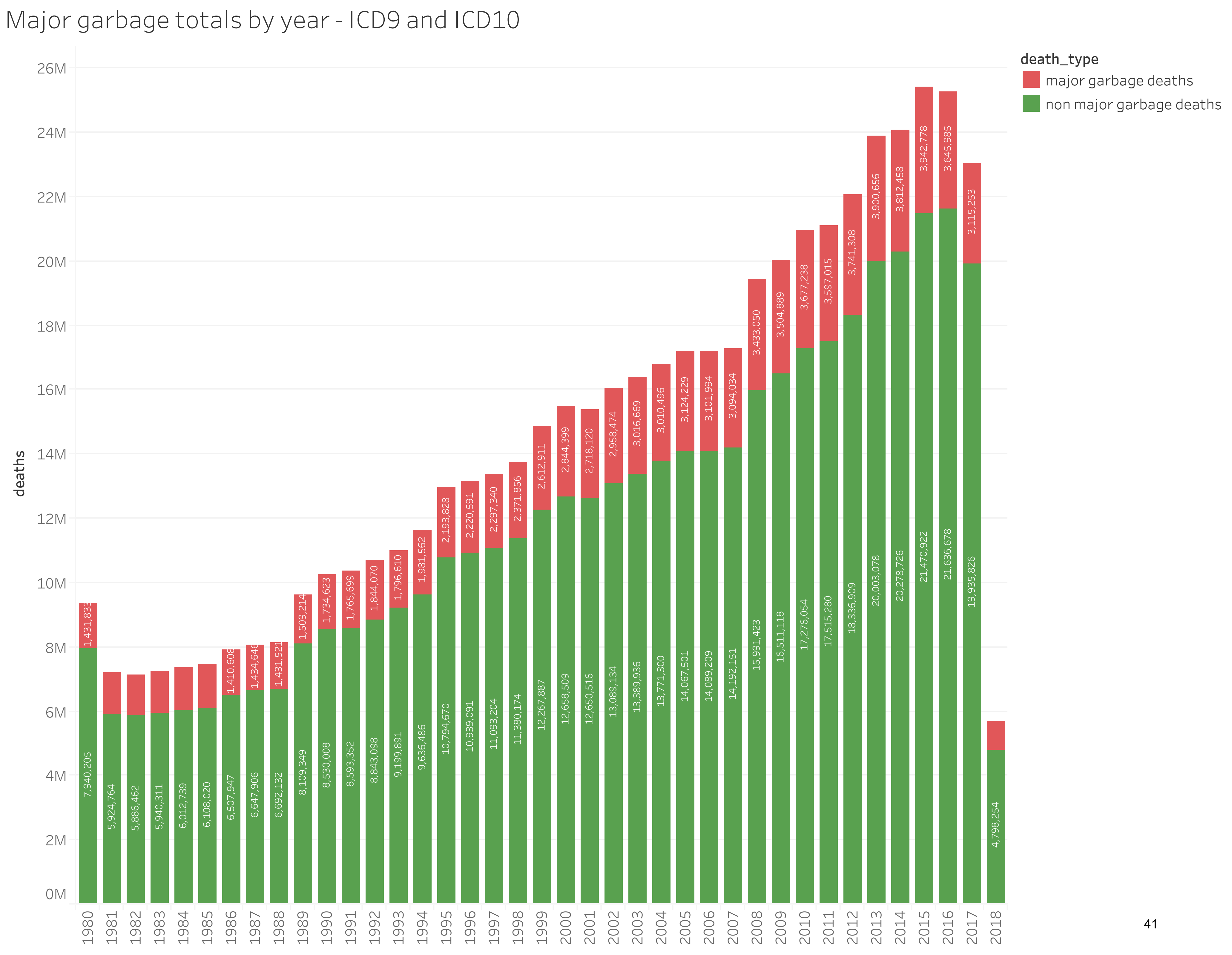

Appendix Figure 6

Percent Well Certified (PWC)	Star Rating
$PWC \geq 0.85$	5 Stars
$0.65 \leq PWC < 0.85$	4 Stars
$0.35 \leq PWC < 0.65$	3 Stars
$0.1 \leq PWC < 0.35$	2 Stars
$0 < PWC < 0.1$	1 Star
$PWC = 0$	0 Star

Appendix Figure 7

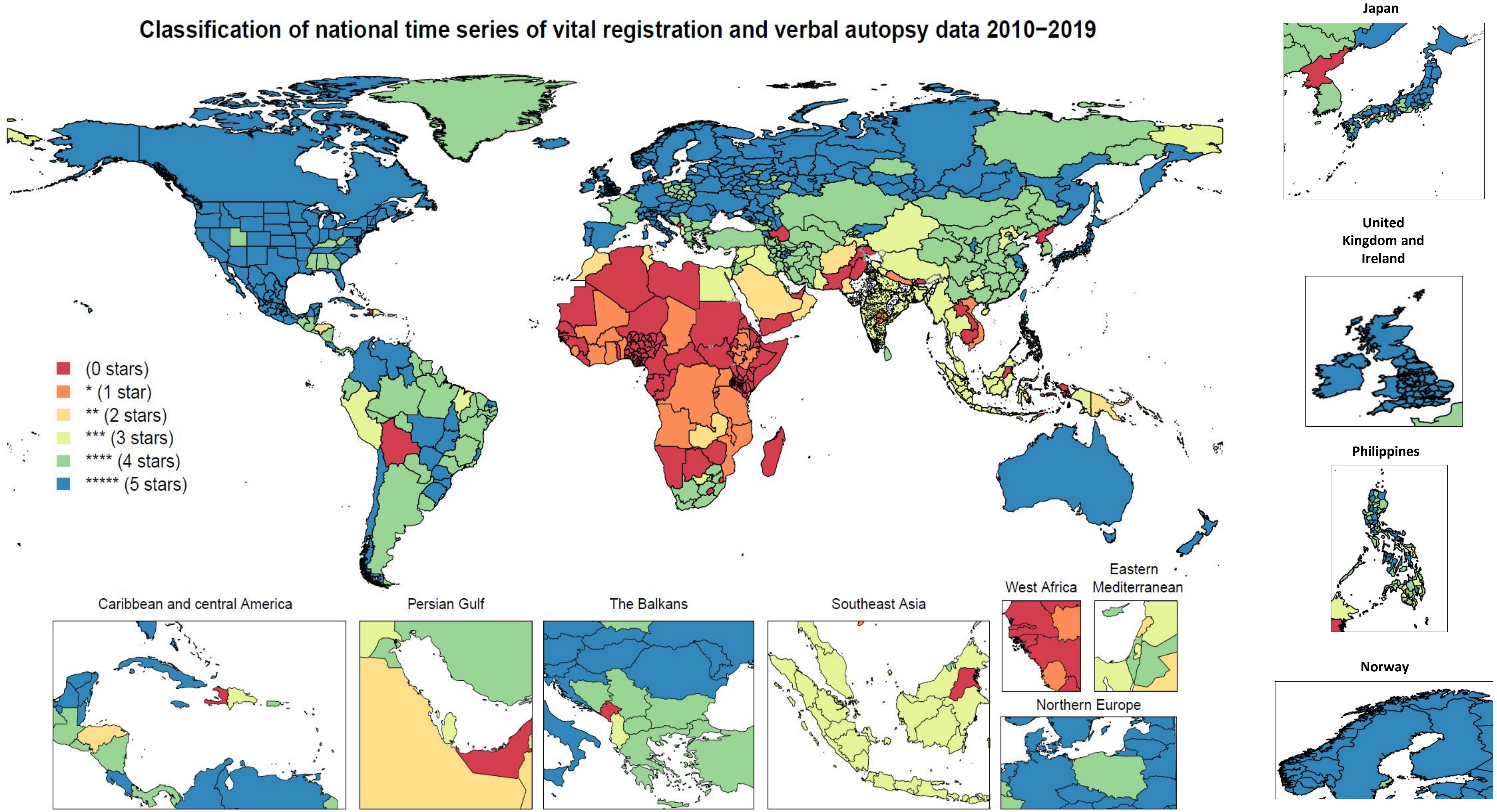

Appendix Figure 8

International Classification of Diseases (ICD) Mapped to Intermediate Causes		
**Intermediate cause**	**ICD 10**	**ICD 9**
Sepsis	A02.1-A02.9, A20.7-A20.9, A21.7-A21.9, A22.7-A22.9, A24.1-A24.9, A26.7-A26.9, A28.2-A28.9, A32.7-A32.9, A39.0, A39.4-A41.9, A42.7-A42.9, A50-A50.9, A54.86, B00.7-B00.9, B37.7-B37.9, B95-B95.8, N98.0, O03.0, O03.38, O03.5, O03.88, O04.5, O04.88, O07.38, O08.0, O08.83, O23-O23.93, O41.1-O41.93, O75.3, O85-O86.89, O88.3-O88.32, O91-O91.23, O98, O98.2-O98.93, P00.2, P22-P23.9, P29.12, P29.81, P35-P37, P37.1-P39.9, R65.2-R65.21, R68.13	038-038.9, 090-097.9, 286.6, 635-639.9, 646.5-646.64, 658.4-658.93, 659.2-659.33, 670-670.9, 672-672.04, 674.1-674.34, 675-675.94, 771, 771.4-771.89, 800-801.99, 803-804.99, 905.0, 995.9-995.94
Amyloidosis	E85.3-E85.9	277.3-277.39
Fluid, electrolyte, and acid base disorders	E86-E87.69, E87.8-E87.99	276.0-276.9
External causes UDI, type unspecified (Y34)	G44.3-G44.32, G91.3, R58, W47., W76-W76.9, Y10-Y14.9, Y16-Y34.9, Y85-Y87, Y87.2, Y89, Y89.9-Y99.9	E000-E80, E88, E980-E989
Cerebral palsy	G80-G80.9, G82.1, G82.4, G83.0, G83.8-G83.89	343-343.9
Plegia	G81-G82.0, G82.2-G82.3, G82.5-G83, G83.1-G83.5, G83.9	342-342.92, 344-344.9
Unspecified CNS signs and symptoms	G91-G91.2, G91.4-G92.6, G93, G93.1-G93.2, G93.40-G93.41, G93.5-G93.6, G93.9, G94.0-G94.8	293.0-293.9, 331.3-331.4, 332.1-332.9, 347-348.9, 349.81-349.9
Hypertension	I10-I10.9, I15-I15.9, I67.4, R03-R03.0, R04.0	401-401.9, 405-405.99, 784.7, 796.2
Pulmonary embolism	I26-I26.99	415-415.9
Right heart failure	I27, I27.2-I27.9	416, 416.2-416.9
Pneumothorax	I31.2-I31.4, J93-J93.12, J93.8-J93.9, J94.2, R04, R04.1-R04.9	423.0, 512-512.9, 513.9
Cardiac rhythm disorders	I44-I45.9, I49-I49.9	426-427, 427.4-427.42
Shock, cardiac arrest, and coma	I46-I46.9, I95-I95.1, I95.8-I95.9, R03.1, R09.0-R09.02, R09.2, R09.8, R40-R40.4, R55, R56, R56.00-R57.9	427.5, 427.9, 458-458.9, 780.0-780.09, 780.2-780.39, 785.5-785.9, 799.0-799.1
Unspecified heart failure	I50, I50.8-I50.9, J81, J81.1	428, 428.6-428.9
Left heart failure	I50.0-I50.43, I51.81	428.1-428.43
Atherosclerosis	I70-I70.1, I70.9-I70.92	440-440.1, 440.3-440.32, 440.8-440.9
Arterial embolism	I74-I75.89	444-445.89
Pneumonitis	J69-J69.9, J85-J85.3	507-507.9, 513-513.1
Acute respiratory failure	J80-J80.9, J81.0, J96-J96.02, J96.9-J96.92, J98.1-J98.3	514-514.9, 515.0-515.9, 518.0, 518.4-518.53
Pleurisy and pyothorax	J86-J86.9, J90-J90.0, J94-J94.1, J94.8-J94.9, R09.1	510-511.9, 518.3
Chronic respiratory failure	J96.1-J96.8	518, 518.8-518.89
Peritonitis and acute abdomen	K65-K66.1, K66.9, K68.1, K68.12-K68.9, R10-R10.9	567-568.9, 789.0-789.09, 789.3-789.49, 789.6-789.9
Alcoholic hepatic failure	K70.4-K70.9	
Hepatic failure	K71-K71.6, K71.8-K72.91, R16-R18.9	570-570.9, 571.41-571.49, 573.1-573.3, 782.4, 789.1-789.2, 789.5-789.59
Gastrointestinal bleeding	K92.0-K92.2	578-578.9
Osteomyelitis	M86-M86.29, M86.5-M86.9	730-730.09, 730.2-730.29, 730.7-730.99
Acute kidney failure	N17-N17.9	584, 584.5-584.9
Renal failure	N19-N19.9	586-587.9
Cachexia	R63.4, R63.6, R64	783.2-783.22, 799.4

Unspecified external factor (X59)	W47, W48, W63, W71-W72, W82, W95-W97, W98, X07, X55, X56, X59-X59.9	E887-E887.0, E928.9-E929.0, E929.8-E929.9

Appendix Figure 9

Covariates Used in Multiple Cause of Death Analysis	
**Intermediate Cause**	**Covariates**
Acute kidney failure	Healthcare access and quality index<br>Sex<br>Age group<br>Underlying cause
Acute respiratory failure	Healthcare access and quality index<br>Sex<br>Age group<br>Underlying cause
Alcoholic hepatic failure	Healthcare access and quality index<br>Alcohol binge drinker proportion, age-standardized<br>Sex<br>Age group<br>Underlying cause
Amyloidosis	Healthcare access and quality index<br>Sex<br>Age group<br>Underlying cause
Arterial embolism	Healthcare access and quality index<br>Sex<br>Age group<br>Underlying cause
Atherosclerosis**	Healthcare access and quality index<br>Mean BMI<br>Sex<br>Age group<br>Underlying cause
Cachexia	Healthcare access and quality index<br>Sex<br>Age group<br>Underlying cause
Cardiac rhythm disorders**	Healthcare access and quality index<br>Systolic Blood Pressure (mmHg)<br>Sex<br>Age group<br>Underlying cause
Cerebral palsy	Healthcare access and quality index<br>Sex<br>Age group<br>Underlying cause
Chronic respiratory failure	Healthcare access and quality index<br>Smoking Prevalence (Age-standardized, both sexes)<br>Sex<br>Age group<br>Underlying cause

External causes UDI, type unspecified (Y34)	Healthcare access and quality index<br>Sex<br>Age group<br>Underlying cause
Fluid, electrolyte, and acid base disorders	Healthcare access and quality index<br>Age- and sex-specific SEV for Unsafe sanitation<br>Sex<br>Age group<br>Underlying cause
Gastrointestinal bleeding	Healthcare access and quality index<br>Sex<br>Age group<br>Underlying cause
Hepatic failure	Healthcare access and quality index<br>Alcohol binge drinker proportion, age-standardized<br>Hepatitis B Seroprevalence (HBsAg) age standardized<br>Sex<br>Age group<br>Underlying cause
Hypertension**	Healthcare access and quality index<br>Mean BMI<br>Sex<br>Age group<br>Underlying cause
Left heart failure**	Healthcare access and quality index<br>Mean BMI<br>Smoking Prevalence (Age-standardized, both sexes)<br>Sex<br>Age group<br>Underlying cause
Osteomyelitis	Healthcare access and quality index<br>Sex<br>Age group<br>Underlying cause
Peritonitis and acute abdomen	Healthcare access and quality index<br>Sex<br>Age group<br>Underlying cause
Plegia	Healthcare access and quality index<br>Sex<br>Age group<br>Underlying cause
Pleurisy and pyothorax	Healthcare access and quality index<br>Sex<br>Age group<br>Underlying cause

Pneumonitis	Healthcare access and quality index<br>Sex<br>Age group<br>Underlying cause
Pneumothorax	Healthcare access and quality index<br>Sex<br>Age group<br>Underlying cause
Pulmonary embolism	Healthcare access and quality index<br>Sex<br>Age group<br>Underlying cause
Renal failure**	Healthcare access and quality index<br>Systolic Blood Pressure (mmHg)<br>Age- and sex-specific SEV for High fasting plasma glucose<br>Sex<br>Age group<br>Underlying cause
Right heart failure	Healthcare access and quality index<br>Smoking Prevalence (Age-standardized, both sexes)<br>Log-transformed age-standardized SEV scalar: TB<br>Sex<br>Age group<br>Underlying cause
Sepsis*	Healthcare access and quality index<br>Sex<br>Underlying cause
Shock, cardiac arrest, and coma**	Healthcare access and quality index<br>Sex<br>Age group<br>Underlying cause
Unspecified CNS signs and symptoms	Healthcare access and quality index<br>Sex<br>Age group<br>Underlying cause
Unspecified external factor (X59)	Healthcare access and quality index<br>Sex<br>Age group<br>Underlying cause
Unspecified heart failure**	Healthcare access and quality index<br>Systolic Blood Pressure (mmHg)<br>Sex<br>Age group<br>Underlying cause
***For sepsis, separate models were run for each age group**	

****For each of these intermediate causes, underlying cause was included as fixed effect. For all other intermediate causes, underlying cause was included as a random effect.**

Appendix Figure 10

Redistribution Proportions for Pulmonary Embolism, Year 2015, Top 20 Underlying Causes							
Age Group and Cause	Central Europe, Eastern Europe, and Central Asia	High-income	Latin America and Caribbean	North Africa and Middle East	South Asia	Southeast Asia, East Asia, and Oceania	Sub-Saharan Africa
**0 to 14**							
Lower respiratory infections	34.35%	4.75%	17.99%	12.20%	41.56%	22.17%	37.20%
Other cardiovascular and circulatory diseases	5.47%	14.22%	13.72%	50.35%	9.29%	10.76%	12.22%
Congenital heart anomalies	23.12%	22.38%	27.15%	15.30%	15.97%	26.15%	7.72%
Sickle cell disorders	0.04%	0.84%	2.81%	0.87%	1.74%	0.06%	15.79%
Falls	8.00%	4.76%	4.09%	4.01%	10.23%	11.34%	2.36%
Adverse effects of medical treatment	1.23%	2.58%	2.09%	1.01%	2.10%	1.35%	3.11%
Tuberculosis	0.64%	0.05%	0.38%	0.21%	2.32%	1.33%	3.01%
Endocrine, metabolic, blood, and immune disorders	3.71%	12.52%	4.46%	3.06%	1.65%	2.32%	0.79%
HIV/AIDS	0.51%	0.16%	1.12%	0.15%	0.33%	0.66%	3.69%
Brain and central nervous system cancer	3.74%	6.47%	2.44%	1.10%	1.67%	2.40%	0.59%
Pedestrian road injuries	1.34%	1.60%	1.67%	1.34%	1.01%	2.26%	1.12%
Paralytic ileus and intestinal obstruction	1.14%	1.55%	2.02%	0.62%	0.94%	1.90%	1.28%
Leukemia	1.64%	2.47%	2.08%	0.72%	0.84%	2.17%	0.65%
Other malignant neoplasms	1.79%	2.60%	1.23%	0.78%	1.26%	1.38%	0.63%
Motor vehicle road injuries	1.25%	2.09%	0.97%	1.46%	0.35%	0.80%	0.94%
Other exposure to mechanical forces	0.64%	0.63%	0.43%	0.70%	0.60%	1.24%	0.57%
Pulmonary aspiration and foreign body in airway	1.67%	2.66%	2.30%	0.32%	0.45%	1.47%	0.20%
Other cardiomyopathy	0.83%	1.97%	1.40%	0.73%	0.02%	0.58%	0.67%
Fire, heat, and hot substances	1.10%	0.84%	0.44%	0.36%	0.45%	0.37%	0.55%
Chronic kidney disease	0.35%	0.37%	0.59%	0.40%	0.47%	0.51%	0.42%
**15 to 29**							
Other cardiovascular and circulatory diseases	13.79%	18.45%	13.98%	22.82%	12.10%	8.23%	12.40%
Maternal disorders	1.36%	1.94%	7.19%	7.55%	17.14%	4.64%	20.16%
Falls	16.22%	9.53%	7.92%	9.80%	10.24%	16.01%	1.88%
HIV/AIDS	1.96%	0.48%	2.35%	0.38%	1.16%	1.66%	13.53%
Motor vehicle road injuries	7.31%	10.54%	4.80%	8.17%	2.01%	3.99%	3.41%
Tuberculosis	1.74%	0.11%	0.85%	0.72%	5.17%	3.09%	6.67%
Sickle cell disorders	0.05%	0.80%	1.75%	3.16%	1.83%	0.07%	11.07%
Motorcyclist road injuries	1.72%	4.21%	6.92%	2.69%	3.83%	6.88%	0.70%
Pedestrian road injuries	2.85%	2.18%	3.63%	3.93%	2.00%	5.41%	2.23%
Lower respiratory infections	3.81%	1.56%	2.94%	2.27%	2.00%	2.08%	4.23%
Ischemic heart disease	2.70%	1.19%	1.58%	3.35%	3.12%	3.15%	0.62%
Rheumatic heart disease	0.81%	0.23%	0.56%	0.69%	4.61%	1.10%	0.72%
Adverse effects of medical treatment	0.90%	1.43%	1.06%	2.09%	2.56%	0.93%	1.48%
Other malignant neoplasms	1.65%	2.43%	1.29%	1.67%	2.23%	1.81%	0.80%
Chronic kidney disease	1.12%	0.50%	1.76%	0.95%	1.56%	1.81%	1.33%
Brain and central nervous system cancer	2.82%	3.58%	1.76%	1.62%	1.01%	2.31%	0.47%
Congenital heart anomalies	1.41%	1.77%	1.42%	1.29%	1.27%	2.75%	0.51%
Other exposure to mechanical forces	1.18%	0.81%	0.72%	3.54%	0.94%	2.09%	0.64%
Leukemia	1.35%	1.98%	1.89%	1.39%	0.76%	2.35%	0.44%
Intracerebral hemorrhage	0.86%	0.50%	0.76%	1.78%	0.80%	2.22%	0.93%
**30 to 44**							
Other cardiovascular and circulatory diseases	15.63%	21.07%	14.56%	20.84%	13.97%	6.86%	11.78%
Falls	9.46%	5.85%	6.44%	8.22%	9.02%	11.32%	1.60%
HIV/AIDS	3.48%	0.73%	3.53%	0.74%	1.96%	2.01%	23.60%
Maternal disorders	0.42%	0.90%	3.68%	5.29%	7.64%	2.19%	15.38%
Ischemic heart disease	6.56%	4.13%	4.08%	8.95%	9.11%	6.82%	1.77%
Tuberculosis	1.31%	0.10%	0.84%	0.75%	7.04%	2.31%	7.89%
Breast cancer	2.85%	5.98%	3.81%	3.97%	3.47%	4.59%	2.11%
Intracerebral hemorrhage	2.01%	1.06%	1.54%	2.23%	2.08%	5.17%	1.91%
Lower respiratory infections	3.91%	1.46%	2.96%	1.92%	1.61%	1.50%	3.84%
Stomach cancer	2.12%	2.12%	2.49%	1.88%	1.90%	3.76%	0.76%
Tracheal, bronchus, and lung cancer	2.14%	3.60%	1.41%	1.71%	0.99%	4.70%	0.48%
Cervical cancer	1.81%	1.98%	3.52%	0.66%	1.81%	2.06%	2.70%
Colon and rectum cancer	1.96%	3.89%	2.11%	1.67%	1.07%	3.74%	0.66%
Motor vehicle road injuries	2.19%	2.49%	2.32%	5.51%	1.17%	1.74%	1.57%
Cirrhosis and other chronic liver diseases	3.28%	1.35%	1.79%	0.83%	2.42%	1.81%	1.36%
Pedestrian road injuries	1.18%	0.80%	2.21%	2.53%	1.39%	3.16%	1.04%
Rheumatic heart disease	0.59%	0.24%	0.58%	0.71%	4.65%	0.95%	0.36%
Chronic kidney disease	0.65%	0.81%	2.28%	1.36%	2.08%	1.92%	1.11%
Motorcyclist road injuries	0.37%	1.09%	2.50%	1.10%	2.18%	2.23%	0.27%
Brain and central nervous system cancer	2.16%	3.32%	1.80%	1.88%	0.94%	2.03%	0.42%
**45 to 59**							

Other cardiovascular and circulatory diseases	14.91%	14.48%	13.58%	22.69%	14.21%	5.68%	16.45%
Ischemic heart disease	12.81%	6.39%	7.54%	16.78%	14.46%	8.57%	4.98%
Tracheal, bronchus, and lung cancer	7.84%	11.53%	4.13%	4.93%	2.29%	10.30%	1.98%
Falls	4.87%	4.10%	4.63%	3.00%	7.00%	6.50%	2.41%
Intracerebral hemorrhage	3.26%	1.32%	2.62%	2.72%	4.73%	7.84%	4.80%
Breast cancer	3.74%	5.77%	4.30%	4.05%	4.11%	4.06%	3.41%
Stomach cancer	3.28%	2.43%	3.41%	2.44%	2.33%	5.95%	1.56%
Colon and rectum cancer	3.85%	5.70%	3.23%	2.46%	1.80%	4.60%	1.48%
Chronic obstructive pulmonary disease	1.39%	2.25%	1.98%	2.02%	5.73%	2.96%	1.73%
Tuberculosis	0.78%	0.07%	0.50%	0.43%	5.24%	1.34%	7.32%
Diabetes mellitus type 2	0.83%	1.23%	4.08%	2.40%	2.36%	2.54%	2.70%
Pancreatic cancer	2.83%	4.61%	2.21%	1.79%	0.87%	2.43%	0.94%
Lower respiratory infections	2.20%	1.26%	2.76%	1.63%	1.93%	1.27%	5.51%
Cervical cancer	1.34%	1.08%	2.73%	0.66%	1.77%	1.99%	3.50%
Chronic kidney disease	0.53%	0.91%	3.65%	1.98%	2.38%	1.73%	1.48%
Ischemic stroke	2.80%	0.43%	0.85%	3.14%	1.09%	2.86%	0.86%
Ovarian cancer	2.33%	2.73%	1.93%	1.22%	1.39%	1.69%	0.98%
Cirrhosis and other chronic liver diseases	2.15%	1.65%	2.02%	1.32%	1.73%	1.43%	1.81%
HIV/AIDS	0.29%	0.26%	1.07%	0.19%	0.46%	0.47%	10.91%
Esophageal cancer	0.89%	1.29%	1.03%	0.54%	1.08%	2.44%	1.39%
**60 to 69**							
Other cardiovascular and circulatory diseases	14.77%	12.61%	13.64%	23.30%	13.97%	5.62%	19.58%
Ischemic heart disease	16.85%	6.52%	8.97%	19.04%	12.94%	9.25%	7.20%
Tracheal, bronchus, and lung cancer	9.40%	14.17%	5.25%	5.37%	2.29%	12.65%	2.60%
Chronic obstructive pulmonary disease	2.36%	4.13%	3.78%	3.30%	12.09%	6.65%	3.51%
Intracerebral hemorrhage	3.03%	1.32%	2.69%	2.31%	5.37%	8.17%	6.00%
Falls	2.76%	3.40%	3.79%	1.85%	9.26%	4.07%	2.82%
Ischemic stroke	6.33%	1.02%	2.26%	5.46%	2.94%	5.96%	2.74%
Stomach cancer	3.43%	2.61%	3.61%	2.48%	1.87%	6.89%	1.84%
Colon and rectum cancer	5.07%	6.02%	3.41%	2.34%	1.58%	4.35%	1.76%
Pancreatic cancer	3.33%	5.75%	2.68%	1.90%	0.98%	2.60%	1.26%
Diabetes mellitus type 2	1.25%	1.33%	5.09%	3.10%	2.87%	2.51%	4.08%
Lower respiratory infections	1.34%	1.50%	2.98%	1.87%	3.10%	1.53%	6.90%
Breast cancer	2.80%	3.78%	2.53%	1.84%	1.95%	2.03%	2.31%
Hypertensive heart disease	1.19%	0.77%	1.44%	3.15%	1.32%	2.17%	3.11%
Chronic kidney disease	0.53%	1.12%	4.02%	2.47%	1.88%	1.58%	1.72%
Esophageal cancer	0.81%	1.42%	0.92%	0.49%	0.79%	3.19%	1.48%
Tuberculosis	0.23%	0.05%	0.31%	0.30%	3.22%	0.97%	4.69%
Ovarian cancer	1.89%	2.32%	1.39%	0.83%	0.96%	1.06%	0.83%
Prostate cancer	1.46%	1.93%	2.34%	0.82%	0.59%	0.81%	2.45%
Liver cancer	0.62%	1.54%	0.68%	1.31%	0.46%	1.94%	0.64%
**70 to 79**							
Other cardiovascular and circulatory diseases	14.68%	13.83%	14.96%	27.01%	14.86%	6.37%	22.35%
Ischemic heart disease	23.45%	6.95%	9.43%	18.45%	11.58%	10.83%	8.22%
Chronic obstructive pulmonary disease	2.96%	5.36%	5.61%	4.11%	16.16%	11.89%	4.51%
Ischemic stroke	12.37%	2.54%	4.66%	8.01%	4.63%	9.85%	4.87%
Tracheal, bronchus, and lung cancer	5.24%	11.13%	4.24%	3.51%	1.72%	9.57%	2.03%
Falls	2.20%	4.54%	4.53%	2.13%	12.66%	4.36%	3.77%
Intracerebral hemorrhage	2.87%	1.47%	2.20%	1.98%	3.86%	8.04%	5.51%
Colon and rectum cancer	4.76%	5.51%	3.07%	2.00%	1.40%	3.54%	1.58%
Stomach cancer	2.74%	2.56%	3.31%	2.16%	1.29%	5.33%	1.58%
Lower respiratory infections	1.12%	2.34%	4.13%	2.21%	3.83%	2.27%	8.00%
Diabetes mellitus type 2	1.23%	1.37%	4.90%	2.65%	2.62%	2.11%	3.70%
Pancreatic cancer	2.56%	4.96%	2.27%	1.41%	0.83%	1.85%	1.05%
Hypertensive heart disease	1.83%	0.87%	1.88%	4.06%	1.66%	3.08%	3.56%
Prostate cancer	1.86%	2.89%	3.74%	1.40%	0.89%	1.17%	3.47%
Chronic kidney disease	0.56%	1.42%	3.40%	2.56%	1.47%	1.50%	1.94%
Breast cancer	1.93%	2.60%	1.53%	0.98%	1.12%	1.05%	1.48%
Esophageal cancer	0.43%	0.98%	0.63%	0.38%	0.54%	2.35%	0.94%
Tuberculosis	0.08%	0.06%	0.23%	0.27%	2.33%	0.77%	3.85%
Other malignant neoplasms	0.95%	1.15%	0.90%	0.78%	1.05%	0.79%	0.68%
Ovarian cancer	1.19%	1.70%	0.89%	0.49%	0.60%	0.53%	0.51%
**80 plus**							
Other cardiovascular and circulatory diseases	14.34%	16.67%	17.99%	29.99%	16.28%	7.23%	22.70%
Ischemic heart disease	29.98%	9.84%	10.98%	19.07%	11.39%	14.43%	9.09%
Chronic obstructive pulmonary disease	2.83%	5.07%	7.02%	4.47%	18.37%	15.56%	4.27%
Ischemic stroke	17.45%	5.97%	6.70%	9.97%	5.52%	12.04%	7.37%
Falls	3.33%	8.84%	8.13%	3.77%	15.72%	8.16%	6.23%
Lower respiratory infections	1.50%	4.62%	6.75%	2.90%	4.33%	3.70%	9.68%
Tracheal, bronchus, and lung cancer	1.86%	4.45%	2.03%	1.58%	0.85%	4.89%	1.01%
Hypertensive heart disease	2.72%	2.11%	2.68%	5.10%	2.06%	4.25%	4.24%
Intracerebral hemorrhage	2.02%	1.26%	1.25%	1.45%	2.16%	6.60%	3.75%
Colon and rectum cancer	3.08%	4.08%	2.22%	1.46%	1.13%	2.35%	1.32%

Stomach cancer	1.40%	1.77%	2.12%	1.41%	0.88%	2.94%	1.14%
Prostate cancer	1.29%	2.38%	3.50%	1.42%	0.96%	1.03%	3.08%
Diabetes mellitus type 2	0.83%	1.28%	3.50%	1.92%	2.69%	1.44%	3.34%
Chronic kidney disease	0.59%	2.09%	2.76%	2.44%	1.20%	1.33%	2.17%
Pancreatic cancer	1.33%	2.52%	1.29%	0.79%	0.52%	0.98%	0.64%
Breast cancer	1.23%	1.75%	0.97%	0.54%	0.61%	0.54%	1.22%
Other cardiomyopathy	2.77%	1.52%	1.30%	0.40%	0.06%	0.39%	1.20%
Bladder cancer	0.74%	1.30%	0.58%	0.68%	0.36%	0.56%	0.51%
Endocrine, metabolic, blood, and immune disorders	0.14%	1.35%	1.23%	0.71%	0.16%	0.47%	0.46%
Lower extremity peripheral arterial disease	1.28%	1.22%	0.64%	0.20%	0.13%	0.08%	0.42%

Proportions in the above table are unique by super region and age group. For each individual percentage in the table, the denominator used in its calculation was the total deaths redistributed across all causes within its respective super region and age group. Thus, column percentages within each age group will add to, or very close to, 100%. Only the top 20 redistribution targets are listed, so it is possible that some columns may not quite total 100%.

Appendix Figure 11

Redistribution Proportions for Unspecified Heart Failure, Year 2015, Top 20 Underlying Causes							
Age Group and Cause	Central Europe, Eastern Europe, and Central Asia	High-income	Latin America and Caribbean	North Africa and Middle East	South Asia	Southeast Asia, East Asia, and Oceania	Sub-Saharan Africa
**0 to 14**							
Congenital heart anomalies	76.34%	60.17%	68.01%	65.80%	57.18%	73.30%	40.16%
Protein-energy malnutrition	0.13%	0.23%	3.19%	1.52%	6.10%	1.33%	14.89%
Neonatal preterm birth	2.63%	3.55%	2.65%	3.47%	6.42%	3.03%	4.57%
Other cardiomyopathy	4.60%	9.29%	5.86%	5.16%	0.13%	2.81%	5.75%
Sickle cell disorders	0.02%	0.27%	0.84%	0.45%	0.77%	0.02%	9.79%
Rheumatic heart disease	0.98%	0.53%	1.21%	2.32%	5.88%	2.58%	2.61%
Chronic kidney disease	1.95%	1.67%	2.48%	2.86%	2.88%	2.44%	3.56%
Down syndrome	1.48%	3.80%	3.22%	3.03%	1.19%	1.83%	2.78%
Neonatal encephalopathy due to birth asphyxia and trauma	1.17%	0.54%	0.71%	0.33%	2.67%	0.90%	2.68%
Endocrine, metabolic, blood, and immune disorders	2.49%	7.00%	2.37%	2.74%	1.29%	1.39%	0.86%
Thalassemias	1.80%	0.83%	0.17%	0.98%	2.83%	2.26%	0.46%
Other neonatal disorders	0.58%	0.68%	0.54%	1.00%	2.91%	0.34%	1.01%
Chronic kidney disease due to other and unspecified causes	0.75%	0.66%	0.81%	1.30%	1.04%	0.94%	1.04%
Pulmonary Arterial Hypertension	1.05%	4.73%	1.80%	0.94%	0.91%	0.95%	0.73%
Chronic kidney disease due to glomerulonephritis	0.50%	0.39%	0.88%	0.41%	0.87%	0.52%	1.51%
Cirrhosis and other chronic liver diseases	0.74%	0.22%	0.46%	0.77%	1.27%	0.54%	1.10%
Other cardiovascular and circulatory diseases	0.26%	0.56%	0.50%	3.07%	0.49%	0.45%	0.91%
Endocarditis	0.33%	0.72%	1.13%	0.67%	0.44%	0.67%	1.33%
Neonatal sepsis and other neonatal infections	0.32%	0.24%	0.63%	0.16%	0.69%	0.41%	1.12%
Diabetes mellitus type 1	0.43%	0.45%	0.50%	0.44%	0.69%	0.49%	0.85%
**15 to 29**							
Ischemic heart disease	17.27%	11.89%	14.18%	27.81%	18.46%	22.26%	5.94%
Rheumatic heart disease	5.91%	2.50%	5.19%	6.14%	29.15%	8.73%	7.22%
Maternal disorders	1.11%	2.50%	8.24%	8.14%	13.14%	4.18%	24.24%
Chronic kidney disease	6.68%	4.48%	14.41%	7.06%	8.40%	11.75%	11.63%
Congenital heart anomalies	4.66%	9.26%	6.67%	5.62%	3.94%	10.15%	2.56%
Cirrhosis and other chronic liver diseases	6.56%	2.03%	3.14%	2.68%	6.28%	4.09%	5.25%
Other cardiomyopathy	19.37%	17.22%	7.10%	4.26%	0.29%	3.39%	5.20%
Chronic kidney disease due to glomerulonephritis	2.16%	1.56%	6.14%	1.50%	2.73%	2.31%	5.93%
Hypertensive heart disease	1.18%	4.66%	2.37%	5.30%	1.06%	3.28%	3.70%
Chronic kidney disease due to other and unspecified causes	2.02%	1.18%	2.94%	2.22%	2.40%	1.64%	1.74%
Sickle cell disorders	0.02%	0.55%	1.04%	1.82%	0.75%	0.03%	7.22%
Motor vehicle road injuries	2.40%	5.54%	2.31%	3.75%	0.68%	1.52%	1.77%
Endocarditis	2.86%	3.28%	2.53%	1.21%	0.93%	2.55%	1.46%
Diabetes mellitus type 1	2.28%	3.35%	2.49%	1.41%	1.30%	1.76%	1.50%
Chronic obstructive pulmonary disease	1.23%	1.33%	1.72%	2.43%	0.97%	2.24%	1.33%
Pulmonary Arterial Hypertension	1.29%	3.67%	1.35%	1.18%	1.02%	1.27%	1.19%
Chronic kidney disease due to hypertension	0.23%	0.40%	0.84%	0.78%	0.73%	2.32%	1.59%
Intracerebral hemorrhage	0.71%	0.65%	0.90%	1.99%	0.65%	2.09%	1.20%
Other cardiovascular and circulatory diseases	0.87%	1.82%	1.27%	1.96%	0.75%	0.60%	1.24%
Endocrine, metabolic, blood, and immune disorders	0.88%	6.97%	2.19%	1.33%	0.41%	0.96%	0.51%
**30 to 44**							
Ischemic heart disease	27.87%	28.40%	24.76%	47.24%	38.22%	35.71%	16.15%
Rheumatic heart disease	2.88%	1.82%	3.75%	4.06%	20.68%	5.62%	3.49%
Chronic kidney disease	2.53%	5.10%	12.65%	6.39%	7.84%	9.19%	8.99%
Cirrhosis and other chronic liver diseases	8.33%	5.62%	6.96%	2.72%	6.51%	6.01%	7.86%
Hypertensive heart disease	2.39%	10.37%	5.10%	8.87%	3.02%	7.59%	11.32%
Maternal disorders	0.23%	0.80%	2.88%	3.54%	4.07%	1.46%	17.66%
Other cardiomyopathy	17.28%	10.74%	6.50%	2.24%	0.19%	2.41%	5.64%
Alcoholic cardiomyopathy	24.85%	2.20%	1.06%	0.15%	0.14%	0.60%	0.13%
Chronic obstructive pulmonary disease	1.08%	1.57%	1.81%	2.24%	2.15%	2.68%	1.79%
Diabetes mellitus type 2	0.31%	1.61%	4.37%	1.51%	1.37%	2.97%	2.42%
Intracerebral hemorrhage	1.09%	0.93%	1.22%	1.53%	1.16%	3.55%	2.30%
Chronic kidney disease due to glomerulonephritis	0.75%	1.41%	4.04%	0.83%	2.06%	1.01%	3.02%
Chagas disease			1.82%				
Chronic kidney disease due to diabetes mellitus type 1	0.31%	0.66%	2.26%	1.25%	0.94%	2.96%	1.48%
Chronic kidney disease due to hypertension	0.19%	0.84%	1.38%	1.24%	1.17%	2.25%	2.22%
Diabetes mellitus type 1	0.88%	2.35%	1.55%	0.91%	0.95%	1.52%	1.52%
Congenital heart anomalies	0.80%	2.35%	1.45%	1.01%	1.09%	1.70%	0.77%
Endocarditis	1.43%	2.41%	1.62%	0.84%	0.72%	1.49%	1.22%

Chronic kidney disease due to other and unspecified causes	0.54%	0.83%	1.84%	1.16%	1.69%	0.65%	0.80%
Tracheal, bronchus, and lung cancer	0.75%	2.02%	0.73%	0.76%	0.37%	2.08%	0.38%
**45 to 59**							
Ischemic heart disease	48.15%	37.30%	30.83%	55.57%	47.74%	38.27%	27.46%
Hypertensive heart disease	5.05%	9.25%	6.68%	11.59%	4.97%	11.17%	18.41%
Chronic kidney disease	1.81%	4.89%	13.78%	5.82%	7.04%	7.11%	7.33%
Rheumatic heart disease	2.37%	1.25%	1.79%	1.74%	11.22%	3.89%	2.33%
Chronic obstructive pulmonary disease	2.01%	5.03%	3.11%	2.62%	7.62%	5.24%	3.79%
Cirrhosis and other chronic liver diseases	4.86%	5.89%	5.34%	2.74%	3.70%	4.09%	6.52%
Diabetes mellitus type 2	1.40%	3.23%	7.58%	3.48%	3.46%	5.02%	6.73%
Other cardiomyopathy	8.33%	5.25%	4.66%	1.36%	0.11%	1.52%	5.63%
Intracerebral hemorrhage	1.59%	1.00%	1.42%	1.18%	2.10%	4.63%	3.58%
Tracheal, bronchus, and lung cancer	2.49%	5.60%	1.46%	1.44%	0.68%	4.00%	0.97%
Chronic kidney disease due to hypertension	0.25%	1.22%	2.78%	1.71%	1.53%	2.58%	2.57%
Chagas disease			1.83%				
Alcoholic cardiomyopathy	13.35%	2.40%	0.76%	0.12%	0.13%	0.36%	0.24%
Ischemic stroke	2.13%	0.51%	0.72%	2.13%	0.76%	2.62%	0.97%
Chronic kidney disease due to diabetes mellitus type 2	0.25%	0.95%	2.08%	1.23%	1.19%	1.55%	1.13%
Colon and rectum cancer	1.13%	2.65%	1.09%	0.66%	0.49%	1.69%	0.68%
Chronic kidney disease due to glomerulonephritis	0.44%	0.83%	3.48%	0.45%	1.04%	0.53%	1.55%
Chronic kidney disease due to diabetes mellitus type 1	0.16%	0.33%	1.40%	0.51%	0.81%	1.13%	0.78%
Chronic kidney disease due to other and unspecified causes	0.31%	0.63%	1.64%	0.79%	1.05%	0.39%	0.50%
Other cardiovascular and circulatory diseases	0.63%	0.94%	0.65%	0.89%	0.57%	0.29%	1.11%
**60 to 69**							
Ischemic heart disease	59.06%	37.01%	32.20%	53.76%	43.20%	36.41%	30.61%
Chronic obstructive pulmonary disease	3.27%	9.12%	5.33%	3.75%	16.58%	10.60%	6.07%
Hypertensive heart disease	6.31%	6.56%	7.39%	12.90%	6.06%	12.59%	18.40%
Chronic kidney disease	1.67%	5.87%	13.40%	6.28%	5.73%	5.78%	6.78%
Diabetes mellitus type 2	2.00%	3.46%	8.47%	3.94%	4.41%	4.52%	7.96%
Rheumatic heart disease	1.49%	1.34%	1.03%	0.99%	7.12%	3.50%	2.17%
Ischemic stroke	4.65%	1.20%	1.76%	3.28%	2.16%	5.00%	2.51%
Tracheal, bronchus, and lung cancer	2.93%	6.98%	1.71%	1.40%	0.73%	4.54%	1.03%
Intracerebral hemorrhage	1.44%	1.00%	1.34%	0.89%	2.54%	4.45%	3.65%
Cirrhosis and other chronic liver diseases	2.47%	3.24%	3.30%	2.43%	2.20%	2.14%	4.95%
Chronic kidney disease due to hypertension	0.36%	1.88%	3.85%	2.35%	1.62%	2.57%	2.93%
Other cardiomyopathy	3.73%	4.17%	3.67%	1.07%	0.12%	1.02%	4.17%
Chagas disease			1.69%				
Chronic kidney disease due to diabetes mellitus type 2	0.27%	1.37%	2.53%	1.57%	1.29%	1.45%	1.34%
Colon and rectum cancer	1.46%	2.80%	1.05%	0.56%	0.47%	1.47%	0.65%
Alcoholic cardiomyopathy	4.76%	1.57%	0.38%	0.08%	0.10%	0.13%	0.11%
Chronic kidney disease due to glomerulonephritis	0.35%	0.65%	2.69%	0.33%	0.70%	0.35%	1.03%
Chronic kidney disease due to other and unspecified causes	0.27%	0.72%	1.55%	0.77%	0.72%	0.34%	0.46%
Non-rheumatic calcific aortic valve disease	0.56%	2.06%	1.01%	0.49%	0.35%	0.25%	0.38%
Other cardiovascular and circulatory diseases	0.59%	0.79%	0.58%	0.79%	0.57%	0.26%	1.02%
**70 to 79**							
Ischemic heart disease	65.77%	35.58%	33.38%	50.68%	39.42%	35.59%	32.41%
Chronic obstructive pulmonary disease	3.40%	10.99%	8.01%	4.64%	23.07%	16.15%	7.46%
Hypertensive heart disease	7.34%	6.19%	8.96%	15.23%	7.30%	14.06%	18.39%
Chronic kidney disease	1.44%	6.74%	11.27%	6.45%	4.61%	4.60%	7.24%
Ischemic stroke	7.53%	2.81%	3.69%	4.91%	3.62%	7.19%	4.33%
Diabetes mellitus type 2	1.61%	3.28%	8.24%	3.39%	4.29%	3.26%	6.99%
Rheumatic heart disease	0.74%	1.67%	0.70%	0.72%	5.28%	2.93%	1.50%
Intracerebral hemorrhage	1.15%	1.06%	1.14%	0.79%	1.99%	3.86%	3.29%
Tracheal, bronchus, and lung cancer	1.39%	5.28%	1.44%	0.94%	0.60%	3.02%	0.80%
Chronic kidney disease due to hypertension	0.41%	2.48%	3.88%	2.74%	1.47%	2.17%	3.41%
Other cardiomyopathy	2.62%	4.00%	3.32%	0.82%	0.16%	0.74%	2.93%
Cirrhosis and other chronic liver diseases	0.85%	1.57%	2.12%	2.22%	1.40%	1.16%	2.99%
Chagas disease			1.29%				
Chronic kidney disease due to diabetes mellitus type 2	0.24%	1.51%	2.25%	1.54%	1.03%	1.14%	1.51%
Colon and rectum cancer	1.16%	2.44%	0.98%	0.49%	0.44%	1.05%	0.57%
Non-rheumatic calcific aortic valve disease	0.61%	3.50%	0.99%	0.45%	0.42%	0.13%	0.42%
Chronic kidney disease due to other and unspecified causes	0.24%	0.89%	1.40%	0.82%	0.62%	0.35%	0.54%
Other cardiovascular and circulatory diseases	0.47%	0.79%	0.62%	0.89%	0.62%	0.24%	1.09%
Interstitial lung disease and pulmonary sarcoidosis	0.09%	1.37%	0.67%	0.18%	1.08%	0.13%	0.21%
Chronic kidney disease due to glomerulonephritis	0.24%	0.59%	1.78%	0.29%	0.59%	0.27%	0.91%
**80 plus**							
Ischemic heart disease	67.43%	37.32%	37.65%	50.61%	38.34%	39.75%	33.49%

Chronic obstructive pulmonary disease	2.68%	7.97%	10.07%	5.00%	26.45%	18.15%	6.83%
Hypertensive heart disease	8.10%	10.17%	11.30%	17.02%	8.20%	15.01%	18.78%
Ischemic stroke	9.06%	5.22%	5.49%	6.28%	4.60%	7.87%	6.60%
Chronic kidney disease	1.24%	7.38%	8.99%	6.05%	3.81%	3.50%	7.60%
Diabetes mellitus type 2	0.91%	2.36%	5.94%	2.48%	4.58%	1.97%	6.19%
Chronic kidney disease due to hypertension	0.48%	3.54%	3.94%	2.89%	1.46%	1.77%	4.03%
Other cardiomyopathy	4.41%	3.98%	2.90%	0.73%	0.13%	0.72%	2.89%
Non-rheumatic calcific aortic valve disease	0.64%	6.11%	0.78%	0.39%	0.48%	0.09%	0.50%
Rheumatic heart disease	0.36%	1.68%	0.38%	0.47%	4.06%	2.26%	1.00%
Intracerebral hemorrhage	0.70%	0.73%	0.68%	0.60%	1.21%	2.88%	2.30%
Tracheal, bronchus, and lung cancer	0.43%	1.70%	0.72%	0.44%	0.31%	1.39%	0.40%
Chronic kidney disease due to diabetes mellitus type 2	0.17%	1.26%	1.80%	1.22%	0.72%	0.84%	1.47%
Colon and rectum cancer	0.65%	1.45%	0.74%	0.37%	0.38%	0.63%	0.48%
Cirrhosis and other chronic liver diseases	0.27%	0.60%	1.13%	1.71%	0.98%	0.62%	1.88%
Chagas disease			0.75%				
Chronic kidney disease due to other and unspecified causes	0.22%	1.12%	1.13%	0.89%	0.57%	0.31%	0.68%
Other cardiovascular and circulatory diseases	0.37%	0.69%	0.71%	0.93%	0.66%	0.23%	1.01%
Endocarditis	0.07%	1.21%	0.25%	0.19%	0.20%	0.17%	0.21%
Interstitial lung disease and pulmonary sarcoidosis	0.05%	0.75%	0.70%	0.14%	0.90%	0.08%	0.16%

Proportions in the above table are unique by super region and age group. For each individual percentage in the table, the denominator used in its calculation was the total deaths redistributed across all causes within its respective super region and age group. Thus, column percentages within each age group will add to, or very close to, 100%. Only the top 20 redistribution targets are listed, so it is possible that some columns may not quite total 100%.

Appendix Figure 12

Custom N-Code Groups for X59/Y34 Redistribution by ICD Classification System		
	ICD Classification System	
**Group Name**	**ICD9**	**ICD10**
Group 1	896.0, 885.1, 886, 886.0, 886.1, 887, 887.0, 887.1, 887.2, 887.3, 885.0, 896.1, 896, 895.1, 895.0, 895, 888.9, 888.2, 888.1, 888, 887.7, 887.4, 887.6, 885, 896.3, 896.2, 897.7, 897.6, 897.5, 897.4, 887.5, 897.2, 897.1, 897.0, 897, 897.3	T05.6, S68.4, S68.5, S68.6, S68.7, S68.8, S68.9, S58.1, S58.0, S68.3, S58, S78.0, S78.1, S78.9, S48, S48.0, S48.1, S48.9, T05.5, S78, S68.2, S58.9, S68.0, T05.2, T05.3, S68.1, T05.4, T05.0, S98.9, S98.4, T11.6, S98.2, S98.1, S98.3, S98, S68, S88.9, S88.1, S88.0, S88, S98.0, T05.1
Group 2	944.4, 944.2, 944.1, 944.0, 944, 943.5, 943.4, 943.3, 943.2, 943.1, 944.3, 949.5, 944.5, 946.0, 946, 945.5, 945.4, 949.4, 945.3, 945.2, 945.1, 945.0, 946.2, 946.3, 946.4, 946.5, 947.0, 947.4, 947, 947.1, 947.2, 947.3, 947.8, 945, 943.0, 946.1, 943, 942.4, 949.0, 948.1, 948.2, 948.3, 949, 948.9, 906.5, 948.4, 948.5, 948.6, 948.7, 948.8, 906.9, 906.8, 942.5, 949.1, 906.6, 949.2, 941.2, 942.3, 942.2, 942.1, 942.0, 942, 941.5, 941.4, 941.3, 906.7, 941.1, 941.0, 941, 947.9, 948, 948.0, 949.3	T23.7, T23.6, T24, T24.1, T24.2, T24.3, T23.5, T25.2, T24.5, T24.6, T24.7, T25, T25.0, T25.1, T25.3, T25.4, T25.5, T23.4, T25.6, T25.7, T24.4, T23.3, T22, T23.1, T20, T20.0, T20.1, T20.2, T20.3, T20.4, T20.5, T20.6, T20.7, T21, T21.0, T21.1, T21.2, T21.3, T23.2, T21.4, T21.6, T21.7, T21.9, T27, T22.0, T22.1, T22.2, T22.3, T22.4, T22.5, T22.6, T22.7, T23, T23.0, T21.5, T27.0, T24.0, T27.2, T29, T29.0, T29.1, T95.0, T30.1, T32.7, T32.6, T32.5, T32.4, T32.3, T32.2, T31.4, T31.3, T31.2, T31.1, T31.0, T30.7, T30.2, T30.3, T30.4, T30.0, T30, T29.7, T28.9, T29.6, T29.4, T29.3, T29.2, T27.1, T30.5, T30.6, T29.5, T28.8, T95, T28.6, T95.2, T95.3, T95.4, T32.8, T27.5, T32.9, T28.7, T27.6, T27.7, T27.4, T28, T28.0, T31.7, T95.1, T32.0, T28.5, T28.4, T31.5, T28.3, T31.8, T27.3, T31.9, T31.6, T32.1, T28.1, T28.2
Group 3	835, 835.0, 835.1	S73.0, S73
Group 4	836.6, 836, 836.0, 836.1, 836.2, 836.3, 836.4, 836.5	S83.7, S83.9, S83.8, S83.0, S83.1, S83
Group 5	838, 840.7, 840.6, 840.5, 840.4, 840.3, 840.2, 837, 840.1, 837.0, 840.0, 838.0, 838.1, 839, 839.0, 839.1, 839.2, 837.1, 839.3, 839.5, 839.6, 839.7, 839.8, 839.9, 840, 839.4, 840.8, 848, 841, 846.0, 846.1, 846.2, 846.3, 846.8, 846.9, 847, 847.0, 847.2, 847.3, 847.4, 847.9, 848.0, 848.1, 848.2, 848.3, 848.4, 848.5, 848.8, 848.9, 849, 846, 840.9, 845.1, 845, 841.0, 841.1, 841.2, 841.3, 841.8, 841.9, 842, 842.0, 842.1, 843, 843.0, 843.1, 843.8, 843.9, 844, 844.0, 844.1, 844.2, 844.3, 844.8, 844.9, 845.0, 834.1, 847.1, 834, 905.8, 834.0, 830, 905.7, 830.1, 832, 830.0, 832.1, 832.2, 833, 833.0, 833.1, 832.0, 905.6	S86.8, S43.8, S43.7, S43.6, S43.5, S86.9, S66.5, S66.6, S43.9, S66.7, S46, S43.4, S96.0, S93.0, T03.9, T03.8, T03.4, T03.3, T03.2, T03.1, T03.0, T03, SO6.9, S99.9, S99.8, S99.7, S99, S66.9, S93, S66.8, S96.8, S96.7, S96.2, S96.1, S69, S39.0, S39.6, S96, S93.6, S93.5, S93.4, S93.3, S93.2, S93.1, S96.9, S69.7, S46.9, S69.9, S73.1, S76, S76.0, S53.4, S53.7, S56, S56.0, S76.1, S76.2, S76.3, S56.1, S56.2, S56.3, S56.4, S56.5, S56.7, S56.8, S56.9, S76.4, S76.7, S76.8, S59.7, S59.8, S59.9, S66.4, S66.3, S66.2, S66.1, S76.9, S53.3, S53.2, S53.1, S63, S63.9, S63.7, S63.6, S63.5, S63.4, S63.3, S46.0, S46.1, S46.2, S46.3, S46.7, S46.8, S86.7, S86.3, S69.8, S86.2, S49.8, S49.9, S86.0, S86, S83.6, S83.5, S83.4, S83.3, S83.2, S63.2, S63.1, S63.0, S53, S53.0, S86.1, S33.8, S33.2, S66.0, S23.3, S23.4, S23.5, S23.8, S23.9, T93.3, S23.2, T93.5, S29, S29.0, S03.9, S03.8, S03.5, S03.4, S13.8, S23.1, S23.0, S23, S13.6, S13.5, S16, S16.1, S13.4, S13.3, S13.2, S16.2, S16.8, S16.9, T92.3, S13.1, S13.0, S13, T06.4, S03.3, S03.2, S13.9, S33.4, T14.3, S33.9, S33, S33.1, S33.7, T13.2, S33.5, S33.6, S66, T11.2, T09.5, S03, S03.0, S03.1, T09.2, S33.0, S33.3
Group 6	810, 810.0, 810.1, 811, 812.1, 811.1, 812, 812.0, 812.2, 812.3, 812.4, 812.5, 811.0	T92.1, S49.7, S49.1, S49.0, S42.9, S42.7, S49, S42.8, S42.0, S42.1, S42, S42.2, S42.3, S42.4
Group 7	802.8, 802.9, 802.7, 802.2, 802.5, 802.4, 802.3, 802.1, 802.0, 802, 802.6	S02.2, S02.7, S02.6, S02.5, S02.4, S02.3
Group 8	826.6, 826.1, 826.0, 826, 825.3, 825.1, 825.0, 825, 825.2	S92.9, S92.7, S92.2, S92.4, S92, S92.0, S92.1, S92.3, S92.5
Group 9	814, 814.0, 814.1, 815, 815.1, 815.0, 816.0, 817.1, 816, 816.1, 817	S62.3, S62.7, S62.0, S62.1, S62.2, T92.2, S62.4, S62.5, S62.6, S62.8, S62.9, S62
Group 10	820.9, 820.8, 820.3, 820.2, 820.0, 820.1, 820, 905.3	S72.0, S72.1, S72.2
Group 11	823.8, 823.9, 824, 824.0, 824.1, 824.2, 824.5, 905.4, 824.9, 824.8, 824.7, 823.4, 824.4, 824.6, 824.3, 823.3, 822.0, 822, 822.1, 823, 823.0, 823.1, 823.2	S82.5, S89.1, S89.2, S89.3, S89.7, S82.9, S82.8, S82.7, S82.6, S82.4, S89.0, S82.2, S82.1, S82.0, S82, S82.3
Group 12	808.8, 808.5, 808.0, 808, 808.4, 808.9, 808.2, 808.1, 808.3	S32.3, S32.9, S32.8, S32.7, S32.5, S32.4
Group 13	813, 813.0, 813.1, 813.3, 813.4, 813.5, 813.8, 813.9, 905.2, 813.2	S52.6, S52.7, S59.0, S52.9, S59.1, S52.8, S52.4, S52.5, S52.2, S59.2, S52.3, S59, S52.1, S52, S52.0
Group 14	uns, 905.0, 803.1, 803.2, 803.3, 803.4, 803.5, 803.6, 803.7, 803.8, 803.9, 804, 803.0, 804.0, 804.2, 804.4, 804.5, 804.6, 804.7, 804.8, 804.9, 831, 831.0, 831.1, 804.1, 803, 804.3, 801.8, 800.2, 800.3, 800.4, 800.5, 800.6, 800.7, 801.9, 800.8, 800.9, 800.0, 800.1, 801, 800, 801.6, 801.5, 801.4, 801.7, 801.2, 801.1, 801.0, 801.3	S02.1, S02, S43.0, S43, S02.8, S43.3, S43.2, S43.1, S02.0, S02.9
Group 15	807.3, 807.2, 807.1, 807.0, 807	S22.9, S22.5, S22.4, S22.3, S22.2, S22.8
Group 16	805.9, 805.8, 805.5, 805.6, 805.7, 805, 805.0, 805.1, 905.1, 805.3, 805.2, 805.4	S12.6, S12.9, S12.4, S12.3, S12.2, S12.1, S12, S12.7, S12.8, S12.5, S12.0, T08, S22, T08.0, S32.2, T91.1, S22.0, S22.1, S32.1, S32.0, S32
Group 17	821.2, 821.3, 821.0, 821, 821.1	S72.4, S72.7, S72.8, S72.9, S72.3, S72, T93.1, S79, S79.1, S79.0
Group 18	850, 850.9, 850.5, 850.4, 850.3, 850.2, 850.1, 850.0	S06.0
Group 19	907, 852.0, 907.0, 852.1, 852, 852.2, 852.3, 852.4, 852.5	T90.5, S06.3, S06.4, S06.6, S06.9, S06.5, T90.2, S06, S06.1, S07

Group 20	851.0, 851.8, 851.7, 851, 854.1, 854.0, 854, 853.1, 851.9, 851.5, 851.4, 851.3, 851.6, 851.2, 853.0, 853, 851.1	S06.2, S06.8, S06.7
Group 21	935.1, 935.2, 936, 937, 939, 938.9, 935.0, 939.0, 939.1, 939.2, 938, 935, 934.0, 934.8, 934.1, 934, 933.1, 933.0, 933, 932, 939.9, 931, 934.9, 939.3	T17.8, T17.4, T17.9, T18, T17.5, T16.2, T17.3, T17.2, T17.1, T17.0, T16, T17, T16.9, T16.1, T18.0, T19.0, T18.2, T18.3, T18.4, T18.5, T18.8, T18.9, T19, T19.1, T19.2, T19.3, T19.4, T19.8, T19.9, T18.1
Group 22	952, 952.0, 806.0, 806, 806.1	S14.1, T91.3, S14.0, S14
Group 23	806.9, 952.3, 952.4, 952.8, 952.9, 952.2, 806.8, 806.7, 806.6, 806.5, 952.1, 806.3, 806.2, 806.4	S24.1, S24.0, S24, S34, S34.0, S34.1
Group 24	994.1, 995.0	T75.1
Group 25	994.7, 995.1	T71.2, T71.9, T71, T71.1, T71.0
Group 26	906.4, 995.2, 927.3, 927.2, 927.1, 926.8, 926.9, 926.1, 926.0, 926, 925.2, 925.1, 925, 927, 927.0, 928, 928.3, 928.8, 928.2, 928.1, 928.0, 928.9, 927.9, 927.8, 929.9	S67.9, S97.0, S77.0, S77.1, S77.2, S87, S87.0, S87.8, S97, S97.1, S97.8, S67.8, S77, S67.4, S67.1, S67.2, S47.1, S47, S57, S38.2, S57.8, S57.9, T92.6, S47.2, S67.3, S07.8, S07.1, S07.0, T93.6, S38, S38.0, S38.1, S07.9, S47.9, S57.0, S38.3, S18, S17.8, S17.0, S17.9, S17, S67.0, S67
Group 27	955.2, 955.4, 955.1, 907.8, 907.5, 907.4, 955.0, 955, 907.1, 954.9, 955.6, 954.8, 955.3, 955.5, 951.8, 955.7, 955.8, 956.9, 956.8, 956.5, 956.4, 951.0, 956.3, 956.2, 956.1, 956.0, 956, 955.9, 954.1, 954.0, 953.4, 953.9, 951.9, 957.0, 957, 951.6, 951.5, 951.4, 951.3, 953, 953.0, 953.1, 953.2, 957.1, 951.2, 954, 957.8, 950, 950.0, 950.1, 950.2, 950.3, 950.9, 951, 995.2, 907.9, 953.3, 951.7, 953.5, 953.8, 957.9, 951.1, 907.3	S54.2, S94.9, S44.0, S54.3, S94.8, S94.7, S94.3, S94.2, S94.1, S94, S44, S44.1, S84.8, S54.1, S84.9, S54, S84, S84.0, S44.3, S84.1, S44.4, S44.5, S44.7, S44.8, S44.9, S84.2, S84.7, S44.2, S54.0, S04.2, S04.0, S14.2, S14.3, S14.4, S14.5, S14.6, S14.8, S14.9, T90.3, S24.9, S24.8, S24.6, S24.5, S24.4, S24.3, S24.2, S34.2, T13.3, S34.3, S34.4, S04.1, S54.7, S04.3, S04.4, S04.5, S04.6, S04.7, S04, S04.8, T93.4, T92.4, S34.9, S34.8, S34.6, S34.5, T11.3, S04.9, S54.8, S94.0, S64.3, S64.0, S64, S64.4, S74, S74.0, S74.1, S74.2, S64.9, S74.8, S74.7, S64.1, S64.2, S54.9, S64.7, S74.9, S64.8
Group 28	871.0, 940.4, 940.3, 940.2, 940.1, 940.0, 940, 918.0, 870.4, 930.9, 930.8, 930.2, 930.1, 930.0, 930, 940.5, 870.3, 870.2, 870.1, 870.0, 870, 921.9, 921.3, 921.2, 921.1, 921.0, 921, 871.1, 918.9, 918.2, 918.1, 995.2, 940.9, 918, 871, 871.2, 871.3, 871.4, 871.5, 871.6, 871.7, 871.9, 870.9, 870.8	S05.9, T26.9, T26.1, T26.0, T15.9, T15.8, T26.3, T15.0, T15, T14.4, T26.4, T26.5, T26.6, T26.7, T26.8, T15.1, T26, T26.2, S05.7, S05.6, S05.5, S05.4, S05.3, T90.4, S05.8, S05.2, S01.1, S05.1, S05.0, S05
Group 29	883.2, 884, 884.2, 884.1, 883.1, 884.0, 873, 873.0, 883, 882.2, 890, 882.1, 873.1, 873.2, 873.3, 873.4, 883.0, 890.0, 874.9, 890.2, 872, 872.0, 872.1, 872.6, 872.7, 872.8, 872.9, 893, 892.2, 892.1, 892.0, 892, 891.2, 891.1, 873.5, 891.0, 891, 890.1, 873.6, 882, 874.8, 874.3, 874.4, 874.5, 878.6, 878.5, 878.4, 878.3, 878.2, 878.1, 874.2, 878.0, 877.1, 877.0, 877, 876.1, 876.0, 876, 875.1, 875.0, 875, 878, 882.0, 873.9, 878.8, 881.2, 881.1, 881.0, 881, 880.2, 880.1, 880.0, 880, 879.9, 878.7, 879.8, 879.6, 879.5, 879.4, 879.3, 873.7, 873.8, 879.0, 879, 878.9, 879.7, 879.1, 879.2, 900.1, 904.9, 900.8, 900.0, 900.9, 893.2, 894, 893.1, 894.0, 893.0, 906, 906.0, 906.1, 906.2, 894.1, 894.2, 904.8, 900, 904.7, 904.5, 903, 903.0, 903.1, 903.2, 903.3, 903.4, 903.5, 903.8, 903.9, 904, 904.0, 904.1, 904.2, 904.3, 904.4, 904.6, 995.2	S10, T01.8, S10.7, T93.0, S11, S09.3, S11.1, T01.3, T92.5, T01.1, T01.0, T01.6, T01.9, S61.2, S09.1, S09.0, S09, S08.9, S61.3, S61.4, S61.5, S08.8, S08.1, S08.0, S08, S61.9, S61.8, S61.7, S09.2, S55, S71.0, T09.1, S15.3, S15.7, S15.8, S15.9, S65, S65.0, S65.1, T92.0, S65.2, S21, S21.0, S21.1, S21.2, S21.3, T90.1, S65.3, S65.4, S65.5, S65.7, S65.8, S65.9, S21.9, S21.8, S15.2, S15.1, S15.0, S15, S71.7, S71.1, T11.1, T01, S71, T11.4, T11.5, T13.1, T13.4, T13.5, T14.1, S71.8, T14.5, S31.8, S31.7, S31.5, S31.4, S31.3, S31.2, S31.1, S31.0, S31, S11.8, S11.9, T14.6, S01.9, T01.2, S01.7, S45.8, S45.7, S45.3, S45.2, S45.1, S45.0, S45, S55.7, S55.8, S45.9, S55.9, S91, S91.0, S91.1, S91.2, S91.3, S91.7, S41.8, S41.7, S41.1, S01.8, S41.0, S85.9, S85.7, S55.0, S55.1, S55.2, S81.9, S81.8, S51.9, S51.8, S51.7, S51.0, S85.8, S51, S81.0, S81, S85, S85.0, S85.1, S85.2, S85.3, S85.4, S85.5, S81.7, S41, S21.4, S75.7, S95, S95.0, S95.1, S95.2, S95.7, S75.9, S95.8, S95.9, S39.7, S39, S61.1, S61.0, S75.8, S61, S01.2, S21.7, S75.1, S75.2, S75, S01, S01.0, S75.0, S01.3, S01.4, S01.5

Group 30	969.6, 964.9, 965, 965.0, 965.1, 965.5, 964.8, 965.4, 964.3, 964.6, 964.5, 964.4, 964.2, 964.1, 964.0, 964, 963.9, 963.8, 963.5, 964.7, 965.6, 967.1, 965.8, 968.7, 968.6, 968.5, 968.4, 968.3, 968.2, 968.1, 968.0, 968, 967.9, 967.8, 967.6, 965.7, 967.5, 967.3, 967.2, 963.4, 967.0, 967, 966.4, 966.3, 966.2, 966.1, 966.0, 966, 965.9, 967.4, 963.3, 963, 963.1, 972.1, 972.2, 972.3, 960.0, 960.1, 960.2, 960.3, 972.0, 960.4, 972.4, 972.5, 972.6, 972.7, 972.8, 972.9, 973, 960.5, 972, 971.9, 960, 969.7, 969.8, 969.9, 970, 970.0, 970.1, 970.8, 969.4, 969.3, 969.2, 969.1, 969.0, 970.9, 971, 971.0, 971.1, 971.2, 973.0, 963.2, 973.1, 973.3, 961.7, 961.8, 961.9, 962, 962.0, 962.1, 962.2, 961.6, 962.3, 962.5, 962.6, 962.7, 962.8, 962.9, 969.5, 963.0, 962.4, 961.5, 961.4, 961.3, 973.4, 973.5, 973.6, 973.8, 973.9, 974, 974.0, 974.1, 974.2, 960.6, 960.7, 960.8, 960.9, 961, 961.0, 961.1, 961.2, 973.2, 971.3, 989, 979.1, 981.5, 995.2, 981.6, 981.7, 981.9, 982, 982.0, 982.1, 982.2, 982.3, 982.4, 982.8, 983, 983.0, 983.1, 981.3, 981.2, 981, 980.9, 978.9, 979, 979.0, 979.2, 979.3, 979.4, 979.5, 983.2, 979.6, 979.9, 980, 980.0, 980.1, 980.2, 980.3, 980.8, 979.7, 978.8, 983.5, 983.9, 987.2, 987.3, 987.4, 987.5, 987.6, 987.7, 988.9, 987.8, 987.9, 988, 988.0, 988.1, 988.2, 988.6, 988.8, 987.1, 987.0, 987, 986, 984, 984.0, 984.1, 984.3, 984.8, 984.9, 985, 983.7, 985.0, 985.2, 985.3, 985.4, 985.5, 985.6, 985.8, 985.9, 985.1, 978.6, 969, 989.6, 975.4, 975.5, 977.3, 977.2, 977.1, 977.0, 977, 976.9, 989.9, 989.8, 989.7, 975.6, 989.5, 989.4, 989.3, 989.2, 989.1, 976.8, 976.7, 976.6, 976.5, 976.4, 976.3, 976.2, 976.1, 976.0, 976, 975.3, 975.2, 975.1, 975.0, 968.9, 989.0, 978.5, 974.3, 978.4, 978.3, 978.2, 978.1, 978.0, 978, 977.9, 977.8, 975.8, 977.4, 974.5, 974.6, 974.7, 975, 974.4, 975.7	T45.4, T45.3, T45.5, T40, T40.0, T45.6, T42.6, T45.8, T39.9, T96, T96.0, T46.2, T42.8, T43, T43.0, T43.1, T43.2, T46.1, T46.0, T46, T42.7, T40.1, T42.5, T42.4, T45.9, T45.7, T40.2, T44.8, T43.3, T40.7, T44.4, T44.5, T40.8, T40.9, T41.3, T41.2, T44.3, T41.1, T41, T45.2, T45.1, T45.0, T45, T44.6, T44.7, T41.0, T40.3, T40.6, T44.1, T43.4, T42.3, T42.2, T42.1, T42.0, T44.9, T42, T40.5, T41.5, T43.5, T43.6, T43.8, T40.4, T43.9, T44, T44.0, T41.4, T44.2, T60.2, T65.8, T57.8, T57.3, T50.4, T57.2, T57.1, T57.0, T57, T57.9, T56.9, T56.7, T56.6, T56.5, T50.5, T50.6, T50.7, T50.8, T56.8, T58, T58.0, T58.1, T49.6, T49.7, T49.8, T49.9, T50, T50.0, T50.1, T50.2, T50.3, T59.3, T59.2, T59.1, T59.0, T59, T58.9, T58.8, T58.2, T50.9, T51, T51.0, T51.1, T54.3, T54.2, T54.1, T54.0, T54, T53.9, T53.7, T52.9, T53, T53.0, T53.1, T53.2, T53.3, T53.4, T53.5, T53.6, T39.8, T54.9, T49.5, T55, T52.8, T51.2, T51.3, T51.8, T56.4, T56.3, T56.2, T56.1, T56.0, T56, T55.1, T51.9, T52, T52.0, T52.1, T52.2, T52.3, T52.4, T55.0, T65.9, T49.4, T49.2, T63, T62.9, T62.8, T62.2, T62.1, T62.0, T62, T63.0, T61.9, T61.7, T61.2, T46.3, T46.4, T46.5, T46.6, T61.1, T61.8, T63.1, T63.2, T63.3, T65.6, T65.5, T65.4, T65.3, T65.2, T65.1, T65.0, T65, T64.8, T64.0, T64, T63.9, T63.8, T63.7, T63.6, T63.5, T63.4, T46.7, T46.8, T46.9, T47, T48.3, T48.4, T48.5, T60.0, T60, T59.9, T59.8, T59.7, T59.6, T59.5, T59.4, T48.6, T48.7, T48.9, T49, T49.0, T49.1, T48.2, T49.3, T48.1, T48, T61.0, T47.0, T47.1, T47.2, T47.3, T47.4, T47.5, T47.6, T61, T60.9, T60.8, T60.4, T60.3, T60.1, T47.7, T47.8, T47.9, T48.0, T39.4, T97.0, T39.2, T37.1, T37.0, T37, T36.9, T36.8, T36.7, T36.6, T36.5, T36.4, T36.3, T36.2, T36.1, T36.0, T36, T39.3, T37.2, T37.3, T97, T37.5, T39.1, T39.0, T39, T38.9, T38.8, T37.4, T38.6, T38.5, T38.7, T38.3, T38.2, T38.1, T38.0, T38, T37.9, T37.8, [illegible]
Group 31	901.9, 901.8, 901.4, 901.3, 901.2, 901.1, 901.0, 901, 908.0, 874.1, 807.1, 862.9, 807.4, 807.5, 807.6, 874, 860, 860.0, 860.1, 860.2, 860.3, 860.4, 860.5, 861, 861.0, 861.1, 995.2, 861.2, 861.3, 862, 862.0, 862.1, 862.2, 862.3, 862.8, 807.0, 874.0	S27.1, S27.2, S27.3, S27.4, S27.5, S27.6, S29.8, S27.8, S27.9, S28, S28.1, S28.2, S29.7, S27.0, S27.7, S27, S25.5, S26.8, S11.0, S29.9, S11.7, T91.4, S25, S25.0, S25.1, S26.9, S25.2, S25.4, S25.7, S25.8, S25.9, S26, S26.0, S26.1, S25.3, S11.2, S28.0
Group 32	902.9, 868.0, 866.1, 867, 867.0, 867.2, 867.3, 867.4, 867.5, 867.6, 867.7, 867.8, 867.9, 868, 902.8, 868.1, 868.3, 869, 869.0, 869.1, 908.3, 908.2, 908.1, 866.0, 866, 867.1, 865.0, 902.5, 902.4, 902.3, 902.2, 902.1, 902.0, 865.1, 995.2, 863, 863.0, 863.1, 902, 863.3, 863.2, 864.1, 864.0, 864, 865, 863.8, 863.5, 863.4, 863.9	S37.5, S36.9, S36.6, S35, S35.0, S35.1, S35.2, S35.3, S35.4, S35.5, S35.7, S35.8, S35.9, S36.7, S37.6, S36, S36.0, S36.1, S36.2, S36.3, S36.4, S36.5, S37, S37.0, S36.8, S37.9, T79.2, T79.1, T79.0, T79, S37.7, S37.8, T79.3, S39.8, T79.5, T79.4, T79.7, S37.4, T79.8, S37.3, S37.2, T91.5, S37.1, T79.9, T79.6
Group 33	922.9, 995.2, 920, 922, 922.0, 922.1, 922.2, 922.3, 922.4, 922.8, 923, 924.9, 923.1, 923.0, 924.8, 924.5, 924.4, 924.3, 924.2, 906.3, 924.0, 924, 923.9, 923.8, 923.3, 923.2, 924.1	S70, S60.9, S60.8, S60.7, S50.0, S60.1, S60.3, S60.2, S50, S60.0, S40, S40.0, S40.2, S60, S50.1, S60.5, S70.0, S60.4, S70.2, S20, S20.0, S30, S20.2, S30.0, S30.1, S70.1, S30.3, S90.4, S30.2, S80.0, S80, S90.3, S80.1, S80.2, S80.7, S90, S90.1, S90.2, S90.0
Group 34	993.2, 993.3, 993.4, 993.8, 993.9, 994, 994.0, 994.5, 994.3, 994.4, 994.6, 994.8, 994.9, 993.1, 995.3, 994.2, 993.0, 992, 992.9, 991.0, 991, 990, 993, 991.1, 991.2, 991.3, 991.5, 991.6, 991.8, 991.4, 992.0, 992.1, 992.8, 992.7, 992.2, 992.3, 992.4, 992.6, 991.9, 992.5	T34.1, T34.2, T34.3, T34.0, T34, T33.9, T33.8, T33.7, T33.6, T33.5, T34.4, T69.0, T34.6, T70.1, T70.2, T70.3, T70.4, T70.8, T70.9, T34.8, T34.9, T70.0, T35, T35.1, T75, T75.0, T35.2, T75.2, T75.3, T75.4, T75.8, T35.0, T70, T69.9, T69.8, T34.7, T66, T67, T67.0, T67.1, T67.2, T33.4, T67.3, T67.4, T67.5, T67.6, T67.7, T67.8, T67.9, T68, T68.0, T68.7, T69, T69.1, T34.5, T33.3, T33, T33.1, T95.9, T35.7, T35.6, T35.5, T35.4, T35.3, T95.8, T33.2, T33.0
Group 35	997.9, 998.4, 998.1, 998.2, 998.0, 998, 998.5, 998.3, 998.6, 999.2, 998.8, 998.9, 999, 999.0, 999.1, 997.8, 999.3, 999.4, 999.5, 998.7, 997.7, 996.8, 997.5, 999.7, 995, 995.4, 995.5, 995.6, 995.7, 995.8, 996, 996.0, 996.1, 996.2, 996.3, 996.4, 996.5, 996.6, 996.7, 996.9, 997, 997.0, 997.1, 997.2, 997.3, 997.4, 997.6, 999.6, 999.9, 999.8	T84.5, T84.8, T81.7, T81.6, T81.5, T81.4, T81.3, T84.9, T81.2, T85, T85.0, T85.1, T81.1, T81.0, T81, T80.9, T80.8, T80.6, T80.5, T80.4, T80.3, T80.2, T80.1, T80.0, T80, T81.8, T81.9, T82, T82.0, T84.4, T84.3, T84.2, T84.1, T84.0, T84, T83.9, T83.8, T83.7, T83.6, T83.5, T83.4, T84.6, T83.3, T83.1, T83.0, T83, T82.9, T82.8, T82.7, T82.6, T82.5, T82.4, T84.7, T82.2, T82.1, T83.2, T82.3, T85.3, T88.3, T87, T87.0, T88.9, T88.8, T88.7, T88.6, T88.5, T88.4, T87.1, T85.4, T88.2, T88.1, T88.0, T88, T87.9, T87.8, T87.6, T87.5, T87.4, T86.9, T87.3, T86.8, T86.4, T85.2, T85.5, T85.6, T85.7, T85.8, T85.9, T78.9, T78.8, T78.4, T78.3, T78.2, T78.1, T78.0, T78, T86, T86.0, T86.1, T86.2, T86.3, T86.5, T87.2

Group 36	912.2, 912.3, 912.4, 912.5, 912.6, 912.7, 912.8, 912.9, 913, 913.0, 919.9, 913.2, 913.3, 913.4, 913.5, 913.6, 913.7, 913.8, 914, 914.0, 914.1, 912.1, 913.1, 912.0, 911.9, 910, 910.0, 910.1, 910.2, 910.3, 910.4, 910.5, 910.6, 910.7, 910.8, 910.9, 911, 911.0, 911.1, 911.2, 911.3, 911.4, 911.5, 911.6, 911.7, 911.8, 912, 914.3, 913.9, 914.5, 916.9, 917, 917.0, 914.4, 917.2, 917.3, 917.4, 917.5, 917.6, 917.7, 916.8, 917.8, 919, 919.0, 919.1, 919.2, 919.3, 919.4, 919.5, 919.6, 919.7, 919.8, 917.9, 916.7, 917.1, 916.5, 914.6, 914.7, 914.8, 914.9, 916.6, 915.0, 915.1, 915.2, 915.3, 915.4, 915, 915.6, 915.5, 916.4, 916.3, 916.2, 916.1, 914.2, 916.0, 916, 915.9, 915.8, 915.7	S00.3, T90.0, S00.2, S00.7, S00.8, S00.1, S00.0, S00.9, S10.0, S00.4, S10.1, S10.9, S20.1, S20.3, S20.4, S20.7, S20.8, S20.9, S10.8, S00.5, S00, T14.0, S70.9, S80.8, S80.9, S90.4, S90.5, S90.7, S90.8, S90.9, T00, T00.0, T00.1, T00.3, T00.6, T00.8, T00.9, S70.8, S70.7, T00.2, S70.2, T13.0, T11.0, T09.0, S70.3, S30.7, S30.8, S40.2, S30.9, S40.8, S40.9, S50.3, S50.7, S50.8, S50.9, S40.7
Group 37	828.0, 828, 827.1, 827.0, 827, 819.1, 819.0, 819, 817.0, 929.0, 828.1	T04.7, T04.3, S09.7, T04.4, T04.2, T04.8, T06, T05, T05.8, T05.9, T04.1, T06.0, T06.1, T06.2, T04.9, T04.0, T02.4, T02.9, S79.7, T06.8, T07, T06.5, T07.0, T02, T02.0, T02.1, T02.2, T02.3, T02.5, T02.6, T02.7, S19.7, T02.8, T04, T06.3

Appendix Figure 13

Redistribution Proportions for Unspecified External Factor (X59), Year 2015, Top 20 Underlying Causes

Age Group and Cause	Central Europe, Eastern Europe, and Central Asia	High-income	Latin America and Caribbean	North Africa and Middle East	South Asia	Southeast Asia, East Asia, and Oceania	Sub-Saharan Africa
**0 to 14**							
Falls	26.08%	17.32%	14.88%	23.63%	47.13%	30.68%	16.34%
Other unintentional injuries	28.14%	12.53%	21.91%	27.53%	16.03%	16.32%	29.38%
Pedestrian road injuries	12.61%	18.31%	22.24%	17.43%	14.84%	22.71%	18.28%
Pulmonary aspiration and foreign body in airway	11.03%	19.32%	18.32%	4.31%	2.72%	9.70%	6.81%
Motor vehicle road injuries	6.77%	12.64%	6.80%	13.69%	2.41%	3.45%	8.01%
Other exposure to mechanical forces	2.24%	2.72%	1.89%	3.50%	2.37%	3.97%	4.88%
Motorcyclist road injuries	1.06%	1.92%	3.05%	2.97%	1.74%	3.00%	1.84%
Poisoning by other means	1.16%	1.26%	1.16%	0.88%	1.45%	1.20%	2.69%
Cyclist road injuries	1.23%	3.55%	2.00%	0.70%	1.40%	1.99%	1.15%
Other transport injuries	1.13%	3.09%	1.17%	1.15%	1.45%	0.94%	1.49%
Drowning	1.19%	0.86%	0.81%	0.43%	1.59%	1.86%	1.05%
Adverse effects of medical treatment	0.87%	1.40%	1.44%	0.82%	1.04%	0.82%	1.88%
Non-venomous animal contact	0.57%	0.75%	0.56%	0.43%	0.78%	0.49%	1.85%
Venomous animal contact	0.05%	0.05%	0.21%	0.20%	2.49%	0.17%	0.56%
Other road injuries	0.64%	1.11%	1.54%	0.43%	0.69%	0.59%	0.85%
Environmental heat and cold exposure	0.79%	0.61%	0.31%	0.23%	1.05%	0.23%	0.89%
Poisoning by carbon monoxide	2.76%	0.85%	0.29%	0.72%	0.19%	1.16%	0.33%
Fire, heat, and hot substances	0.78%	0.64%	0.33%	0.40%	0.36%	0.21%	0.73%
Unintentional firearm injuries	0.35%	0.70%	0.65%	0.37%	0.05%	0.11%	0.62%
Foreign body in other body part	0.57%	0.34%	0.43%	0.18%	0.21%	0.40%	0.37%
**15-49 years**							
Other unintentional injuries	24.90%	10.65%	22.98%	24.68%	30.29%	26.06%	20.50%
Falls	29.89%	27.27%	16.45%	17.08%	25.19%	24.91%	15.42%
Pedestrian road injuries	10.66%	11.96%	18.22%	16.01%	13.45%	21.36%	22.13%
Motorcyclist road injuries	3.36%	11.49%	17.43%	7.50%	13.60%	9.71%	4.67%
Motor vehicle road injuries	12.59%	23.08%	12.38%	21.84%	5.77%	6.39%	18.91%
Other exposure to mechanical forces	2.25%	2.33%	1.53%	4.93%	1.66%	3.36%	3.54%
Other transport injuries	2.36%	3.27%	1.90%	2.17%	3.00%	1.44%	2.98%
Cyclist road injuries	1.02%	2.12%	2.04%	0.78%	2.59%	2.64%	2.23%
Other road injuries	0.43%	0.86%	2.06%	0.45%	0.51%	0.81%	1.46%
Pulmonary aspiration and foreign body in airway	2.76%	2.41%	1.65%	0.72%	0.13%	0.44%	1.21%
Environmental heat and cold exposure	4.43%	0.77%	0.23%	0.24%	0.69%	0.18%	1.20%
Poisoning by carbon monoxide	2.82%	1.03%	0.22%	0.89%	0.11%	0.77%	0.35%
Poisoning by other means	0.34%	0.58%	0.39%	0.39%	0.28%	0.89%	1.05%
Adverse effects of medical treatment	0.42%	0.69%	0.44%	0.73%	0.70%	0.28%	1.19%
Venomous animal contact	0.04%	0.03%	0.06%	0.03%	1.11%	0.05%	0.31%
Unintentional firearm injuries	0.41%	0.62%	1.20%	0.71%	0.07%	0.10%	1.15%
Drowning	0.45%	0.27%	0.32%	0.18%	0.29%	0.28%	0.26%
Non-venomous animal contact	0.16%	0.20%	0.19%	0.12%	0.19%	0.19%	0.95%
Fire, heat, and hot substances	0.37%	0.21%	0.10%	0.34%	0.30%	0.07%	0.35%
Foreign body in other body part	0.34%	0.15%	0.20%	0.21%	0.06%	0.08%	0.13%
**50 to 59**							
Falls	41.33%	56.77%	37.23%	27.80%	49.08%	38.74%	33.98%
Pedestrian road injuries	9.42%	10.37%	21.78%	21.78%	13.58%	24.48%	21.75%
Other unintentional injuries	18.89%	6.19%	18.13%	13.22%	20.59%	19.31%	14.28%
Motor vehicle road injuries	5.99%	10.75%	8.95%	23.37%	4.36%	4.44%	12.90%
Other exposure to mechanical forces	2.35%	2.55%	1.86%	4.52%	1.57%	3.41%	4.31%
Cyclist road injuries	1.27%	2.27%	2.76%	1.21%	2.43%	3.75%	2.08%
Other transport injuries	1.64%	2.24%	1.61%	2.49%	2.76%	1.30%	1.55%
Environmental heat and cold exposure	7.77%	1.23%	0.46%	0.46%	1.48%	0.26%	1.02%
Pulmonary aspiration and foreign body in airway	4.04%	3.52%	2.33%	1.04%	0.31%	0.57%	1.15%
Motorcyclist road injuries	0.91%						
Other road injuries	0.46%	0.56%	1.90%	0.54%	0.72%	0.77%	1.22%
Poisoning by carbon monoxide	3.25%	0.63%	0.17%	0.77%	0.06%	0.77%	0.49%
Adverse effects of medical treatment	0.58%	1.18%	0.88%	1.06%	1.06%	0.29%	1.01%
Poisoning by other means	0.36%	0.45%	0.33%	0.32%	0.17%	1.03%	1.16%
Venomous animal contact	0.04%	0.04%	0.11%	0.04%	1.05%	0.10%	0.70%
Non-venomous animal contact	0.27%	0.28%	0.35%	0.18%	0.27%	0.30%	1.13%
Drowning	0.33%	0.22%	0.24%	0.11%	0.22%	0.23%	0.18%

Unintentional firearm injuries	0.34%	0.35%	0.55%	0.57%	0.06%	0.07%	0.58%
Fire, heat, and hot substances	0.51%	0.27%	0.14%	0.31%	0.16%	0.08%	0.42%
Foreign body in other body part	0.25%	0.11%	0.22%	0.19%	0.06%	0.09%	0.07%
**60 to 69**							
Falls	51.05%	67.52%	50.86%	37.85%	69.88%	47.22%	45.75%
Pedestrian road injuries	9.05%	8.19%	20.73%	23.17%	10.13%	25.70%	19.62%
Other unintentional injuries	15.77%	3.57%	10.81%	8.49%	9.22%	11.68%	11.82%
Motor vehicle road injuries	4.15%	6.98%	6.00%	18.86%	2.19%	3.04%	9.93%
Cyclist road injuries	1.40%	2.03%	2.25%	0.95%	1.62%	4.13%	1.94%
Other exposure to mechanical forces	1.68%	1.84%	1.35%	3.32%	1.05%	2.31%	2.10%
Pulmonary aspiration and foreign body in airway	3.73%	3.75%	2.37%	1.11%	0.29%	0.73%	1.03%
Other transport injuries	1.31%	1.47%	1.03%	2.18%	1.29%	0.98%	0.88%
Environmental heat and cold exposure	6.11%	1.07%	0.34%	0.31%	1.14%	0.32%	0.91%
Adverse effects of medical treatment	0.85%	1.61%	1.27%	1.39%	1.49%	0.36%	1.30%
Poisoning by carbon monoxide	2.75%	0.37%	0.14%	0.64%	0.05%	1.02%	0.38%
Poisoning by other means	0.30%	0.26%	0.29%	0.27%	0.11%	1.14%	1.06%
Other road injuries	0.35%	0.32%	1.28%	0.35%	0.28%	0.47%	0.52%
Venomous animal contact	0.04%	0.03%	0.11%	0.04%	0.78%	0.09%	0.75%
Non-venomous animal contact	0.24%	0.20%	0.32%	0.19%	0.14%	0.30%	0.76%
Drowning	0.26%	0.22%	0.18%	0.09%	0.14%	0.26%	0.17%
Fire, heat, and hot substances	0.50%	0.26%	0.15%	0.28%	0.12%	0.10%	0.47%
Unintentional firearm injuries	0.26%	0.22%	0.34%	0.37%	0.04%	0.05%	0.58%
Foreign body in other body part	0.21%	0.09%	0.18%	0.12%	0.03%	0.10%	0.04%
**70 to 79**							
Falls	65.49%	79.59%	66.07%	55.00%	85.46%	67.45%	61.41%
Pedestrian road injuries	10.07%	6.29%	16.79%	18.85%	5.59%	18.27%	14.52%
Other unintentional injuries	8.66%	1.40%	5.81%	4.73%	2.92%	4.85%	7.26%
Motor vehicle road injuries	2.89%	3.81%	3.33%	12.45%	1.04%	1.97%	7.49%
Pulmonary aspiration and foreign body in airway	2.03%	3.43%	2.32%	0.97%	0.37%	1.02%	1.14%
Other exposure to mechanical forces	1.21%	1.02%	0.92%	2.48%	0.62%	1.56%	1.50%
Adverse effects of medical treatment	1.22%	1.58%	1.45%	1.55%	1.12%	0.44%	1.11%
Environmental heat and cold exposure	3.22%	0.85%	0.30%	0.34%	0.99%	0.42%	1.11%
Other transport injuries	1.22%	0.72%	0.61%	1.46%	0.63%	0.67%	0.47%
Poisoning by carbon monoxide	2.01%	0.19%	0.13%	0.56%	0.04%	1.06%	0.29%
Poisoning by other means	0.23%	0.17%	0.22%	0.23%	0.04%	0.98%	0.72%
Other road injuries	0.36%	0.17%	0.88%	0.36%	0.29%	0.37%	0.48%
Venomous animal contact	0.03%	0.02%	0.11%	0.04%	0.51%	0.10%	0.59%
Non-venomous animal contact	0.26%	0.13%	0.32%	0.22%	0.14%	0.24%	0.57%
Fire, heat, and hot substances	0.50%	0.22%	0.17%	0.29%	0.09%	0.14%	0.51%
Drowning	0.16%	0.19%	0.11%	0.06%	0.09%	0.27%	0.15%
Unintentional firearm injuries	0.23%	0.14%	0.25%	0.28%	0.04%	0.06%	0.62%
Foreign body in other body part	0.22%	0.09%	0.21%	0.13%	0.03%	0.15%	0.05%
**80 to 89**							
Falls	81.33%	87.49%	79.74%	73.13%	88.84%	84.12%	75.90%
Pedestrian road injuries	5.47%	3.34%	9.11%	11.50%	3.54%	7.88%	8.19%
Other unintentional injuries	4.16%	0.63%	3.66%	2.94%	3.01%	2.23%	5.00%
Pulmonary aspiration and foreign body in airway	1.05%	3.33%	2.13%	0.79%	0.50%	0.93%	1.04%
Motor vehicle road injuries	1.34%	1.68%	1.44%	6.08%	0.59%	0.80%	3.93%
Adverse effects of medical treatment	1.08%	1.15%	1.23%	1.37%	0.96%	0.31%	1.04%
Other exposure to mechanical forces	0.90%	0.65%	0.54%	1.59%	0.41%	0.88%	1.39%
Environmental heat and cold exposure	1.46%	0.72%	0.25%	0.24%	0.76%	0.50%	0.60%
Other transport injuries	0.60%	0.19%	0.28%	0.65%	0.46%	0.30%	0.22%
Poisoning by carbon monoxide	1.29%	0.09%	0.08%	0.36%	0.05%	0.63%	0.14%
Poisoning by other means	0.16%	0.11%	0.14%	0.19%	0.04%	0.47%	0.42%
Other road injuries	0.15%	0.08%	0.42%	0.26%	0.23%	0.16%	0.36%
Fire, heat, and hot substances	0.43%	0.20%	0.17%	0.28%	0.09%	0.15%	0.41%
Non-venomous animal contact	0.16%	0.07%	0.21%	0.20%	0.09%	0.13%	0.41%
Venomous animal contact	0.02%	0.01%	0.09%	0.04%	0.28%	0.05%	0.30%
Unintentional firearm injuries	0.17%	0.10%	0.23%	0.23%	0.05%	0.08%	0.51%
Drowning	0.07%	0.10%	0.05%	0.03%	0.07%	0.18%	0.09%
Foreign body in other body part	0.15%	0.07%	0.22%	0.11%	0.04%	0.17%	0.04%
**90 plus**							
Falls	89.50%	90.65%	85.53%	82.04%	90.96%	90.85%	81.42%
Pedestrian road injuries	2.12%	1.60%	4.51%	5.86%	1.95%	3.40%	4.38%
Pulmonary aspiration and foreign body in airway	0.80%	3.50%	2.23%	0.86%	0.65%	0.88%	1.24%
Other unintentional injuries	2.62%	0.45%	3.19%	3.06%	2.90%	1.68%	5.05%
Motor vehicle road injuries	0.74%	0.92%	0.91%	3.43%	0.65%	0.57%	2.33%

Adverse effects of medical treatment	0.91%	0.94%	1.10%	1.19%	1.00%	0.24%	1.13%
Other exposure to mechanical forces	0.77%	0.63%	0.53%	1.28%	0.35%	0.59%	1.42%
Environmental heat and cold exposure	0.83%	0.63%	0.32%	0.28%	0.54%	0.48%	0.68%
Fire, heat, and hot substances	0.29%	0.20%	0.19%	0.24%	0.06%	0.12%	0.32%
Other transport injuries	0.24%	0.07%	0.20%	0.46%	0.33%	0.19%	0.15%
Unintentional firearm injuries	0.13%	0.09%	0.29%	0.22%	0.05%	0.11%	0.73%
Poisoning by carbon monoxide	0.64%	0.04%	0.09%	0.25%	0.04%	0.25%	0.09%
Foreign body in other body part	0.12%	0.06%	0.27%	0.12%	0.04%	0.19%	0.05%
Poisoning by other means	0.10%	0.07%	0.13%	0.15%	0.03%	0.17%	0.22%
Non-venomous animal contact	0.08%	0.05%	0.20%	0.33%	0.07%	0.08%	0.36%
Other road injuries	0.05%	0.05%	0.18%	0.14%	0.17%	0.07%	0.21%
Drowning	0.04%	0.04%	0.04%	0.02%	0.04%	0.09%	0.05%
Venomous animal contact	0.01%	0.01%	0.10%	0.05%	0.17%	0.03%	0.16%

Proportions in the above table are unique by super region and age group. For each individual percentage in the table, the denominator used in its calculation was the total deaths redistributed across all causes within its respective super region and age group. Thus, column percentages within each age group will add to, or very close to, 100%. Only the top 20 redistribution targets are listed, so it is possible that some columns may not quite total 100%.

Appendix Figure 14

Redistribution Proportions for External Causes UDI, Type Unspecified (Y34), Year 2015, Top 20 Underlying Causes							
Age Group and Cause	Central Europe, Eastern Europe, and Central Asia	High-income	Latin America and Caribbean	North Africa and Middle East	South Asia	Southeast Asia, East Asia, and Oceania	Sub-Saharan Africa
**0 to 14**							
Drowning	23.03%	10.98%	13.07%	13.36%	26.48%	34.70%	20.43%
Fire, heat, and hot substances	17.94%	10.61%	7.00%	10.51%	6.90%	5.89%	14.97%
Venomous animal contact	0.42%	0.37%	1.44%	2.36%	20.51%	2.78%	4.63%
Falls	8.65%	3.73%	3.69%	13.67%	14.29%	10.95%	5.44%
Pedestrian road injuries	5.83%	6.20%	10.22%	11.36%	7.35%	10.63%	10.35%
Physical violence by other means	13.57%	31.59%	26.03%	8.88%	8.08%	6.97%	9.01%
Pulmonary aspiration and foreign body in airway	12.01%	16.56%	16.57%	7.49%	3.01%	10.50%	7.98%
Motor vehicle road injuries	3.83%	5.51%	3.83%	10.96%	1.53%	2.60%	5.71%
Other unintentional injuries	4.65%	2.31%	3.48%	8.45%	2.59%	3.10%	5.04%
Other exposure to mechanical forces	1.18%	1.13%	1.05%	3.10%	1.46%	2.76%	3.35%
Poisoning by other means	1.14%	1.02%	1.21%	1.32%	1.56%	1.18%	3.22%
Adverse effects of medical treatment	0.74%	0.91%	1.08%	1.10%	0.91%	0.95%	1.77%
Motorcyclist road injuries	0.54%	0.88%	1.44%	2.40%	0.96%	1.90%	1.16%
Environmental heat and cold exposure	0.81%	0.46%	0.35%	0.44%	1.25%	0.28%	1.05%
Other transport injuries	0.55%	1.07%	0.68%	0.72%	0.82%	0.74%	0.98%
Cyclist road injuries	0.55%	1.29%	0.96%	0.46%	0.80%	0.99%	0.73%
Non-venomous animal contact	0.29%	0.29%	0.25%	0.32%	0.41%	0.40%	1.08%
Physical violence by firearm	0.14%	2.48%	4.65%	0.83%	0.15%	0.39%	0.78%
Poisoning by carbon monoxide	2.44%	0.84%	0.26%	1.04%	0.20%	0.81%	0.38%
Physical violence by sharp object	0.43%	0.60%	0.98%	0.24%	0.13%	0.49%	0.53%
**15-49 years**							
Self-harm by other specified means	26.03%	34.58%	6.87%	11.42%	25.29%	15.62%	13.92%
Physical violence by other means	24.06%	7.56%	32.82%	17.98%	11.07%	7.56%	23.23%
Pedestrian road injuries	3.86%	3.90%	4.95%	9.59%	7.14%	13.61%	9.99%
Falls	6.78%	5.31%	2.74%	6.21%	7.84%	11.60%	3.90%
Motor vehicle road injuries	5.72%	9.12%	4.42%	15.16%	3.96%	7.21%	10.30%
Motorcyclist road injuries	1.38%	4.36%	5.35%	5.38%	8.11%	8.76%	2.35%
Physical violence by firearm	0.98%	8.64%	24.80%	3.95%	1.35%	2.98%	5.56%
Drowning	6.03%	3.17%	3.27%	3.85%	5.31%	7.75%	3.96%
Fire, heat, and hot substances	6.11%	3.17%	1.23%	7.34%	5.69%	2.70%	5.11%
Venomous animal contact	0.27%	0.19%	0.29%	0.33%	9.29%	1.16%	1.89%
Other unintentional injuries	3.22%	1.53%	2.16%	6.49%	5.10%	6.55%	2.73%
Physical violence by sharp object	3.14%	1.88%	4.42%	1.42%	1.06%	2.37%	4.26%
Other exposure to mechanical forces	0.96%	0.90%	0.53%	3.44%	1.10%	3.01%	1.97%
Other transport injuries	0.89%	1.14%	0.60%	1.18%	1.84%	1.48%	1.61%
Self-harm by firearm	0.90%	8.77%	0.88%	0.80%	1.60%	0.40%	1.44%
Cyclist road injuries	0.38%	0.72%	0.66%	0.40%	1.61%	1.76%	1.14%
Environmental heat and cold exposure	3.49%	0.50%	0.16%	0.36%	0.85%	0.30%	1.12%
Pulmonary aspiration and foreign body in airway	2.22%	1.95%	1.03%	0.89%	0.14%	0.83%	1.10%
Adverse effects of medical treatment	0.25%	0.38%	0.22%	0.73%	0.64%	0.55%	0.89%
Poisoning by other means	0.27%	0.39%	0.26%	0.50%	0.32%	1.41%	0.94%
**50 to 59**							
Falls	10.59%	14.50%	10.18%	11.46%	20.04%	19.97%	10.65%
Self-harm by other specified means	21.32%	33.95%	8.80%	10.82%	15.83%	15.08%	17.74%
Pedestrian road injuries	3.89%	4.33%	9.98%	14.53%	9.55%	17.22%	11.90%
Physical violence by other means	21.98%	6.44%	23.86%	9.00%	6.08%	4.80%	11.03%
Venomous animal contact	0.32%	0.35%	0.83%	0.49%	10.93%	2.00%	5.12%
Motor vehicle road injuries	3.21%	5.63%	5.36%	19.59%	4.03%	5.27%	9.24%
Fire, heat, and hot substances	9.26%	5.05%	2.63%	8.20%	4.06%	2.72%	7.44%
Drowning	4.96%	3.01%	4.02%	2.51%	5.16%	5.93%	3.31%
Motorcyclist road injuries	0.50%	2.40%	4.59%	2.98%	5.70%	5.64%	1.72%
Other unintentional injuries	2.79%	1.06%	2.86%	3.90%	4.69%	5.03%	2.40%
Physical violence by firearm	0.41%	2.45%	12.47%	1.76%	0.96%	1.61%	3.05%
Other exposure to mechanical forces	1.15%	1.26%	1.07%	3.83%	1.42%	3.33%	3.17%

Environmental heat and cold exposure	7.09%	1.05%	0.52%	0.78%	2.51%	0.41%	1.16%
Cyclist road injuries	0.55%	1.01%	1.40%	0.76%	2.05%	2.72%	1.33%
Other transport injuries	0.71%	1.02%	0.87%	1.69%	2.31%	1.33%	1.07%
Physical violence by sharp object	2.11%	0.92%	3.57%	0.61%	0.83%	1.16%	1.90%
Self-harm by firearm	0.97%	9.76%	1.07%	0.89%	0.85%	0.28%	1.60%
Pulmonary aspiration and foreign body in airway	3.71%	3.23%	2.39%	1.53%	0.46%	1.07%	1.29%
Adverse effects of medical treatment	0.39%	0.84%	0.70%	1.25%	1.35%	0.58%	1.02%
Poisoning by other means	0.34%	0.39%	0.36%	0.50%	0.26%	1.55%	1.38%
**60 to 69**							
Falls	15.40%	24.36%	19.04%	18.02%	37.87%	27.04%	16.41%
Self-harm by other specified means	18.85%	25.01%	7.99%	9.42%	9.46%	16.59%	16.18%
Pedestrian road injuries	4.30%	4.73%	12.79%	17.09%	9.27%	17.25%	12.14%
Venomous animal contact	0.33%	0.35%	1.21%	0.57%	10.72%	1.86%	6.24%
Physical violence by other means	19.93%	5.30%	18.85%	5.97%	3.88%	3.92%	8.48%
Fire, heat, and hot substances	10.73%	6.67%	3.80%	8.35%	3.87%	3.42%	9.65%
Drowning	4.42%	3.97%	4.18%	2.22%	4.34%	6.27%	3.45%
Motor vehicle road injuries	2.57%	5.09%	4.89%	17.91%	2.61%	3.42%	8.15%
Other unintentional injuries	2.85%	0.86%	2.42%	2.84%	2.87%	3.29%	2.32%
Motorcyclist road injuries	0.29%	1.49%	2.05%	2.57%	2.85%	2.91%	1.49%
Environmental heat and cold exposure	6.59%	1.22%	0.53%	0.61%	2.53%	0.47%	1.22%
Adverse effects of medical treatment	0.63%	1.59%	1.34%	1.89%	2.51%	0.71%	1.44%
Cyclist road injuries	0.69%	1.25%	1.53%	0.70%	1.76%	2.81%	1.41%
Other exposure to mechanical forces	0.96%	1.24%	1.05%	3.26%	1.25%	2.39%	1.62%
Pulmonary aspiration and foreign body in airway	4.08%	4.63%	3.29%	1.88%	0.56%	1.28%	1.33%
Other transport injuries	0.67%	0.93%	0.75%	1.67%	1.39%	0.97%	0.66%
Physical violence by firearm	0.28%	1.36%	7.89%	1.03%	0.45%	0.68%	1.43%
Self-harm by firearm	0.91%	7.83%	1.07%	0.84%	0.47%	0.18%	1.50%
Physical violence by sharp object	1.46%	0.63%	2.66%	0.39%	0.51%	0.67%	1.45%
Poisoning by other means	0.32%	0.32%	0.41%	0.43%	0.21%	1.64%	1.44%
**70 to 79**							
Falls	23.57%	41.13%	33.59%	30.90%	58.29%	41.23%	27.10%
Self-harm by other specified means	19.73%	15.96%	5.94%	6.74%	5.63%	14.93%	14.30%
Pedestrian road injuries	5.55%	4.83%	13.47%	15.70%	6.20%	13.63%	10.58%
Venomous animal contact	0.26%	0.24%	1.52%	0.67%	8.48%	2.16%	5.75%
Fire, heat, and hot substances	12.26%	7.48%	5.56%	9.73%	3.61%	4.35%	11.99%
Physical violence by other means	16.40%	4.19%	13.55%	4.47%	2.65%	3.10%	4.78%
Drowning	3.16%	4.33%	3.10%	1.72%	3.31%	6.22%	3.52%
Motor vehicle road injuries	2.08%	3.68%	3.54%	13.77%	1.47%	2.45%	7.13%
Environmental heat and cold exposure	4.05%	1.27%	0.59%	0.88%	2.67%	0.58%	1.84%
Adverse effects of medical treatment	1.02%	2.06%	1.97%	2.59%	2.23%	0.81%	1.43%
Pulmonary aspiration and foreign body in airway	2.58%	5.72%	4.14%	1.95%	0.85%	1.99%	1.73%
Other unintentional injuries	2.09%	0.51%	1.92%	2.07%	1.24%	1.50%	1.85%
Other exposure to mechanical forces	0.80%	0.92%	0.93%	3.07%	0.89%	1.97%	1.35%
Other transport injuries	0.73%	0.62%	0.59%	1.24%	0.82%	0.72%	0.41%
Self-harm by firearm	0.81%	4.89%	0.92%	0.80%	0.24%	0.14%	1.29%
Physical violence by firearm	0.20%	0.58%	4.40%	0.61%	0.37%	0.30%	0.68%
Physical violence by sharp object	1.03%	0.39%	1.83%	0.35%	0.26%	0.34%	1.12%
Poisoning by other means	0.30%	0.28%	0.41%	0.45%	0.10%	1.34%	1.13%
Poisoning by carbon monoxide	2.55%	0.35%	0.21%	0.92%	0.10%	1.24%	0.47%
Other road injuries	0.19%	0.12%	0.61%	0.26%	0.27%	0.31%	0.28%
**80 to 89**							
Falls	41.74%	61.46%	55.63%	49.47%	69.20%	61.19%	45.45%
Self-harm by other specified means	16.32%	7.19%	3.37%	5.77%	4.04%	9.68%	10.18%
Fire, heat, and hot substances	14.41%	8.04%	7.08%	10.76%	3.57%	5.15%	11.79%
Pedestrian road injuries	4.29%	3.28%	9.52%	10.82%	4.39%	7.20%	7.64%
Venomous animal contact	0.24%	0.14%	1.45%	0.74%	5.00%	1.33%	3.62%
Drowning	1.99%	2.72%	1.66%	1.10%	2.87%	4.89%	2.60%
Pulmonary aspiration and foreign body in airway	1.82%	6.91%	4.69%	1.78%	1.23%	2.06%	1.99%
Motor vehicle road injuries	1.36%	1.96%	1.96%	7.75%	0.91%	1.09%	4.72%
Adverse effects of medical treatment	1.25%	1.80%	2.12%	2.54%	2.09%	0.68%	1.65%

Environmental heat and cold exposure	2.52%	1.33%	0.59%	0.71%	2.22%	0.78%	1.20%
Other unintentional injuries	1.67%	0.32%	1.78%	1.57%	1.51%	1.00%	1.82%
Physical violence by other means	6.63%	0.71%	5.22%	0.84%	0.64%	0.45%	1.67%
Other exposure to mechanical forces	0.81%	0.71%	0.69%	2.17%	0.62%	1.23%	1.56%
Self-harm by firearm	0.55%	2.20%	0.67%	0.79%	0.20%	0.11%	0.96%
Other transport injuries	0.52%	0.21%	0.34%	0.64%	0.66%	0.40%	0.24%
Poisoning by carbon monoxide	2.30%	0.20%	0.17%	0.68%	0.12%	0.94%	0.28%
Poisoning by other means	0.29%	0.24%	0.33%	0.41%	0.09%	0.87%	0.82%
Other road injuries	0.12%	0.07%	0.40%	0.23%	0.25%	0.19%	0.28%
Unintentional firearm injuries	0.18%	0.14%	0.31%	0.37%	0.08%	0.09%	0.72%
Non-venomous animal contact	0.13%	0.07%	0.23%	0.25%	0.12%	0.21%	0.42%
**90 plus**							
Falls	70.11%	75.05%	69.92%	63.94%	79.97%	79.70%	63.74%
Fire, heat, and hot substances	13.54%	8.55%	8.02%	10.49%	2.71%	4.71%	10.38%
Pulmonary aspiration and foreign body in airway	1.99%	7.81%	5.13%	2.17%	1.64%	2.41%	2.82%
Pedestrian road injuries	2.41%	1.80%	5.13%	6.36%	2.63%	3.36%	4.78%
Drowning	1.40%	1.08%	1.42%	0.86%	1.65%	2.74%	1.84%
Venomous animal contact	0.29%	0.11%	1.62%	1.05%	3.10%	0.85%	2.36%
Adverse effects of medical treatment	1.51%	1.51%	1.91%	2.39%	2.20%	0.58%	2.03%
Other unintentional injuries	1.76%	0.28%	1.94%	2.08%	1.68%	1.11%	2.53%
Environmental heat and cold exposure	2.04%	1.27%	0.71%	0.88%	1.65%	0.85%	1.66%
Motor vehicle road injuries	1.07%	1.11%	1.29%	4.94%	1.05%	0.75%	3.06%
Other exposure to mechanical forces	0.96%	0.72%	0.69%	1.96%	0.55%	0.99%	1.89%
Other transport injuries	0.30%	0.08%	0.25%	0.52%	0.49%	0.28%	0.18%
Unintentional firearm injuries	0.21%	0.13%	0.39%	0.39%	0.09%	0.13%	1.25%
Poisoning by carbon monoxide	1.64%	0.10%	0.18%	0.55%	0.11%	0.44%	0.23%
Poisoning by other means	0.26%	0.17%	0.30%	0.36%	0.06%	0.42%	0.50%
Foreign body in other body part	0.32%	0.12%	0.54%	0.28%	0.11%	0.41%	0.13%
Non-venomous animal contact	0.11%	0.06%	0.22%	0.46%	0.09%	0.16%	0.42%
Other road injuries	0.07%	0.05%	0.19%	0.16%	0.20%	0.10%	0.20%
Police conflict and executions	0.03%	0.00%	0.13%	0.16%	0.01%	0.02%	0.01%

Proportions in the above table are unique by super region and age group. For each individual percentage in the table, the denominator used in its calculation was the total deaths redistributed across all causes within its respective super region and age group. Thus, column percentages within each age group will add to, or very close to, 100%. Only the top 20 redistribution targets are listed, so it is possible that some columns may not quite total 100%.

Appendix Figure 15

Accidental Poisoning Redistribution Proportions, Year Group 2003 - 2017, By Drug Use Disorder and Age Group					
	Super Region				
**Cause and Age Group**	**Latin America and Caribbean**	**High-income**	**Southeast Asia, East Asia, and Oceania**	**Central Europe, Eastern Europe, and Central Asia**	**North Africa and Middle East**
Alcohol use disorders					
15 to 29	2.46%	2.30%	4.93%	0.25%	0.00%
30 to 49	11.20%	3.91%	4.60%	0.72%	0.00%
50-69 years	18.54%	5.26%	6.63%	2.22%	0.00%
70 to 79	46.67%	7.85%	0.00%	0.00%	0.00%
80 plus	55.56%	4.52%	0.00%	50.00%	0.00%
Cocaine use disorders					
15 to 29	63.01%	4.80%	0.00%	1.98%	2.78%
30 to 49	47.50%	10.25%	0.00%	2.59%	0.00%
50-69 years	33.71%	12.72%	0.00%	4.44%	50.00%
70 to 79	33.33%	11.11%	0.00%	0.00%	0.00%
80 plus	22.22%	2.51%	0.00%	0.00%	0.00%
Other drug use disorders					
15 to 29	15.82%	6.50%	29.23%	9.14%	2.78%
30 to 49	10.65%	6.22%	24.14%	7.90%	0.00%
50-69 years	11.24%	5.37%	20.48%	4.44%	0.00%
70 to 79	0.00%	6.37%	0.00%	0.00%	0.00%
80 plus	22.22%	12.55%	0.00%	0.00%	0.00%
Opioid use disorders					
0 to 14	0.00%	100.00%	0.00%	0.00%	0.00%
15 to 29	12.57%	82.21%	29.23%	80.00%	88.89%
30 to 49	21.96%	73.88%	56.31%	84.05%	100.00%
50-69 years	25.84%	69.94%	58.43%	88.89%	25.00%
70 to 79	20.00%	70.13%	100.00%	100.00%	0.00%
80 plus	0.00%	79.05%	50.00%	50.00%	0.00%
Amphetamine use disorders					
15 to 29	6.15%	4.19%	36.62%	8.64%	5.56%
30 to 49	8.70%	5.74%	14.95%	4.74%	0.00%
50-69 years	10.67%	6.71%	14.46%	0.00%	25.00%
70 to 79	0.00%	4.55%	0.00%	0.00%	0.00%
80 plus	0.00%	1.38%	50.00%	0.00%	0.00%

Multiple cause data is also used to adjust for other types of miscoding where there is evidence that observed mortality trends for a given cause are not reflective of additional epidemiological knowledge. Here, we show an example of proportions generated from multiple cause data that we used to adjust deaths due to drug overdoses that were misclassified as accidental poisoning.

Appendix Figure 16

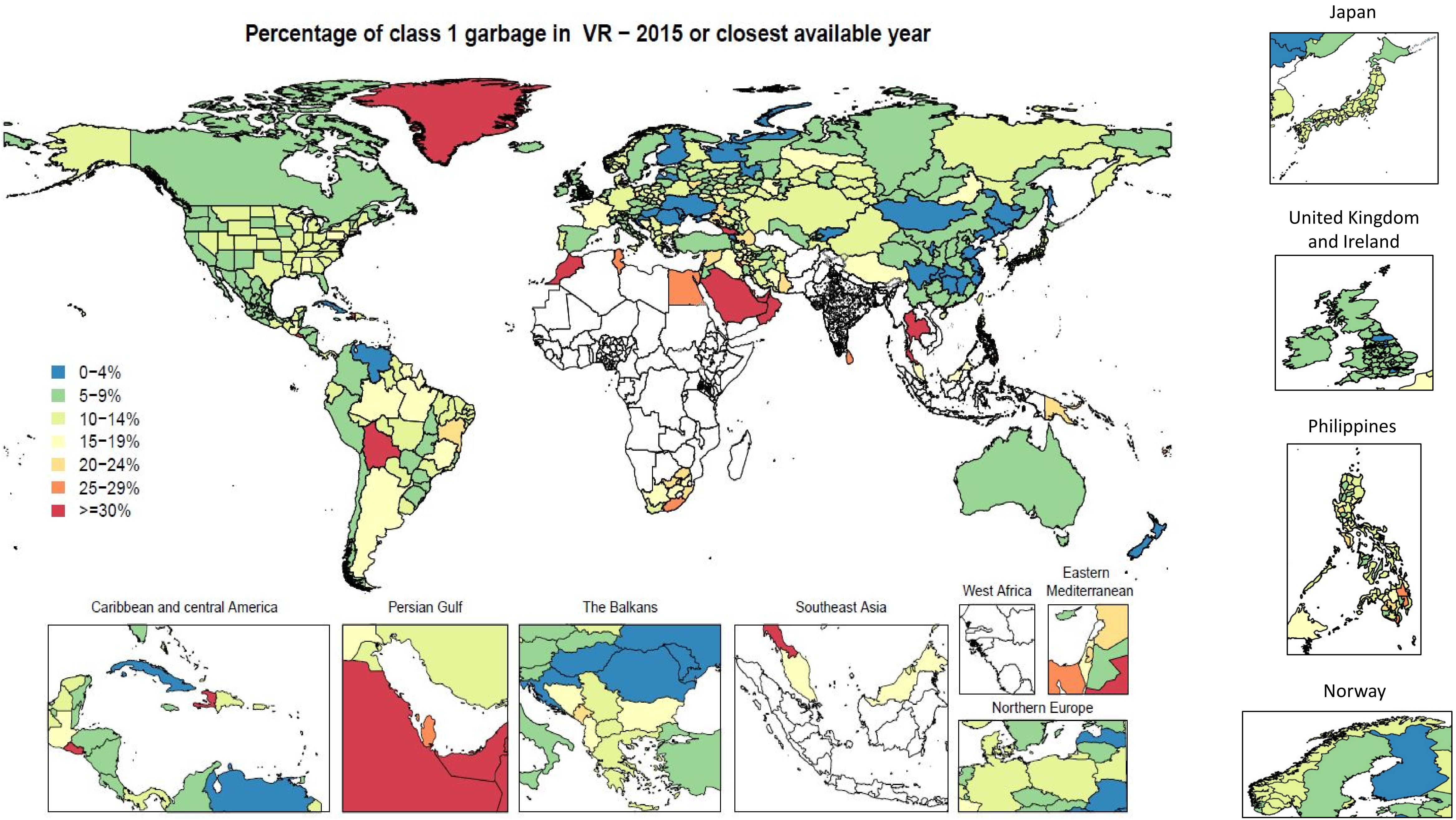

Percentage of class 2 garbage in VR − 2015 or closest available year

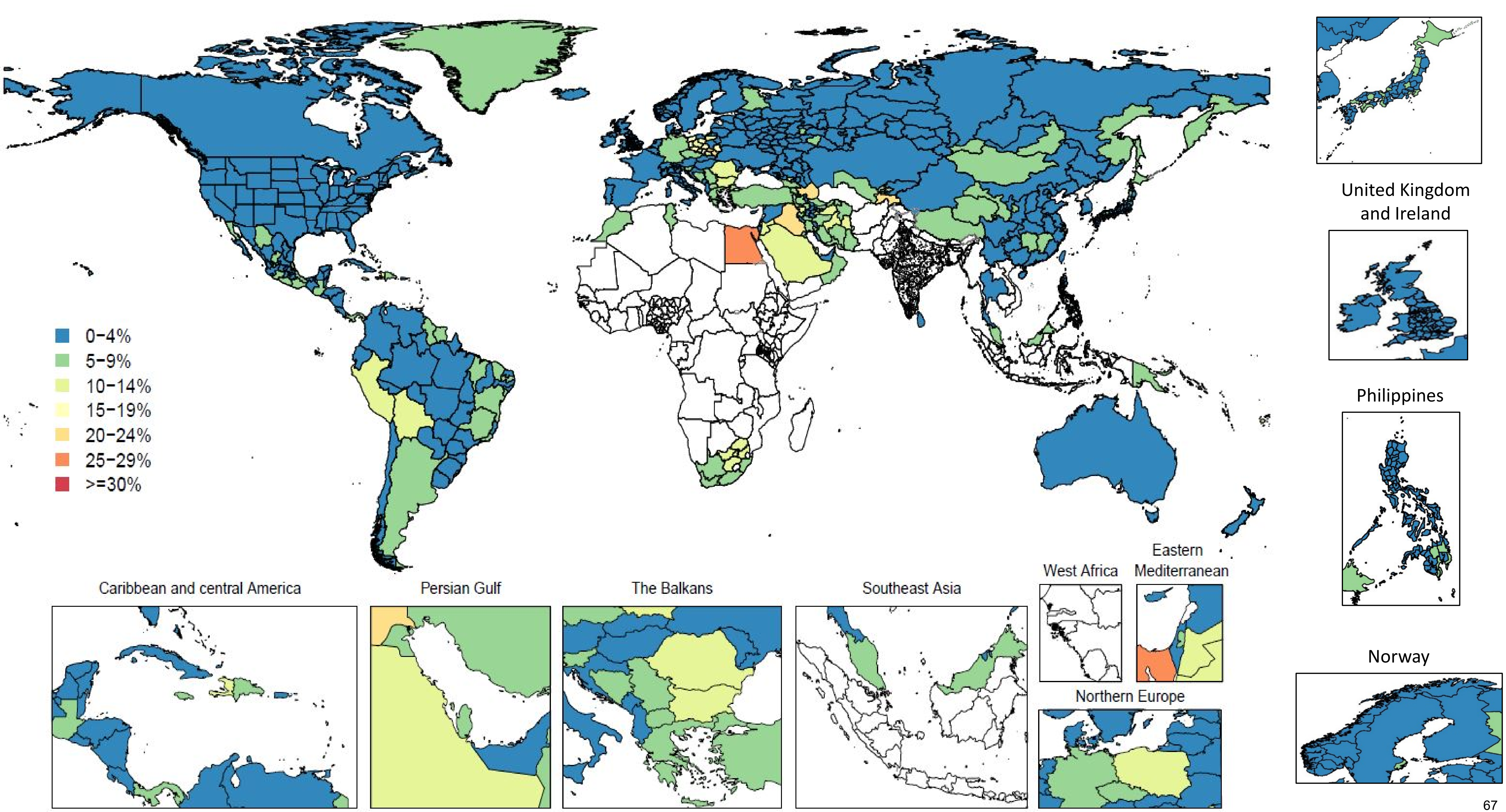

Percentage of class 3 garbage in VR − 2015 or closest available year

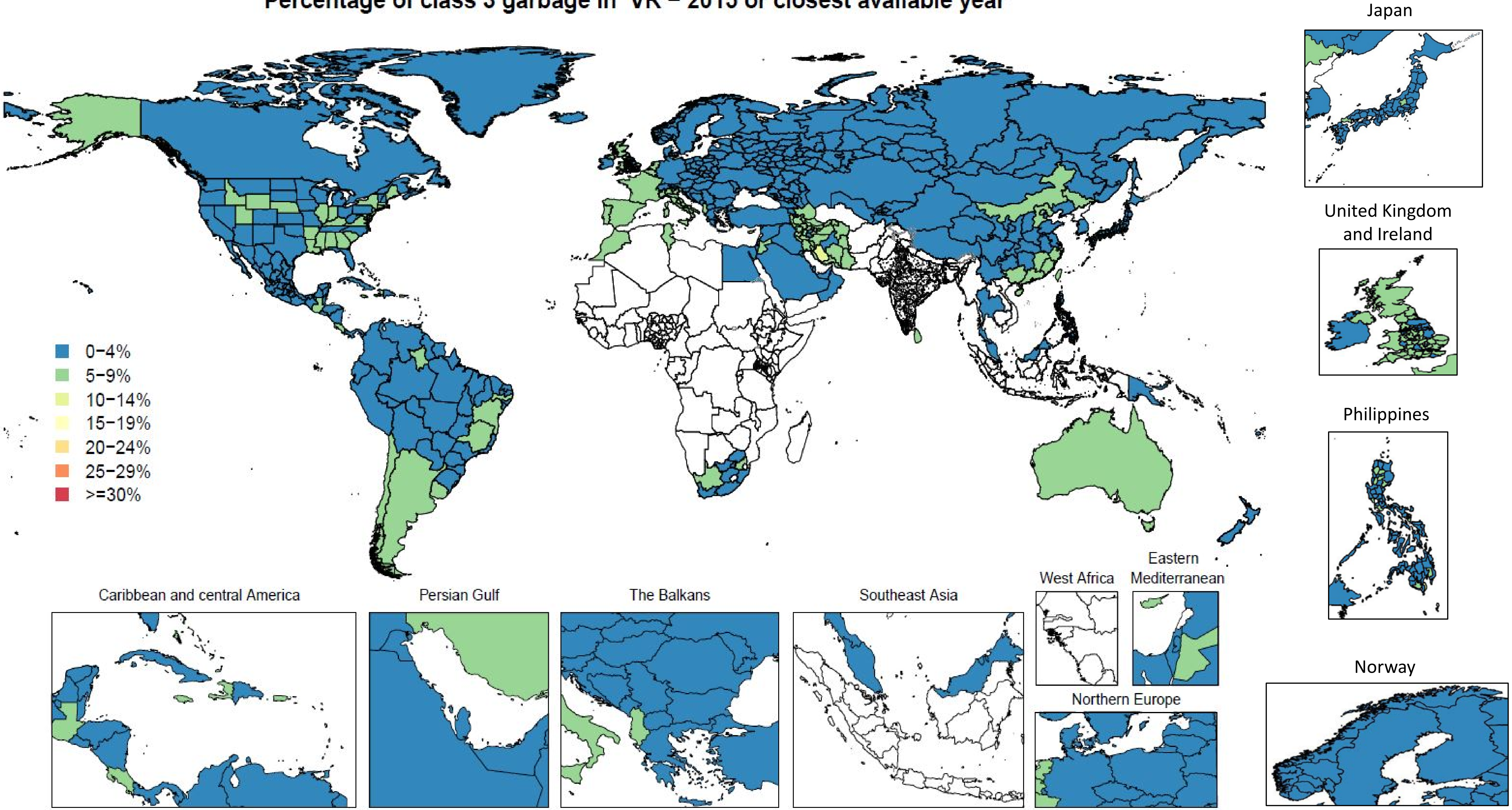

Percentage of class 4 garbage in VR – 2015 or closest available year

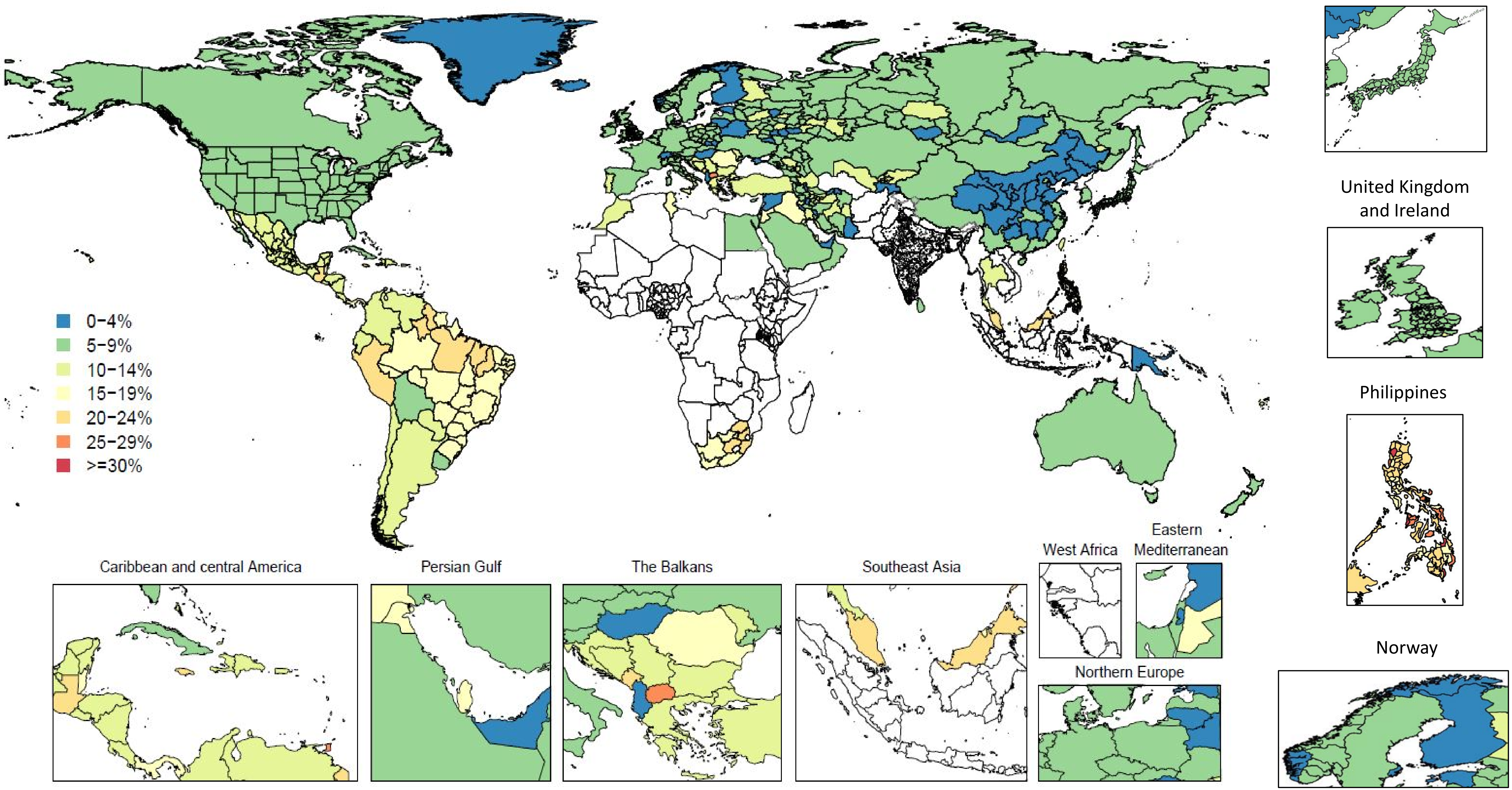

Appendix Figure 17

Top 5 ICD10 garbage codes by country - 2015 or most recent year

Country	Garbage code	Garbage class	Deaths	Percentage of all garbage deaths	Percentage of total deaths
American Samoa	Sepsis (Non- maternal and neonatal sepsis)	1	10	9.41%	3.58%
	Fluid, Electrolyte, Acid Base Disorders	1	5	4.60%	1.75%
	Unspecified Site Cancer	3	4	4.32%	1.65%
	All, Ill Defined code for causes of death	1	3	3.07%	1.17%
	Gastrointestinal Bleeding	2	3	3.45%	1.31%
Antigua and Barbuda	Hypertension	2	26	10.50%	5.01%
	Sepsis (Non- maternal and neonatal sepsis)	1	24	9.82%	4.68%
	Exposure to unspecified factor X59	2	14	5.94%	2.83%
	Left heart failure	1	10	4.05%	1.93%
	Fluid, Electrolyte, Acid Base Disorders	1	9	3.90%	1.86%
Argentina	Heart failure unspecified right or left	2	21214	13.06%	6.42%
	All, Ill Defined code for causes of death	1	12822	7.89%	3.88%
	Sepsis (Non- maternal and neonatal sepsis)	1	9595	5.91%	2.90%
	Acute Respiratory Failure	1	7511	4.62%	2.27%
	Left heart failure	1	4871	3.00%	1.47%
Armenia	Atherosclerosis	2	405	8.51%	1.46%
	External Causes UDI, type unspecified	2	393	8.24%	1.41%
	All, Ill Defined code for causes of death	1	259	5.43%	0.93%
	right heart failure and pulmonary heart disease	2	211	4.43%	0.76%
	Senility	1	188	3.96%	0.68%
Australia	Unspecified Site Cancer	3	2596	6.94%	1.63%
	Sepsis (Non- maternal and neonatal sepsis)	1	1790	4.78%	1.13%
	Left heart failure	1	1651	4.41%	1.04%
	All, Ill Defined code for causes of death	1	1628	4.35%	1.02%
	Unspecified GI Cancer	3	1310	3.50%	0.82%
Austria	Heart failure unspecified right or left	2	2169	11.66%	2.61%
	Unspecified Site Cancer	3	1475	7.93%	1.78%
	Unspecified Heart Diseases	3	1185	6.37%	1.43%
	All, Ill Defined code for causes of death	1	1158	6.23%	1.39%
	Exposure to unspecified factor X59	2	818	4.40%	0.98%
Azerbaijan	Heart failure unspecified right or left	2	7832	25.51%	15.53%
	Shock, Cardiac Arrest, Coma	1	4729	15.40%	9.38%
	Hepatic Failure	1	2085	6.79%	4.13%
	External Causes UDI, type unspecified	2	1876	6.11%	3.72%
	Unspecified Site Cancer	3	1537	5.01%	3.05%
Bahamas	Sepsis (Non- maternal and neonatal sepsis)	1	58	8.67%	2.70%
	Hypertension	2	40	6.01%	1.87%

Top 5 ICD10 garbage codes by country - 2015 or most recent year

Country	Garbage code	Garbage class	Deaths	Percentage of all garbage deaths	Percentage of total deaths
Bahamas	Unspecified Site Cancer	3	28	4.31%	1.34%
	Fluid, Electrolyte, Acid Base Disorders	1	25	3.84%	1.20%
	Unspecified Heart Diseases	3	23	3.51%	1.09%
Bahrain	Shock, Cardiac Arrest, Coma	1	382	32.33%	14.18%
	Hypertension	2	159	13.50%	5.92%
	All, Ill Defined code for causes of death	1	127	10.76%	4.72%
	Senility	1	111	9.47%	4.16%
	Undetermined intent Strangulation	2	29	2.54%	1.11%
Barbados	Sepsis (Non- maternal and neonatal sepsis)	1	96	8.50%	3.87%
	Unspecified Site Cancer	3	54	4.85%	2.21%
	Pulmonary Embolism	1	48	4.26%	1.94%
	Hypertension	2	41	3.69%	1.68%
	All, Ill Defined code for causes of death	1	31	2.80%	1.27%
Belgium	Heart failure unspecified right or left	2	4221	10.90%	3.82%
	All, Ill Defined code for causes of death	1	3395	8.77%	3.07%
	Shock, Cardiac Arrest, Coma	1	2360	6.09%	2.14%
	Senility	1	1414	3.65%	1.28%
	Left heart failure	1	1400	3.62%	1.27%
Belize	Left heart failure	1	30	5.35%	1.74%
	Hypertension	2	25	4.47%	1.45%
	Sepsis (Non- maternal and neonatal sepsis)	1	21	3.78%	1.23%
	Pneumonitis	1	16	2.86%	0.93%
	Primary or secondary Liver Cancer Unspecified	3	15	2.61%	0.85%
Bermuda	Heart failure unspecified right or left	2	9	7.30%	1.93%
	Unspecified Site Cancer	3	8	7.28%	1.93%
	Hypertension	2	7	6.26%	1.66%
	Sepsis (Non- maternal and neonatal sepsis)	1	7	6.29%	1.66%
	Pneumonitis	1	4	3.72%	0.98%
Bolivia (Plurinational State of)	All, Ill Defined code for causes of death	1	9005	53.21%	40.07%
	External Causes UDI, type unspecified	2	2159	12.76%	9.61%
	Sepsis (Non- maternal and neonatal sepsis)	1	593	3.50%	2.64%
	Acute Respiratory Failure	1	428	2.53%	1.91%
	Heart failure unspecified right or left	2	407	2.40%	1.81%
Bosnia and Herzegovina	All, Ill Defined code for causes of death	1	2245	15.51%	6.24%
	Shock, Cardiac Arrest, Coma	1	2167	14.97%	6.02%
	Heart failure unspecified right or left	2	1739	12.01%	4.83%
	Hypertension	2	1171	8.09%	3.25%

Top 5 ICD10 garbage codes by country - 2015 or most recent year

Country	Garbage code	Garbage class	Deaths	Percentage of all garbage deaths	Percentage of total deaths
Herzegovina	Atherosclerosis	2	434	3.00%	1.21%
Brazil	All, Ill Defined code for causes of death	1	56582	11.86%	4.49%
	Hypertension	2	21967	4.60%	1.74%
	Sepsis (Non- maternal and neonatal sepsis)	1	19083	4.00%	1.51%
	Heart failure unspecified right or left	2	14853	3.11%	1.18%
	Left heart failure	1	14292	3.00%	1.13%
Brunei Darussalam	All, Ill Defined code for causes of death	1	51	8.50%	3.31%
	Sepsis (Non- maternal and neonatal sepsis)	1	51	8.57%	3.34%
	Senility	1	25	4.17%	1.63%
	Exposure to unspecified factor X59	2	16	2.81%	1.09%
	Hypertension	2	16	2.76%	1.07%
Bulgaria	Left heart failure	1	11458	23.37%	10.41%
	Heart failure unspecified right or left	2	8666	17.67%	7.87%
	Hypertension	2	2258	4.61%	2.05%
	Shock, Cardiac Arrest, Coma	1	2161	4.41%	1.96%
	Atherosclerosis	2	2126	4.34%	1.93%
Cabo Verde	All, Ill Defined code for causes of death	1	168	12.58%	6.09%
	External Causes UDI, type unspecified	2	121	9.08%	4.40%
	Heart failure unspecified right or left	2	115	8.59%	4.16%
	Senility	1	89	6.67%	3.23%
	Sepsis (Non- maternal and neonatal sepsis)	1	73	5.52%	2.67%
Canada	All, Ill Defined code for causes of death	1	3265	4.89%	1.24%
	Unspecified Site Cancer	3	3227	4.84%	1.22%
	Left heart failure	1	3016	4.52%	1.14%
	Sepsis (Non- maternal and neonatal sepsis)	1	2526	3.79%	0.96%
	Undetermined intent Poisoning by multiple or unspecified drug	1	2190	3.28%	0.83%
Chile	Hypertension	2	2084	7.37%	2.02%
	All, Ill Defined code for causes of death	1	1859	6.57%	1.80%
	Alcoholic hepatic failure	3	1020	3.61%	0.99%
	Unspecified Site Cancer	3	986	3.49%	0.95%
	Heart failure unspecified right or left	2	882	3.12%	0.85%
China	Primary or secondary Liver Cancer Unspecified	3	135451	15.37%	2.24%
	Hypertension	2	83852	9.52%	1.39%
	Senility	1	38607	4.38%	0.64%
	Shock, Cardiac Arrest, Coma	1	35266	4.00%	0.58%
	right heart failure and pulmonary heart disease	2	34077	3.87%	0.56%
Colombia	All, Ill Defined code for causes of death	1	2840	6.03%	1.29%

Top 5 ICD10 garbage codes by country - 2015 or most recent year

Country	Garbage code	Garbage class	Deaths	Percentage of all garbage deaths	Percentage of total deaths
Colombia	Hypertension	2	2251	4.78%	1.03%
	Unspecified Site Cancer	3	1989	4.23%	0.91%
	Heart failure unspecified right or left	2	1333	2.83%	0.61%
	External Causes UDI, type unspecified	2	1033	2.19%	0.47%
Costa Rica	Unspecified Site Cancer	3	356	6.43%	1.70%
	All, Ill Defined code for causes of death	1	290	5.24%	1.38%
	Exposure to unspecified factor X59	2	279	5.04%	1.33%
	Alcoholic hepatic failure	3	139	2.52%	0.67%
	Unspecified chronic respiratory diseases	3	119	2.16%	0.57%
Croatia	Heart failure unspecified right or left	2	1298	9.97%	2.39%
	Atherosclerosis	2	461	3.55%	0.85%
	Unspecified Site Cancer	3	431	3.32%	0.80%
	Unspecified Mental/Brain Disorders	3	340	2.62%	0.63%
	Shock, Cardiac Arrest, Coma	1	297	2.28%	0.55%
Cuba	Atherosclerosis	2	1595	7.04%	1.60%
	External Causes UDI, type unspecified	2	1371	6.06%	1.38%
	Left heart failure	1	1177	5.20%	1.18%
	All, Ill Defined code for causes of death	1	1138	5.03%	1.14%
	Hypertension	2	668	2.95%	0.67%
Cyprus	Heart failure unspecified right or left	2	253	10.94%	4.23%
	Unspecified chronic respiratory diseases	3	233	10.11%	3.90%
	Shock, Cardiac Arrest, Coma	1	142	6.17%	2.38%
	Senility	1	101	4.40%	1.70%
	Sepsis (Non- maternal and neonatal sepsis)	1	93	4.03%	1.55%
Czechia	Heart failure unspecified right or left	2	2464	10.04%	2.22%
	Exposure to unspecified factor X59	2	1469	5.99%	1.32%
	Atherosclerosis	2	1434	5.84%	1.29%
	Sepsis (Non- maternal and neonatal sepsis)	1	1367	5.57%	1.23%
	Left heart failure	1	1314	5.35%	1.18%
Denmark	All, Ill Defined code for causes of death	1	3294	21.28%	6.31%
	Senility	1	822	5.31%	1.58%
	Unspecified Site Cancer	3	722	4.67%	1.38%
	Heart failure unspecified right or left	2	714	4.61%	1.37%
	Hypertension	2	459	2.97%	0.88%
Dominica	Hypertension	2	29	9.50%	4.40%
	All, Ill Defined code for causes of death	1	20	6.84%	3.17%
	Shock, Cardiac Arrest, Coma	1	14	4.85%	2.24%

Top 5 ICD10 garbage codes by country - 2015 or most recent year

Country	Garbage code	Garbage class	Deaths	Percentage of all garbage deaths	Percentage of total deaths
Dominica	Left heart failure	1	13	4.29%	1.99%
	Pulmonary Embolism	1	12	4.15%	1.92%
Dominican Republic	All, Ill Defined code for causes of death	1	1827	19.72%	6.99%
	Heart failure unspecified right or left	2	548	5.91%	2.10%
	External Causes UDI, type unspecified	2	446	4.82%	1.71%
	Hypertension	2	422	4.56%	1.62%
	Left heart failure	1	309	3.34%	1.18%
Ecuador	All, Ill Defined code for causes of death	1	3102	13.91%	4.79%
	Hypertension	2	1003	4.50%	1.55%
	Heart failure unspecified right or left	2	665	2.98%	1.03%
	Senility	1	578	2.59%	0.89%
	Shock, Cardiac Arrest, Coma	1	513	2.30%	0.79%
Egypt	Heart failure unspecified right or left	2	75104	20.06%	13.09%
	Hypertension	2	42179	11.27%	7.35%
	Shock, Cardiac Arrest, Coma	1	39828	10.64%	6.94%
	All, Ill Defined code for causes of death	1	28811	7.70%	5.02%
	Hepatic Failure	1	27214	7.27%	4.74%
El Salvador	Shock, Cardiac Arrest, Coma	1	4253	20.90%	10.41%
	All, Ill Defined code for causes of death	1	3184	15.65%	7.79%
	Sepsis (Non- maternal and neonatal sepsis)	1	1660	8.16%	4.06%
	Senility	1	1115	5.48%	2.73%
	Acute kidney failure	1	731	3.59%	1.79%
Estonia	Heart failure unspecified right or left	2	380	20.51%	2.49%
	All, Ill Defined code for causes of death	1	203	10.97%	1.33%
	Unspecified Site Cancer	3	123	6.67%	0.81%
	Atherosclerosis	2	103	5.59%	0.68%
	Undetermined intent Poisoning by multiple or unspecified drug	1	87	4.75%	0.58%
Fiji	Hypertension	2	600	18.19%	9.27%
	Left heart failure	1	406	12.33%	6.28%
	Senility	1	350	10.61%	5.41%
	Sepsis (Non- maternal and neonatal sepsis)	1	241	7.32%	3.73%
	Undetermined intent Strangulation	2	97	2.97%	1.51%
Finland	All, Ill Defined code for causes of death	1	676	7.95%	1.29%
	Unspecified Site Cancer	3	387	4.56%	0.74%
	Undetermined intent Poisoning by multiple or unspecified drug	1	225	2.66%	0.43%
	Unspecified Heart Diseases	3	191	2.26%	0.37%
	Unspecified GI Cancer	3	184	2.18%	0.35%

Top 5 ICD10 garbage codes by country - 2015 or most recent year

Country	Garbage code	Garbage class	Deaths	Percentage of all garbage deaths	Percentage of total deaths
France	All, Ill Defined code for causes of death	1	32660	15.86%	5.63%
	Shock, Cardiac Arrest, Coma	1	20851	10.13%	3.60%
	Heart failure unspecified right or left	2	15742	7.65%	2.71%
	Exposure to unspecified factor X59	2	9383	4.56%	1.62%
	Unspecified Site Cancer	3	7859	3.82%	1.36%
Georgia	All, Ill Defined code for causes of death	1	12222	48.18%	24.88%
	Heart failure unspecified right or left	2	1860	7.33%	3.79%
	Shock, Cardiac Arrest, Coma	1	1386	5.47%	2.82%
	Left heart failure	1	704	2.78%	1.43%
	Atherosclerosis	2	578	2.28%	1.18%
Germany	Heart failure unspecified right or left	2	39985	16.88%	4.32%
	All, Ill Defined code for causes of death	1	21544	9.10%	2.33%
	Unspecified Site Cancer	3	10799	4.56%	1.17%
	Sepsis (Non- maternal and neonatal sepsis)	1	9238	3.90%	1.00%
	Left heart failure	1	7505	3.17%	0.81%
Greece	Heart failure unspecified right or left	2	7752	15.49%	6.38%
	Shock, Cardiac Arrest, Coma	1	6185	12.36%	5.09%
	Sepsis (Non- maternal and neonatal sepsis)	1	3591	7.18%	2.96%
	Left heart failure	1	1944	3.88%	1.60%
	Unspecified Site Cancer	3	1623	3.24%	1.34%
Greenland	All, Ill Defined code for causes of death	1	64	30.37%	13.28%
	Senility	1	32	15.20%	6.65%
	Heart failure unspecified right or left	2	22	10.44%	4.56%
	Sepsis (Non- maternal and neonatal sepsis)	1	13	6.58%	2.88%
	Unspecified Site Cancer	3	9	4.74%	2.07%
Grenada	Atherosclerosis	2	20	5.37%	2.37%
	All, Ill Defined code for causes of death	1	16	4.29%	1.89%
	Left heart failure	1	16	4.19%	1.85%
	Hypertension	2	15	3.92%	1.73%
	Fluid, Electrolyte, Acid Base Disorders	1	12	3.24%	1.43%
Guam	Sepsis (Non- maternal and neonatal sepsis)	1	34	15.40%	3.52%
	Acute kidney failure	1	7	3.44%	0.79%
	Unspecified Site Cancer	3	7	3.57%	0.82%
	Pneumonitis	1	5	2.48%	0.57%
	Gastrointestinal Bleeding	2	4	2.21%	0.51%
Guatemala	All, Ill Defined code for causes of death	1	4574	11.89%	5.65%
	Exposure to unspecified factor X59	2	3032	7.88%	3.75%

Top 5 ICD10 garbage codes by country - 2015 or most recent year

Country	Garbage code	Garbage class	Deaths	Percentage of all garbage deaths	Percentage of total deaths
Guatemala	Senility	1	1438	3.74%	1.78%
	Acute kidney failure	1	1339	3.48%	1.65%
	Sepsis (Non- maternal and neonatal sepsis)	1	1209	3.14%	1.49%
Guyana	Hypertension	2	192	8.27%	3.46%
	Left heart failure	1	110	4.76%	1.99%
	External Causes UDI, type unspecified	2	108	4.68%	1.96%
	Sepsis (Non- maternal and neonatal sepsis)	1	102	4.42%	1.85%
	Heart failure unspecified right or left	2	77	3.31%	1.39%
Haiti	All, Ill Defined code for causes of death	1	672	44.40%	29.12%
	Shock, Cardiac Arrest, Coma	1	119	7.87%	5.16%
	Hypertension	2	96	6.34%	4.16%
	Sepsis (Non- maternal and neonatal sepsis)	1	72	4.81%	3.16%
	Unspecified Heart Diseases	3	53	3.52%	2.31%
Honduras	Sepsis (Non- maternal and neonatal sepsis)	1	89	5.62%	1.38%
	Exposure to unspecified factor X59	2	70	4.42%	1.09%
	Gastrointestinal Bleeding	2	56	3.52%	0.87%
	Left heart failure	1	41	2.63%	0.65%
	All, Ill Defined code for causes of death	1	38	2.40%	0.59%
Hungary	Atherosclerosis	2	2138	12.54%	1.62%
	Hypertension	2	1713	10.05%	1.30%
	Heart failure unspecified right or left	2	1208	7.09%	0.92%
	Left heart failure	1	943	5.54%	0.72%
	Primary or secondary Liver Cancer Unspecified	3	603	3.54%	0.46%
Iceland	Heart failure unspecified right or left	2	84	15.96%	3.85%
	All, Ill Defined code for causes of death	1	28	5.39%	1.30%
	Exposure to unspecified factor X59	2	24	4.68%	1.13%
	Acute kidney failure	1	14	2.82%	0.68%
	Unspecified Site Cancer	3	14	2.85%	0.69%
India	External Causes UDI, type unspecified	2	2815	17.98%	8.24%
	Shock, Cardiac Arrest, Coma	1	1588	10.15%	4.65%
	Sepsis (Non- maternal and neonatal sepsis)	1	1447	9.24%	4.24%
	Hypertension	2	1218	7.78%	3.57%
	All, Ill Defined code for causes of death	1	1190	7.60%	3.48%
Iran (Islamic Republic of)	Shock, Cardiac Arrest, Coma	1	10060	9.17%	3.12%
	All, Ill Defined code for causes of death	1	7043	6.42%	2.19%
	Hypertension	2	6740	6.15%	2.09%
	senility(CS-IRN)	1	5755	5.25%	1.79%

Top 5 ICD10 garbage codes by country - 2015 or most recent year

Country	Garbage code	Garbage class	Deaths	Percentage of all garbage deaths	Percentage of total deaths
Republic of)	right heart failure and pulmonary heart disease	2	5067	4.62%	1.57%
Iraq	Heart failure unspecified right or left	2	9490	16.53%	8.62%
	All, Ill Defined code for causes of death	1	8305	14.47%	7.55%
	Hypertension	2	5698	9.93%	5.18%
	Acute kidney failure	1	2857	4.98%	2.60%
	External Causes UDI, type unspecified	2	2732	4.76%	2.48%
Ireland	Left heart failure	1	356	5.11%	1.18%
	Unspecified Site Cancer	3	285	4.09%	0.95%
	Pneumonitis	1	256	3.67%	0.85%
	Sepsis (Non- maternal and neonatal sepsis)	1	216	3.10%	0.72%
	Undetermined intent Poisoning by multiple or unspecified drug	1	178	2.56%	0.59%
Israel	All, Ill Defined code for causes of death	1	2153	13.96%	4.86%
	Sepsis (Non- maternal and neonatal sepsis)	1	2027	13.14%	4.58%
	Acute kidney failure	1	874	5.67%	1.97%
	Left heart failure	1	583	3.78%	1.32%
	Hypertension	2	487	3.16%	1.10%
Italy	Unspecified Heart Diseases	3	11568	6.15%	1.79%
	Unspecified Site Cancer	3	9277	4.93%	1.44%
	Sepsis (Non- maternal and neonatal sepsis)	1	9242	4.91%	1.43%
	Exposure to unspecified factor X59	2	7440	3.95%	1.15%
	Heart failure unspecified right or left	2	5784	3.07%	0.90%
Jamaica	Hypertension	2	410	5.65%	2.24%
	Unspecified Site Cancer	3	256	3.53%	1.40%
	Sepsis (Non- maternal and neonatal sepsis)	1	207	2.85%	1.13%
	Undetermined intent shooting by unspecified firearm	2	199	2.75%	1.09%
	Left heart failure	1	116	1.60%	0.64%
Japan	Heart failure unspecified right or left	2	58911	15.68%	4.55%
	Senility	1	56838	15.13%	4.39%
	Pneumonitis	1	21702	5.78%	1.67%
	Left heart failure	1	12417	3.30%	0.96%
	Sepsis (Non- maternal and neonatal sepsis)	1	11335	3.02%	0.87%
Jordan	Heart failure unspecified right or left	2	781	10.08%	4.07%
	Hypertension	2	721	9.31%	3.76%
	Sepsis (Non- maternal and neonatal sepsis)	1	427	5.52%	2.23%
	Unspecified Heart Diseases	3	406	5.25%	2.12%
	Acute kidney failure	1	393	5.08%	2.05%
Kazakhstan	Intermediate cause for CNS	1	4912	12.83%	3.75%

Top 5 ICD10 garbage codes by country - 2015 or most recent year

Country	Garbage code	Garbage class	Deaths	Percentage of all garbage deaths	Percentage of total deaths
Kazakhstan	All, Ill Defined code for causes of death	1	3800	9.92%	2.90%
	Senility	1	3232	8.44%	2.47%
	Heart failure unspecified right or left	2	1207	3.15%	0.92%
	Atherosclerosis	2	1160	3.03%	0.89%
Kiribati	All, Ill Defined code for causes of death	1	100	33.05%	20.97%
	Shock, Cardiac Arrest, Coma	1	12	4.24%	2.69%
	Hepatic Failure	1	10	3.63%	2.30%
	Senility	1	10	3.50%	2.22%
	Fluid, Electrolyte, Acid Base Disorders	1	9	3.29%	2.09%
Kuwait	Sepsis (Non- maternal and neonatal sepsis)	1	345	15.42%	5.31%
	Heart failure unspecified right or left	2	303	13.51%	4.66%
	All, Ill Defined code for causes of death	1	69	3.11%	1.07%
	Unspecified Congenital Diseases	3	58	2.63%	0.90%
	Shock, Cardiac Arrest, Coma	1	57	2.57%	0.89%
Kyrgyzstan	All, Ill Defined code for causes of death	1	833	13.05%	2.39%
	Primary or secondary Liver Cancer Unspecified	3	225	3.52%	0.65%
	Exposure to unspecified factor X59	2	194	3.04%	0.56%
	Shock, Cardiac Arrest, Coma	1	176	2.77%	0.51%
	Hypertension	2	146	2.30%	0.42%
Latvia	Unspecified Site Cancer	3	237	6.66%	0.84%
	All, Ill Defined code for causes of death	1	173	4.87%	0.62%
	Heart failure unspecified right or left	2	132	3.70%	0.47%
	Hypertension	2	132	3.71%	0.47%
	Left heart failure	1	132	3.70%	0.47%
Lithuania	All, Ill Defined code for causes of death	1	483	9.74%	1.16%
	Sepsis (Non- maternal and neonatal sepsis)	1	426	8.59%	1.02%
	Unspecified Site Cancer	3	377	7.61%	0.90%
	Pulmonary Embolism	1	246	4.97%	0.59%
	Undetermined intent Poisoning by multiple or unspecified drug	1	155	3.14%	0.37%
Luxembourg	Heart failure unspecified right or left	2	184	15.16%	4.79%
	Shock, Cardiac Arrest, Coma	1	70	5.84%	1.85%
	All, Ill Defined code for causes of death	1	52	4.33%	1.37%
	Sepsis (Non- maternal and neonatal sepsis)	1	48	4.02%	1.27%
	Cardiac rhythm disorders	3	42	3.47%	1.10%
Malaysia	Sepsis (Non- maternal and neonatal sepsis)	1	4830	12.66%	6.24%
	All, Ill Defined code for causes of death	1	2162	5.67%	2.80%
	External Causes UDI, type unspecified	2	1914	5.02%	2.47%

Top 5 ICD10 garbage codes by country - 2015 or most recent year

Country	Garbage code	Garbage class	Deaths	Percentage of all garbage deaths	Percentage of total deaths
Malaysia	Pneumonitis	1	1782	4.67%	2.30%
	Heart failure unspecified right or left	2	1311	3.44%	1.69%
Maldives	Shock, Cardiac Arrest, Coma	1	155	22.09%	13.37%
	Hypertension	2	84	12.01%	7.26%
	All, Ill Defined code for causes of death	1	65	9.25%	5.60%
	Sepsis (Non- maternal and neonatal sepsis)	1	43	6.17%	3.73%
	Pneumonitis	1	31	4.50%	2.72%
Malta	Left heart failure	1	86	10.19%	2.51%
	Unspecified Site Cancer	3	40	4.82%	1.19%
	Heart failure unspecified right or left	2	23	2.71%	0.67%
	Non-follicular lymphoma, unspecified	3	22	2.60%	0.64%
	Pneumonitis	1	20	2.48%	0.61%
Mauritius	Shock, Cardiac Arrest, Coma	1	246	5.30%	2.60%
	Left heart failure	1	179	3.86%	1.89%
	Unspecified chronic respiratory diseases	3	144	3.10%	1.52%
	Pneumonitis	1	124	2.67%	1.31%
	Heart failure unspecified right or left	2	100	2.15%	1.05%
Mexico	Exposure to unspecified factor X59	2	7523	4.35%	1.15%
	Hypertension	2	6708	3.88%	1.02%
	Hepatic Failure	1	6458	3.74%	0.99%
	All, Ill Defined code for causes of death	1	5841	3.38%	0.89%
	Gastrointestinal Bleeding	2	4656	2.69%	0.71%
Monaco	Heart failure unspecified right or left	2	30	13.62%	5.24%
	Chronic respiratory failure	1	16	7.59%	2.92%
	Atherosclerosis	2	14	6.56%	2.53%
	Sepsis (Non- maternal and neonatal sepsis)	1	7	3.60%	1.38%
	Unspecified Respiratory Cancer	3	7	3.63%	1.40%
Mongolia	Hypertension	2	249	12.36%	1.45%
	right heart failure and pulmonary heart disease	2	200	9.91%	1.16%
	Senility	1	90	4.47%	0.52%
	External Causes UDI, type unspecified	2	83	4.15%	0.49%
	Atherosclerosis	2	75	3.76%	0.44%
Montenegro	All, Ill Defined code for causes of death	1	309	12.47%	6.05%
	Shock, Cardiac Arrest, Coma	1	306	12.34%	5.99%
	Senility	1	198	8.00%	3.89%
	Heart failure unspecified right or left	2	144	5.80%	2.82%
	Unspecified Site Cancer	3	118	4.79%	2.32%

Top 5 ICD10 garbage codes by country - 2015 or most recent year

Country	Garbage code	Garbage class	Deaths	Percentage of all garbage deaths	Percentage of total deaths
Morocco	Shock, Cardiac Arrest, Coma	1	7062	19.73%	13.47%
	Senility	1	6699	18.72%	12.77%
	All, Ill Defined code for causes of death	1	2994	8.37%	5.71%
	Unspecified Heart Diseases	3	2015	5.63%	3.84%
	Exposure to unspecified factor X59	2	1477	4.13%	2.82%
Netherlands	Heart failure unspecified right or left	2	6373	13.14%	4.33%
	All, Ill Defined code for causes of death	1	4180	8.62%	2.84%
	Shock, Cardiac Arrest, Coma	1	2123	4.38%	1.44%
	Unspecified Site Cancer	3	2084	4.30%	1.42%
	Sepsis (Non- maternal and neonatal sepsis)	1	1954	4.03%	1.33%
New Zealand	Unspecified Site Cancer	3	417	8.27%	1.31%
	Left heart failure	1	189	3.76%	0.60%
	All, Ill Defined code for causes of death	1	185	3.67%	0.58%
	Unspecified GI Cancer	3	161	3.21%	0.51%
	Sepsis (Non- maternal and neonatal sepsis)	1	123	2.45%	0.39%
Nicaragua	Hypertension	2	409	7.30%	1.83%
	Alcoholic hepatic failure	3	215	3.85%	0.97%
	Sepsis (Non- maternal and neonatal sepsis)	1	212	3.80%	0.95%
	Hepatic Failure	1	197	3.52%	0.88%
	Primary or secondary Liver Cancer Unspecified	3	192	3.43%	0.86%
North Macedonia	All, Ill Defined code for causes of death	1	982	9.51%	5.11%
	Heart failure unspecified right or left	2	970	9.39%	5.05%
	Shock, Cardiac Arrest, Coma	1	945	9.15%	4.92%
	Hypertension	2	411	3.98%	2.14%
	Atherosclerosis	2	265	2.57%	1.38%
Northern Mariana Islands	Sepsis (Non- maternal and neonatal sepsis)	1	6	8.42%	3.13%
	All, Ill Defined code for causes of death	1	2	3.62%	1.35%
	Exposure to unspecified factor X59	2	2	3.72%	1.38%
	Shock, Cardiac Arrest, Coma	1	2	3.69%	1.37%
	Unspecified Site Cancer	3	2	3.75%	1.39%
Norway	All, Ill Defined code for causes of death	1	1758	15.30%	4.32%
	Heart failure unspecified right or left	2	1210	10.53%	2.98%
	Unspecified Site Cancer	3	379	3.30%	0.93%
	Senility	1	354	3.08%	0.87%
	Unspecified Infectious Diseases	2	330	2.87%	0.81%
Oman	All, Ill Defined code for causes of death	1	2204	41.65%	28.20%
	Shock, Cardiac Arrest, Coma	1	508	9.60%	6.50%

Top 5 ICD10 garbage codes by country - 2015 or most recent year

Country	Garbage code	Garbage class	Deaths	Percentage of all garbage deaths	Percentage of total deaths
Oman	Sepsis (Non- maternal and neonatal sepsis)	1	350	6.63%	4.49%
	External Causes UDI, type unspecified	2	297	5.62%	3.81%
	Hypertension	2	281	5.32%	3.60%
Palestine	Shock, Cardiac Arrest, Coma	1	750	16.16%	5.87%
	Senility	1	694	14.94%	5.43%
	Heart failure unspecified right or left	2	598	12.87%	4.67%
	All, Ill Defined code for causes of death	1	435	9.36%	3.40%
	Sepsis (Non- maternal and neonatal sepsis)	1	328	7.06%	2.57%
Panama	All, Ill Defined code for causes of death	1	482	7.55%	2.65%
	Hypertension	2	365	5.72%	2.01%
	Heart failure unspecified right or left	2	266	4.17%	1.46%
	Unspecified Site Cancer	3	238	3.73%	1.31%
	Shock, Cardiac Arrest, Coma	1	233	3.66%	1.29%
Paraguay	All, Ill Defined code for causes of death	1	2053	22.25%	7.17%
	Heart failure unspecified right or left	2	519	5.62%	1.81%
	Hypertension	2	447	4.85%	1.56%
	Unspecified Site Cancer	3	323	3.50%	1.13%
	Exposure to unspecified factor X59	2	270	2.94%	0.95%
Peru	Unspecified Bacterial Diseases	2	3637	8.60%	3.78%
	Exposure to unspecified factor X59	2	3435	8.12%	3.57%
	Left heart failure	1	2686	6.35%	2.79%
	Hypertension	2	2595	6.13%	2.70%
	Acute kidney failure	1	1177	2.78%	1.22%
Philippines	Shock, Cardiac Arrest, Coma	1	11147	5.32%	1.99%
	All, Ill Defined code for causes of death	1	10457	4.99%	1.87%
	Sepsis (Non- maternal and neonatal sepsis)	1	7440	3.55%	1.33%
	Left heart failure	1	6966	3.32%	1.24%
	Hypertension	2	6656	3.18%	1.19%
Poland	Heart failure unspecified right or left	2	36239	24.07%	9.18%
	Atherosclerosis	2	27298	18.13%	6.91%
	All, Ill Defined code for causes of death	1	13663	9.08%	3.46%
	Left heart failure	1	9371	6.23%	2.37%
	Shock, Cardiac Arrest, Coma	1	9226	6.13%	2.34%
Portugal	Heart failure unspecified right or left	2	3648	8.75%	3.35%
	All, Ill Defined code for causes of death	1	2899	6.96%	2.66%
	Unspecified chronic respiratory diseases	3	1732	4.16%	1.59%
	Shock, Cardiac Arrest, Coma	1	1697	4.07%	1.56%

Top 5 ICD10 garbage codes by country - 2015 or most recent year

Country	Garbage code	Garbage class	Deaths	Percentage of all garbage deaths	Percentage of total deaths
Portugal	Senility	1	1316	3.16%	1.21%
Puerto Rico	Sepsis (Non- maternal and neonatal sepsis)	1	757	7.00%	2.70%
	All, Ill Defined code for causes of death	1	583	5.39%	2.08%
	Acute kidney failure	1	475	4.39%	1.69%
	Unspecified Site Cancer	3	376	3.48%	1.34%
	Hypertension	2	369	3.42%	1.32%
Qatar	All, Ill Defined code for causes of death	1	463	37.76%	21.21%
	Hypertension	2	75	6.19%	3.48%
	Acute Respiratory Failure	1	37	3.04%	1.71%
	Unspecified chronic respiratory diseases	3	35	2.93%	1.64%
	Heart failure unspecified right or left	2	30	2.44%	1.37%
Republic of Korea	Senility	1	12447	15.84%	4.51%
	All, Ill Defined code for causes of death	1	8723	11.10%	3.16%
	Heart failure unspecified right or left	2	4743	6.04%	1.72%
	Shock, Cardiac Arrest, Coma	1	3579	4.55%	1.30%
	Sepsis (Non- maternal and neonatal sepsis)	1	3022	3.85%	1.10%
Republic of Moldova	Primary or secondary Liver Cancer Unspecified	3	416	7.50%	1.04%
	All, Ill Defined code for causes of death	1	262	4.74%	0.66%
	Exposure to unspecified factor X59	2	185	3.34%	0.46%
	Unspecified Site Cancer	3	126	2.28%	0.32%
	Pulmonary Embolism	1	93	1.69%	0.24%
Romania	Hypertension	2	16208	17.49%	6.20%
	Atherosclerosis	2	9415	10.16%	3.60%
	right heart failure and pulmonary heart disease	2	2670	2.88%	1.02%
	All, Ill Defined code for causes of death	1	2454	2.65%	0.94%
	Primary or secondary Liver Cancer Unspecified	3	2192	2.37%	0.84%
Russian Federation	Senility	1	94113	21.20%	5.02%
	All, Ill Defined code for causes of death	1	39956	9.00%	2.13%
	Intermediate cause for CNS	1	24747	5.58%	1.32%
	Undetermined intent of fall	2	10809	2.44%	0.58%
	Atherosclerosis	2	10749	2.42%	0.57%
Saint Kitts and Nevis	Heart failure unspecified right or left	2	6	4.05%	1.62%
	Hypertension	2	6	4.10%	1.64%
	Undetermined intent Drowning	2	6	4.73%	1.89%
	Left heart failure	1	5	3.69%	1.47%
	Unspecified Site Cancer	3	4	3.36%	1.34%
Saint Lucia	Hypertension	2	29	6.32%	2.71%

Top 5 ICD10 garbage codes by country - 2015 or most recent year

Country	Garbage code	Garbage class	Deaths	Percentage of all garbage deaths	Percentage of total deaths
Saint Lucia	Sepsis (Non- maternal and neonatal sepsis)	1	18	3.86%	1.65%
	Unspecified Site Cancer	3	18	4.02%	1.72%
	All, Ill Defined code for causes of death	1	17	3.78%	1.62%
	Exposure to unspecified factor X59	2	11	2.52%	1.08%
Saint Vincent and the Grenadines	Hypertension	2	22	6.36%	2.51%
	Sepsis (Non- maternal and neonatal sepsis)	1	19	5.66%	2.23%
	All, Ill Defined code for causes of death	1	18	5.34%	2.11%
	Pneumonitis	1	14	4.15%	1.64%
	Left heart failure	1	9	2.72%	1.07%
Serbia	Hypertension	2	4564	10.32%	4.40%
	All, Ill Defined code for causes of death	1	3992	9.03%	3.85%
	Shock, Cardiac Arrest, Coma	1	3311	7.49%	3.19%
	Heart failure unspecified right or left	2	2978	6.74%	2.87%
	Atherosclerosis	2	1734	3.92%	1.67%
Singapore	Unspecified Site Cancer	3	151	2.80%	0.76%
	Non-follicular lymphoma, unspecified	3	89	1.65%	0.45%
	All, Ill Defined code for causes of death	1	74	1.39%	0.38%
	Unspecified Bronchitis and Bronchiectasis	3	73	1.36%	0.37%
	Pulmonary Embolism	1	53	1.00%	0.27%
Slovakia	Heart failure unspecified right or left	2	815	9.66%	1.59%
	All, Ill Defined code for causes of death	1	623	7.38%	1.21%
	External Causes UDI, type unspecified	2	527	6.25%	1.03%
	Unspecified Site Cancer	3	426	5.05%	0.83%
	Senility	1	356	4.23%	0.69%
Slovenia	Heart failure unspecified right or left	2	1607	35.48%	8.10%
	All, Ill Defined code for causes of death	1	249	5.51%	1.26%
	Unspecified Site Cancer	3	233	5.16%	1.18%
	Atherosclerosis	2	182	4.03%	0.92%
	Sepsis (Non- maternal and neonatal sepsis)	1	87	1.93%	0.44%
South Africa	All, Ill Defined code for causes of death	1	56759	23.70%	12.03%
	Exposure to unspecified factor X59	2	16386	6.84%	3.47%
	Heart failure unspecified right or left	2	12557	5.24%	2.66%
	HIV correction for Immunodeficiency other	3	10330	4.31%	2.19%
	Hypertension	2	9936	4.15%	2.11%
Spain	Heart failure unspecified right or left	2	15974	12.00%	3.78%
	Unspecified chronic respiratory diseases	3	13091	9.84%	3.10%
	All, Ill Defined code for causes of death	1	8765	6.59%	2.07%

Top 5 ICD10 garbage codes by country - 2015 or most recent year

Country	Garbage code	Garbage class	Deaths	Percentage of all garbage deaths	Percentage of total deaths
Spain	Left heart failure	1	4688	3.52%	1.11%
	Unspecified Site Cancer	3	4462	3.35%	1.06%
Sri Lanka	Senility	1	11112	14.32%	8.53%
	Unspecified Heart Diseases	3	9193	11.85%	7.05%
	Plegia	1	7631	9.83%	5.85%
	Hypertension	2	6945	8.95%	5.33%
	All, Ill Defined code for causes of death	1	6091	7.85%	4.67%
Suriname	Senility	1	203	15.36%	6.48%
	Heart failure unspecified right or left	2	106	7.98%	3.37%
	Sepsis (Non- maternal and neonatal sepsis)	1	69	5.25%	2.22%
	All, Ill Defined code for causes of death	1	35	2.66%	1.12%
	Shock, Cardiac Arrest, Coma	1	34	2.59%	1.09%
Sweden	Heart failure unspecified right or left	2	3338	12.59%	3.67%
	All, Ill Defined code for causes of death	1	1931	7.28%	2.12%
	Unspecified Site Cancer	3	1180	4.45%	1.30%
	Sepsis (Non- maternal and neonatal sepsis)	1	1134	4.28%	1.25%
	Senility	1	1027	3.87%	1.13%
Switzerland	All, Ill Defined code for causes of death	1	2805	15.80%	4.15%
	Heart failure unspecified right or left	2	1431	8.06%	2.12%
	Shock, Cardiac Arrest, Coma	1	831	4.68%	1.23%
	Hypertension	2	777	4.38%	1.15%
	Senility	1	558	3.15%	0.83%
Syrian Arab Republic	Shock, Cardiac Arrest, Coma	1	14401	45.28%	21.49%
	Heart failure unspecified right or left	2	3854	12.12%	5.75%
	All, Ill Defined code for causes of death	1	1884	5.93%	2.81%
	Acute Respiratory Failure	1	1229	3.86%	1.83%
	Sepsis (Non- maternal and neonatal sepsis)	1	1227	3.86%	1.83%
Taiwan (Province of China)	Primary or secondary Liver Cancer Unspecified	3	6966	10.96%	4.26%
	Sepsis (Non- maternal and neonatal sepsis)	1	3349	5.27%	2.05%
	Heart failure unspecified right or left	2	2578	4.06%	1.58%
	All, Ill Defined code for causes of death	1	2232	3.51%	1.37%
	Shock, Cardiac Arrest, Coma	1	2193	3.45%	1.34%
Thailand	All, Ill Defined code for causes of death	1	94862	42.39%	21.27%
	Sepsis (Non- maternal and neonatal sepsis)	1	26327	11.77%	5.90%
	Acute kidney failure	1	8909	3.98%	2.00%
	External Causes UDI, type unspecified	2	7341	3.28%	1.65%
	Hypertension	2	5756	2.57%	1.29%

Top 5 ICD10 garbage codes by country - 2015 or most recent year

Country	Garbage code	Garbage class	Deaths	Percentage of all garbage deaths	Percentage of total deaths
Trinidad and Tobago	Hypertension	2	126	3.47%	1.31%
	Unspecified Site Cancer	3	90	2.50%	0.94%
	All, Ill Defined code for causes of death	1	83	2.30%	0.87%
	Shock, Cardiac Arrest, Coma	1	70	1.93%	0.73%
	Sepsis (Non- maternal and neonatal sepsis)	1	66	1.84%	0.69%
Tunisia	All, Ill Defined code for causes of death	1	1981	16.95%	9.05%
	Shock, Cardiac Arrest, Coma	1	999	8.54%	4.57%
	External Causes UDI, type unspecified	2	771	6.60%	3.53%
	Senility	1	664	5.68%	3.03%
	Sepsis (Non- maternal and neonatal sepsis)	1	446	3.82%	2.04%
Turkey	Heart failure unspecified right or left	2	18210	14.34%	4.59%
	Left heart failure	1	6214	4.89%	1.57%
	Sepsis (Non- maternal and neonatal sepsis)	1	5719	4.50%	1.44%
	All, Ill Defined code for causes of death	1	4949	3.90%	1.25%
	Shock, Cardiac Arrest, Coma	1	4926	3.88%	1.24%
Ukraine	Atherosclerosis	2	15375	19.16%	2.58%
	Senility	1	7525	9.38%	1.27%
	All, Ill Defined code for causes of death	1	3505	4.37%	0.59%
	Heart failure unspecified right or left	2	2882	3.59%	0.48%
	Unspecified Site Cancer	3	2388	2.98%	0.40%
United Kingdom	Unspecified Site Cancer	3	8485	5.87%	1.71%
	Senility	1	7599	5.26%	1.53%
	All, Ill Defined code for causes of death	1	5194	3.59%	1.05%
	Left heart failure	1	3057	2.12%	0.62%
	Unspecified GI Cancer	3	2868	1.98%	0.58%
United States of America	Left heart failure	1	62967	8.20%	2.32%
	Undetermined intent Poisoning by multiple or unspecified drug	1	46011	5.99%	1.70%
	Sepsis (Non- maternal and neonatal sepsis)	1	41965	5.46%	1.55%
	Unspecified Site Cancer	3	37340	4.86%	1.38%
	All, Ill Defined code for causes of death	1	25203	3.28%	0.93%
United States Virgin Islands	Hypertension	2	29	11.00%	4.36%
	All, Ill Defined code for causes of death	1	25	9.74%	3.86%
	Shock, Cardiac Arrest, Coma	1	14	5.62%	2.23%
	Unspecified Site Cancer	3	13	5.25%	2.08%
	Sepsis (Non- maternal and neonatal sepsis)	1	12	4.82%	1.91%
Uruguay	All, Ill Defined code for causes of death	1	2546	19.82%	7.73%
	Heart failure unspecified right or left	2	805	6.27%	2.44%

Top 5 ICD10 garbage codes by country - 2015 or most recent year

Country	Garbage code	Garbage class	Deaths	Percentage of all garbage deaths	Percentage of total deaths
Uruguay	Unspecified Site Cancer	3	615	4.79%	1.87%
	Shock, Cardiac Arrest, Coma	1	571	4.45%	1.73%
	Exposure to unspecified factor X59	2	461	3.59%	1.40%
Uzbekistan	Atherosclerosis	2	3737	10.00%	2.46%
	Heart failure unspecified right or left	2	3143	8.41%	2.07%
	right heart failure and pulmonary heart disease	2	2138	5.72%	1.41%
	Unspecified Heart Diseases	3	1406	3.76%	0.93%
	Hypertension	2	1307	3.50%	0.86%
Venezuela (Bolivarian Republic of)	Undetermined intent shooting by unspecified firearm	2	6287	15.47%	3.88%
	External Causes UDI, type unspecified	2	1247	3.07%	0.77%
	Sepsis (Non- maternal and neonatal sepsis)	1	1116	2.75%	0.69%
	Hypertension	2	1085	2.67%	0.67%
	Unspecified Site Cancer	3	898	2.21%	0.55%
Zimbabwe	All, Ill Defined code for causes of death	1	2924	11.49%	4.70%
	External Causes UDI, type unspecified	2	1935	7.60%	3.11%
	Heart failure unspecified right or left	2	1866	7.33%	3.00%
	Hypertension	2	976	3.84%	1.57%
	Acute kidney failure	1	754	2.96%	1.21%

The above table contains the top 5 all age, both sex ICD10 garbage codes for all countries with at least one year of ICD10 vital registration data. The displayed year is 2015, or the most recent year available for each country if 2015 is not available. The most specific class of garbage, class 4, has been omitted from this table in order to place focus on the most impactful garbage codes with respect to final GBD cause estimation. Lastly, during the correction step for the misassignment of dementia, parkinson's, and atrial fibrillation (Appendix Figure 1), dementia deaths may be recoded to garbage in the event of an overcoding of dementia. Garbage generated in this manner have been excluded from the above table.

Appendix Figure 18

Leading causes of death before and after garbage code redistribution: Albania - 2010.

Causes are connected by arrows before and after redistribution. Infectious diseases are shown in red, non-communicable causes in blue, and injuries in green. In addition to garbage redistribution, the diagram also reflects the deaths moved during misassignment correction for Alzheimer's disease and other dementias.

- Non-Communicable Diseases
- Infectious Diseases
- Injuries

Cause ranking decreased during redistribution (dashed line)

Cause ranking increased or stayed the same during redistribution (solid line)

Leading causes Before Redistribution	Deaths	Leading causes After Redistribution	Deaths
1 Ischemic heart disease	2179.0	1 Ischemic heart disease	3574.51
2 Intracerebral hemorrhage	1794.0	2 Intracerebral hemorrhage	2094.87
3 Ischemic stroke	410.0	3 Ischemic stroke	685.88
4 Tracheal, bronchus, and lung cancer	352.0	4 Tracheal, bronchus, and lung cancer	452.29
5 Other malignant neoplasms (internal)	254.0	5 Alzheimer's disease and other dementias	414.53
6 Other cardiovascular and circulatory diseases (internal)	177.0	6 Other malignant neoplasms (internal)	307.57
7 Stomach cancer	165.0	7 Other cardiovascular and circulatory diseases (internal)	304.37
8 Liver cancer	141.0	8 Chronic obstructive pulmonary disease	230.38
9 Cirrhosis and other chronic liver diseases	138.5	9 Stomach cancer	188.31
10 Breast cancer	132.0	10 Cirrhosis and other chronic liver diseases	168.9
11 Brain and central nervous system cancer	116.0	11 Liver cancer	150.6
12 Prostate cancer	112.0	12 Breast cancer	149.03
13 Chronic kidney disease	92.0	13 Hypertensive heart disease	146.44
14 Other musculoskeletal disorders	90.0	14 Colon and rectum cancer	144.35
15 Other unintentional injuries	87.0	15 Prostate cancer	138.65
16 Self-harm by other specified means	85.0	16 Brain and central nervous system cancer	135.16
17 Colon and rectum cancer	80.0	17 Chronic kidney disease	125.19
18 Pancreatic cancer	77.0	18 Pedestrian road injuries	113.59
19 Idiopathic epilepsy	73.0	19 Self-harm by other specified means	109.78
20 Chronic obstructive pulmonary disease	73.0	20 Parkinson's disease	107.98
25 Hypertensive heart disease		21 Other musculoskeletal disorders	
55 Alzheimer's disease and other dementias		22 Pancreatic cancer	
89 Parkinson's disease		23 Other unintentional injuries	
124 Pedestrian road injuries		25 Idiopathic epilepsy	

Leading causes of death before and after garbage code redistribution: Andorra - 2015.

Causes are connected by arrows before and after redistribution. Infectious diseases are shown in red, non-communicable causes in blue, and injuries in green.
In addition to garbage redistribution, the diagram also reflects the deaths moved during misassignment correction for Alzheimer's disease and other dementias.

Leading causes Before Redistribution	Deaths	Leading causes After Redistribution	Deaths
1 Alzheimer's disease and other dementias	24.65	1 Ischemic heart disease	27.78
2 Tracheal, bronchus, and lung cancer	21.15	2 Tracheal, bronchus, and lung cancer	24.76
3 Ischemic heart disease	17.0	3 Alzheimer's disease and other dementias	22.29
4 Stroke	14.0	4 Colon and rectum cancer	13.95
5 Colon and rectum cancer	11.06	5 Stroke	13.62
6 Falls	11.0	6 Falls	13.53
7 Chronic respiratory diseases	8.0	7 Hypertensive heart disease	9.35
8 Lower respiratory infections	6.51	8 Chronic respiratory diseases	8.52
9 Hypertensive heart disease	6.1	9 Lower respiratory infections	8.07
10 Stomach cancer	5.03	10 Stomach cancer	6.67
11 Breast cancer	5.0	11 Breast cancer	5.84
12 Self-harm	5.0	12 Self-harm	5.32
13 Cirrhosis and other chronic liver diseases	4.05	13 Cirrhosis and other chronic liver diseases	5.07
14 Esophageal cancer	4.0	14 Esophageal cancer	4.93
15 Brain and central nervous system cancer	4.0	15 Brain and central nervous system cancer	4.86
16 Prostate cancer	3.04	16 Prostate cancer	4.75
17 Pancreatic cancer	3.0	17 Interstitial lung disease and pulmonary sarcoidosis	4.36
18 HIV/AIDS	3.0	18 Pancreatic cancer	3.96
19 Leukemia	3.0	19 Leukemia	3.93
20 Interstitial lung disease and pulmonary sarcoidosis	2.74	20 Parkinson's disease	3.77
23 Parkinson's disease		24 HIV/AIDS	

Leading causes of death before and after garbage code redistribution: United Arab Emirates - 2007.

Causes are connected by arrows before and after redistribution. Infectious diseases are shown in red, non-communicable causes in blue, and injuries in green.

In addition to garbage redistribution, the diagram also reflects the deaths moved during misassignment correction for Alzheimer's disease and other dementias.

Leading causes Before Redistribution	Deaths	Leading causes After Redistribution	Deaths
1 Injuries	2382.0	1 Injuries	2629.6
2 Road injuries	915.0	2 Ischemic heart disease	1120.16
3 Ischemic heart disease	510.0	3 Road injuries	1114.79
4 Chronic respiratory diseases	360.0	4 Stroke	574.19
5 Other malignant neoplasms (internal)	336.0	5 Chronic respiratory diseases	410.23
6 Congenital birth defects	292.0	6 Other malignant neoplasms (internal)	391.52
7 Diabetes mellitus	286.0	7 Diabetes mellitus	338.71
8 Stroke	262.0	8 Congenital birth defects	317.77
9 Neonatal disorders	176.0	9 Lower respiratory infections	209.0
10 Lower respiratory infections	173.0	10 Neonatal disorders	186.75
11 Falls	136.0	11 Adverse effects of medical treatment	165.6
12 Adverse effects of medical treatment	135.0	12 Falls	160.3
13 Chronic kidney disease	111.0	13 Chronic kidney disease	132.5
14 Self-harm	102.0	14 Self-harm	122.8
15 Cirrhosis and other chronic liver diseases	101.0	15 Cirrhosis and other chronic liver diseases	116.31
16 Tracheal, bronchus, and lung cancer	92.0	16 Tracheal, bronchus, and lung cancer	108.18
17 Colon and rectum cancer	74.0	17 Colon and rectum cancer	86.54
18 Breast cancer	68.0	18 Breast cancer	76.77
19 Drowning	50.0	19 Meningitis	69.19
20 Leukemia	45.0	20 Fire, heat, and hot substances	59.3
21 Fire, heat, and hot substances		22 Drowning	
26 Meningitis		23 Leukemia	

Leading causes of death before and after garbage code redistribution: Argentina - 2015.

Causes are connected by arrows before and after redistribution. Infectious diseases are shown in red, non-communicable causes in blue, and injuries in green.
In addition to garbage redistribution, the diagram also reflects the deaths moved during misassignment correction for Alzheimer's disease and other dementias.

Leading causes Before Redistribution	Deaths
1 Ischemic heart disease	21923.0
2 Tracheal, bronchus, and lung cancer	9921.0
3 Colon and rectum cancer	7675.0
4 Chronic obstructive pulmonary disease	7106.0
5 Breast cancer	5999.0
6 Hypertensive heart disease	4638.0
7 Intracerebral hemorrhage	4508.0
8 Cirrhosis and other chronic liver diseases	4278.0
9 Pancreatic cancer	4245.0
10 Chronic kidney disease	4057.0
11 Prostate cancer	3837.0
12 Urinary tract infections and interstitial nephritis	3664.0
13 Alzheimer's disease and other dementias	3117.0
14 Other cardiovascular and circulatory diseases (internal)	3068.0
15 Stomach cancer	3043.0
16 Self-harm by other specified means	2441.0
17 Neonatal preterm birth	2330.0
18 Motor vehicle road injuries	2250.0
19 Kidney cancer	2018.0
20 Esophageal cancer	1740.0
26 Diabetes mellitus type 2	
47 Ischemic stroke	
58 Other cardiomyopathy	
96 Other lower respiratory infections	
157 Pneumococcal pneumonia	

Leading causes After Redistribution	Deaths
1 Ischemic heart disease	37588.0
2 Other lower respiratory infections	26821.01
3 Chronic obstructive pulmonary disease	15836.87
4 Ischemic stroke	13174.99
5 Tracheal, bronchus, and lung cancer	12846.45
6 Colon and rectum cancer	11950.59
7 Alzheimer's disease and other dementias	11557.98
8 Intracerebral hemorrhage	10256.75
9 Diabetes mellitus type 2	9675.55
10 Hypertensive heart disease	9453.65
11 Breast cancer	7761.99
12 Chronic kidney disease	7598.14
13 Cirrhosis and other chronic liver diseases	6934.17
14 Other cardiomyopathy	6257.8
15 Pancreatic cancer	5617.25
16 Prostate cancer	5344.46
17 Stomach cancer	5242.33
18 Other cardiovascular and circulatory diseases (internal)	4859.64
19 Urinary tract infections and interstitial nephritis	4830.9
20 Pneumococcal pneumonia	4406.87
22 Self-harm by other specified means	
23 Motor vehicle road injuries	
28 Esophageal cancer	
29 Kidney cancer	
31 Neonatal preterm birth	

Leading causes of death before and after garbage code redistribution: Armenia - 2015.

Causes are connected by arrows before and after redistribution. Infectious diseases are shown in red, non-communicable causes in blue, and injuries in green.
In addition to garbage redistribution, the diagram also reflects the deaths moved during misassignment correction for Alzheimer's disease and other dementias.

Leading causes Before Redistribution	Deaths
1 Ischemic heart disease	8118.0
2 Tracheal, bronchus, and lung cancer	1242.0
3 Chronic obstructive pulmonary disease	1231.0
4 Ischemic stroke	1139.0
5 Cirrhosis and other chronic liver diseases	1039.0
6 Hypertensive heart disease	633.0
7 Breast cancer	577.0
8 Urinary tract infections and interstitial nephritis	546.0
9 Diabetes mellitus type 2	511.0
10 Colon and rectum cancer	508.0
11 Stomach cancer	508.0
12 Intracerebral hemorrhage	507.0
13 Liver cancer	424.0
14 Aortic aneurysm	346.0
15 Pancreatic cancer	344.0
16 Prostate cancer	304.0
17 Chronic kidney disease due to other and unspecified causes	263.0
18 Peptic ulcer disease	250.0
19 Brain and central nervous system cancer	230.0
20 Bladder cancer	198.0
35 Self-harm by other specified means	
47 Other lower respiratory infections	
55 Alzheimer's disease and other dementias	

Leading causes After Redistribution	Deaths
1 Ischemic heart disease	8519.65
2 Ischemic stroke	1620.07
3 Chronic obstructive pulmonary disease	1333.14
4 Tracheal, bronchus, and lung cancer	1293.55
5 Diabetes mellitus type 2	1202.18
6 Cirrhosis and other chronic liver diseases	1076.27
7 Alzheimer's disease and other dementias	686.71
8 Hypertensive heart disease	658.73
9 Intracerebral hemorrhage	657.26
10 Breast cancer	607.79
11 Colon and rectum cancer	577.7
12 Stomach cancer	565.08
13 Other lower respiratory infections	560.21
14 Urinary tract infections and interstitial nephritis	511.7
15 Liver cancer	433.39
16 Pancreatic cancer	371.03
17 Aortic aneurysm	367.43
18 Prostate cancer	318.31
19 Self-harm by other specified means	305.01
20 Chronic kidney disease due to other and unspecified causes	271.18
21 Peptic ulcer disease	
22 Brain and central nervous system cancer	
24 Bladder cancer	

Leading causes of death before and after garbage code redistribution: American Samoa - 2015.

Causes are connected by arrows before and after redistribution. Infectious diseases are shown in red, non-communicable causes in blue, and injuries in green.
In addition to garbage redistribution, the diagram also reflects the deaths moved during misassignment correction for Alzheimer's disease and other dementias.

Leading causes Before Redistribution	Deaths
1 Ischemic heart disease	44.0
2 Chronic kidney disease due to diabetes mellitus type 2	10.0
3 Chronic obstructive pulmonary disease	10.0
4 Intracerebral hemorrhage	9.0
5 Stomach cancer	7.0
6 Breast cancer	6.0
7 Tracheal, bronchus, and lung cancer	6.0
8 Endocrine, metabolic, blood, and immune disorders	6.0
9 Diabetes mellitus type 2	5.0
10 Cirrhosis and other chronic liver diseases	4.0
11 Colon and rectum cancer	4.0
12 Decubitus ulcer	4.0
13 Asthma	4.0
14 Other endocrine, metabolic, blood, and immune disorders	3.0
15 Atrial fibrillation and flutter	3.0
16 Urinary tract infections and interstitial nephritis	3.0
17 Self-harm by other specified means	2.0
18 Other cardiovascular and circulatory diseases (internal)	2.0
19 Pyoderma	2.0
20 Protein-energy malnutrition	2.0
21 Chronic kidney disease due to diabetes mellitus type 1	
45 Ischemic stroke	
46 Diarrheal diseases	
62 Other cardiomyopathy	
63 Other lower respiratory infections	
64 Alzheimer's disease and other dementias	

Leading causes After Redistribution	Deaths
1 Ischemic heart disease	50.39
2 Diabetes mellitus type 2	27.72
3 Intracerebral hemorrhage	22.0
4 Chronic kidney disease due to diabetes mellitus type 2	20.68
5 Chronic obstructive pulmonary disease	11.96
6 Stomach cancer	10.28
7 Ischemic stroke	8.8
8 Tracheal, bronchus, and lung cancer	7.59
9 Breast cancer	7.16
10 Endocrine, metabolic, blood, and immune disorders	5.98
11 Other lower respiratory infections	5.81
12 Colon and rectum cancer	5.31
13 Cirrhosis and other chronic liver diseases	4.46
14 Diarrheal diseases	4.37
15 Alzheimer's disease and other dementias	4.17
16 Asthma	3.96
17 Chronic kidney disease due to diabetes mellitus type 1	3.62
18 Decubitus ulcer	3.5
19 Atrial fibrillation and flutter	3.4
20 Other cardiomyopathy	3.26
21 Other endocrine, metabolic, blood, and immune disorders	
22 Urinary tract infections and interstitial nephritis	
24 Self-harm by other specified means	
26 Other cardiovascular and circulatory diseases (internal)	
31 Pyoderma	
36 Protein-energy malnutrition	

Leading causes of death before and after garbage code redistribution: Antigua and Barbuda - 2015.

Causes are connected by arrows before and after redistribution. Infectious diseases are shown in red, non-communicable causes in blue, and injuries in green.
In addition to garbage redistribution, the diagram also reflects the deaths moved during misassignment correction for Alzheimer’s disease and other dementias.

Leading causes Before Redistribution	Deaths
1 Ischemic heart disease	44.0
2 Hypertensive heart disease	18.0
3 Prostate cancer	17.0
4 Intracerebral hemorrhage	12.0
5 Breast cancer	12.0
6 Colon and rectum cancer	10.0
7 Urinary tract infections and interstitial nephritis	7.0
8 Decubitus ulcer	7.0
9 Chronic kidney disease due to hypertension	6.0
10 Stomach cancer	6.0
11 Liver cancer	6.0
12 Alzheimer's disease and other dementias	5.0
13 Sickle cell disorders	5.0
14 Cirrhosis and other chronic liver diseases	5.0
15 Alcohol use disorders	4.0
16 Unintentional firearm injuries	4.0
17 Diabetes mellitus type 2	4.0
18 Chronic kidney disease due to glomerulonephritis	4.0
19 Cervical cancer	4.0
20 Congenital heart anomalies	4.0
21 Other cardiovascular and circulatory diseases (internal)	
66 Ischemic stroke	
71 Other lower respiratory infections	

Leading causes After Redistribution	Deaths
1 Ischemic heart disease	63.31
2 Diabetes mellitus type 2	44.4
3 Hypertensive heart disease	29.93
4 Ischemic stroke	28.68
5 Intracerebral hemorrhage	26.68
6 Other lower respiratory infections	23.87
7 Prostate cancer	21.04
8 Chronic kidney disease due to hypertension	15.97
9 Breast cancer	14.21
10 Colon and rectum cancer	12.63
11 Alzheimer's disease and other dementias	11.42
12 Chronic kidney disease due to glomerulonephritis	8.58
13 Decubitus ulcer	8.18
14 Stomach cancer	7.95
15 Urinary tract infections and interstitial nephritis	7.75
16 Other cardiovascular and circulatory diseases (internal)	7.66
17 Cirrhosis and other chronic liver diseases	7.14
18 Liver cancer	6.25
19 Unintentional firearm injuries	5.58
20 Sickle cell disorders	5.31
21 Cervical cancer	
23 Alcohol use disorders	
28 Congenital heart anomalies	

Leading causes of death before and after garbage code redistribution: Australia - 2015.

Causes are connected by arrows before and after redistribution. Infectious diseases are shown in red, non-communicable causes in blue, and injuries in green.
In addition to garbage redistribution, the diagram also reflects the deaths moved during misassignment correction for Alzheimer's disease and other dementias.

Leading causes Before Redistribution	Deaths	Leading causes After Redistribution	Deaths
1 Ischemic heart disease	19898.0	1 Ischemic heart disease	22913.16
2 Alzheimer's disease and other dementias	13120.0	2 Alzheimer's disease and other dementias	11333.18
3 Tracheal, bronchus, and lung cancer	8490.0	3 Tracheal, bronchus, and lung cancer	9340.4
4 Chronic obstructive pulmonary disease	7156.0	4 Chronic obstructive pulmonary disease	8552.06
5 Colon and rectum cancer	4460.0	5 Ischemic stroke	7678.01
6 Prostate cancer	3196.0	6 Colon and rectum cancer	6218.72
7 Breast cancer	2968.0	7 Prostate cancer	3893.84
8 Self-harm by other specified means	2871.0	8 Falls	3857.19
9 Pancreatic cancer	2764.0	9 Diabetes mellitus type 2	3578.19
10 Falls	2565.0	10 Breast cancer	3375.2
11 Atrial fibrillation and flutter	2139.0	11 Atrial fibrillation and flutter	3327.41
12 Intracerebral hemorrhage	1784.0	12 Pancreatic cancer	3117.43
13 Parkinson's disease	1712.0	13 Self-harm by other specified means	3049.09
14 Liver cancer	1634.0	14 Intracerebral hemorrhage	2944.3
15 Diabetes mellitus type 2	1550.0	15 Parkinson's disease	2214.35
16 Ischemic stroke	1546.0	16 Influenza	2114.66
17 Malignant skin melanoma	1523.0	17 Cirrhosis and other chronic liver diseases	2045.77
18 Cirrhosis and other chronic liver diseases	1492.0	18 Chronic kidney disease	1837.66
19 Chronic kidney disease	1475.0	19 Liver cancer	1821.27
20 Brain and central nervous system cancer	1410.0	20 Other non-Hodgkin lymphoma	1749.61
48 Other non-Hodgkin lymphoma		22 Malignant skin melanoma	
64 Influenza		24 Brain and central nervous system cancer	

Leading causes of death before and after garbage code redistribution: Austria - 2015.

Causes are connected by arrows before and after redistribution. Infectious diseases are shown in red, non-communicable causes in blue, and injuries in green.
In addition to garbage redistribution, the diagram also reflects the deaths moved during misassignment correction for Alzheimer’s disease and other dementias.

Leading causes Before Redistribution	Deaths	Leading causes After Redistribution	Deaths
1 Ischemic heart disease	14905.0	1 Ischemic heart disease	17453.44
2 Tracheal, bronchus, and lung cancer	3905.0	2 Alzheimer's disease and other dementias	5819.39
3 Chronic obstructive pulmonary disease	2827.0	3 Tracheal, bronchus, and lung cancer	4369.75
4 Hypertensive heart disease	2779.0	4 Hypertensive heart disease	3544.15
5 Alzheimer's disease and other dementias	2238.0	5 Ischemic stroke	3303.69
6 Colon and rectum cancer	2061.0	6 Chronic obstructive pulmonary disease	3046.48
7 Cirrhosis and other chronic liver diseases	1733.0	7 Colon and rectum cancer	2472.04
8 Pancreatic cancer	1671.0	8 Diabetes mellitus type 2	2371.26
9 Breast cancer	1592.0	9 Cirrhosis and other chronic liver diseases	1934.33
10 Ischemic stroke	1319.0	10 Pancreatic cancer	1898.16
11 Atrial fibrillation and flutter	1271.0	11 Breast cancer	1834.28
12 Chronic kidney disease due to hypertension	1258.0	12 Falls	1728.37
13 Prostate cancer	1129.0	13 Chronic kidney disease due to hypertension	1635.26
14 Self-harm by other specified means	1024.0	14 Other cardiomyopathy	1516.18
15 Intracerebral hemorrhage	969.0	15 Atrial fibrillation and flutter	1472.75
16 Diabetes mellitus type 2	956.0	16 Prostate cancer	1455.91
17 Falls	892.0	17 Intracerebral hemorrhage	1325.35
18 Non-rheumatic calcific aortic valve disease	859.0	18 Self-harm by other specified means	1257.37
19 Parkinson's disease	859.0	19 Non-rheumatic calcific aortic valve disease	1083.74
20 Stomach cancer	829.0	20 Chronic kidney disease due to diabetes mellitus type 2	1054.94
30 Chronic kidney disease due to diabetes mellitus type 2		21 Stomach cancer	
107 Other cardiomyopathy		22 Parkinson's disease	

Leading causes of death before and after garbage code redistribution: Azerbaijan - 2007.

Causes are connected by arrows before and after redistribution. Infectious diseases are shown in red, non-communicable causes in blue, and injuries in green.

In addition to garbage redistribution, the diagram also reflects the deaths moved during misassignment correction for Alzheimer's disease and other dementias.

Leading causes Before Redistribution	Deaths
1 Ischemic heart disease	6060.0
2 Intracerebral hemorrhage	4588.0
3 Ischemic stroke	915.0
4 Cirrhosis and other chronic liver diseases	644.0
5 Stomach cancer	608.0
6 Tracheal, bronchus, and lung cancer	544.0
7 Neonatal preterm birth	432.0
8 Drug-susceptible tuberculosis	291.0
9 Chronic kidney disease	264.0
10 Liver cancer	260.0
11 Breast cancer	260.0
12 Esophageal cancer	225.0
13 Colon and rectum cancer	222.0
14 Neonatal encephalopathy due to birth asphyxia and trauma	219.0
15 Other digestive diseases	198.0
16 Brain and central nervous system cancer	196.0
17 Asthma	174.0
18 Leukemia	136.0
19 Other cardiovascular and circulatory diseases (internal)	123.0
20 Diabetes mellitus type 2	118.0
22 Chronic obstructive pulmonary disease	
35 Hypertensive heart disease	
38 Self-harm by other specified means	
48 Other lower respiratory infections	
103 Alzheimer's disease and other dementias	
149 Other cardiomyopathy	

Leading causes After Redistribution	Deaths
1 Ischemic heart disease	15997.29
2 Intracerebral hemorrhage	7507.17
3 Ischemic stroke	4075.91
4 Cirrhosis and other chronic liver diseases	2712.05
5 Other lower respiratory infections	1487.0
6 Tracheal, bronchus, and lung cancer	1096.39
7 Stomach cancer	1078.7
8 Hypertensive heart disease	1014.83
9 Diabetes mellitus type 2	993.08
10 Chronic obstructive pulmonary disease	856.67
11 Other cardiomyopathy	605.08
12 Breast cancer	601.25
13 Alzheimer's disease and other dementias	588.32
14 Chronic kidney disease	586.82
15 Colon and rectum cancer	509.64
16 Drug-susceptible tuberculosis	505.3
17 Self-harm by other specified means	498.93
18 Liver cancer	487.05
19 Neonatal preterm birth	470.41
20 Esophageal cancer	344.99
22 Brain and central nervous system cancer	
26 Asthma	
28 Neonatal encephalopathy due to birth asphyxia and trauma	
29 Other digestive diseases	
36 Other cardiovascular and circulatory diseases (internal)	
37 Leukemia	

Leading causes of death before and after garbage code redistribution: Belgium - 2015.

Causes are connected by arrows before and after redistribution. Infectious diseases are shown in red, non-communicable causes in blue, and injuries in green.
In addition to garbage redistribution, the diagram also reflects the deaths moved during misassignment correction for Alzheimer's disease and other dementias.

Leading causes Before Redistribution	Deaths
1 Ischemic heart disease	7845.0
2 Alzheimer's disease and other dementias	7451.0
3 Tracheal, bronchus, and lung cancer	6489.0
4 Chronic obstructive pulmonary disease	4210.0
5 Colon and rectum cancer	2953.0
6 Breast cancer	2197.0
7 Pancreatic cancer	1711.0
8 Self-harm by other specified means	1637.0
9 Falls	1618.0
10 Intracerebral hemorrhage	1582.0
11 Atrial fibrillation and flutter	1558.0
12 Prostate cancer	1546.0
13 Cirrhosis and other chronic liver diseases	1431.0
14 Parkinson's disease	1062.0
15 Ischemic stroke	1055.0
16 Bladder cancer	996.0
17 Non-rheumatic calcific aortic valve disease	968.0
18 Urinary tract infections and interstitial nephritis	933.0
19 Stomach cancer	764.0
20 Esophageal cancer	755.0
42 Diabetes mellitus type 2	
46 Influenza	
55 Other lower respiratory infections	
85 Pneumococcal pneumonia	

Leading causes After Redistribution	Deaths
1 Ischemic heart disease	12908.77
2 Alzheimer's disease and other dementias	8836.33
3 Tracheal, bronchus, and lung cancer	7827.14
4 Chronic obstructive pulmonary disease	6162.96
5 Ischemic stroke	5380.66
6 Colon and rectum cancer	3981.53
7 Falls	2794.11
8 Breast cancer	2710.08
9 Intracerebral hemorrhage	2640.31
10 Influenza	2624.26
11 Pancreatic cancer	2166.88
12 Self-harm by other specified means	1994.01
13 Prostate cancer	1970.84
14 Non-rheumatic calcific aortic valve disease	1941.71
15 Cirrhosis and other chronic liver diseases	1892.15
16 Other lower respiratory infections	1698.12
17 Atrial fibrillation and flutter	1529.43
18 Diabetes mellitus type 2	1473.75
19 Pneumococcal pneumonia	1339.84
20 Stomach cancer	1286.86
21 Parkinson's disease	
22 Bladder cancer	
24 Urinary tract infections and interstitial nephritis	
25 Esophageal cancer	

Leading causes of death before and after garbage code redistribution: Bulgaria - 2015.

Causes are connected by arrows before and after redistribution. Infectious diseases are shown in red, non-communicable causes in blue, and injuries in green.
In addition to garbage redistribution, the diagram also reflects the deaths moved during misassignment correction for Alzheimer's disease and other dementias.

Leading causes Before Redistribution	Deaths
1 Ischemic heart disease	13046.0
2 Ischemic stroke	9366.0
3 Hypertensive heart disease	5789.0
4 Tracheal, bronchus, and lung cancer	3457.0
5 Colon and rectum cancer	2508.0
6 Intracerebral hemorrhage	2345.0
7 Cirrhosis and other chronic liver diseases	1781.0
8 Chronic obstructive pulmonary disease	1399.0
9 Breast cancer	1345.0
10 Stomach cancer	1173.0
11 Pancreatic cancer	1105.0
12 Prostate cancer	972.0
13 Chronic kidney disease	889.0
14 Brain and central nervous system cancer	735.0
15 Self-harm by other specified means	625.0
16 Atrial fibrillation and flutter	610.0
17 Bladder cancer	577.0
18 Ovarian cancer	439.0
19 Diabetes mellitus type 2	428.0
20 Falls	386.0
27 Parkinson's disease	
36 Other cardiovascular and circulatory diseases (internal)	
38 Alzheimer's disease and other dementias	
46 Other lower respiratory infections	

Leading causes After Redistribution	Deaths
1 Ischemic heart disease	25817.78
2 Ischemic stroke	20438.37
3 Hypertensive heart disease	13888.15
4 Intracerebral hemorrhage	5465.61
5 Tracheal, bronchus, and lung cancer	4148.37
6 Colon and rectum cancer	3271.45
7 Alzheimer's disease and other dementias	2541.84
8 Cirrhosis and other chronic liver diseases	2293.67
9 Chronic obstructive pulmonary disease	2178.65
10 Diabetes mellitus type 2	2027.22
11 Breast cancer	1593.31
12 Stomach cancer	1536.82
13 Chronic kidney disease	1396.24
14 Pancreatic cancer	1341.56
15 Other lower respiratory infections	1246.64
16 Prostate cancer	1178.77
17 Other cardiovascular and circulatory diseases (internal)	1161.86
18 Parkinson's disease	852.33
19 Brain and central nervous system cancer	843.05
20 Self-harm by other specified means	755.0
21 Bladder cancer	
23 Atrial fibrillation and flutter	
25 Falls	
26 Ovarian cancer	

Leading causes of death before and after garbage code redistribution: Bahrain - 2014.

Causes are connected by arrows before and after redistribution. Infectious diseases are shown in red, non-communicable causes in blue, and injuries in green.
In addition to garbage redistribution, the diagram also reflects the deaths moved during misassignment correction for Alzheimer's disease and other dementias.

Leading causes Before Redistribution	Deaths
1 Diabetes mellitus type 2	460.0
2 Ischemic heart disease	85.0
3 Neonatal preterm birth	63.0
4 Atrial fibrillation and flutter	48.0
5 Breast cancer	46.0
6 Cirrhosis and other chronic liver diseases	44.0
7 Colon and rectum cancer	35.0
8 Endocrine, metabolic, blood, and immune disorders	34.0
9 Tracheal, bronchus, and lung cancer	28.0
10 Sickle cell disorders	23.0
11 Other congenital birth defects	20.0
12 Chronic kidney disease	19.0
13 Chronic obstructive pulmonary disease	18.0
14 Sudden infant death syndrome	17.0
15 Pancreatic cancer	17.0
16 Prostate cancer	16.0
17 Other urinary diseases	16.0
18 Hypertensive heart disease	16.0
19 Intracerebral hemorrhage	15.0
20 Other endocrine, metabolic, blood, and immune disorders	15.0
22 Chronic kidney disease due to hypertension	
28 Ischemic stroke	
34 Self-harm by other specified means	
47 Other lower respiratory infections	
48 Motor vehicle road injuries	
54 Alzheimer's disease and other dementias	
57 Pedestrian road injuries	

Leading causes After Redistribution	Deaths
1 Diabetes mellitus type 2	558.68
2 Ischemic heart disease	379.57
3 Ischemic stroke	99.96
4 Intracerebral hemorrhage	76.85
5 Cirrhosis and other chronic liver diseases	73.33
6 Neonatal preterm birth	70.16
7 Motor vehicle road injuries	63.01
8 Breast cancer	59.48
9 Hypertensive heart disease	57.29
10 Tracheal, bronchus, and lung cancer	50.51
11 Atrial fibrillation and flutter	47.77
12 Colon and rectum cancer	47.6
13 Chronic kidney disease due to hypertension	43.46
14 Other lower respiratory infections	41.84
15 Chronic obstructive pulmonary disease	40.24
16 Endocrine, metabolic, blood, and immune disorders	39.5
17 Alzheimer's disease and other dementias	36.19
18 Self-harm by other specified means	34.31
19 Chronic kidney disease	30.03
20 Pedestrian road injuries	29.26
22 Other congenital birth defects	
23 Sickle cell disorders	
25 Pancreatic cancer	
26 Other urinary diseases	
28 Prostate cancer	
33 Other endocrine, metabolic, blood, and immune disorders	
35 Sudden infant death syndrome	

Leading causes of death before and after garbage code redistribution: Bahamas - 2014.

Causes are connected by arrows before and after redistribution. Infectious diseases are shown in red, non-communicable causes in blue, and injuries in green.
In addition to garbage redistribution, the diagram also reflects the deaths moved during misassignment correction for Alzheimer's disease and other dementias.

Leading causes Before Redistribution	Deaths
1 Ischemic heart disease	183.0
2 Hypertensive heart disease	118.0
3 Physical violence by firearm	109.0
4 HIV/AIDS resulting in other diseases	100.0
5 Breast cancer	57.0
6 Prostate cancer	46.0
7 Other cardiovascular and circulatory diseases (internal)	43.0
8 Intracerebral hemorrhage	39.0
9 Cirrhosis and other chronic liver diseases	35.0
10 Colon and rectum cancer	34.0
11 Alzheimer's disease and other dementias	28.0
12 Chronic kidney disease due to hypertension	25.0
13 Motor vehicle road injuries	24.0
14 Tracheal, bronchus, and lung cancer	22.0
15 Stomach cancer	20.0
16 Decubitus ulcer	19.0
17 Urinary tract infections and interstitial nephritis	19.0
18 Drowning	18.0
19 Cervical cancer	16.0
20 Other transport injuries	15.0
30 Diabetes mellitus type 2	
43 Ischemic stroke	
125 Other lower respiratory infections	

Leading causes After Redistribution	Deaths
1 Ischemic heart disease	228.0
2 Hypertensive heart disease	155.52
3 HIV/AIDS resulting in other diseases	113.13
4 Physical violence by firearm	110.16
5 Diabetes mellitus type 2	98.74
6 Intracerebral hemorrhage	83.45
7 Ischemic stroke	72.57
8 Breast cancer	65.9
9 Prostate cancer	60.64
10 Other cardiovascular and circulatory diseases (internal)	50.08
11 Other lower respiratory infections	49.06
12 Colon and rectum cancer	46.83
13 Cirrhosis and other chronic liver diseases	44.64
14 Chronic kidney disease due to hypertension	42.77
15 Alzheimer's disease and other dementias	39.51
16 Tracheal, bronchus, and lung cancer	29.14
17 Stomach cancer	28.5
18 Motor vehicle road injuries	27.25
19 Cervical cancer	24.28
20 Urinary tract infections and interstitial nephritis	22.63
21 Decubitus ulcer	
22 Drowning	
27 Other transport injuries	

Leading causes of death before and after garbage code redistribution: Bosnia and Herzegovina - 2014.

Causes are connected by arrows before and after redistribution. Infectious diseases are shown in red, non-communicable causes in blue, and injuries in green.
In addition to garbage redistribution, the diagram also reflects the deaths moved during misassignment correction for Alzheimer's disease and other dementias.

Leading causes Before Redistribution	Deaths
1 Ischemic heart disease	3882.0
2 Ischemic stroke	3143.0
3 Tracheal, bronchus, and lung cancer	1858.0
4 Diabetes mellitus type 2	1530.0
5 Colon and rectum cancer	909.0
6 Chronic obstructive pulmonary disease	748.0
7 Breast cancer	530.0
8 Stomach cancer	511.0
9 Pancreatic cancer	437.0
10 Cirrhosis and other chronic liver diseases	421.0
11 Intracerebral hemorrhage	420.0
12 Chronic kidney disease	397.0
13 Liver cancer	393.0
14 Prostate cancer	383.0
15 Brain and central nervous system cancer	337.0
16 Bladder cancer	213.0
17 Self-harm by other specified means	203.0
18 Ovarian cancer	192.0
19 Leukemia	187.0
20 Larynx cancer	159.0
23 Parkinson's disease	
31 Alzheimer's disease and other dementias	
33 Hypertensive heart disease	
154 Other cardiomyopathy	

Leading causes After Redistribution	Deaths
1 Ischemic heart disease	7042.03
2 Ischemic stroke	4716.4
3 Other cardiomyopathy	3291.57
4 Diabetes mellitus type 2	2378.21
5 Tracheal, bronchus, and lung cancer	2324.51
6 Colon and rectum cancer	1220.15
7 Chronic obstructive pulmonary disease	1180.94
8 Intracerebral hemorrhage	939.54
9 Alzheimer's disease and other dementias	894.92
10 Hypertensive heart disease	703.68
11 Breast cancer	659.76
12 Stomach cancer	630.29
13 Cirrhosis and other chronic liver diseases	591.34
14 Pancreatic cancer	555.18
15 Chronic kidney disease	534.49
16 Prostate cancer	483.72
17 Liver cancer	431.18
18 Brain and central nervous system cancer	417.63
19 Self-harm by other specified means	291.16
20 Parkinson's disease	278.96
21 Bladder cancer	
22 Ovarian cancer	
23 Leukemia	
28 Larynx cancer	

Leading causes of death before and after garbage code redistribution: Belarus - 2014.

Causes are connected by arrows before and after redistribution. Infectious diseases are shown in red, non-communicable causes in blue, and injuries in green.
In addition to garbage redistribution, the diagram also reflects the deaths moved during misassignment correction for Alzheimer's disease and other dementias.

Leading causes Before Redistribution	Deaths
1 Ischemic heart disease	48977.0
2 Stroke	13714.0
3 Alcohol use disorders	3061.96
4 Tracheal, bronchus, and lung cancer	2893.77
5 Colon and rectum cancer	2203.11
6 Cirrhosis and other chronic liver diseases	2135.59
7 Stomach cancer	1847.89
8 Self-harm	1742.0
9 Breast cancer	1113.48
10 Chronic respiratory diseases	1090.0
11 Transport injuries	1002.0
12 Pancreatic cancer	920.33
13 Prostate cancer	826.62
14 Fire, heat, and hot substances	804.76
15 Falls	704.0
16 Drowning	629.0
17 Other malignant neoplasms (internal)	624.25
18 Alzheimer's disease and other dementias	620.45
19 Environmental heat and cold exposure	614.82
20 Lower respiratory infections	587.54
21 Alcoholic cardiomyopathy	
57 Parkinson's disease	
105 Ischemic stroke	

Leading causes After Redistribution	Deaths
1 Ischemic heart disease	58518.82
2 Stroke	15043.97
3 Alcohol use disorders	3391.59
4 Alzheimer's disease and other dementias	3165.56
5 Tracheal, bronchus, and lung cancer	3153.59
6 Colon and rectum cancer	2521.39
7 Cirrhosis and other chronic liver diseases	2213.23
8 Stomach cancer	2090.5
9 Self-harm	1839.85
10 Breast cancer	1233.51
11 Ischemic stroke	1172.15
12 Chronic respiratory diseases	1165.2
13 Alcoholic cardiomyopathy	1035.49
14 Transport injuries	1033.42
15 Pancreatic cancer	1019.7
16 Falls	988.06
17 Fire, heat, and hot substances	946.51
18 Prostate cancer	933.1
19 Parkinson's disease	712.26
20 Drowning	702.79
21 Other malignant neoplasms (internal)	
22 Environmental heat and cold exposure	
24 Lower respiratory infections	

Leading causes of death before and after garbage code redistribution: Belize - 2015.

Causes are connected by arrows before and after redistribution. Infectious diseases are shown in red, non-communicable causes in blue, and injuries in green.
In addition to garbage redistribution, the diagram also reflects the deaths moved during misassignment correction for Alzheimer's disease and other dementias.

Leading causes Before Redistribution	Deaths
1 Ischemic heart disease	128.0
2 HIV/AIDS resulting in other diseases	109.0
3 Physical violence by firearm	75.0
4 Cirrhosis and other chronic liver diseases	49.0
5 Diabetes mellitus type 2	47.0
6 Chronic obstructive pulmonary disease	37.0
7 Neonatal preterm birth	35.0
8 Prostate cancer	30.0
9 Tracheal, bronchus, and lung cancer	26.0
10 Hypertensive heart disease	25.0
11 Physical violence by sharp object	23.0
12 Self-harm by other specified means	23.0
13 Drowning	22.0
14 Chronic kidney disease	20.0
15 Colon and rectum cancer	19.0
16 Chronic kidney disease due to diabetes mellitus type 2	18.0
17 Stomach cancer	17.0
18 Congenital heart anomalies	17.0
19 Breast cancer	17.0
20 Motor vehicle road injuries	15.0
22 Intracerebral hemorrhage	
25 Chronic kidney disease due to hypertension	
29 Alzheimer's disease and other dementias	
54 Ischemic stroke	
121 Other lower respiratory infections	

Leading causes After Redistribution	Deaths
1 Ischemic heart disease	168.13
2 Diabetes mellitus type 2	134.53
3 HIV/AIDS resulting in other diseases	115.34
4 Physical violence by firearm	76.5
5 Other lower respiratory infections	70.86
6 Intracerebral hemorrhage	62.78
7 Cirrhosis and other chronic liver diseases	60.18
8 Chronic obstructive pulmonary disease	45.94
9 Chronic kidney disease due to diabetes mellitus type 2	42.95
10 Ischemic stroke	41.39
11 Motor vehicle road injuries	39.54
12 Prostate cancer	38.28
13 Hypertensive heart disease	37.76
14 Neonatal preterm birth	35.92
15 Alzheimer's disease and other dementias	31.82
16 Tracheal, bronchus, and lung cancer	28.3
17 Colon and rectum cancer	25.97
18 Chronic kidney disease	24.64
19 Stomach cancer	24.3
20 Chronic kidney disease due to hypertension	24.1
21 Self-harm by other specified means	
22 Physical violence by sharp object	
23 Drowning	
24 Breast cancer	
26 Congenital heart anomalies	

Leading causes of death before and after garbage code redistribution: Bermuda - 2015.

Causes are connected by arrows before and after redistribution. Infectious diseases are shown in red, non-communicable causes in blue, and injuries in green.
In addition to garbage redistribution, the diagram also reflects the deaths moved during misassignment correction for Alzheimer’s disease and other dementias.

Leading causes Before Redistribution	Deaths	Leading causes After Redistribution	Deaths
1 Ischemic heart disease	70.0	1 Ischemic heart disease	85.84
2 Alzheimer's disease and other dementias	32.0	2 Tracheal, bronchus, and lung cancer	28.55
3 Tracheal, bronchus, and lung cancer	25.0	3 Diabetes mellitus type 2	27.98
4 Diabetes mellitus type 2	22.0	4 Ischemic stroke	26.16
5 Colon and rectum cancer	17.0	5 Colon and rectum cancer	20.56
6 Ischemic stroke	11.0	6 Alzheimer's disease and other dementias	18.76
7 Non-rheumatic calcific aortic valve disease	10.0	7 Prostate cancer	13.89
8 Pancreatic cancer	10.0	8 Non-rheumatic calcific aortic valve disease	11.32
9 Prostate cancer	9.0	9 Pancreatic cancer	11.22
10 Chronic kidney disease	7.0	10 Intracerebral hemorrhage	9.24
11 Breast cancer	7.0	11 Breast cancer	8.77
12 Motorcyclist road injuries	7.0	12 Other cardiomyopathy	7.86
13 Liver cancer	6.0	13 Chronic kidney disease	7.47
14 Brain and central nervous system cancer	6.0	14 Motorcyclist road injuries	7.07
15 Interstitial lung disease and pulmonary sarcoidosis	6.0	15 Interstitial lung disease and pulmonary sarcoidosis	6.65
16 Parkinson's disease	4.0	16 Brain and central nervous system cancer	6.64
17 Bladder cancer	4.0	17 Liver cancer	6.25
18 Cervical cancer	4.0	18 Parkinson's disease	6.1
19 Chronic obstructive pulmonary disease	4.0	19 Other lower respiratory infections	5.86
20 Other malignant neoplasms (internal)	4.0	20 Chronic obstructive pulmonary disease	5.77
22 Intracerebral hemorrhage		25 Bladder cancer	
66 Other lower respiratory infections		26 Cervical cancer	
78 Other cardiomyopathy		27 Other malignant neoplasms (internal)	

Leading causes of death before and after garbage code redistribution: Bolivia (Plurinational State of) - 2003.

Causes are connected by arrows before and after redistribution. Infectious diseases are shown in red, non-communicable causes in blue, and injuries in green.
In addition to garbage redistribution, the diagram also reflects the deaths moved during misassignment correction for Alzheimer's disease and other dementias.

Leading causes Before Redistribution	Deaths
1 Cirrhosis and other chronic liver diseases	432.0
2 Ischemic heart disease	373.0
3 Intracerebral hemorrhage	250.0
4 Chronic kidney disease	224.0
5 Drug-susceptible tuberculosis	204.0
6 Gallbladder and biliary tract cancer	197.0
7 Alcohol use disorders	178.0
8 Protein-energy malnutrition	169.0
9 Tracheal, bronchus, and lung cancer	153.0
10 Ischemic stroke	153.0
11 Diarrheal diseases	148.0
12 Cervical cancer	132.0
13 Neonatal preterm birth	128.0
14 Chagas disease	126.0
15 Stomach cancer	112.0
16 Breast cancer	87.0
17 Drowning	83.0
18 Interstitial lung disease and pulmonary sarcoidosis	74.0
19 Paralytic ileus and intestinal obstruction	74.0
20 Prostate cancer	72.0
29 Chronic obstructive pulmonary disease	
32 Pulmonary aspiration and foreign body in airway	
33 Diabetes mellitus type 2	
37 Other lower respiratory infections	
67 Self-harm by other specified means	
72 Physical violence by other means	
105 Pedestrian road injuries	
106 Falls	

Leading causes After Redistribution	Deaths
1 Other lower respiratory infections	1681.96
2 Ischemic heart disease	1458.86
3 Cirrhosis and other chronic liver diseases	1231.5
4 Intracerebral hemorrhage	880.47
5 Ischemic stroke	869.28
6 Chronic kidney disease	812.88
7 Stomach cancer	695.29
8 Diabetes mellitus type 2	493.04
9 Pedestrian road injuries	491.91
10 Gallbladder and biliary tract cancer	454.49
11 Drug-susceptible tuberculosis	452.97
12 Pulmonary aspiration and foreign body in airway	445.53
13 Protein-energy malnutrition	414.49
14 Tracheal, bronchus, and lung cancer	405.11
15 Physical violence by other means	396.52
16 Self-harm by other specified means	367.49
17 Diarrheal diseases	358.67
18 Cervical cancer	356.09
19 Falls	354.1
20 Chronic obstructive pulmonary disease	338.42
21 Drowning	
22 Interstitial lung disease and pulmonary sarcoidosis	
24 Alcohol use disorders	
26 Paralytic ileus and intestinal obstruction	
27 Chagas disease	
28 Prostate cancer	
31 Breast cancer	
33 Neonatal preterm birth	

Leading causes of death before and after garbage code redistribution: Brazil - 2015.

Causes are connected by arrows before and after redistribution. Infectious diseases are shown in red, non-communicable causes in blue, and injuries in green.
In addition to garbage redistribution, the diagram also reflects the deaths moved during misassignment correction for Alzheimer's disease and other dementias.

Leading causes Before Redistribution	Deaths
1 Ischemic heart disease	110961.0
2 Chronic obstructive pulmonary disease	42041.0
3 Physical violence by firearm	40456.0
4 Tracheal, bronchus, and lung cancer	26582.0
5 Cirrhosis and other chronic liver diseases	23206.0
6 Alzheimer's disease and other dementias	21284.0
7 Urinary tract infections and interstitial nephritis	17720.0
8 Hypertensive heart disease	16836.0
9 Colon and rectum cancer	16684.0
10 Breast cancer	15605.0
11 Intracerebral hemorrhage	15519.0
12 Prostate cancer	14451.0
13 Stomach cancer	14248.0
14 Falls	12395.0
15 Motorcyclist road injuries	11508.0
16 HIV/AIDS resulting in other diseases	10989.0
17 Motor vehicle road injuries	9941.0
18 Pancreatic cancer	9440.0
19 Self-harm by other specified means	9426.0
20 Brain and central nervous system cancer	9180.0
31 Ischemic stroke	
36 Diabetes mellitus type 2	
54 Other cardiomyopathy	
89 Other lower respiratory infections	

Leading causes After Redistribution	Deaths
1 Ischemic heart disease	145018.61
2 Ischemic stroke	64893.2
3 Other lower respiratory infections	64271.91
4 Chronic obstructive pulmonary disease	58551.18
5 Diabetes mellitus type 2	53297.31
6 Alzheimer's disease and other dementias	52673.3
7 Physical violence by firearm	47123.47
8 Intracerebral hemorrhage	40999.3
9 Tracheal, bronchus, and lung cancer	33109.7
10 Cirrhosis and other chronic liver diseases	31607.64
11 Hypertensive heart disease	25988.96
12 Colon and rectum cancer	23078.47
13 Stomach cancer	22164.52
14 Urinary tract infections and interstitial nephritis	20682.26
15 Breast cancer	19130.18
16 Prostate cancer	18734.28
17 Falls	17772.69
18 Other cardiomyopathy	16714.99
19 Motorcyclist road injuries	15709.7
20 Motor vehicle road injuries	13913.08
21 HIV/AIDS resulting in other diseases	
23 Pancreatic cancer	
24 Self-harm by other specified means	
27 Brain and central nervous system cancer	

Leading causes of death before and after garbage code redistribution: Barbados - 2013.

Causes are connected by arrows before and after redistribution. Infectious diseases are shown in red, non-communicable causes in blue, and injuries in green.
In addition to garbage redistribution, the diagram also reflects the deaths moved during misassignment correction for Alzheimer's disease and other dementias.

Leading causes Before Redistribution	Deaths
1 Ischemic heart disease	202.0
2 Prostate cancer	97.0
3 Colon and rectum cancer	79.0
4 Alzheimer's disease and other dementias	63.0
5 Breast cancer	55.0
6 Decubitus ulcer	41.0
7 Urinary tract infections and interstitial nephritis	39.0
8 Intracerebral hemorrhage	36.0
9 Hypertensive heart disease	36.0
10 Diabetes mellitus type 2	32.0
11 Pancreatic cancer	31.0
12 Cervical cancer	28.0
13 Tracheal, bronchus, and lung cancer	27.0
14 Cirrhosis and other chronic liver diseases	17.0
15 Atrial fibrillation and flutter	16.0
16 Endocrine, metabolic, blood, and immune disorders	16.0
17 Multiple myeloma	16.0
18 Subarachnoid hemorrhage	16.0
19 Asthma	15.0
20 Other cardiovascular and circulatory diseases (internal)	15.0
21 Stomach cancer	
22 Chronic kidney disease due to hypertension	
30 Ischemic stroke	
97 Other lower respiratory infections	

Leading causes After Redistribution	Deaths
1 Ischemic heart disease	246.91
2 Diabetes mellitus type 2	221.34
3 Ischemic stroke	173.93
4 Prostate cancer	125.36
5 Colon and rectum cancer	104.54
6 Other lower respiratory infections	102.88
7 Intracerebral hemorrhage	96.8
8 Breast cancer	73.37
9 Alzheimer's disease and other dementias	68.08
10 Decubitus ulcer	53.96
11 Urinary tract infections and interstitial nephritis	53.73
12 Hypertensive heart disease	50.43
13 Pancreatic cancer	37.58
14 Cervical cancer	35.01
15 Tracheal, bronchus, and lung cancer	34.8
16 Chronic kidney disease due to hypertension	34.31
17 Other cardiovascular and circulatory diseases (internal)	30.17
18 Stomach cancer	27.61
19 Cirrhosis and other chronic liver diseases	26.45
20 Atrial fibrillation and flutter	25.34
22 Subarachnoid hemorrhage	
23 Multiple myeloma	
33 Endocrine, metabolic, blood, and immune disorders	
38 Asthma	

Leading causes of death before and after garbage code redistribution: Brunei Darussalam - 2015.

Causes are connected by arrows before and after redistribution. Infectious diseases are shown in red, non-communicable causes in blue, and injuries in green.
In addition to garbage redistribution, the diagram also reflects the deaths moved during misassignment correction for Alzheimer's disease and other dementias.

Leading causes Before Redistribution	Deaths
1 Ischemic heart disease	143.0
2 Tracheal, bronchus, and lung cancer	52.0
3 Colon and rectum cancer	43.0
4 Intracerebral hemorrhage	42.0
5 Breast cancer	33.0
6 Chronic obstructive pulmonary disease	30.0
7 Chronic kidney disease due to hypertension	28.0
8 Liver cancer	28.0
9 Diabetes mellitus type 2	25.0
10 Alzheimer's disease and other dementias	24.0
11 Cirrhosis and other chronic liver diseases	23.0
12 Neonatal preterm birth	20.0
13 Ischemic stroke	17.0
14 Drowning	17.0
15 Endocrine, metabolic, blood, and immune disorders	16.0
16 Chronic kidney disease	15.0
17 Drug-susceptible tuberculosis	15.0
18 Parkinson's disease	14.0
19 Asthma	13.0
20 Ovarian cancer	12.0
21 Other non-Hodgkin lymphoma	
26 Stomach cancer	
37 Falls	
111 Other lower respiratory infections	

Leading causes After Redistribution	Deaths
1 Ischemic heart disease	168.75
2 Diabetes mellitus type 2	151.1
3 Other lower respiratory infections	68.9
4 Tracheal, bronchus, and lung cancer	66.47
5 Intracerebral hemorrhage	65.88
6 Colon and rectum cancer	55.47
7 Ischemic stroke	52.85
8 Chronic obstructive pulmonary disease	46.5
9 Chronic kidney disease due to hypertension	38.98
10 Breast cancer	37.79
11 Cirrhosis and other chronic liver diseases	36.08
12 Liver cancer	32.68
13 Falls	22.08
14 Other non-Hodgkin lymphoma	21.07
15 Neonatal preterm birth	20.94
16 Chronic kidney disease	20.32
17 Stomach cancer	18.91
18 Drowning	18.58
19 Alzheimer's disease and other dementias	18.49
20 Endocrine, metabolic, blood, and immune disorders	17.64
21 Drug-susceptible tuberculosis	
23 Parkinson's disease	
26 Asthma	
28 Ovarian cancer	

Leading causes of death before and after garbage code redistribution: Canada - 2015.

Causes are connected by arrows before and after redistribution. Infectious diseases are shown in red, non-communicable causes in blue, and injuries in green.
In addition to garbage redistribution, the diagram also reflects the deaths moved during misassignment correction for Alzheimer's disease and other dementias.

Leading causes Before Redistribution	Deaths	Leading causes After Redistribution	Deaths
1 Ischemic heart disease	34030.0	1 Ischemic heart disease	41297.08
2 Alzheimer's disease and other dementias	24852.0	2 Tracheal, bronchus, and lung cancer	22271.33
3 Tracheal, bronchus, and lung cancer	20111.0	3 Alzheimer's disease and other dementias	16373.66
4 Chronic obstructive pulmonary disease	12004.0	4 Chronic obstructive pulmonary disease	14322.76
5 Colon and rectum cancer	8492.0	5 Colon and rectum cancer	10490.17
6 Breast cancer	5188.0	6 Ischemic stroke	10203.87
7 Pancreatic cancer	4764.0	7 Influenza	6974.88
8 Falls	4711.0	8 Falls	6348.82
9 Prostate cancer	4013.0	9 Breast cancer	5993.2
10 Cirrhosis and other chronic liver diseases	3833.0	10 Pancreatic cancer	5518.17
11 Self-harm by other specified means	3740.0	11 Diabetes mellitus type 2	5486.97
12 Intracerebral hemorrhage	2786.0	12 Prostate cancer	4906.84
13 Atrial fibrillation and flutter	2765.0	13 Cirrhosis and other chronic liver diseases	4663.72
14 Parkinson's disease	2518.0	14 Intracerebral hemorrhage	4396.71
15 Interstitial lung disease and pulmonary sarcoidosis	2445.0	15 Self-harm by other specified means	4254.35
16 Liver cancer	2408.0	16 Parkinson's disease	3838.52
17 Brain and central nervous system cancer	2226.0	17 Other non-Hodgkin lymphoma	3288.15
18 Bladder cancer	2156.0	18 Atrial fibrillation and flutter	3200.0
19 Esophageal cancer	1956.0	19 Stomach cancer	2858.08
20 Non-rheumatic calcific aortic valve disease	1945.0	20 Interstitial lung disease and pulmonary sarcoidosis	2728.45
22 Stomach cancer		21 Liver cancer	
25 Influenza		22 Bladder cancer	
27 Diabetes mellitus type 2		23 Non-rheumatic calcific aortic valve disease	
43 Ischemic stroke		24 Brain and central nervous system cancer	
48 Other non-Hodgkin lymphoma		25 Esophageal cancer	

Leading causes of death before and after garbage code redistribution: Switzerland - 2015.

Causes are connected by arrows before and after redistribution. Infectious diseases are shown in red, non-communicable causes in blue, and injuries in green.
In addition to garbage redistribution, the diagram also reflects the deaths moved during misassignment correction for Alzheimer’s disease and other dementias.

Leading causes Before Redistribution	Deaths
1 Ischemic heart disease	7373.0
2 Alzheimer's disease and other dementias	6552.0
3 Tracheal, bronchus, and lung cancer	3276.0
4 Chronic obstructive pulmonary disease	1992.0
5 Colon and rectum cancer	1789.0
6 Falls	1735.0
7 Hypertensive heart disease	1491.0
8 Breast cancer	1432.0
9 Prostate cancer	1359.0
10 Pancreatic cancer	1273.0
11 Chronic kidney disease due to hypertension	874.0
12 Self-harm by other specified means	843.0
13 Ischemic stroke	833.0
14 Parkinson's disease	790.0
15 Endocarditis	693.0
16 Cirrhosis and other chronic liver diseases	693.0
17 Liver cancer	684.0
18 Atrial fibrillation and flutter	621.0
19 Intracerebral hemorrhage	593.0
20 Bladder cancer	568.0
21 Diabetes mellitus type 2	
42 Influenza	

Leading causes After Redistribution	Deaths
1 Ischemic heart disease	10283.31
2 Alzheimer's disease and other dementias	5772.19
3 Tracheal, bronchus, and lung cancer	3754.32
4 Ischemic stroke	3104.79
5 Chronic obstructive pulmonary disease	2503.38
6 Hypertensive heart disease	2353.16
7 Colon and rectum cancer	2224.63
8 Falls	2146.11
9 Breast cancer	1655.53
10 Prostate cancer	1625.34
11 Pancreatic cancer	1497.46
12 Chronic kidney disease due to hypertension	1487.76
13 Diabetes mellitus type 2	1239.14
14 Influenza	1087.03
15 Intracerebral hemorrhage	1027.03
16 Parkinson's disease	958.5
17 Self-harm by other specified means	958.37
18 Endocarditis	922.83
19 Cirrhosis and other chronic liver diseases	900.02
20 Liver cancer	780.6
23 Atrial fibrillation and flutter	
24 Bladder cancer	

Leading causes of death before and after garbage code redistribution: Chile - 2015.

Causes are connected by arrows before and after redistribution. Infectious diseases are shown in red, non-communicable causes in blue, and injuries in green.
In addition to garbage redistribution, the diagram also reflects the deaths moved during misassignment correction for Alzheimer's disease and other dementias.

Leading causes Before Redistribution	Deaths	Leading causes After Redistribution	Deaths
1 Ischemic heart disease	8390.0	1 Ischemic heart disease	10027.36
2 Stomach cancer	3389.0	2 Ischemic stroke	5916.51
3 Cirrhosis and other chronic liver diseases	3352.0	3 Cirrhosis and other chronic liver diseases	4718.54
4 Alzheimer's disease and other dementias	3255.0	4 Stomach cancer	4085.48
5 Tracheal, bronchus, and lung cancer	3191.0	5 Alzheimer's disease and other dementias	4015.09
6 Chronic obstructive pulmonary disease	3156.0	6 Chronic obstructive pulmonary disease	3774.23
7 Colon and rectum cancer	2622.0	7 Diabetes mellitus type 2	3595.27
8 Hypertensive heart disease	2452.0	8 Tracheal, bronchus, and lung cancer	3543.98
9 Prostate cancer	2141.0	9 Hypertensive heart disease	3230.3
10 Intracerebral hemorrhage	1813.0	10 Colon and rectum cancer	3168.66
11 Diabetes mellitus type 2	1790.0	11 Intracerebral hemorrhage	3064.14
12 Self-harm by other specified means	1722.0	12 Prostate cancer	2461.54
13 Gallbladder and biliary tract cancer	1660.0	13 Chronic kidney disease due to hypertension	1955.91
14 Interstitial lung disease and pulmonary sarcoidosis	1536.0	14 Gallbladder and biliary tract cancer	1928.38
15 Breast cancer	1524.0	15 Self-harm by other specified means	1811.0
16 Pancreatic cancer	1349.0	16 Breast cancer	1742.71
17 Urinary tract infections and interstitial nephritis	1291.0	17 Interstitial lung disease and pulmonary sarcoidosis	1739.52
18 Chronic kidney disease	1250.0	18 Pancreatic cancer	1544.89
19 Chronic kidney disease due to hypertension	1187.0	19 Other lower respiratory infections	1480.2
20 Atrial fibrillation and flutter	990.0	20 Chronic kidney disease	1456.05
35 Ischemic stroke		21 Urinary tract infections and interstitial nephritis	
95 Other lower respiratory infections		28 Atrial fibrillation and flutter	

Leading causes of death before and after garbage code redistribution: China - 2015.

Causes are connected by arrows before and after redistribution. Infectious diseases are shown in red, non-communicable causes in blue, and injuries in green.
In addition to garbage redistribution, the diagram also reflects the deaths moved during misassignment correction for Alzheimer's disease and other dementias.

Leading causes Before Redistribution	Deaths
1 Ischemic heart disease	1000566.0
2 Intracerebral hemorrhage	534843.0
3 Ischemic stroke	526198.0
4 Chronic obstructive pulmonary disease	500189.0
5 Tracheal, bronchus, and lung cancer	409319.0
6 Stomach cancer	196008.0
7 Hypertensive heart disease	193116.0
8 Esophageal cancer	114787.0
9 Colon and rectum cancer	96849.0
10 Liver cancer	95472.0
11 Pedestrian road injuries	92201.0
12 Falls	80070.0
13 Cirrhosis and other chronic liver diseases	79048.0
14 Self-harm by other specified means	61169.0
15 Subarachnoid hemorrhage	58317.0
16 Diabetes mellitus type 2	56892.0
17 Alzheimer's disease and other dementias	45455.0
18 Pancreatic cancer	44365.0
19 Rheumatic heart disease	40443.0
20 Breast cancer	35555.0
31 Other lower respiratory infections	

Leading causes After Redistribution	Deaths
1 Ischemic heart disease	1062231.77
2 Intracerebral hemorrhage	620339.94
3 Ischemic stroke	567854.11
4 Chronic obstructive pulmonary disease	557153.03
5 Tracheal, bronchus, and lung cancer	431031.98
6 Stomach cancer	249146.26
7 Hypertensive heart disease	213864.75
8 Alzheimer's disease and other dementias	206092.38
9 Esophageal cancer	146028.12
10 Colon and rectum cancer	139453.95
11 Liver cancer	122691.3
12 Pedestrian road injuries	101628.55
13 Diabetes mellitus type 2	94864.65
14 Falls	87546.51
15 Cirrhosis and other chronic liver diseases	87251.72
16 Other lower respiratory infections	78062.97
17 Self-harm by other specified means	67020.51
18 Subarachnoid hemorrhage	60543.87
19 Pancreatic cancer	59502.78
20 Breast cancer	48675.56
21 Rheumatic heart disease	

Leading causes of death before and after garbage code redistribution: Colombia - 2015.

Causes are connected by arrows before and after redistribution. Infectious diseases are shown in red, non-communicable causes in blue, and injuries in green.

In addition to garbage redistribution, the diagram also reflects the deaths moved during misassignment correction for Alzheimer's disease and other dementias.

Leading causes Before Redistribution	Deaths
1 Ischemic heart disease	36192.0
2 Chronic obstructive pulmonary disease	13125.0
3 Physical violence by firearm	8874.0
4 Stomach cancer	5217.0
5 Tracheal, bronchus, and lung cancer	5049.0
6 Hypertensive heart disease	3661.0
7 Colon and rectum cancer	3572.0
8 Motorcyclist road injuries	3139.0
9 Chronic kidney disease	3107.0
10 Intracerebral hemorrhage	2999.0
11 Prostate cancer	2938.0
12 Breast cancer	2895.0
13 Physical violence by sharp object	2721.0
14 Cirrhosis and other chronic liver diseases	2716.0
15 Urinary tract infections and interstitial nephritis	2509.0
16 Chronic kidney disease due to hypertension	2342.0
17 Subarachnoid hemorrhage	2335.0
18 HIV/AIDS resulting in other diseases	2160.0
19 Self-harm by other specified means	2016.0
20 Pedestrian road injuries	1827.0
25 Ischemic stroke	
28 Alzheimer's disease and other dementias	
29 Diabetes mellitus type 2	
97 Other lower respiratory infections	

Leading causes After Redistribution	Deaths
1 Ischemic heart disease	38875.44
2 Chronic obstructive pulmonary disease	13786.17
3 Physical violence by firearm	9468.19
4 Alzheimer's disease and other dementias	8524.76
5 Ischemic stroke	7119.07
6 Stomach cancer	6767.81
7 Other lower respiratory infections	6117.65
8 Diabetes mellitus type 2	5687.51
9 Tracheal, bronchus, and lung cancer	5657.79
10 Intracerebral hemorrhage	4753.39
11 Colon and rectum cancer	4478.85
12 Hypertensive heart disease	4230.0
13 Prostate cancer	3422.17
14 Motorcyclist road injuries	3393.62
15 Chronic kidney disease	3335.44
16 Breast cancer	3254.32
17 Subarachnoid hemorrhage	3100.88
18 Cirrhosis and other chronic liver diseases	2969.58
19 Chronic kidney disease due to hypertension	2907.05
20 Physical violence by sharp object	2874.28
21 Pedestrian road injuries	
22 HIV/AIDS resulting in other diseases	
23 Self-harm by other specified means	
24 Urinary tract infections and interstitial nephritis	

Leading causes of death before and after garbage code redistribution: Cabo Verde - 2012.

Causes are connected by arrows before and after redistribution. Infectious diseases are shown in red, non-communicable causes in blue, and injuries in green.
In addition to garbage redistribution, the diagram also reflects the deaths moved during misassignment correction for Alzheimer's disease and other dementias.

Leading causes Before Redistribution	Deaths	Leading causes After Redistribution	Deaths
1 Ischemic heart disease	124.0	1 Ischemic heart disease	244.44
2 Neonatal encephalopathy due to birth asphyxia and trauma	100.0	2 Intracerebral hemorrhage	232.86
3 Neonatal preterm birth	87.0	3 Other lower respiratory infections	188.82
4 Alcohol use disorders	81.0	4 Ischemic stroke	140.85
5 HIV/AIDS resulting in other diseases	79.0	5 Neonatal encephalopathy due to birth asphyxia and trauma	103.56
6 Stomach cancer	52.0	6 HIV/AIDS resulting in other diseases	98.15
7 Prostate cancer	46.0	7 Diabetes mellitus type 2	93.36
8 Cirrhosis and other chronic liver diseases	42.0	8 Neonatal preterm birth	92.1
9 Intracerebral hemorrhage	41.0	9 Alcohol use disorders	84.42
10 Self-harm by other specified means	40.0	10 Cirrhosis and other chronic liver diseases	72.52
11 Neonatal sepsis and other neonatal infections	40.0	11 Stomach cancer	68.96
12 Esophageal cancer	35.0	12 Prostate cancer	62.9
13 Tracheal, bronchus, and lung cancer	32.0	13 Alzheimer's disease and other dementias	62.89
14 Liver cancer	32.0	14 Self-harm by other specified means	59.84
15 Drowning	31.0	15 Hypertensive heart disease	51.07
16 Chronic kidney disease	28.0	16 Chronic kidney disease	50.19
17 Physical violence by firearm	27.0	17 Diarrheal diseases	47.44
18 Diarrheal diseases	24.0	18 Chronic obstructive pulmonary disease	44.49
19 Other congenital birth defects	23.0	19 Tracheal, bronchus, and lung cancer	44.07
20 Cervical cancer	20.0	20 Neonatal sepsis and other neonatal infections	43.36
27 Ischemic stroke		21 Esophageal cancer	
31 Hypertensive heart disease		22 Drowning	
33 Other lower respiratory infections		23 Liver cancer	
35 Chronic obstructive pulmonary disease		24 Physical violence by firearm	
43 Alzheimer's disease and other dementias		25 Cervical cancer	
98 Diabetes mellitus type 2		30 Other congenital birth defects	

Leading causes of death before and after garbage code redistribution: Costa Rica - 2015.

Causes are connected by arrows before and after redistribution. Infectious diseases are shown in red, non-communicable causes in blue, and injuries in green.
In addition to garbage redistribution, the diagram also reflects the deaths moved during misassignment correction for Alzheimer's disease and other dementias.

Leading causes Before Redistribution	Deaths	Leading causes After Redistribution	Deaths
1 Ischemic heart disease	2749.0	1 Ischemic heart disease	2991.06
2 Chronic obstructive pulmonary disease	776.0	2 Chronic obstructive pulmonary disease	908.6
3 Stomach cancer	666.0	3 Stomach cancer	851.76
4 Colon and rectum cancer	536.0	4 Alzheimer's disease and other dementias	821.29
5 Cirrhosis and other chronic liver diseases	521.0	5 Cirrhosis and other chronic liver diseases	742.26
6 Alzheimer's disease and other dementias	451.0	6 Ischemic stroke	709.43
7 Prostate cancer	392.0	7 Colon and rectum cancer	673.09
8 Physical violence by firearm	372.0	8 Diabetes mellitus type 2	565.55
9 Chronic kidney disease	346.0	9 Other lower respiratory infections	479.53
10 Hypertensive heart disease	331.0	10 Prostate cancer	478.4
11 Tracheal, bronchus, and lung cancer	330.0	11 Pedestrian road injuries	468.96
12 Breast cancer	327.0	12 Chronic kidney disease due to diabetes mellitus type 2	442.32
13 Self-harm by other specified means	282.0	13 Physical violence by firearm	404.21
14 Diabetes mellitus type 2	251.0	14 Breast cancer	389.82
15 Chronic kidney disease due to hypertension	241.0	15 Tracheal, bronchus, and lung cancer	388.22
16 Pancreatic cancer	234.0	16 Hypertensive heart disease	387.1
17 Liver cancer	211.0	17 Chronic kidney disease	374.7
18 Intracerebral hemorrhage	210.0	18 Intracerebral hemorrhage	362.45
19 Chronic kidney disease due to diabetes mellitus type 2	206.0	19 Falls	320.13
20 Urinary tract infections and interstitial nephritis	201.0	20 Self-harm by other specified means	307.73
31 Ischemic stroke		21 Chronic kidney disease due to hypertension	
35 Falls		22 Pancreatic cancer	
42 Pedestrian road injuries		23 Liver cancer	
108 Other lower respiratory infections		27 Urinary tract infections and interstitial nephritis	

Leading causes of death before and after garbage code redistribution: Cuba - 2015.

Causes are connected by arrows before and after redistribution. Infectious diseases are shown in red, non-communicable causes in blue, and injuries in green.
In addition to garbage redistribution, the diagram also reflects the deaths moved during misassignment correction for Alzheimer’s disease and other dementias.

Leading causes Before Redistribution	Deaths
1 Ischemic heart disease	17022.0
2 Tracheal, bronchus, and lung cancer	5550.0
3 Alzheimer's disease and other dementias	5053.0
4 Ischemic stroke	4880.0
5 Chronic obstructive pulmonary disease	3815.0
6 Prostate cancer	2995.0
7 Colon and rectum cancer	2701.0
8 Falls	2487.0
9 Intracerebral hemorrhage	2288.0
10 Hypertensive heart disease	1979.0
11 Cirrhosis and other chronic liver diseases	1704.0
12 Breast cancer	1569.0
13 Self-harm by other specified means	1493.0
14 Diabetes mellitus type 2	1127.0
15 Stomach cancer	917.0
16 Larynx cancer	898.0
17 Chronic kidney disease due to hypertension	869.0
18 Pancreatic cancer	861.0
19 Esophageal cancer	788.0
20 Bladder cancer	679.0
61 Atrial fibrillation and flutter	
96 Other lower respiratory infections	

Leading causes After Redistribution	Deaths
1 Ischemic heart disease	20097.98
2 Ischemic stroke	6875.1
3 Tracheal, bronchus, and lung cancer	5904.75
4 Other lower respiratory infections	5449.43
5 Chronic obstructive pulmonary disease	4371.92
6 Falls	3525.66
7 Prostate cancer	3314.25
8 Alzheimer's disease and other dementias	3202.35
9 Colon and rectum cancer	3101.45
10 Intracerebral hemorrhage	3027.39
11 Hypertensive heart disease	2427.46
12 Cirrhosis and other chronic liver diseases	1852.71
13 Breast cancer	1755.71
14 Diabetes mellitus type 2	1692.97
15 Self-harm by other specified means	1667.85
16 Chronic kidney disease due to hypertension	1082.13
17 Stomach cancer	1068.83
18 Pancreatic cancer	967.78
19 Larynx cancer	936.26
20 Atrial fibrillation and flutter	926.68
22 Esophageal cancer	
24 Bladder cancer	

Leading causes of death before and after garbage code redistribution: Cyprus - 2015.

Causes are connected by arrows before and after redistribution. Infectious diseases are shown in red, non-communicable causes in blue, and injuries in green.
In addition to garbage redistribution, the diagram also reflects the deaths moved during misassignment correction for Alzheimer’s disease and other dementias.

Leading causes Before Redistribution	Deaths
1 Ischemic heart disease	669.0
2 Alzheimer's disease and other dementias	272.0
3 Tracheal, bronchus, and lung cancer	237.0
4 Chronic obstructive pulmonary disease	118.0
5 Colon and rectum cancer	118.0
6 Breast cancer	114.0
7 Hypertensive heart disease	100.0
8 Prostate cancer	100.0
9 Atrial fibrillation and flutter	98.0
10 Intracerebral hemorrhage	93.0
11 Pancreatic cancer	85.0
12 Urinary tract infections and interstitial nephritis	83.0
13 Non-rheumatic calcific aortic valve disease	69.0
14 Stomach cancer	61.0
15 Chronic kidney disease	55.0
16 Cirrhosis and other chronic liver diseases	55.0
17 Bladder cancer	54.0
18 Aortic aneurysm	51.0
19 Brain and central nervous system cancer	49.0
20 Diarrheal diseases	38.0
22 Chronic kidney disease due to hypertension	
29 Diabetes mellitus type 2	
36 Falls	
64 Ischemic stroke	
135 Other lower respiratory infections	

Leading causes After Redistribution	Deaths
1 Ischemic heart disease	914.23
2 Chronic obstructive pulmonary disease	377.79
3 Diabetes mellitus type 2	326.5
4 Ischemic stroke	287.27
5 Tracheal, bronchus, and lung cancer	282.29
6 Alzheimer's disease and other dementias	246.71
7 Hypertensive heart disease	173.74
8 Colon and rectum cancer	173.06
9 Intracerebral hemorrhage	163.13
10 Breast cancer	135.22
11 Prostate cancer	126.06
12 Pancreatic cancer	113.0
13 Urinary tract infections and interstitial nephritis	103.32
14 Non-rheumatic calcific aortic valve disease	99.6
15 Atrial fibrillation and flutter	98.47
16 Stomach cancer	98.31
17 Falls	84.93
18 Chronic kidney disease due to hypertension	83.73
19 Other lower respiratory infections	76.76
20 Chronic kidney disease	75.38
22 Cirrhosis and other chronic liver diseases	
24 Bladder cancer	
26 Brain and central nervous system cancer	
27 Aortic aneurysm	
29 Diarrheal diseases	

Leading causes of death before and after garbage code redistribution: Czechia - 2015.

Causes are connected by arrows before and after redistribution. Infectious diseases are shown in red, non-communicable causes in blue, and injuries in green.
In addition to garbage redistribution, the diagram also reflects the deaths moved during misassignment correction for Alzheimer’s disease and other dementias.

Leading causes Before Redistribution	Deaths	Leading causes After Redistribution	Deaths
1 Ischemic heart disease	26659.0	1 Ischemic heart disease	31532.9
2 Tracheal, bronchus, and lung cancer	5315.0	2 Ischemic stroke	8298.19
3 Ischemic stroke	4562.0	3 Tracheal, bronchus, and lung cancer	5943.08
4 Colon and rectum cancer	3600.0	4 Colon and rectum cancer	4381.62
5 Chronic obstructive pulmonary disease	3500.0	5 Alzheimer's disease and other dementias	4305.4
6 Alzheimer's disease and other dementias	2906.0	6 Chronic obstructive pulmonary disease	3984.61
7 Diabetes mellitus type 2	2615.0	7 Diabetes mellitus type 2	3679.31
8 Pancreatic cancer	2021.0	8 Pancreatic cancer	2346.91
9 Cirrhosis and other chronic liver diseases	1803.0	9 Other lower respiratory infections	2324.57
10 Breast cancer	1623.0	10 Cirrhosis and other chronic liver diseases	2299.98
11 Intracerebral hemorrhage	1408.0	11 Intracerebral hemorrhage	1995.81
12 Hypertensive heart disease	1369.0	12 Falls	1989.5
13 Prostate cancer	1328.0	13 Breast cancer	1853.8
14 Self-harm by other specified means	1226.0	14 Hypertensive heart disease	1738.3
15 Kidney cancer	1165.0	15 Prostate cancer	1583.65
16 Stomach cancer	1042.0	16 Self-harm by other specified means	1365.86
17 Bladder cancer	819.0	17 Kidney cancer	1313.11
18 Atrial fibrillation and flutter	811.0	18 Stomach cancer	1240.01
19 Ovarian cancer	725.0	19 Parkinson's disease	1013.68
20 Brain and central nervous system cancer	686.0	20 Other cardiovascular and circulatory diseases (internal)	982.52
22 Other cardiovascular and circulatory diseases (internal)		21 Atrial fibrillation and flutter	
23 Falls		22 Bladder cancer	
35 Other lower respiratory infections		23 Ovarian cancer	
44 Parkinson's disease		25 Brain and central nervous system cancer	

Leading causes of death before and after garbage code redistribution: Germany - 2015.

Causes are connected by arrows before and after redistribution. Infectious diseases are shown in red, non-communicable causes in blue, and injuries in green.
In addition to garbage redistribution, the diagram also reflects the deaths moved during misassignment correction for Alzheimer's disease and other dementias.

Leading causes Before Redistribution	Deaths	Leading causes After Redistribution	Deaths
1 Ischemic heart disease	128228.0	1 Ischemic heart disease	161650.27
2 Tracheal, bronchus, and lung cancer	45590.0	2 Alzheimer's disease and other dementias	66568.2
3 Alzheimer's disease and other dementias	44075.0	3 Tracheal, bronchus, and lung cancer	52435.83
4 Chronic obstructive pulmonary disease	33310.0	4 Ischemic stroke	45649.57
5 Colon and rectum cancer	25636.0	5 Chronic obstructive pulmonary disease	39687.31
6 Hypertensive heart disease	25363.0	6 Hypertensive heart disease	36053.03
7 Atrial fibrillation and flutter	19262.0	7 Colon and rectum cancer	31948.17
8 Ischemic stroke	18405.0	8 Breast cancer	20993.72
9 Breast cancer	18315.0	9 Diabetes mellitus type 2	19975.94
10 Pancreatic cancer	17161.0	10 Pancreatic cancer	19867.51
11 Cirrhosis and other chronic liver diseases	15095.0	11 Atrial fibrillation and flutter	18508.21
12 Prostate cancer	13980.0	12 Cirrhosis and other chronic liver diseases	18221.85
13 Falls	12867.0	13 Falls	17189.53
14 Chronic kidney disease	10408.0	14 Prostate cancer	16655.43
15 Parkinson's disease	10229.0	15 Intracerebral hemorrhage	15551.44
16 Non-rheumatic calcific aortic valve disease	9579.0	16 Chronic kidney disease due to hypertension	14908.53
17 Intracerebral hemorrhage	9523.0	17 Non-rheumatic calcific aortic valve disease	13579.37
18 Stomach cancer	9356.0	18 Chronic kidney disease	13127.5
19 Chronic kidney disease due to hypertension	9183.0	19 Other lower respiratory infections	12818.03
20 Self-harm by other specified means	9023.0	20 Stomach cancer	12651.25
21 Diabetes mellitus type 2		21 Parkinson's disease	
80 Other lower respiratory infections		22 Self-harm by other specified means	

Leading causes of death before and after garbage code redistribution: Dominica - 2015.

Causes are connected by arrows before and after redistribution. Infectious diseases are shown in red, non-communicable causes in blue, and injuries in green.
In addition to garbage redistribution, the diagram also reflects the deaths moved during misassignment correction for Alzheimer’s disease and other dementias.

Leading causes Before Redistribution	Deaths	Leading causes After Redistribution	Deaths
1 Prostate cancer	39.0	1 Ischemic heart disease	69.86
2 Ischemic heart disease	37.0	2 Prostate cancer	45.23
3 Neonatal encephalopathy due to birth asphyxia and trauma	11.0	3 Ischemic stroke	43.96
4 Hypertensive heart disease	11.0	4 Diabetes mellitus type 2	42.55
5 Tracheal, bronchus, and lung cancer	9.0	5 Other cardiomyopathy	31.05
6 Chronic kidney disease due to hypertension	9.0	6 Intracerebral hemorrhage	22.67
7 Colon and rectum cancer	9.0	7 Other lower respiratory infections	20.34
8 Chronic obstructive pulmonary disease	8.0	8 Hypertensive heart disease	19.18
9 Other cardiomyopathy	8.0	9 Chronic kidney disease due to hypertension	16.17
10 Breast cancer	8.0	10 Colon and rectum cancer	14.28
11 Pancreatic cancer	8.0	11 Alzheimer's disease and other dementias	14.06
12 Alzheimer's disease and other dementias	7.0	12 Neonatal encephalopathy due to birth asphyxia and trauma	13.1
13 Neonatal preterm birth	6.0	13 Tracheal, bronchus, and lung cancer	11.51
14 Exposure to forces of nature	6.0	14 Chronic obstructive pulmonary disease	11.11
15 Stomach cancer	6.0	15 Breast cancer	11.01
16 Ischemic stroke	6.0	16 Pancreatic cancer	9.47
17 Protein-energy malnutrition	5.0	17 Stomach cancer	9.35
18 Self-harm by other specified means	5.0	18 Falls	8.97
19 Intracerebral hemorrhage	5.0	19 Neonatal preterm birth	6.95
20 Other congenital birth defects	5.0	20 Other non-Hodgkin lymphoma	6.88
71 Diabetes mellitus type 2		22 Exposure to forces of nature	
81 Other lower respiratory infections		23 Self-harm by other specified means	
84 Other non-Hodgkin lymphoma		28 Other congenital birth defects	
86 Falls		29 Protein-energy malnutrition	

Leading causes of death before and after garbage code redistribution: Denmark - 2015.

Causes are connected by arrows before and after redistribution. Infectious diseases are shown in red, non-communicable causes in blue, and injuries in green.
In addition to garbage redistribution, the diagram also reflects the deaths moved during misassignment correction for Alzheimer’s disease and other dementias.

Leading causes Before Redistribution	Deaths
1 Ischemic heart disease	3779.0
2 Tracheal, bronchus, and lung cancer	3702.0
3 Alzheimer's disease and other dementias	3664.0
4 Chronic obstructive pulmonary disease	3425.0
5 Colon and rectum cancer	1838.0
6 Prostate cancer	1170.0
7 Breast cancer	1070.0
8 Pancreatic cancer	964.0
9 Atrial fibrillation and flutter	749.0
10 Alcohol use disorders	710.0
11 Intracerebral hemorrhage	681.0
12 Cirrhosis and other chronic liver diseases	591.0
13 Self-harm by other specified means	521.0
14 Ischemic stroke	513.0
15 Bladder cancer	502.0
16 Other lower respiratory infections	478.0
17 Aortic aneurysm	471.0
18 Falls	463.0
19 Brain and central nervous system cancer	459.0
20 Non-rheumatic calcific aortic valve disease	441.0
26 Chronic kidney disease due to hypertension	
27 Diabetes mellitus type 2	

Leading causes After Redistribution	Deaths
1 Ischemic heart disease	5502.17
2 Tracheal, bronchus, and lung cancer	4345.26
3 Chronic obstructive pulmonary disease	4162.27
4 Alzheimer's disease and other dementias	3271.52
5 Ischemic stroke	2797.77
6 Colon and rectum cancer	2425.95
7 Other lower respiratory infections	1964.84
8 Prostate cancer	1451.68
9 Breast cancer	1326.67
10 Diabetes mellitus type 2	1308.81
11 Intracerebral hemorrhage	1212.4
12 Pancreatic cancer	1176.39
13 Falls	799.52
14 Alcohol use disorders	786.0
15 Atrial fibrillation and flutter	768.23
16 Cirrhosis and other chronic liver diseases	761.91
17 Non-rheumatic calcific aortic valve disease	651.57
18 Chronic kidney disease due to hypertension	627.14
19 Bladder cancer	623.45
20 Self-harm by other specified means	595.87
21 Aortic aneurysm	
23 Brain and central nervous system cancer	

Leading causes of death before and after garbage code redistribution: Dominican Republic - 2002.

Causes are connected by arrows before and after redistribution. Infectious diseases are shown in red, non-communicable causes in blue, and injuries in green.
In addition to garbage redistribution, the diagram also reflects the deaths moved during misassignment correction for Alzheimer's disease and other dementias.

Leading causes Before Redistribution	Deaths
1 Ischemic heart disease	2916.0
2 Motor vehicle road injuries	1258.0
3 Neonatal preterm birth	1001.0
4 HIV/AIDS resulting in other diseases	904.0
5 Cirrhosis and other chronic liver diseases	838.0
6 Ischemic stroke	648.0
7 Prostate cancer	607.0
8 Intracerebral hemorrhage	573.0
9 Tracheal, bronchus, and lung cancer	428.0
10 Hypertensive heart disease	404.0
11 Drug-susceptible tuberculosis	361.0
12 Neonatal sepsis and other neonatal infections	340.0
13 Breast cancer	264.0
14 Chronic obstructive pulmonary disease	242.0
15 Asthma	239.0
16 Neonatal encephalopathy due to birth asphyxia and trauma	230.0
17 Stomach cancer	227.0
18 Diarrheal diseases	197.28
19 Diabetes mellitus type 2	194.0
20 Colon and rectum cancer	174.0
21 Physical violence by firearm	
30 Self-harm by other specified means	
39 Alzheimer's disease and other dementias	
62 Other lower respiratory infections	

Leading causes After Redistribution	Deaths
1 Ischemic heart disease	4002.52
2 Motor vehicle road injuries	1398.32
3 Ischemic stroke	1363.29
4 Intracerebral hemorrhage	1293.71
5 Cirrhosis and other chronic liver diseases	1031.36
6 Neonatal preterm birth	1030.38
7 HIV/AIDS resulting in other diseases	1020.91
8 Diabetes mellitus type 2	941.68
9 Prostate cancer	784.09
10 Other lower respiratory infections	695.01
11 Hypertensive heart disease	651.95
12 Tracheal, bronchus, and lung cancer	538.41
13 Alzheimer's disease and other dementias	525.78
14 Self-harm by other specified means	450.4
15 Physical violence by firearm	413.57
16 Drug-susceptible tuberculosis	396.39
17 Chronic obstructive pulmonary disease	373.99
18 Breast cancer	365.51
19 Neonatal sepsis and other neonatal infections	357.81
20 Stomach cancer	341.12
21 Colon and rectum cancer	
22 Asthma	
24 Diarrheal diseases	
25 Neonatal encephalopathy due to birth asphyxia and trauma	

Leading causes of death before and after garbage code redistribution: Ecuador - 2015.

Causes are connected by arrows before and after redistribution. Infectious diseases are shown in red, non-communicable causes in blue, and injuries in green.
In addition to garbage redistribution, the diagram also reflects the deaths moved during misassignment correction for Alzheimer’s disease and other dementias.

Leading causes Before Redistribution	Deaths	Leading causes After Redistribution	Deaths
1 Ischemic heart disease	5061.0	1 Ischemic heart disease	6620.79
2 Cirrhosis and other chronic liver diseases	2114.0	2 Diabetes mellitus type 2	3324.09
3 Chronic obstructive pulmonary disease	1566.0	3 Other lower respiratory infections	2560.06
4 Stomach cancer	1533.0	4 Cirrhosis and other chronic liver diseases	2517.2
5 Hypertensive heart disease	1528.0	5 Ischemic stroke	2380.53
6 Diabetes mellitus type 2	1360.0	6 Stomach cancer	2167.09
7 Chronic kidney disease	1134.0	7 Hypertensive heart disease	2007.75
8 Self-harm by other specified means	1002.0	8 Chronic obstructive pulmonary disease	2002.63
9 Prostate cancer	948.0	9 Chronic kidney disease due to diabetes mellitus type 2	1805.58
10 Chronic kidney disease due to hypertension	934.0	10 Intracerebral hemorrhage	1676.21
11 Intracerebral hemorrhage	904.0	11 Chronic kidney disease due to hypertension	1525.1
12 Chronic kidney disease due to diabetes mellitus type 2	837.0	12 Chronic kidney disease	1399.72
13 Neonatal preterm birth	779.0	13 Pedestrian road injuries	1314.87
14 Ischemic stroke	771.0	14 Alzheimer's disease and other dementias	1210.81
15 Colon and rectum cancer	765.0	15 Self-harm by other specified means	1176.88
16 Tracheal, bronchus, and lung cancer	742.0	16 Prostate cancer	1173.95
17 HIV/AIDS resulting in other diseases	658.0	17 Motor vehicle road injuries	1117.79
18 Interstitial lung disease and pulmonary sarcoidosis	619.0	18 Colon and rectum cancer	1067.14
19 Pedestrian road injuries	611.0	19 Pneumococcal pneumonia	947.51
20 Motorcyclist road injuries	586.0	20 Tracheal, bronchus, and lung cancer	906.54
27 Alzheimer's disease and other dementias		22 Neonatal preterm birth	
43 Motor vehicle road injuries		24 Interstitial lung disease and pulmonary sarcoidosis	
76 Other lower respiratory infections		25 Motorcyclist road injuries	
150 Pneumococcal pneumonia		27 HIV/AIDS resulting in other diseases	

Leading causes of death before and after garbage code redistribution: Egypt - 2015.

Causes are connected by arrows before and after redistribution. Infectious diseases are shown in red, non-communicable causes in blue, and injuries in green.
In addition to garbage redistribution, the diagram also reflects the deaths moved during misassignment correction for Alzheimer's disease and other dementias.

Leading causes Before Redistribution	Deaths
1 Cirrhosis and other chronic liver diseases	32458.0
2 Ischemic heart disease	30364.0
3 Intracerebral hemorrhage	20705.0
4 Ischemic stroke	8586.0
5 Liver cancer	6785.0
6 Chronic kidney disease	6625.0
7 Congenital heart anomalies	5990.0
8 Other lower respiratory infections	5780.0
9 Diarrheal diseases	4255.0
10 Motor vehicle road injuries	3779.0
11 Neonatal preterm birth	3654.0
12 Tracheal, bronchus, and lung cancer	3495.0
13 Breast cancer	3110.0
14 Myocarditis	3104.0
15 Brain and central nervous system cancer	3096.0
16 Leukemia	3006.0
17 Other cardiovascular and circulatory diseases (internal)	2473.0
18 Asthma	1954.0
19 Pedestrian road injuries	1898.0
20 Chronic obstructive pulmonary disease	1872.0
25 Pneumococcal pneumonia	
32 Hypertensive heart disease	
40 Alzheimer's disease and other dementias	
73 Chronic kidney disease due to hypertension	
79 Diabetes mellitus type 2	
181 Other cardiomyopathy	

Leading causes After Redistribution	Deaths
1 Ischemic heart disease	134800.49
2 Cirrhosis and other chronic liver diseases	65261.67
3 Ischemic stroke	37773.68
4 Intracerebral hemorrhage	36817.5
5 Diabetes mellitus type 2	29756.81
6 Other lower respiratory infections	21172.3
7 Hypertensive heart disease	20503.49
8 Chronic kidney disease	14891.03
9 Congenital heart anomalies	13681.8
10 Other cardiomyopathy	11432.17
11 Liver cancer	11321.84
12 Chronic obstructive pulmonary disease	9920.64
13 Chronic kidney disease due to hypertension	9223.55
14 Tracheal, bronchus, and lung cancer	7737.07
15 Alzheimer's disease and other dementias	7714.23
16 Neonatal preterm birth	7078.0
17 Motor vehicle road injuries	6801.92
18 Diarrheal diseases	6490.78
19 Other cardiovascular and circulatory diseases (internal)	6213.28
20 Pneumococcal pneumonia	6165.6
21 Breast cancer	
23 Leukemia	
24 Brain and central nervous system cancer	
26 Myocarditis	
27 Asthma	
28 Pedestrian road injuries	

Leading causes of death before and after garbage code redistribution: Spain - 2015.

Causes are connected by arrows before and after redistribution. Infectious diseases are shown in red, non-communicable causes in blue, and injuries in green.
In addition to garbage redistribution, the diagram also reflects the deaths moved during misassignment correction for Alzheimer's disease and other dementias.

Leading causes Before Redistribution	Deaths
1 Alzheimer's disease and other dementias	37034.0
2 Ischemic heart disease	33768.0
3 Tracheal, bronchus, and lung cancer	21739.0
4 Colon and rectum cancer	15530.0
5 Chronic obstructive pulmonary disease	14857.0
6 Hypertensive heart disease	6419.0
7 Pancreatic cancer	6412.0
8 Intracerebral hemorrhage	6321.0
9 Breast cancer	6320.0
10 Atrial fibrillation and flutter	6173.0
11 Prostate cancer	5829.0
12 Cirrhosis and other chronic liver diseases	5668.0
13 Stomach cancer	5556.0
14 Bladder cancer	5039.0
15 Urinary tract infections and interstitial nephritis	4779.0
16 Liver cancer	4408.0
17 Parkinson's disease	4070.0
18 Non-rheumatic calcific aortic valve disease	3663.0
19 Chronic kidney disease	3538.0
20 Ischemic stroke	3447.0
25 Chronic kidney disease due to hypertension	
40 Diabetes mellitus type 2	
101 Pneumococcal pneumonia	

Leading causes After Redistribution	Deaths
1 Ischemic heart disease	45895.5
2 Alzheimer's disease and other dementias	34893.76
3 Chronic obstructive pulmonary disease	31838.17
4 Tracheal, bronchus, and lung cancer	24646.27
5 Ischemic stroke	20760.2
6 Colon and rectum cancer	19013.67
7 Intracerebral hemorrhage	11305.26
8 Hypertensive heart disease	10211.63
9 Diabetes mellitus type 2	9410.43
10 Pneumococcal pneumonia	8632.9
11 Pancreatic cancer	7435.34
12 Breast cancer	7385.95
13 Stomach cancer	7323.15
14 Cirrhosis and other chronic liver diseases	7022.71
15 Prostate cancer	6948.64
16 Atrial fibrillation and flutter	6235.4
17 Parkinson's disease	6134.72
18 Bladder cancer	6051.59
19 Urinary tract infections and interstitial nephritis	5956.6
20 Chronic kidney disease due to hypertension	5727.68
21 Non-rheumatic calcific aortic valve disease	
23 Chronic kidney disease	
25 Liver cancer	

Leading causes of death before and after garbage code redistribution: Estonia - 2015.

Causes are connected by arrows before and after redistribution. Infectious diseases are shown in red, non-communicable causes in blue, and injuries in green.
In addition to garbage redistribution, the diagram also reflects the deaths moved during misassignment correction for Alzheimer's disease and other dementias.

Leading causes Before Redistribution	Deaths
1 Ischemic heart disease	3175.0
2 Hypertensive heart disease	2246.0
3 Tracheal, bronchus, and lung cancer	672.0
4 Ischemic stroke	516.0
5 Chronic kidney disease due to hypertension	484.0
6 Colon and rectum cancer	471.0
7 Cirrhosis and other chronic liver diseases	321.0
8 Prostate cancer	292.0
9 Stomach cancer	276.0
10 Breast cancer	243.0
11 Pancreatic cancer	238.0
12 Chronic obstructive pulmonary disease	193.0
13 Other lower respiratory infections	180.0
14 Self-harm by other specified means	178.0
15 Alcohol use disorders	175.0
16 Kidney cancer	164.0
17 Alzheimer's disease and other dementias	139.0
18 Intracerebral hemorrhage	136.0
19 Leukemia	130.0
20 Atrial fibrillation and flutter	125.0

Leading causes After Redistribution	Deaths
1 Ischemic heart disease	3410.2
2 Hypertensive heart disease	2419.55
3 Tracheal, bronchus, and lung cancer	729.83
4 Alzheimer's disease and other dementias	615.97
5 Ischemic stroke	611.61
6 Colon and rectum cancer	515.94
7 Chronic kidney disease due to hypertension	501.49
8 Cirrhosis and other chronic liver diseases	342.46
9 Prostate cancer	314.72
10 Stomach cancer	303.81
11 Breast cancer	266.43
12 Pancreatic cancer	257.14
13 Other lower respiratory infections	215.11
14 Chronic obstructive pulmonary disease	207.32
15 Self-harm by other specified means	199.47
16 Alcohol use disorders	186.12
17 Kidney cancer	174.99
18 Intracerebral hemorrhage	168.37
19 Atrial fibrillation and flutter	142.24
20 Leukemia	136.1

Leading causes of death before and after garbage code redistribution: Finland - 2015.

Causes are connected by arrows before and after redistribution. Infectious diseases are shown in red, non-communicable causes in blue, and injuries in green.

In addition to garbage redistribution, the diagram also reflects the deaths moved during misassignment correction for Alzheimer's disease and other dementias.

Leading causes Before Redistribution	Deaths	Leading causes After Redistribution	Deaths
1 Ischemic heart disease	10209.0	1 Ischemic heart disease	12346.65
2 Alzheimer's disease and other dementias	8786.0	2 Alzheimer's disease and other dementias	4160.23
3 Ischemic stroke	2911.0	3 Ischemic stroke	3361.32
4 Tracheal, bronchus, and lung cancer	2255.0	4 Tracheal, bronchus, and lung cancer	2540.49
5 Hypertensive heart disease	1357.0	5 Hypertensive heart disease	1695.31
6 Colon and rectum cancer	1222.0	6 Colon and rectum cancer	1522.59
7 Chronic obstructive pulmonary disease	1205.0	7 Chronic obstructive pulmonary disease	1377.13
8 Falls	1143.0	8 Pancreatic cancer	1368.4
9 Pancreatic cancer	1139.0	9 Falls	1261.46
10 Cirrhosis and other chronic liver diseases	1069.0	10 Cirrhosis and other chronic liver diseases	1170.29
11 Intracerebral hemorrhage	925.0	11 Prostate cancer	1095.74
12 Prostate cancer	899.0	12 Intracerebral hemorrhage	1049.15
13 Breast cancer	824.0	13 Breast cancer	995.42
14 Parkinson's disease	681.0	14 Atrial fibrillation and flutter	746.99
15 Self-harm by other specified means	608.0	15 Parkinson's disease	700.12
16 Alcohol use disorders	551.0	16 Self-harm by other specified means	683.2
17 Non-rheumatic calcific aortic valve disease	538.0	17 Non-rheumatic calcific aortic valve disease	662.13
18 Aortic aneurysm	457.0	18 Stomach cancer	618.67
19 Lower extremity peripheral arterial disease	455.0	19 Other non-Hodgkin lymphoma	583.42
20 Stomach cancer	442.0	20 Alcohol use disorders	575.13
21 Other non-Hodgkin lymphoma		21 Lower extremity peripheral arterial disease	
34 Atrial fibrillation and flutter		22 Aortic aneurysm	

Leading causes of death before and after garbage code redistribution: Fiji - 2008.

Causes are connected by arrows before and after redistribution. Infectious diseases are shown in red, non-communicable causes in blue, and injuries in green.
In addition to garbage redistribution, the diagram also reflects the deaths moved during misassignment correction for Alzheimer's disease and other dementias.

Leading causes Before Redistribution	Deaths
1 Diabetes mellitus type 2	746.0
2 Ischemic heart disease	745.0
3 Asthma	140.0
4 Breast cancer	105.0
5 Diarrheal diseases	87.0
6 Cervical cancer	80.0
7 Neonatal preterm birth	69.0
8 Chronic kidney disease	61.0
9 Drowning	59.0
10 Neonatal encephalopathy due to birth asphyxia and trauma	45.0
11 Ovarian cancer	44.0
12 Congenital heart anomalies	39.0
13 Stomach cancer	38.0
14 Self-harm by other specified means	36.0
15 Prostate cancer	34.0
16 Fire, heat, and hot substances	30.0
17 Rheumatic heart disease	30.0
18 Pulmonary aspiration and foreign body in airway	28.0
19 Liver cancer	27.0
20 Drug-susceptible tuberculosis	24.0
22 Intracerebral hemorrhage	
25 Chronic obstructive pulmonary disease	
57 Other lower respiratory infections	
71 Ischemic stroke	
105 Alzheimer's disease and other dementias	
109 Chronic kidney disease due to hypertension	
115 Hypertensive heart disease	

Leading causes After Redistribution	Deaths
1 Diabetes mellitus type 2	1486.86
2 Ischemic heart disease	1330.75
3 Intracerebral hemorrhage	350.25
4 Asthma	181.05
5 Diarrheal diseases	163.45
6 Other lower respiratory infections	134.01
7 Hypertensive heart disease	130.47
8 Breast cancer	128.68
9 Chronic obstructive pulmonary disease	123.43
10 Ischemic stroke	106.56
11 Chronic kidney disease	101.0
12 Cervical cancer	100.44
13 Self-harm by other specified means	99.48
14 Rheumatic heart disease	79.95
15 Stomach cancer	77.54
16 Neonatal preterm birth	72.87
17 Chronic kidney disease due to hypertension	67.18
18 Drowning	61.31
19 Alzheimer's disease and other dementias	56.86
20 Ovarian cancer	53.98
22 Congenital heart anomalies	
23 Prostate cancer	
24 Neonatal encephalopathy due to birth asphyxia and trauma	
29 Pulmonary aspiration and foreign body in airway	
31 Drug-susceptible tuberculosis	
32 Liver cancer	
36 Fire, heat, and hot substances	

Leading causes of death before and after garbage code redistribution: France - 2015.

Causes are connected by arrows before and after redistribution. Infectious diseases are shown in red, non-communicable causes in blue, and injuries in green.
In addition to garbage redistribution, the diagram also reflects the deaths moved during misassignment correction for Alzheimer’s disease and other dementias.

Leading causes Before Redistribution	Deaths
1 Alzheimer's disease and other dementias	42045.0
2 Ischemic heart disease	33422.0
3 Tracheal, bronchus, and lung cancer	32214.0
4 Colon and rectum cancer	17930.0
5 Breast cancer	12434.0
6 Pancreatic cancer	10760.0
7 Chronic obstructive pulmonary disease	9509.0
8 Prostate cancer	8751.0
9 Ischemic stroke	8074.0
10 Atrial fibrillation and flutter	8016.0
11 Cirrhosis and other chronic liver diseases	7759.0
12 Falls	7590.0
13 Self-harm by other specified means	7241.0
14 Liver cancer	6729.0
15 Intracerebral hemorrhage	6641.0
16 Parkinson's disease	6089.0
17 Bladder cancer	5557.0
18 Other cardiovascular and circulatory diseases (internal)	4786.0
19 Non-rheumatic calcific aortic valve disease	4756.0
20 Hypertensive heart disease	4578.0
25 Diabetes mellitus type 2	
43 Influenza	

Leading causes After Redistribution	Deaths
1 Ischemic heart disease	56841.71
2 Alzheimer's disease and other dementias	43216.43
3 Tracheal, bronchus, and lung cancer	41351.15
4 Ischemic stroke	28096.33
5 Colon and rectum cancer	24568.04
6 Falls	18247.2
7 Breast cancer	15722.89
8 Chronic obstructive pulmonary disease	15706.37
9 Pancreatic cancer	13781.56
10 Diabetes mellitus type 2	12468.18
11 Intracerebral hemorrhage	11744.09
12 Prostate cancer	11700.5
13 Influenza	10903.62
14 Cirrhosis and other chronic liver diseases	10051.38
15 Hypertensive heart disease	8962.05
16 Atrial fibrillation and flutter	8930.62
17 Self-harm by other specified means	8815.07
18 Non-rheumatic calcific aortic valve disease	8614.11
19 Other cardiovascular and circulatory diseases (internal)	8208.4
20 Liver cancer	8160.85
21 Parkinson's disease	
22 Bladder cancer	

Leading causes of death before and after garbage code redistribution: United Kingdom - 2015.

Causes are connected by arrows before and after redistribution. Infectious diseases are shown in red, non-communicable causes in blue, and injuries in green.
In addition to garbage redistribution, the diagram also reflects the deaths moved during misassignment correction for Alzheimer’s disease and other dementias.

Leading causes Before Redistribution	Deaths
1 Alzheimer's disease and other dementias	70953.44
2 Ischemic heart disease	69540.38
3 Tracheal, bronchus, and lung cancer	35573.4
4 Chronic obstructive pulmonary disease	33074.88
5 Colon and rectum cancer	16633.21
6 Prostate cancer	11825.0
7 Breast cancer	11534.62
8 Pancreatic cancer	8924.0
9 Esophageal cancer	7950.77
10 Intracerebral hemorrhage	7351.0
11 Atrial fibrillation and flutter	6686.7
12 Interstitial lung disease and pulmonary sarcoidosis	6601.0
13 Falls	6360.18
14 Parkinson's disease	6117.0
15 Aortic aneurysm	6071.46
16 Cirrhosis and other chronic liver diseases	5966.67
17 Urinary tract infections and interstitial nephritis	5617.0
18 Bladder cancer	5447.88
19 Liver cancer	4812.0
20 Self-harm by other specified means	4621.6
24 Ischemic stroke	
86 Influenza	
93 Other lower respiratory infections	
116 Pneumococcal pneumonia	

Leading causes After Redistribution	Deaths
1 Ischemic heart disease	83188.7
2 Chronic obstructive pulmonary disease	41073.78
3 Tracheal, bronchus, and lung cancer	40677.72
4 Alzheimer's disease and other dementias	37794.03
5 Ischemic stroke	29619.77
6 Colon and rectum cancer	21531.66
7 Influenza	15598.56
8 Prostate cancer	14531.37
9 Breast cancer	14478.96
10 Other lower respiratory infections	12693.75
11 Intracerebral hemorrhage	12348.01
12 Pneumococcal pneumonia	12163.29
13 Pancreatic cancer	11240.23
14 Esophageal cancer	9650.16
15 Falls	9375.02
16 Cirrhosis and other chronic liver diseases	9354.26
17 Atrial fibrillation and flutter	8671.14
18 Parkinson's disease	7699.46
19 Interstitial lung disease and pulmonary sarcoidosis	7535.54
20 Urinary tract infections and interstitial nephritis	7362.7
22 Aortic aneurysm	
23 Bladder cancer	
24 Liver cancer	
25 Self-harm by other specified means	

Leading causes of death before and after garbage code redistribution: Georgia - 2015.

Causes are connected by arrows before and after redistribution. Infectious diseases are shown in red, non-communicable causes in blue, and injuries in green.
In addition to garbage redistribution, the diagram also reflects the deaths moved during misassignment correction for Alzheimer's disease and other dementias.

Leading causes Before Redistribution	Deaths
1 Ischemic heart disease	4880.0
2 Hypertensive heart disease	2528.0
3 Ischemic stroke	1745.0
4 Intracerebral hemorrhage	1450.0
5 Tracheal, bronchus, and lung cancer	1026.0
6 Cirrhosis and other chronic liver diseases	704.0
7 Breast cancer	611.0
8 Diabetes mellitus type 2	562.0
9 Subarachnoid hemorrhage	492.0
10 Stomach cancer	483.0
11 Colon and rectum cancer	427.0
12 Motor vehicle road injuries	339.0
13 Atrial fibrillation and flutter	335.0
14 Rheumatic heart disease	290.0
15 Prostate cancer	282.0
16 Brain and central nervous system cancer	244.0
17 Chronic obstructive pulmonary disease	243.0
18 Pedestrian road injuries	198.0
19 Pancreatic cancer	184.0
20 Bladder cancer	184.0
23 Falls	
42 Alzheimer's disease and other dementias	
83 Other lower respiratory infections	

Leading causes After Redistribution	Deaths
1 Ischemic heart disease	9941.1
2 Ischemic stroke	5602.01
3 Hypertensive heart disease	5056.41
4 Intracerebral hemorrhage	3764.37
5 Tracheal, bronchus, and lung cancer	1810.65
6 Diabetes mellitus type 2	1424.64
7 Cirrhosis and other chronic liver diseases	1424.29
8 Alzheimer's disease and other dementias	1286.05
9 Breast cancer	998.18
10 Stomach cancer	976.16
11 Other lower respiratory infections	921.34
12 Colon and rectum cancer	851.08
13 Subarachnoid hemorrhage	839.85
14 Chronic obstructive pulmonary disease	735.8
15 Rheumatic heart disease	615.12
16 Prostate cancer	541.5
17 Motor vehicle road injuries	525.48
18 Brain and central nervous system cancer	375.29
19 Pancreatic cancer	348.23
20 Falls	346.76
22 Atrial fibrillation and flutter	
23 Pedestrian road injuries	
24 Bladder cancer	

Leading causes of death before and after garbage code redistribution: Greece - 2015.

Causes are connected by arrows before and after redistribution. Infectious diseases are shown in red, non-communicable causes in blue, and injuries in green.
In addition to garbage redistribution, the diagram also reflects the deaths moved during misassignment correction for Alzheimer's disease and other dementias.

Leading causes Before Redistribution	Deaths
1 Ischemic heart disease	12296.0
2 Tracheal, bronchus, and lung cancer	7156.0
3 Chronic obstructive pulmonary disease	3448.0
4 Colon and rectum cancer	2805.0
5 Breast cancer	2138.0
6 Pancreatic cancer	1859.0
7 Intracerebral hemorrhage	1802.0
8 Prostate cancer	1763.0
9 Alzheimer's disease and other dementias	1757.0
10 Chronic kidney disease	1690.0
11 Chronic kidney disease due to hypertension	1578.0
12 Stomach cancer	1357.0
13 Hypertensive heart disease	1328.0
14 Bladder cancer	1306.0
15 Parkinson's disease	1158.0
16 Brain and central nervous system cancer	1128.0
17 Urinary tract infections and interstitial nephritis	961.0
18 Cirrhosis and other chronic liver diseases	896.0
19 Aortic aneurysm	882.0
20 Kidney cancer	629.0
41 Ischemic stroke	
91 Influenza	
95 Diabetes mellitus type 2	
112 Other lower respiratory infections	

Leading causes After Redistribution	Deaths
1 Ischemic heart disease	19354.52
2 Ischemic stroke	11047.35
3 Tracheal, bronchus, and lung cancer	8443.29
4 Alzheimer's disease and other dementias	8152.26
5 Intracerebral hemorrhage	5394.25
6 Chronic obstructive pulmonary disease	5007.25
7 Colon and rectum cancer	3882.07
8 Other lower respiratory infections	3484.8
9 Hypertensive heart disease	2915.73
10 Chronic kidney disease due to hypertension	2766.64
11 Breast cancer	2730.97
12 Pancreatic cancer	2401.38
13 Prostate cancer	2374.08
14 Chronic kidney disease	2318.19
15 Influenza	2191.45
16 Stomach cancer	1985.24
17 Bladder cancer	1668.96
18 Parkinson's disease	1531.0
19 Diabetes mellitus type 2	1420.32
20 Brain and central nervous system cancer	1377.16
23 Cirrhosis and other chronic liver diseases	
25 Urinary tract infections and interstitial nephritis	
26 Aortic aneurysm	
29 Kidney cancer	

Leading causes of death before and after garbage code redistribution: Grenada - 2015.

Causes are connected by arrows before and after redistribution. Infectious diseases are shown in red, non-communicable causes in blue, and injuries in green.
In addition to garbage redistribution, the diagram also reflects the deaths moved during misassignment correction for Alzheimer's disease and other dementias.

Leading causes Before Redistribution	Deaths
1 Ischemic heart disease	66.0
2 Prostate cancer	33.0
3 Diabetes mellitus type 2	20.0
4 Hypertensive heart disease	19.0
5 Breast cancer	18.0
6 Colon and rectum cancer	16.0
7 Ischemic stroke	12.0
8 Decubitus ulcer	12.0
9 Cirrhosis and other chronic liver diseases	11.0
10 Intracerebral hemorrhage	11.0
11 Falls	10.0
12 Aortic aneurysm	10.0
13 Chronic kidney disease due to hypertension	9.0
14 Chronic kidney disease due to glomerulonephritis	9.0
15 Cervical cancer	8.0
16 Tracheal, bronchus, and lung cancer	8.0
17 Neonatal encephalopathy due to birth asphyxia and trauma	8.0
18 Pulmonary aspiration and foreign body in airway	8.0
19 Chronic kidney disease due to diabetes mellitus type 2	6.0
20 Urinary tract infections and interstitial nephritis	6.0
66 Alzheimer's disease and other dementias	
67 Influenza	
89 Other lower respiratory infections	

Leading causes After Redistribution	Deaths
1 Ischemic heart disease	92.51
2 Diabetes mellitus type 2	81.02
3 Ischemic stroke	60.62
4 Prostate cancer	37.61
5 Other lower respiratory infections	35.46
6 Intracerebral hemorrhage	33.74
7 Hypertensive heart disease	29.09
8 Breast cancer	22.08
9 Colon and rectum cancer	20.74
10 Influenza	19.72
11 Chronic kidney disease due to hypertension	19.29
12 Chronic kidney disease due to diabetes mellitus type 2	16.65
13 Falls	16.29
14 Alzheimer's disease and other dementias	15.47
15 Cirrhosis and other chronic liver diseases	13.23
16 Chronic kidney disease due to glomerulonephritis	13.05
17 Decubitus ulcer	12.59
18 Aortic aneurysm	11.41
19 Cervical cancer	11.13
20 Tracheal, bronchus, and lung cancer	10.99
21 Neonatal encephalopathy due to birth asphyxia and trauma	
23 Pulmonary aspiration and foreign body in airway	
30 Urinary tract infections and interstitial nephritis	

Leading causes of death before and after garbage code redistribution: Greenland - 2015.

Causes are connected by arrows before and after redistribution. Infectious diseases are shown in red, non-communicable causes in blue, and injuries in green.
In addition to garbage redistribution, the diagram also reflects the deaths moved during misassignment correction for Alzheimer’s disease and other dementias.

Leading causes Before Redistribution	Deaths
1 Tracheal, bronchus, and lung cancer	34.0
2 Chronic obstructive pulmonary disease	27.0
3 Self-harm by other specified means	26.0
4 Ischemic heart disease	20.0
5 Colon and rectum cancer	16.0
6 Alzheimer's disease and other dementias	10.0
7 Pancreatic cancer	8.0
8 Intracerebral hemorrhage	8.0
9 Drowning	7.0
10 Peptic ulcer disease	6.0
11 Self-harm by firearm	6.0
12 Falls	6.0
13 Diabetes mellitus type 2	6.0
14 Fire, heat, and hot substances	4.0
15 Stomach cancer	4.0
16 Ovarian cancer	4.0
17 Alcohol use disorders	4.0
18 Parkinson's disease	4.0
19 Vascular intestinal disorders	3.0
20 Cervical cancer	3.0
21 Breast cancer	
26 Neonatal preterm birth	
48 Ischemic stroke	

Leading causes After Redistribution	Deaths
1 Tracheal, bronchus, and lung cancer	49.87
2 Ischemic heart disease	45.49
3 Chronic obstructive pulmonary disease	43.62
4 Self-harm by other specified means	31.78
5 Colon and rectum cancer	23.86
6 Intracerebral hemorrhage	21.38
7 Ischemic stroke	14.32
8 Pancreatic cancer	11.97
9 Falls	11.76
10 Diabetes mellitus type 2	11.34
11 Peptic ulcer disease	9.62
12 Alzheimer's disease and other dementias	9.38
13 Drowning	8.13
14 Self-harm by firearm	7.93
15 Ovarian cancer	6.71
16 Stomach cancer	6.16
17 Breast cancer	5.77
18 Parkinson's disease	5.76
19 Neonatal preterm birth	5.71
20 Fire, heat, and hot substances	4.99
21 Alcohol use disorders	
25 Vascular intestinal disorders	
34 Cervical cancer	

Leading causes of death before and after garbage code redistribution: Guatemala - 2015.

Causes are connected by arrows before and after redistribution. Infectious diseases are shown in red, non-communicable causes in blue, and injuries in green.
In addition to garbage redistribution, the diagram also reflects the deaths moved during misassignment correction for Alzheimer's disease and other dementias.

**Leading causes Before Redistribution**	**Deaths**
1 Ischemic heart disease	5740.0
2 Cirrhosis and other chronic liver diseases	3442.0
3 Physical violence by firearm	3273.0
4 Diarrheal diseases	2143.0
5 Stomach cancer	1375.0
6 Chronic kidney disease	1367.0
7 Neonatal preterm birth	1310.0
8 Diabetes mellitus type 2	1269.0
9 Alcohol use disorders	1132.0
10 Protein-energy malnutrition	1110.0
11 Chronic obstructive pulmonary disease	987.0
12 Unintentional firearm injuries	765.0
13 Prostate cancer	618.0
14 Peptic ulcer disease	590.0
15 Neonatal encephalopathy due to birth asphyxia and trauma	561.0
16 Congenital heart anomalies	529.0
17 Intracerebral hemorrhage	498.0
18 Self-harm by other specified means	453.0
19 Other neonatal disorders	407.0
20 Neonatal sepsis and other neonatal infections	407.0
28 Chronic kidney disease due to diabetes mellitus type 2	
50 Alzheimer's disease and other dementias	
68 Ischemic stroke	
103 Other lower respiratory infections	
144 Falls	
156 Pedestrian road injuries	
181 Pneumococcal pneumonia	

**Leading causes After Redistribution**	**Deaths**
1 Ischemic heart disease	8014.3
2 Other lower respiratory infections	6809.56
3 Diabetes mellitus type 2	5661.58
4 Cirrhosis and other chronic liver diseases	4400.44
5 Physical violence by firearm	3749.53
6 Diarrheal diseases	2791.52
7 Stomach cancer	2653.22
8 Intracerebral hemorrhage	2144.15
9 Chronic kidney disease	1988.42
10 Ischemic stroke	1629.43
11 Neonatal preterm birth	1519.09
12 Chronic obstructive pulmonary disease	1517.97
13 Chronic kidney disease due to diabetes mellitus type 2	1412.51
14 Protein-energy malnutrition	1375.57
15 Alcohol use disorders	1299.58
16 Pedestrian road injuries	1219.48
17 Alzheimer's disease and other dementias	1157.47
18 Falls	1089.89
19 Pneumococcal pneumonia	1051.31
20 Prostate cancer	965.73
22 Unintentional firearm injuries	
23 Peptic ulcer disease	
27 Congenital heart anomalies	
28 Self-harm by other specified means	
29 Neonatal encephalopathy due to birth asphyxia and trauma	
35 Neonatal sepsis and other neonatal infections	
37 Other neonatal disorders	

Leading causes of death before and after garbage code redistribution: Guam - 2015.

Causes are connected by arrows before and after redistribution. Infectious diseases are shown in red, non-communicable causes in blue, and injuries in green.
In addition to garbage redistribution, the diagram also reflects the deaths moved during misassignment correction for Alzheimer's disease and other dementias.

Leading causes Before Redistribution	Deaths
1 Ischemic heart disease	246.0
2 Tracheal, bronchus, and lung cancer	51.0
3 Chronic obstructive pulmonary disease	30.0
4 Self-harm by other specified means	29.0
5 Cirrhosis and other chronic liver diseases	25.0
6 Intracerebral hemorrhage	23.0
7 Hypertensive heart disease	22.0
8 Ischemic stroke	19.0
9 Alzheimer's disease and other dementias	18.0
10 Breast cancer	17.0
11 Neonatal preterm birth	15.0
12 Colon and rectum cancer	15.0
13 Prostate cancer	12.0
14 Chronic kidney disease	12.0
15 Chronic kidney disease due to hypertension	11.0
16 Pancreatic cancer	11.0
17 Liver cancer	10.0
18 Falls	10.0
19 Drowning	8.0
20 Esophageal cancer	7.0
23 Stomach cancer	
68 Chronic kidney disease due to diabetes mellitus type 2	
77 Diabetes mellitus type 2	
78 Other lower respiratory infections	

Leading causes After Redistribution	Deaths
1 Ischemic heart disease	260.15
2 Tracheal, bronchus, and lung cancer	53.83
3 Intracerebral hemorrhage	41.69
4 Chronic obstructive pulmonary disease	39.78
5 Diabetes mellitus type 2	37.08
6 Ischemic stroke	30.36
7 Self-harm by other specified means	29.43
8 Cirrhosis and other chronic liver diseases	28.63
9 Hypertensive heart disease	24.6
10 Alzheimer's disease and other dementias	20.27
11 Other lower respiratory infections	19.3
12 Breast cancer	19.09
13 Colon and rectum cancer	18.27
14 Neonatal preterm birth	17.87
15 Chronic kidney disease due to diabetes mellitus type 2	16.5
16 Prostate cancer	13.43
17 Chronic kidney disease	12.65
18 Chronic kidney disease due to hypertension	12.54
19 Pancreatic cancer	12.07
20 Stomach cancer	11.58
21 Falls	
22 Liver cancer	
23 Drowning	
25 Esophageal cancer	

Leading causes of death before and after garbage code redistribution: Guyana - 2014.

Causes are connected by arrows before and after redistribution. Infectious diseases are shown in red, non-communicable causes in blue, and injuries in green.
In addition to garbage redistribution, the diagram also reflects the deaths moved during misassignment correction for Alzheimer’s disease and other dementias.

Leading causes Before Redistribution	Deaths
1 Ischemic heart disease	564.0
2 Self-harm by other specified means	203.0
3 Hypertensive heart disease	161.0
4 HIV/AIDS resulting in other diseases	139.0
5 Cirrhosis and other chronic liver diseases	135.0
6 Drowning	83.0
7 Neonatal preterm birth	81.0
8 Drug-susceptible tuberculosis	76.0
9 Intracerebral hemorrhage	76.0
10 Prostate cancer	74.0
11 Chronic kidney disease due to hypertension	66.0
12 Diabetes mellitus type 2	61.0
13 Breast cancer	61.0
14 Peptic ulcer disease	54.0
15 Chronic obstructive pulmonary disease	53.0
16 HIV/AIDS - Drug-susceptible Tuberculosis	48.0
17 Physical violence by firearm	46.0
18 Aortic aneurysm	43.0
19 Physical violence by sharp object	42.0
20 Chronic kidney disease	41.0
21 Colon and rectum cancer	
28 Ischemic stroke	
52 Chronic kidney disease due to diabetes mellitus type 2	
144 Other lower respiratory infections	

Leading causes After Redistribution	Deaths
1 Ischemic heart disease	746.31
2 Diabetes mellitus type 2	391.6
3 Ischemic stroke	314.02
4 Intracerebral hemorrhage	311.41
5 Hypertensive heart disease	250.67
6 Self-harm by other specified means	240.94
7 Cirrhosis and other chronic liver diseases	197.4
8 Other lower respiratory infections	194.29
9 HIV/AIDS resulting in other diseases	149.43
10 Chronic kidney disease due to hypertension	115.06
11 Drowning	96.52
12 Chronic kidney disease due to diabetes mellitus type 2	95.21
13 Prostate cancer	90.84
14 Neonatal preterm birth	84.17
15 Drug-susceptible tuberculosis	81.47
16 Breast cancer	80.0
17 Chronic obstructive pulmonary disease	68.0
18 Colon and rectum cancer	62.39
19 Physical violence by firearm	60.82
20 Peptic ulcer disease	56.63
21 Physical violence by sharp object	
23 Chronic kidney disease	
25 HIV/AIDS - Drug-susceptible Tuberculosis	
29 Aortic aneurysm	

Leading causes of death before and after garbage code redistribution: Honduras - 2013.

Causes are connected by arrows before and after redistribution. Infectious diseases are shown in red, non-communicable causes in blue, and injuries in green.
In addition to garbage redistribution, the diagram also reflects the deaths moved during misassignment correction for Alzheimer's disease and other dementias.

Leading causes Before Redistribution	Deaths	Leading causes After Redistribution	Deaths
1 Neonatal preterm birth	523.0	1 Neonatal preterm birth	535.91
2 Diabetes mellitus type 2	363.0	2 Diabetes mellitus type 2	415.66
3 Ischemic heart disease	285.0	3 Other lower respiratory infections	353.84
4 Neonatal sepsis and other neonatal infections	246.0	4 Ischemic heart disease	338.17
5 Chronic obstructive pulmonary disease	214.0	5 Cirrhosis and other chronic liver diseases	264.65
6 Cirrhosis and other chronic liver diseases	213.0	6 Neonatal sepsis and other neonatal infections	255.28
7 Neonatal encephalopathy due to birth asphyxia and trauma	191.0	7 Chronic obstructive pulmonary disease	235.86
8 Congenital heart anomalies	157.0	8 Neonatal encephalopathy due to birth asphyxia and trauma	194.37
9 Other congenital birth defects	127.0	9 Intracerebral hemorrhage	185.62
10 HIV/AIDS resulting in other diseases	127.0	10 Congenital heart anomalies	167.4
11 Physical violence by firearm	110.0	11 Ischemic stroke	167.03
12 Intracerebral hemorrhage	102.0	12 HIV/AIDS resulting in other diseases	133.99
13 Self-harm by other specified means	93.0	13 Other congenital birth defects	131.69
14 Drug-susceptible tuberculosis	91.0	14 Physical violence by firearm	129.18
15 Hypertensive heart disease	83.0	15 Chronic kidney disease due to diabetes mellitus type 2	109.15
16 Chronic kidney disease due to diabetes mellitus type 2	83.0	16 Self-harm by other specified means	101.46
17 Chronic kidney disease	73.0	17 Chronic kidney disease due to hypertension	94.68
18 Chronic kidney disease due to hypertension	69.0	18 Drug-susceptible tuberculosis	93.37
19 Diarrheal diseases	67.0	19 Hypertensive heart disease	91.96
20 Pedestrian road injuries	60.0	20 Pedestrian road injuries	86.44
49 Ischemic stroke		21 Chronic kidney disease	
59 Other lower respiratory infections		24 Diarrheal diseases	

Leading causes of death before and after garbage code redistribution: Croatia - 2015.

Causes are connected by arrows before and after redistribution. Infectious diseases are shown in red, non-communicable causes in blue, and injuries in green.
In addition to garbage redistribution, the diagram also reflects the deaths moved during misassignment correction for Alzheimer’s disease and other dementias.

Leading causes Before Redistribution	Deaths	Leading causes After Redistribution	Deaths
1 Ischemic heart disease	11509.0	1 Ischemic heart disease	13051.48
2 Tracheal, bronchus, and lung cancer	2826.0	2 Ischemic stroke	5823.23
3 Colon and rectum cancer	2056.0	3 Tracheal, bronchus, and lung cancer	3008.96
4 Chronic obstructive pulmonary disease	1872.0	4 Colon and rectum cancer	2336.42
5 Hypertensive heart disease	1696.0	5 Hypertensive heart disease	2015.04
6 Ischemic stroke	1172.0	6 Chronic obstructive pulmonary disease	1978.27
7 Falls	1160.0	7 Alzheimer's disease and other dementias	1620.21
8 Breast cancer	1054.0	8 Intracerebral hemorrhage	1606.45
9 Cirrhosis and other chronic liver diseases	1003.0	9 Falls	1293.54
10 Alzheimer's disease and other dementias	935.0	10 Diabetes mellitus type 2	1151.75
11 Prostate cancer	832.0	11 Breast cancer	1135.28
12 Stomach cancer	801.0	12 Cirrhosis and other chronic liver diseases	1094.77
13 Pancreatic cancer	763.0	13 Prostate cancer	918.71
14 Self-harm by other specified means	648.0	14 Stomach cancer	911.12
15 Intracerebral hemorrhage	551.0	15 Pancreatic cancer	841.61
16 Urinary tract infections and interstitial nephritis	504.0	16 Brain and central nervous system cancer	738.56
17 Bladder cancer	475.0	17 Self-harm by other specified means	674.48
18 Brain and central nervous system cancer	438.0	18 Bladder cancer	520.95
19 Chronic kidney disease	400.0	19 Parkinson's disease	510.34
20 Diabetes mellitus type 2	392.0	20 Urinary tract infections and interstitial nephritis	506.31
22 Parkinson's disease		22 Chronic kidney disease	

Leading causes of death before and after garbage code redistribution: Haiti - 2004.

Causes are connected by arrows before and after redistribution. Infectious diseases are shown in red, non-communicable causes in blue, and injuries in green.
In addition to garbage redistribution, the diagram also reflects the deaths moved during misassignment correction for Alzheimer's disease and other dementias.

Leading causes Before Redistribution	Deaths
1 HIV/AIDS resulting in other diseases	67.0
2 Diarrheal diseases	57.0
3 Protein-energy malnutrition	45.0
4 Physical violence by firearm	42.0
5 Drug-susceptible tuberculosis	39.0
6 Intracerebral hemorrhage	37.0
7 Ischemic stroke	30.0
8 Hypertensive heart disease	27.0
9 Typhoid fever	26.0
10 Ischemic heart disease	25.0
11 Neonatal sepsis and other neonatal infections	23.0
12 Cirrhosis and other chronic liver diseases	21.0
13 Neonatal preterm birth	20.0
14 Fire, heat, and hot substances	16.0
15 Neonatal encephalopathy due to birth asphyxia and trauma	15.0
16 Malaria	14.0
17 Maternal hypertensive disorders	13.0
18 HIV/AIDS - Drug-susceptible Tuberculosis	13.0
19 Prostate cancer	13.0
20 Tetanus	13.0
21 Colon and rectum cancer	
23 Stomach cancer	
32 Diabetes mellitus type 2	
45 Chronic kidney disease due to hypertension	
100 Other lower respiratory infections	

Leading causes After Redistribution	Deaths
1 Ischemic heart disease	202.82
2 HIV/AIDS resulting in other diseases	150.86
3 Intracerebral hemorrhage	147.85
4 Diabetes mellitus type 2	128.78
5 Hypertensive heart disease	102.03
6 Diarrheal diseases	97.48
7 Ischemic stroke	94.3
8 Physical violence by firearm	88.05
9 Other lower respiratory infections	66.9
10 Drug-susceptible tuberculosis	63.31
11 Protein-energy malnutrition	62.52
12 Cirrhosis and other chronic liver diseases	48.01
13 Typhoid fever	40.21
14 Colon and rectum cancer	32.03
15 Neonatal sepsis and other neonatal infections	31.44
16 Chronic kidney disease due to hypertension	28.65
17 Prostate cancer	28.54
18 Neonatal preterm birth	28.25
19 Fire, heat, and hot substances	27.09
20 Stomach cancer	25.52
21 HIV/AIDS - Drug-susceptible Tuberculosis	
24 Maternal hypertensive disorders	
27 Malaria	
28 Tetanus	
29 Neonatal encephalopathy due to birth asphyxia and trauma	

Leading causes of death before and after garbage code redistribution: Hungary - 2015.

Causes are connected by arrows before and after redistribution. Infectious diseases are shown in red, non-communicable causes in blue, and injuries in green.

In addition to garbage redistribution, the diagram also reflects the deaths moved during misassignment correction for Alzheimer's disease and other dementias.

Leading causes Before Redistribution	Deaths	Leading causes After Redistribution	Deaths
1 Ischemic heart disease	33006.0	1 Ischemic heart disease	36727.67
2 Tracheal, bronchus, and lung cancer	8805.0	2 Ischemic stroke	10582.15
3 Ischemic stroke	8262.0	3 Tracheal, bronchus, and lung cancer	9103.65
4 Chronic obstructive pulmonary disease	6078.0	4 Chronic obstructive pulmonary disease	6580.68
5 Hypertensive heart disease	5611.0	5 Hypertensive heart disease	6483.48
6 Colon and rectum cancer	5045.0	6 Colon and rectum cancer	5485.16
7 Alzheimer's disease and other dementias	3775.0	7 Alzheimer's disease and other dementias	4150.4
8 Cirrhosis and other chronic liver diseases	3165.0	8 Cirrhosis and other chronic liver diseases	3519.81
9 Breast cancer	2254.0	9 Intracerebral hemorrhage	2603.02
10 Intracerebral hemorrhage	2122.0	10 Breast cancer	2390.38
11 Pancreatic cancer	1978.0	11 Diabetes mellitus type 2	2322.89
12 Falls	1838.0	12 Pancreatic cancer	2128.54
13 Self-harm by other specified means	1813.0	13 Falls	1973.59
14 Diabetes mellitus type 2	1688.0	14 Self-harm by other specified means	1947.23
15 Stomach cancer	1504.0	15 Stomach cancer	1630.3
16 Prostate cancer	1269.0	16 Alcoholic cardiomyopathy	1396.17
17 Chronic kidney disease due to hypertension	1220.0	17 Prostate cancer	1395.28
18 Bladder cancer	971.0	18 Chronic kidney disease due to hypertension	1378.41
19 Non-rheumatic calcific aortic valve disease	956.0	19 Other lower respiratory infections	1289.59
20 Kidney cancer	758.0	20 Bladder cancer	1039.03
40 Other lower respiratory infections		21 Non-rheumatic calcific aortic valve disease	
57 Alcoholic cardiomyopathy		24 Kidney cancer	

Leading causes of death before and after garbage code redistribution: Ireland - 2015.

Causes are connected by arrows before and after redistribution. Infectious diseases are shown in red, non-communicable causes in blue, and injuries in green.
In addition to garbage redistribution, the diagram also reflects the deaths moved during misassignment correction for Alzheimer's disease and other dementias.

Leading causes Before Redistribution	Deaths
1 Ischemic heart disease	4492.0
2 Alzheimer's disease and other dementias	2204.0
3 Tracheal, bronchus, and lung cancer	1834.0
4 Chronic obstructive pulmonary disease	1572.0
5 Colon and rectum cancer	1019.0
6 Breast cancer	682.0
7 Pancreatic cancer	549.0
8 Prostate cancer	527.0
9 Atrial fibrillation and flutter	464.0
10 Self-harm by other specified means	408.0
11 Esophageal cancer	406.0
12 Parkinson's disease	374.0
13 Intracerebral hemorrhage	367.0
14 Interstitial lung disease and pulmonary sarcoidosis	351.0
15 Stomach cancer	329.0
16 Brain and central nervous system cancer	326.0
17 Cirrhosis and other chronic liver diseases	306.0
18 Ovarian cancer	269.0
19 Liver cancer	258.0
20 Falls	244.0
23 Ischemic stroke	
30 Diabetes mellitus type 2	
78 Influenza	

Leading causes After Redistribution	Deaths
1 Ischemic heart disease	5156.87
2 Tracheal, bronchus, and lung cancer	1979.76
3 Chronic obstructive pulmonary disease	1919.28
4 Alzheimer's disease and other dementias	1868.91
5 Ischemic stroke	1453.28
6 Colon and rectum cancer	1166.0
7 Influenza	898.56
8 Breast cancer	750.24
9 Pancreatic cancer	619.12
10 Prostate cancer	599.48
11 Intracerebral hemorrhage	521.97
12 Atrial fibrillation and flutter	473.1
13 Esophageal cancer	461.58
14 Self-harm by other specified means	437.93
15 Stomach cancer	415.24
16 Diabetes mellitus type 2	401.34
17 Parkinson's disease	400.69
18 Interstitial lung disease and pulmonary sarcoidosis	391.94
19 Cirrhosis and other chronic liver diseases	381.3
20 Brain and central nervous system cancer	353.01
22 Falls	
24 Ovarian cancer	
25 Liver cancer	

Leading causes of death before and after garbage code redistribution: Iran (Islamic Republic of) - 2015.

Causes are connected by arrows before and after redistribution. Infectious diseases are shown in red, non-communicable causes in blue, and injuries in green.

In addition to garbage redistribution, the diagram also reflects the deaths moved during misassignment correction for Alzheimer's disease and other dementias.

Leading causes Before Redistribution	Deaths	Leading causes After Redistribution	Deaths
1 Ischemic heart disease	49274.0	1 Ischemic heart disease	69619.34
2 Ischemic stroke	18657.0	2 Ischemic stroke	26998.51
3 Hypertensive heart disease	9930.0	3 Diabetes mellitus type 2	15524.87
4 Neonatal preterm birth	9885.0	4 Hypertensive heart disease	13842.73
5 Stomach cancer	7317.0	5 Neonatal preterm birth	10752.32
6 Pedestrian road injuries	6004.0	6 Alzheimer's disease and other dementias	10686.6
7 Tracheal, bronchus, and lung cancer	5157.0	7 Chronic obstructive pulmonary disease	9395.58
8 Chronic obstructive pulmonary disease	4717.0	8 Stomach cancer	8873.28
9 Alzheimer's disease and other dementias	3730.0	9 Pedestrian road injuries	7595.08
10 Chronic kidney disease due to hypertension	3398.0	10 Other lower respiratory infections	6744.38
11 Colon and rectum cancer	3385.0	11 Tracheal, bronchus, and lung cancer	6577.85
12 Brain and central nervous system cancer	3071.0	12 Intracerebral hemorrhage	6207.02
13 Intracerebral hemorrhage	2908.0	13 Chronic kidney disease due to hypertension	5848.94
14 Breast cancer	2839.0	14 Motor vehicle road injuries	5177.29
15 Prostate cancer	2651.0	15 Colon and rectum cancer	4798.99
16 Motor vehicle road injuries	2649.0	16 Cirrhosis and other chronic liver diseases	4185.49
17 Congenital heart anomalies	2569.0	17 Breast cancer	3520.56
18 Other neonatal disorders	2463.0	18 Brain and central nervous system cancer	3480.85
19 Chronic kidney disease	2274.0	19 Congenital heart anomalies	3263.95
20 Cirrhosis and other chronic liver diseases	2204.0	20 Prostate cancer	3177.96
29 Diabetes mellitus type 2		22 Chronic kidney disease	
38 Other lower respiratory infections		23 Other neonatal disorders	

Leading causes of death before and after garbage code redistribution: Iraq - 2015.

Causes are connected by arrows before and after redistribution. Infectious diseases are shown in red, non-communicable causes in blue, and injuries in green.
In addition to garbage redistribution, the diagram also reflects the deaths moved during misassignment correction for Alzheimer's disease and other dementias.

Leading causes Before Redistribution	Deaths
1 Ischemic heart disease	9580.0
2 Conflict and terrorism	5431.0
3 Neonatal preterm birth	5311.0
4 Chronic kidney disease	3925.0
5 Ischemic stroke	1834.0
6 Tracheal, bronchus, and lung cancer	1584.0
7 Congenital heart anomalies	1470.0
8 Other congenital birth defects	1268.0
9 Intracerebral hemorrhage	1264.0
10 Neonatal encephalopathy due to birth asphyxia and trauma	1199.0
11 Breast cancer	1089.0
12 Brain and central nervous system cancer	1012.0
13 Neonatal sepsis and other neonatal infections	896.0
14 Other exposure to mechanical forces	857.0
15 Electrocution	731.0
16 Leukemia	709.0
17 Liver cancer	639.0
18 Bladder cancer	495.0
19 Colon and rectum cancer	484.0
20 Pancreatic cancer	479.0
21 Cirrhosis and other chronic liver diseases	
23 Other lower respiratory infections	
30 Physical violence by firearm	
35 Hypertensive heart disease	
45 Motor vehicle road injuries	
105 Diabetes mellitus type 2	
130 Alzheimer's disease and other dementias	

Leading causes After Redistribution	Deaths
1 Ischemic heart disease	20463.95
2 Ischemic stroke	9252.0
3 Conflict and terrorism	6423.3
4 Neonatal preterm birth	6181.16
5 Chronic kidney disease	5275.0
6 Intracerebral hemorrhage	5241.45
7 Diabetes mellitus type 2	4582.84
8 Other lower respiratory infections	2712.27
9 Hypertensive heart disease	2682.14
10 Congenital heart anomalies	2150.48
11 Tracheal, bronchus, and lung cancer	1968.87
12 Alzheimer's disease and other dementias	1965.75
13 Other congenital birth defects	1653.77
14 Cirrhosis and other chronic liver diseases	1641.95
15 Motor vehicle road injuries	1636.67
16 Physical violence by firearm	1389.76
17 Breast cancer	1353.23
18 Neonatal encephalopathy due to birth asphyxia and trauma	1326.48
19 Brain and central nervous system cancer	1289.37
20 Other exposure to mechanical forces	1185.1
21 Neonatal sepsis and other neonatal infections	
23 Leukemia	
26 Electrocution	
28 Liver cancer	
32 Colon and rectum cancer	
34 Pancreatic cancer	
36 Bladder cancer	

Leading causes of death before and after garbage code redistribution: Iceland - 2015.

Causes are connected by arrows before and after redistribution. Infectious diseases are shown in red, non-communicable causes in blue, and injuries in green.

In addition to garbage redistribution, the diagram also reflects the deaths moved during misassignment correction for Alzheimer's disease and other dementias.

Leading causes Before Redistribution	Deaths	Leading causes After Redistribution	Deaths
1 Ischemic heart disease	314.0	1 Ischemic heart disease	406.93
2 Alzheimer's disease and other dementias	215.0	2 Alzheimer's disease and other dementias	181.72
3 Tracheal, bronchus, and lung cancer	117.0	3 Tracheal, bronchus, and lung cancer	130.46
4 Chronic obstructive pulmonary disease	85.0	4 Ischemic stroke	123.35
5 Colon and rectum cancer	70.0	5 Chronic obstructive pulmonary disease	104.71
6 Prostate cancer	56.0	6 Colon and rectum cancer	78.38
7 Breast cancer	55.0	7 Prostate cancer	62.59
8 Pancreatic cancer	53.0	8 Breast cancer	60.95
9 Atrial fibrillation and flutter	49.0	9 Pancreatic cancer	58.53
10 Self-harm by other specified means	39.0	10 Atrial fibrillation and flutter	55.78
11 Ischemic stroke	38.0	11 Self-harm by other specified means	46.21
12 Non-rheumatic calcific aortic valve disease	30.0	12 Intracerebral hemorrhage	40.3
13 Parkinson's disease	30.0	13 Non-rheumatic calcific aortic valve disease	39.35
14 Intracerebral hemorrhage	29.0	14 Falls	38.68
15 Kidney cancer	24.0	15 Other lower respiratory infections	37.84
16 Stomach cancer	23.0	16 Parkinson's disease	37.06
17 Brain and central nervous system cancer	22.0	17 Influenza	27.85
18 Liver cancer	19.0	18 Stomach cancer	26.55
19 Leukemia	19.0	19 Kidney cancer	26.51
20 Other neurological disorders	17.0	20 Brain and central nervous system cancer	24.6
22 Falls		23 Liver cancer	
54 Influenza		24 Leukemia	
70 Other lower respiratory infections		29 Other neurological disorders	

Leading causes of death before and after garbage code redistribution: Israel - 2015.

Causes are connected by arrows before and after redistribution. Infectious diseases are shown in red, non-communicable causes in blue, and injuries in green.
In addition to garbage redistribution, the diagram also reflects the deaths moved during misassignment correction for Alzheimer's disease and other dementias.

Leading causes Before Redistribution	Deaths
1 Ischemic heart disease	3915.0
2 Alzheimer's disease and other dementias	2024.0
3 Tracheal, bronchus, and lung cancer	1930.0
4 Colon and rectum cancer	1296.0
5 Chronic obstructive pulmonary disease	1195.0
6 Breast cancer	1052.0
7 Pancreatic cancer	950.0
8 Chronic kidney disease	700.0
9 Diabetes mellitus type 2	560.0
10 Stomach cancer	496.0
11 Urinary tract infections and interstitial nephritis	484.0
12 Atrial fibrillation and flutter	476.0
13 Brain and central nervous system cancer	475.0
14 Prostate cancer	471.0
15 Intracerebral hemorrhage	466.0
16 Cirrhosis and other chronic liver diseases	406.0
17 Bladder cancer	396.0
18 Non-rheumatic calcific aortic valve disease	351.0
19 Decubitus ulcer	344.0
20 Parkinson's disease	294.0
22 Chronic kidney disease due to hypertension	
42 Other non-Hodgkin lymphoma	
48 Ischemic stroke	
50 Chronic kidney disease due to diabetes mellitus type 2	
127 Other lower respiratory infections	

Leading causes After Redistribution	Deaths
1 Ischemic heart disease	4939.93
2 Alzheimer's disease and other dementias	3116.35
3 Diabetes mellitus type 2	2443.26
4 Tracheal, bronchus, and lung cancer	2294.12
5 Colon and rectum cancer	1709.59
6 Ischemic stroke	1698.15
7 Chronic obstructive pulmonary disease	1523.15
8 Breast cancer	1238.59
9 Pancreatic cancer	1144.4
10 Intracerebral hemorrhage	1106.25
11 Chronic kidney disease	1049.48
12 Other lower respiratory infections	813.41
13 Urinary tract infections and interstitial nephritis	750.46
14 Chronic kidney disease due to hypertension	747.47
15 Atrial fibrillation and flutter	688.52
16 Stomach cancer	686.79
17 Chronic kidney disease due to diabetes mellitus type 2	669.73
18 Other non-Hodgkin lymphoma	622.03
19 Prostate cancer	600.05
20 Brain and central nervous system cancer	583.7
21 Parkinson's disease	
22 Cirrhosis and other chronic liver diseases	
23 Decubitus ulcer	
24 Bladder cancer	
25 Non-rheumatic calcific aortic valve disease	

Leading causes of death before and after garbage code redistribution: Italy - 2015.

Causes are connected by arrows before and after redistribution. Infectious diseases are shown in red, non-communicable causes in blue, and injuries in green.
In addition to garbage redistribution, the diagram also reflects the deaths moved during misassignment correction for Alzheimer’s disease and other dementias.

Leading causes Before Redistribution	Deaths
1 Ischemic heart disease	73168.0
2 Tracheal, bronchus, and lung cancer	33987.0
3 Alzheimer's disease and other dementias	33490.0
4 Hypertensive heart disease	23106.0
5 Chronic obstructive pulmonary disease	21521.0
6 Colon and rectum cancer	19008.0
7 Breast cancer	12463.0
8 Pancreatic cancer	11506.0
9 Intracerebral hemorrhage	10733.0
10 Cirrhosis and other chronic liver diseases	9637.0
11 Stomach cancer	9443.0
12 Atrial fibrillation and flutter	9143.0
13 Prostate cancer	7757.0
14 Bladder cancer	6705.0
15 Liver cancer	6323.0
16 Parkinson's disease	6147.0
17 Chronic kidney disease due to hypertension	5867.0
18 Ischemic stroke	5153.0
19 Chronic kidney disease	5141.0
20 Brain and central nervous system cancer	4158.0
21 Diabetes mellitus type 2	
22 Falls	

Leading causes After Redistribution	Deaths
1 Ischemic heart disease	93026.1
2 Alzheimer's disease and other dementias	63542.39
3 Ischemic stroke	42634.18
4 Tracheal, bronchus, and lung cancer	38270.1
5 Hypertensive heart disease	30924.29
6 Chronic obstructive pulmonary disease	25966.78
7 Colon and rectum cancer	23704.5
8 Diabetes mellitus type 2	21633.04
9 Intracerebral hemorrhage	19386.03
10 Breast cancer	14552.55
11 Pancreatic cancer	13892.63
12 Stomach cancer	12684.75
13 Cirrhosis and other chronic liver diseases	11372.29
14 Falls	11357.12
15 Prostate cancer	10039.79
16 Atrial fibrillation and flutter	9162.26
17 Chronic kidney disease due to hypertension	8693.32
18 Parkinson's disease	8369.69
19 Bladder cancer	8190.94
20 Liver cancer	7331.49
21 Chronic kidney disease	
28 Brain and central nervous system cancer	

Leading causes of death before and after garbage code redistribution: Jamaica - 2014.

Causes are connected by arrows before and after redistribution. Infectious diseases are shown in red, non-communicable causes in blue, and injuries in green.
In addition to garbage redistribution, the diagram also reflects the deaths moved during misassignment correction for Alzheimer’s disease and other dementias.

Leading causes Before Redistribution	Deaths
1 Ischemic heart disease	1568.0
2 Hypertensive heart disease	716.0
3 Prostate cancer	676.0
4 HIV/AIDS resulting in other diseases	586.0
5 Tracheal, bronchus, and lung cancer	401.0
6 Breast cancer	399.0
7 Chronic obstructive pulmonary disease	385.0
8 Intracerebral hemorrhage	366.0
9 Colon and rectum cancer	353.0
10 Neonatal preterm birth	242.0
11 Chronic kidney disease due to hypertension	217.0
12 Decubitus ulcer	203.0
13 Stomach cancer	171.0
14 Alzheimer's disease and other dementias	170.0
15 Cervical cancer	167.0
16 Diabetes mellitus type 2	148.0
17 Urinary tract infections and interstitial nephritis	136.0
18 Ischemic stroke	130.0
19 Neonatal encephalopathy due to birth asphyxia and trauma	115.0
20 Endocrine, metabolic, blood, and immune disorders	113.0

25 Atrial fibrillation and flutter

158 Other lower respiratory infections

Leading causes After Redistribution	Deaths
1 Diabetes mellitus type 2	1934.51
2 Ischemic heart disease	1793.5
3 Ischemic stroke	1576.99
4 Intracerebral hemorrhage	1105.33
5 Hypertensive heart disease	861.48
6 Prostate cancer	774.2
7 HIV/AIDS resulting in other diseases	623.34
8 Alzheimer's disease and other dementias	586.17
9 Tracheal, bronchus, and lung cancer	469.0
10 Breast cancer	448.42
11 Colon and rectum cancer	446.96
12 Chronic obstructive pulmonary disease	431.72
13 Chronic kidney disease due to hypertension	331.52
14 Other lower respiratory infections	330.59
15 Neonatal preterm birth	275.69
16 Stomach cancer	246.37
17 Cervical cancer	206.96
18 Urinary tract infections and interstitial nephritis	203.52
19 Atrial fibrillation and flutter	187.85
20 Decubitus ulcer	174.14

25 Neonatal encephalopathy due to birth asphyxia and trauma

30 Endocrine, metabolic, blood, and immune disorders

Leading causes of death before and after garbage code redistribution: Jordan - 2015.

Causes are connected by arrows before and after redistribution. Infectious diseases are shown in red, non-communicable causes in blue, and injuries in green.
In addition to garbage redistribution, the diagram also reflects the deaths moved during misassignment correction for Alzheimer's disease and other dementias.

Leading causes Before Redistribution	Deaths
1 Ischemic heart disease	1895.0
2 Ischemic stroke	1377.0
3 Hypertensive heart disease	808.0
4 Neonatal preterm birth	518.0
5 Tracheal, bronchus, and lung cancer	505.0
6 Intracerebral hemorrhage	455.0
7 Chronic kidney disease due to hypertension	395.0
8 Breast cancer	367.0
9 Colon and rectum cancer	325.0
10 Congenital heart anomalies	307.0
11 Chronic obstructive pulmonary disease	222.0
12 Leukemia	219.0
13 Chronic kidney disease	218.0
14 Other congenital birth defects	215.0
15 Cirrhosis and other chronic liver diseases	209.0
16 Pancreatic cancer	137.0
17 Neonatal sepsis and other neonatal infections	134.0
18 Stomach cancer	113.0
19 Brain and central nervous system cancer	109.0
20 Other neoplasms	107.0
42 Pedestrian road injuries	
64 Alzheimer's disease and other dementias	
111 Other lower respiratory infections	
140 Diabetes mellitus type 2	
145 Motor vehicle road injuries	

Leading causes After Redistribution	Deaths
1 Ischemic heart disease	3204.12
2 Ischemic stroke	1716.4
3 Diabetes mellitus type 2	1198.34
4 Hypertensive heart disease	1184.42
5 Intracerebral hemorrhage	643.36
6 Chronic kidney disease due to hypertension	584.47
7 Tracheal, bronchus, and lung cancer	576.3
8 Neonatal preterm birth	571.08
9 Other lower respiratory infections	483.46
10 Alzheimer's disease and other dementias	447.66
11 Colon and rectum cancer	443.75
12 Breast cancer	441.88
13 Motor vehicle road injuries	379.64
14 Cirrhosis and other chronic liver diseases	357.84
15 Congenital heart anomalies	336.69
16 Chronic obstructive pulmonary disease	299.01
17 Chronic kidney disease	270.89
18 Other congenital birth defects	267.44
19 Leukemia	254.88
20 Pedestrian road injuries	254.1
22 Stomach cancer	
24 Pancreatic cancer	
25 Neonatal sepsis and other neonatal infections	
29 Brain and central nervous system cancer	
35 Other neoplasms	

Leading causes of death before and after garbage code redistribution: Japan - 2015.

Causes are connected by arrows before and after redistribution. Infectious diseases are shown in red, non-communicable causes in blue, and injuries in green.
In addition to garbage redistribution, the diagram also reflects the deaths moved during misassignment correction for Alzheimer's disease and other dementias.

Leading causes Before Redistribution	Deaths
1 Tracheal, bronchus, and lung cancer	75305.0
2 Ischemic heart disease	72048.0
3 Ischemic stroke	64924.0
4 Colon and rectum cancer	50555.0
5 Stomach cancer	47107.0
6 Intracerebral hemorrhage	32662.0
7 Pancreatic cancer	32027.0
8 Liver cancer	28657.0
9 Alzheimer's disease and other dementias	25109.0
10 Self-harm by other specified means	23068.0
11 Gallbladder and biliary tract cancer	18240.0
12 Cirrhosis and other chronic liver diseases	17218.0
13 Interstitial lung disease and pulmonary sarcoidosis	17101.0
14 Aortic aneurysm	16965.0
15 Chronic obstructive pulmonary disease	15820.0
16 Chronic kidney disease	15813.0
17 Breast cancer	13825.0
18 Subarachnoid hemorrhage	12722.0
19 Esophageal cancer	11789.0
20 Prostate cancer	11365.0
28 Parkinson's disease	
54 Influenza	
63 Other lower respiratory infections	
100 Pneumococcal pneumonia	

Leading causes After Redistribution	Deaths
1 Alzheimer's disease and other dementias	141017.03
2 Ischemic heart disease	113624.62
3 Tracheal, bronchus, and lung cancer	86210.68
4 Ischemic stroke	82407.27
5 Colon and rectum cancer	62150.02
6 Stomach cancer	58642.38
7 Influenza	44801.87
8 Other lower respiratory infections	42454.79
9 Intracerebral hemorrhage	39325.86
10 Pancreatic cancer	35925.22
11 Liver cancer	32589.55
12 Chronic obstructive pulmonary disease	26393.34
13 Self-harm by other specified means	26027.49
14 Chronic kidney disease	23027.65
15 Cirrhosis and other chronic liver diseases	21814.56
16 Gallbladder and biliary tract cancer	21087.26
17 Interstitial lung disease and pulmonary sarcoidosis	19736.54
18 Aortic aneurysm	19714.06
19 Pneumococcal pneumonia	18651.1
20 Parkinson's disease	16264.77
21 Breast cancer	
22 Subarachnoid hemorrhage	
23 Esophageal cancer	
26 Prostate cancer	

Leading causes of death before and after garbage code redistribution: Kazakhstan - 2015.

Causes are connected by arrows before and after redistribution. Infectious diseases are shown in red, non-communicable causes in blue, and injuries in green.

In addition to garbage redistribution, the diagram also reflects the deaths moved during misassignment correction for Alzheimer's disease and other dementias.

Leading causes Before Redistribution	Deaths	Leading causes After Redistribution	Deaths
1 Chronic obstructive pulmonary disease	12895.0	1 Ischemic heart disease	16697.32
2 Ischemic heart disease	12572.0	2 Chronic obstructive pulmonary disease	14974.54
3 Cirrhosis and other chronic liver diseases	9251.0	3 Ischemic stroke	11072.67
4 Ischemic stroke	3987.0	4 Cirrhosis and other chronic liver diseases	10731.62
5 Alzheimer's disease and other dementias	3567.0	5 Intracerebral hemorrhage	7218.63
6 Intracerebral hemorrhage	3197.0	6 Other lower respiratory infections	4635.53
7 Diabetes mellitus type 2	2848.0	7 Self-harm by other specified means	4209.22
8 Self-harm by other specified means	2829.0	8 Diabetes mellitus type 2	3929.99
9 Tracheal, bronchus, and lung cancer	2715.0	9 Other cardiomyopathy	3107.54
10 Urinary tract infections and interstitial nephritis	2297.0	10 Tracheal, bronchus, and lung cancer	3099.1
11 Stomach cancer	1941.0	11 Urinary tract infections and interstitial nephritis	3029.61
12 Colon and rectum cancer	1596.0	12 Stomach cancer	2217.05
13 Motor vehicle road injuries	1500.0	13 Alzheimer's disease and other dementias	1994.39
14 Breast cancer	1307.0	14 Colon and rectum cancer	1863.78
15 Neonatal preterm birth	1163.0	15 Motor vehicle road injuries	1764.38
16 Alcohol use disorders	1117.0	16 Chronic kidney disease	1523.06
17 Chronic kidney disease	1096.0	17 Breast cancer	1497.43
18 Hypertensive heart disease	1047.0	18 Hypertensive heart disease	1293.46
19 Pancreatitis	982.0	19 Alcohol use disorders	1280.53
20 Other musculoskeletal disorders	965.0	20 Neonatal preterm birth	1223.39
22 Other lower respiratory infections		21 Pancreatitis	
173 Other cardiomyopathy		23 Other musculoskeletal disorders	

Leading causes of death before and after garbage code redistribution: Kyrgyzstan - 2015.

Causes are connected by arrows before and after redistribution. Infectious diseases are shown in red, non-communicable causes in blue, and injuries in green.
In addition to garbage redistribution, the diagram also reflects the deaths moved during misassignment correction for Alzheimer's disease and other dementias.

Leading causes Before Redistribution	Deaths
1 Ischemic heart disease	11415.0
2 Cirrhosis and other chronic liver diseases	1923.0
3 Ischemic stroke	1348.0
4 Chronic obstructive pulmonary disease	1122.0
5 Other neonatal disorders	1010.0
6 Stomach cancer	700.0
7 Intracerebral hemorrhage	608.0
8 Neonatal preterm birth	589.0
9 Neonatal encephalopathy due to birth asphyxia and trauma	532.0
10 Tracheal, bronchus, and lung cancer	454.0
11 Drug-susceptible tuberculosis	453.0
12 Self-harm by other specified means	410.0
13 Hypertensive heart disease	390.0
14 Pedestrian road injuries	341.0
15 Alcohol use disorders	315.0
16 Cervical cancer	261.0
17 Breast cancer	258.0
18 Motor vehicle road injuries	254.0
19 Alcoholic cardiomyopathy	248.0
20 Colon and rectum cancer	246.0
70 Other lower respiratory infections	
98 Alzheimer's disease and other dementias	

Leading causes After Redistribution	Deaths
1 Ischemic heart disease	11702.63
2 Ischemic stroke	2559.17
3 Cirrhosis and other chronic liver diseases	2106.1
4 Intracerebral hemorrhage	1629.62
5 Chronic obstructive pulmonary disease	1161.38
6 Other neonatal disorders	1011.99
7 Stomach cancer	846.06
8 Neonatal preterm birth	601.13
9 Self-harm by other specified means	560.0
10 Alzheimer's disease and other dementias	548.74
11 Neonatal encephalopathy due to birth asphyxia and trauma	536.83
12 Motor vehicle road injuries	513.65
13 Drug-susceptible tuberculosis	499.7
14 Tracheal, bronchus, and lung cancer	494.24
15 Pedestrian road injuries	493.1
16 Other lower respiratory infections	490.02
17 Hypertensive heart disease	410.51
18 Alcoholic cardiomyopathy	364.09
19 Alcohol use disorders	346.82
20 Colon and rectum cancer	295.47
21 Cervical cancer	
22 Breast cancer	

Leading causes of death before and after garbage code redistribution: Kiribati - 2001.

Causes are connected by arrows before and after redistribution. Infectious diseases are shown in red, non-communicable causes in blue, and injuries in green.
In addition to garbage redistribution, the diagram also reflects the deaths moved during misassignment correction for Alzheimer's disease and other dementias.

Leading causes Before Redistribution	Deaths
1 Cirrhosis and other chronic liver diseases	26.0
2 Other unspecified infectious diseases	24.0
3 Dengue	23.0
4 Protein-energy malnutrition	15.0
5 Asthma	10.0
6 Ulcerative colitis	9.0
7 Drug-susceptible tuberculosis	7.0
8 Other neonatal disorders	7.0
9 Peptic ulcer disease	5.0
10 Influenza	4.0
11 Cervical cancer	4.0
12 Physical violence by sharp object	4.0
13 Paralytic ileus and intestinal obstruction	3.0
14 Leukemia	3.0
15 Other endocrine, metabolic, blood, and immune disorders	3.0
16 Other lower respiratory infections	2.0
17 Gastritis and duodenitis	2.0
18 Idiopathic epilepsy	2.0
19 Tracheal, bronchus, and lung cancer	2.0
20 Stomach cancer	2.0
24 Ischemic heart disease	
25 Other cardiovascular and circulatory diseases (internal)	
39 HIV/AIDS resulting in other diseases	
40 Diarrheal diseases	
41 Diabetes mellitus type 2	
42 Ischemic stroke	
43 Intracerebral hemorrhage	

Leading causes After Redistribution	Deaths
1 Intracerebral hemorrhage	38.99
2 Cirrhosis and other chronic liver diseases	38.78
3 Diabetes mellitus type 2	34.46
4 Other unspecified infectious diseases	32.6
5 Dengue	29.2
6 Influenza	23.93
7 Other lower respiratory infections	18.84
8 Protein-energy malnutrition	18.26
9 Ischemic heart disease	17.89
10 Ulcerative colitis	16.32
11 Asthma	13.75
12 Drug-susceptible tuberculosis	12.49
13 Ischemic stroke	10.66
14 Peptic ulcer disease	9.54
15 Other neonatal disorders	9.17
16 Diarrheal diseases	8.57
17 Other endocrine, metabolic, blood, and immune disorders	6.16
18 Other cardiovascular and circulatory diseases (internal)	6.12
19 Physical violence by sharp object	6.06
20 HIV/AIDS resulting in other diseases	6.03
21 Tracheal, bronchus, and lung cancer	
22 Cervical cancer	
24 Leukemia	
25 Stomach cancer	
28 Paralytic ileus and intestinal obstruction	
32 Idiopathic epilepsy	
33 Gastritis and duodenitis	

Leading causes of death before and after garbage code redistribution: Saint Kitts and Nevis - 2015.

Causes are connected by arrows before and after redistribution. Infectious diseases are shown in red, non-communicable causes in blue, and injuries in green.
In addition to garbage redistribution, the diagram also reflects the deaths moved during misassignment correction for Alzheimer’s disease and other dementias.

Leading causes Before Redistribution	Deaths
1 Ischemic heart disease	30.0
2 Physical violence by firearm	21.0
3 Prostate cancer	13.0
4 Chronic kidney disease due to hypertension	12.0
5 Colon and rectum cancer	12.0
6 Breast cancer	11.0
7 Hypertensive heart disease	9.0
8 Pancreatic cancer	8.0
9 Neonatal preterm birth	7.0
10 Tracheal, bronchus, and lung cancer	7.0
11 Unintentional firearm injuries	6.0
12 Alcohol use disorders	5.0
13 Neonatal encephalopathy due to birth asphyxia and trauma	5.0
14 Chronic kidney disease	5.0
15 Cirrhosis and other chronic liver diseases	4.0
16 Congenital heart anomalies	3.0
17 Urinary tract infections and interstitial nephritis	3.0
18 Atrial fibrillation and flutter	3.0
19 Brain and central nervous system cancer	3.0
20 Intracerebral hemorrhage	3.0
27 Alzheimer's disease and other dementias	
43 Ischemic stroke	
52 Other cardiomyopathy	
61 Diabetes mellitus type 2	

Leading causes After Redistribution	Deaths
1 Ischemic heart disease	39.35
2 Diabetes mellitus type 2	29.74
3 Ischemic stroke	28.18
4 Physical violence by firearm	21.03
5 Intracerebral hemorrhage	18.9
6 Colon and rectum cancer	14.98
7 Prostate cancer	14.57
8 Chronic kidney disease due to hypertension	13.39
9 Breast cancer	12.29
10 Hypertensive heart disease	11.63
11 Tracheal, bronchus, and lung cancer	8.82
12 Pancreatic cancer	8.56
13 Neonatal preterm birth	7.0
14 Cirrhosis and other chronic liver diseases	6.47
15 Alzheimer's disease and other dementias	6.04
16 Unintentional firearm injuries	6.0
17 Other cardiomyopathy	5.88
18 Chronic kidney disease	5.35
19 Alcohol use disorders	5.29
20 Neonatal encephalopathy due to birth asphyxia and trauma	5.0
21 Atrial fibrillation and flutter	
27 Brain and central nervous system cancer	
28 Congenital heart anomalies	
29 Urinary tract infections and interstitial nephritis	

Leading causes of death before and after garbage code redistribution: Republic of Korea - 2015.

Causes are connected by arrows before and after redistribution. Infectious diseases are shown in red, non-communicable causes in blue, and injuries in green.

In addition to garbage redistribution, the diagram also reflects the deaths moved during misassignment correction for Alzheimer's disease and other dementias.

Leading causes Before Redistribution	Deaths	Leading causes After Redistribution	Deaths
1 Tracheal, bronchus, and lung cancer	17399.0	1 Ischemic heart disease	21192.91
2 Ischemic heart disease	14724.0	2 Tracheal, bronchus, and lung cancer	20244.36
3 Self-harm by other specified means	13431.0	3 Ischemic stroke	16605.9
4 Liver cancer	11311.0	4 Alzheimer's disease and other dementias	16259.42
5 Alzheimer's disease and other dementias	9516.0	5 Self-harm by other specified means	15230.95
6 Stomach cancer	8526.0	6 Other lower respiratory infections	14616.29
7 Colon and rectum cancer	8382.0	7 Liver cancer	12622.25
8 Ischemic stroke	7945.0	8 Diabetes mellitus type 2	11438.19
9 Cirrhosis and other chronic liver diseases	7217.0	9 Colon and rectum cancer	10355.06
10 Diabetes mellitus type 2	6700.0	10 Stomach cancer	10274.52
11 Chronic obstructive pulmonary disease	5697.0	11 Intracerebral hemorrhage	9393.7
12 Pancreatic cancer	5439.0	12 Cirrhosis and other chronic liver diseases	8834.81
13 Intracerebral hemorrhage	5328.0	13 Chronic obstructive pulmonary disease	7955.42
14 Gallbladder and biliary tract cancer	4211.0	14 Pancreatic cancer	6340.02
15 Chronic kidney disease	3706.0	15 Chronic kidney disease	5005.43
16 Parkinson's disease	3401.0	16 Gallbladder and biliary tract cancer	4939.37
17 Drug-susceptible tuberculosis	2406.0	17 Falls	4571.72
18 Breast cancer	2355.0	18 Hypertensive heart disease	3577.27
19 Falls	2320.0	19 Parkinson's disease	3450.76
20 Hypertensive heart disease	2260.0	20 Drug-susceptible tuberculosis	2824.88
29 Other lower respiratory infections		23 Breast cancer	

Leading causes of death before and after garbage code redistribution: Kuwait - 2015.

Causes are connected by arrows before and after redistribution. Infectious diseases are shown in red, non-communicable causes in blue, and injuries in green.
In addition to garbage redistribution, the diagram also reflects the deaths moved during misassignment correction for Alzheimer's disease and other dementias.

Leading causes Before Redistribution	Deaths
1 Ischemic heart disease	1497.0
2 Ischemic stroke	235.0
3 Neonatal preterm birth	196.0
4 Intracerebral hemorrhage	145.0
5 Tracheal, bronchus, and lung cancer	115.0
6 Falls	114.0
7 Colon and rectum cancer	113.0
8 Breast cancer	104.0
9 Hypertensive heart disease	98.0
10 Cirrhosis and other chronic liver diseases	73.0
11 Self-harm by other specified means	71.0
12 Congenital heart anomalies	59.0
13 Pancreatic cancer	58.0
14 Urinary tract infections and interstitial nephritis	54.0
15 Opioid use disorders	44.0
16 Stomach cancer	41.0
17 Adverse effects of medical treatment	41.0
18 Other non-Hodgkin lymphoma	39.0
19 Chronic kidney disease	36.0
20 Brain and central nervous system cancer	33.0
33 Pedestrian road injuries	
42 Influenza	
68 Motor vehicle road injuries	
73 Other lower respiratory infections	
104 Diabetes mellitus type 2	
119 Alzheimer's disease and other dementias	

Leading causes After Redistribution	Deaths
1 Ischemic heart disease	1701.48
2 Other lower respiratory infections	407.1
3 Motor vehicle road injuries	315.4
4 Ischemic stroke	250.95
5 Neonatal preterm birth	214.87
6 Alzheimer's disease and other dementias	193.71
7 Hypertensive heart disease	164.76
8 Colon and rectum cancer	162.2
9 Diabetes mellitus type 2	157.37
10 Intracerebral hemorrhage	156.47
11 Pedestrian road injuries	136.58
12 Tracheal, bronchus, and lung cancer	135.95
13 Breast cancer	135.49
14 Falls	130.68
15 Congenital heart anomalies	100.34
16 Cirrhosis and other chronic liver diseases	99.39
17 Self-harm by other specified means	78.96
18 Pancreatic cancer	75.44
19 Influenza	71.6
20 Chronic kidney disease	65.74
21 Other non-Hodgkin lymphoma	
22 Stomach cancer	
23 Opioid use disorders	
24 Urinary tract infections and interstitial nephritis	
25 Adverse effects of medical treatment	
29 Brain and central nervous system cancer	

Leading causes of death before and after garbage code redistribution: Saint Lucia - 2015.

Causes are connected by arrows before and after redistribution. Infectious diseases are shown in red, non-communicable causes in blue, and injuries in green.
In addition to garbage redistribution, the diagram also reflects the deaths moved during misassignment correction for Alzheimer's disease and other dementias.

Leading causes Before Redistribution	Deaths
1 Ischemic heart disease	49.0
2 Prostate cancer	42.0
3 Hypertensive heart disease	32.0
4 Chronic obstructive pulmonary disease	31.0
5 Tracheal, bronchus, and lung cancer	23.0
6 Breast cancer	22.0
7 Intracerebral hemorrhage	21.0
8 Cirrhosis and other chronic liver diseases	17.0
9 Colon and rectum cancer	15.0
10 Neonatal preterm birth	12.0
11 Atrial fibrillation and flutter	11.0
12 Neonatal encephalopathy due to birth asphyxia and trauma	11.0
13 Decubitus ulcer	11.0
14 Diabetes mellitus type 2	10.0
15 Stomach cancer	10.0
16 Drowning	10.0
17 Aortic aneurysm	9.0
18 Physical violence by firearm	9.0
19 Peptic ulcer disease	9.0
20 Pancreatic cancer	8.0
22 Chronic kidney disease due to hypertension	
23 Cervical cancer	
59 Chronic kidney disease due to diabetes mellitus type 2	
86 Alzheimer's disease and other dementias	
93 Ischemic stroke	
106 Other lower respiratory infections	

Leading causes After Redistribution	Deaths
1 Diabetes mellitus type 2	87.89
2 Ischemic stroke	82.39
3 Ischemic heart disease	68.02
4 Intracerebral hemorrhage	61.62
5 Prostate cancer	51.19
6 Hypertensive heart disease	44.75
7 Chronic obstructive pulmonary disease	36.88
8 Alzheimer's disease and other dementias	29.22
9 Tracheal, bronchus, and lung cancer	26.42
10 Breast cancer	26.36
11 Cirrhosis and other chronic liver diseases	25.27
12 Colon and rectum cancer	19.91
13 Other lower respiratory infections	19.52
14 Chronic kidney disease due to hypertension	18.21
15 Neonatal preterm birth	17.34
16 Neonatal encephalopathy due to birth asphyxia and trauma	15.3
17 Atrial fibrillation and flutter	14.77
18 Stomach cancer	14.22
19 Cervical cancer	12.23
20 Chronic kidney disease due to diabetes mellitus type 2	11.93
23 Decubitus ulcer	
24 Drowning	
25 Aortic aneurysm	
26 Pancreatic cancer	
28 Physical violence by firearm	
29 Peptic ulcer disease	

Leading causes of death before and after garbage code redistribution: Sri Lanka - 2013.

Causes are connected by arrows before and after redistribution. Infectious diseases are shown in red, non-communicable causes in blue, and injuries in green.
In addition to garbage redistribution, the diagram also reflects the deaths moved during misassignment correction for Alzheimer's disease and other dementias.

Leading causes Before Redistribution	Deaths	Leading causes After Redistribution	Deaths
1 Chronic respiratory diseases	7538.0	1 Ischemic heart disease	14820.42
2 Ischemic heart disease	6588.0	2 Diabetes mellitus type 2	13485.74
3 Parkinson's disease	4103.92	3 Chronic respiratory diseases	10148.32
4 Stroke	3488.0	4 Hypertensive heart disease	6714.47
5 Lower respiratory infections	3347.06	5 Lower respiratory infections	5141.18
6 Hypertensive heart disease	3214.48	6 Stroke	4298.34
7 Self-harm	3164.0	7 Parkinson's disease	4089.78
8 Cirrhosis and other chronic liver diseases	2646.09	8 Cirrhosis and other chronic liver diseases	3676.07
9 Transport injuries	2451.0	9 Self-harm	3550.61
10 Chronic kidney disease	1955.02	10 Chronic kidney disease	3054.92
11 Neonatal disorders	1743.0	11 Alzheimer's disease and other dementias	2783.33
12 Tracheal, bronchus, and lung cancer	1234.96	12 Intracerebral hemorrhage	2771.77
13 Congenital birth defects	1164.0	13 Transport injuries	2719.54
14 Motor neuron disease	1138.47	14 Ischemic stroke	2561.34
15 Falls	954.0	15 Chronic kidney disease due to hypertension	2455.75
16 Chronic kidney disease due to hypertension	940.53	16 Other cardiomyopathy	2445.01
17 Drowning	894.0	17 Tracheal, bronchus, and lung cancer	2071.52
18 Other malignant neoplasms (internal)	860.16	18 Falls	1933.84
19 Alzheimer's disease and other dementias	850.35	19 Neonatal disorders	1786.1
20 Other neurological disorders	833.97	20 Chronic obstructive pulmonary disease	1751.3
43 Other cardiomyopathy		21 Motor neuron disease	
65 Ischemic stroke		22 Congenital birth defects	
176 Chronic obstructive pulmonary disease		25 Other malignant neoplasms (internal)	
177 Diabetes mellitus type 2		26 Other neurological disorders	
178 Intracerebral hemorrhage		27 Drowning	

Leading causes of death before and after garbage code redistribution: Lithuania - 2015.

Causes are connected by arrows before and after redistribution. Infectious diseases are shown in red, non-communicable causes in blue, and injuries in green.
In addition to garbage redistribution, the diagram also reflects the deaths moved during misassignment correction for Alzheimer's disease and other dementias.

Leading causes Before Redistribution	Deaths	Leading causes After Redistribution	Deaths
1 Ischemic heart disease	15566.0	1 Ischemic heart disease	15588.08
2 Ischemic stroke	3531.0	2 Ischemic stroke	4255.41
3 Tracheal, bronchus, and lung cancer	1353.0	3 Tracheal, bronchus, and lung cancer	1484.13
4 Colon and rectum cancer	986.0	4 Alzheimer's disease and other dementias	1356.74
5 Self-harm by other specified means	870.0	5 Colon and rectum cancer	1123.73
6 Cirrhosis and other chronic liver diseases	823.0	6 Self-harm by other specified means	1050.48
7 Stomach cancer	685.0	7 Cirrhosis and other chronic liver diseases	945.84
8 Chronic obstructive pulmonary disease	663.0	8 Stomach cancer	790.71
9 Intracerebral hemorrhage	608.0	9 Intracerebral hemorrhage	779.11
10 Breast cancer	576.0	10 Chronic obstructive pulmonary disease	687.31
11 Prostate cancer	544.0	11 Breast cancer	638.69
12 Hypertensive heart disease	538.0	12 Prostate cancer	614.31
13 Pancreatic cancer	471.0	13 Hypertensive heart disease	542.66
14 Falls	394.0	14 Falls	541.19
15 Kidney cancer	319.0	15 Pancreatic cancer	530.91
16 Peptic ulcer disease	305.0	16 Other lower respiratory infections	379.78
17 Alcohol use disorders	296.0	17 Kidney cancer	344.21
18 Alzheimer's disease and other dementias	289.0	18 Alcohol use disorders	322.94
19 Ovarian cancer	278.0	19 Peptic ulcer disease	310.84
20 Vascular intestinal disorders	276.0	20 Vascular intestinal disorders	309.26
76 Other lower respiratory infections		21 Ovarian cancer	

Leading causes of death before and after garbage code redistribution: Luxembourg - 2015.

Causes are connected by arrows before and after redistribution. Infectious diseases are shown in red, non-communicable causes in blue, and injuries in green.

In addition to garbage redistribution, the diagram also reflects the deaths moved during misassignment correction for Alzheimer's disease and other dementias.

Leading causes Before Redistribution	Deaths	Leading causes After Redistribution	Deaths
1 Ischemic heart disease	308.0	1 Ischemic heart disease	491.63
2 Alzheimer's disease and other dementias	252.0	2 Tracheal, bronchus, and lung cancer	271.55
3 Tracheal, bronchus, and lung cancer	228.0	3 Chronic obstructive pulmonary disease	199.2
4 Chronic obstructive pulmonary disease	154.0	4 Alzheimer's disease and other dementias	198.22
5 Colon and rectum cancer	115.0	5 Ischemic stroke	182.6
6 Breast cancer	93.0	6 Colon and rectum cancer	148.35
7 Cirrhosis and other chronic liver diseases	64.0	7 Breast cancer	109.66
8 Pancreatic cancer	61.0	8 Falls	91.95
9 Parkinson's disease	60.0	9 Intracerebral hemorrhage	82.73
10 Self-harm by other specified means	55.0	10 Atrial fibrillation and flutter	79.34
11 Prostate cancer	55.0	11 Cirrhosis and other chronic liver diseases	77.34
12 Falls	53.0	12 Pancreatic cancer	75.53
13 Atrial fibrillation and flutter	50.0	13 Diabetes mellitus type 2	70.59
14 Intracerebral hemorrhage	44.0	14 Parkinson's disease	67.15
15 Ovarian cancer	35.0	15 Self-harm by other specified means	66.68
16 Liver cancer	34.0	16 Prostate cancer	65.51
17 Bladder cancer	33.0	17 Hypertensive heart disease	56.6
18 Stomach cancer	32.0	18 Influenza	54.86
19 Ischemic stroke	31.0	19 Other lower respiratory infections	50.62
20 Alcohol use disorders	30.0	20 Stomach cancer	47.52
21 Hypertensive heart disease		24 Ovarian cancer	
39 Diabetes mellitus type 2		25 Liver cancer	
57 Other lower respiratory infections		26 Bladder cancer	
69 Influenza		30 Alcohol use disorders	

Leading causes of death before and after garbage code redistribution: Latvia - 2015.

Causes are connected by arrows before and after redistribution. Infectious diseases are shown in red, non-communicable causes in blue, and injuries in green.

In addition to garbage redistribution, the diagram also reflects the deaths moved during misassignment correction for Alzheimer's disease and other dementias.

Leading causes Before Redistribution	Deaths
1 Ischemic heart disease	8034.0
2 Ischemic stroke	3561.0
3 Tracheal, bronchus, and lung cancer	892.0
4 Hypertensive heart disease	809.0
5 Colon and rectum cancer	705.0
6 Stomach cancer	473.0
7 Intracerebral hemorrhage	463.0
8 Breast cancer	447.0
9 Cirrhosis and other chronic liver diseases	434.0
10 Prostate cancer	392.0
11 Pancreatic cancer	372.0
12 Diabetes mellitus type 2	369.0
13 Self-harm by other specified means	363.0
14 Alcohol use disorders	327.0
15 Alcoholic cardiomyopathy	290.0
16 Alzheimer's disease and other dementias	254.0
17 Chronic obstructive pulmonary disease	240.0
18 Kidney cancer	221.0
19 Bladder cancer	220.0
20 Ovarian cancer	210.0
21 Falls	
22 Other cardiomyopathy	

Leading causes After Redistribution	Deaths
1 Ischemic heart disease	8376.06
2 Ischemic stroke	4130.17
3 Tracheal, bronchus, and lung cancer	961.74
4 Alzheimer's disease and other dementias	921.67
5 Hypertensive heart disease	857.59
6 Colon and rectum cancer	772.78
7 Intracerebral hemorrhage	656.75
8 Stomach cancer	531.73
9 Alcoholic cardiomyopathy	504.14
10 Breast cancer	482.57
11 Cirrhosis and other chronic liver diseases	466.05
12 Prostate cancer	435.33
13 Self-harm by other specified means	426.64
14 Pancreatic cancer	407.13
15 Diabetes mellitus type 2	378.67
16 Other cardiomyopathy	372.63
17 Alcohol use disorders	337.82
18 Falls	273.88
19 Chronic obstructive pulmonary disease	253.04
20 Bladder cancer	236.57
21 Kidney cancer	
22 Ovarian cancer	

Leading causes of death before and after garbage code redistribution: Morocco - 2014.

Causes are connected by arrows before and after redistribution. Infectious diseases are shown in red, non-communicable causes in blue, and injuries in green.
In addition to garbage redistribution, the diagram also reflects the deaths moved during misassignment correction for Alzheimer's disease and other dementias.

Leading causes Before Redistribution	Deaths
1 Neonatal preterm birth	2590.0
2 Tracheal, bronchus, and lung cancer	1007.0
3 Ischemic heart disease	805.0
4 Drug-susceptible tuberculosis	562.0
5 Neonatal encephalopathy due to birth asphyxia and trauma	515.0
6 Cirrhosis and other chronic liver diseases	495.0
7 Neonatal sepsis and other neonatal infections	492.0
8 Breast cancer	475.0
9 Asthma	430.0
10 Brain and central nervous system cancer	352.0
11 Prostate cancer	337.0
12 Stomach cancer	337.0
13 Drowning	310.0
14 Colon and rectum cancer	300.0
15 Chronic kidney disease	276.0
16 Pancreatic cancer	230.0
17 Ischemic stroke	225.0
18 Alzheimer's disease and other dementias	204.0
19 Congenital heart anomalies	190.0
20 Leukemia	187.0
24 Diabetes mellitus type 2	
25 Falls	
31 Chronic obstructive pulmonary disease	
39 Intracerebral hemorrhage	
40 Hypertensive heart disease	
41 Other lower respiratory infections	
163 Motor vehicle road injuries	

Leading causes After Redistribution	Deaths
1 Ischemic heart disease	8041.24
2 Diabetes mellitus type 2	3324.62
3 Ischemic stroke	3073.55
4 Neonatal preterm birth	2936.6
5 Tracheal, bronchus, and lung cancer	1966.9
6 Cirrhosis and other chronic liver diseases	1758.67
7 Hypertensive heart disease	1323.99
8 Intracerebral hemorrhage	1311.02
9 Other lower respiratory infections	1167.36
10 Alzheimer's disease and other dementias	1132.32
11 Chronic kidney disease	1079.76
12 Asthma	1010.59
13 Breast cancer	988.34
14 Prostate cancer	965.29
15 Colon and rectum cancer	946.25
16 Motor vehicle road injuries	937.18
17 Stomach cancer	921.31
18 Drug-susceptible tuberculosis	913.04
19 Chronic obstructive pulmonary disease	783.96
20 Falls	652.37
21 Brain and central nervous system cancer	
23 Neonatal encephalopathy due to birth asphyxia and trauma	
24 Neonatal sepsis and other neonatal infections	
25 Pancreatic cancer	
27 Congenital heart anomalies	
30 Drowning	
34 Leukemia	

Leading causes of death before and after garbage code redistribution: Monaco - 2013.

Causes are connected by arrows before and after redistribution. Infectious diseases are shown in red, non-communicable causes in blue, and injuries in green.

In addition to garbage redistribution, the diagram also reflects the deaths moved during misassignment correction for Alzheimer’s disease and other dementias.

Leading causes Before Redistribution	Deaths
1 Tracheal, bronchus, and lung cancer	71.0
2 Alzheimer's disease and other dementias	35.0
3 Ischemic heart disease	25.0
4 Colon and rectum cancer	23.0
5 Breast cancer	23.0
6 Pancreatic cancer	19.0
7 Leukemia	19.0
8 Multiple myeloma	13.0
9 Stomach cancer	11.0
10 Liver cancer	10.0
11 Bladder cancer	10.0
12 Ovarian cancer	9.0
13 Prostate cancer	9.0
14 Cirrhosis and other chronic liver diseases	8.0
15 Aortic aneurysm	7.0
16 Esophageal cancer	7.0
17 Other non-Hodgkin lymphoma	6.0
18 Adverse effects of medical treatment	6.0
19 Cervical cancer	4.0
20 Parkinson's disease	4.0
55 Hypertensive heart disease	
56 Other cardiomyopathy	
57 Ischemic stroke	
58 Other lower respiratory infections	

Leading causes After Redistribution	Deaths
1 Tracheal, bronchus, and lung cancer	80.77
2 Ischemic heart disease	50.95
3 Alzheimer's disease and other dementias	33.71
4 Ischemic stroke	31.13
5 Colon and rectum cancer	27.99
6 Breast cancer	25.0
7 Other cardiomyopathy	22.6
8 Pancreatic cancer	20.9
9 Leukemia	20.45
10 Other lower respiratory infections	20.0
11 Multiple myeloma	14.06
12 Stomach cancer	13.61
13 Other non-Hodgkin lymphoma	13.31
14 Bladder cancer	10.95
15 Liver cancer	10.85
16 Cirrhosis and other chronic liver diseases	10.35
17 Prostate cancer	10.2
18 Ovarian cancer	9.59
19 Aortic aneurysm	8.1
20 Hypertensive heart disease	7.89
21 Esophageal cancer	
24 Parkinson's disease	
25 Adverse effects of medical treatment	
28 Cervical cancer	

Leading causes of death before and after garbage code redistribution: Republic of Moldova - 2015.

Causes are connected by arrows before and after redistribution. Infectious diseases are shown in red, non-communicable causes in blue, and injuries in green.

In addition to garbage redistribution, the diagram also reflects the deaths moved during misassignment correction for Alzheimer's disease and other dementias.

Leading causes Before Redistribution	Deaths
1 Ischemic heart disease	14280.0
2 Cirrhosis and other chronic liver diseases	2965.0
3 Hypertensive heart disease	1911.0
4 Ischemic stroke	1826.0
5 Intracerebral hemorrhage	1176.0
6 Tracheal, bronchus, and lung cancer	961.0
7 Colon and rectum cancer	829.0
8 Chronic obstructive pulmonary disease	748.0
9 Self-harm by other specified means	537.0
10 Breast cancer	536.0
11 Alcohol use disorders	480.0
12 Stomach cancer	470.0
13 Pancreatic cancer	350.0
14 Drug-susceptible tuberculosis	273.0
15 Pulmonary aspiration and foreign body in airway	236.0
16 Environmental heat and cold exposure	232.0
17 Pancreatitis	230.0
18 Poisoning by carbon monoxide	225.0
19 Prostate cancer	213.0
20 Peptic ulcer disease	212.0
26 Falls	
32 Pneumococcal pneumonia	
33 Other lower respiratory infections	
113 Alzheimer's disease and other dementias	

Leading causes After Redistribution	Deaths
1 Ischemic heart disease	14167.77
2 Ischemic stroke	3510.38
3 Cirrhosis and other chronic liver diseases	3035.12
4 Hypertensive heart disease	1882.96
5 Intracerebral hemorrhage	1792.17
6 Tracheal, bronchus, and lung cancer	1016.99
7 Colon and rectum cancer	980.98
8 Alzheimer's disease and other dementias	783.27
9 Chronic obstructive pulmonary disease	753.38
10 Breast cancer	600.44
11 Self-harm by other specified means	590.63
12 Stomach cancer	568.49
13 Other lower respiratory infections	496.21
14 Alcohol use disorders	494.57
15 Pneumococcal pneumonia	433.03
16 Pancreatic cancer	421.15
17 Drug-susceptible tuberculosis	281.27
18 Prostate cancer	261.42
19 Environmental heat and cold exposure	257.57
20 Falls	257.28
22 Pulmonary aspiration and foreign body in airway	
23 Poisoning by carbon monoxide	
25 Pancreatitis	
26 Peptic ulcer disease	

Leading causes of death before and after garbage code redistribution: Maldives - 2015.

Causes are connected by arrows before and after redistribution. Infectious diseases are shown in red, non-communicable causes in blue, and injuries in green.
In addition to garbage redistribution, the diagram also reflects the deaths moved during misassignment correction for Alzheimer's disease and other dementias.

Leading causes Before Redistribution	Deaths
1 Ischemic heart disease	91.0
2 Chronic obstructive pulmonary disease	45.0
3 Intracerebral hemorrhage	23.0
4 Other neonatal disorders	12.0
5 Liver cancer	11.0
6 Interstitial lung disease and pulmonary sarcoidosis	10.0
7 Chronic kidney disease due to hypertension	10.0
8 Neonatal encephalopathy due to birth asphyxia and trauma	9.0
9 Tracheal, bronchus, and lung cancer	8.0
10 Diabetes mellitus type 2	8.0
11 Congenital heart anomalies	7.0
12 Neonatal preterm birth	7.0
13 Endocrine, metabolic, blood, and immune disorders	7.0
14 Neonatal sepsis and other neonatal infections	7.0
15 Other endocrine, metabolic, blood, and immune disorders	6.0
16 Drug-susceptible tuberculosis	6.0
17 Chronic kidney disease	6.0
18 Asthma	6.0
19 Ovarian cancer	5.0
20 Drowning	5.0
21 Cirrhosis and other chronic liver diseases	
23 Ischemic stroke	
30 Falls	
32 Colon and rectum cancer	
48 Hypertensive heart disease	
51 Alzheimer's disease and other dementias	
52 Other lower respiratory infections	
72 Diarrheal diseases	

Leading causes After Redistribution	Deaths
1 Ischemic heart disease	186.53
2 Intracerebral hemorrhage	117.55
3 Ischemic stroke	94.09
4 Chronic obstructive pulmonary disease	87.84
5 Diabetes mellitus type 2	47.37
6 Chronic kidney disease due to hypertension	42.5
7 Other lower respiratory infections	32.77
8 Alzheimer's disease and other dementias	27.33
9 Hypertensive heart disease	26.4
10 Cirrhosis and other chronic liver diseases	24.15
11 Tracheal, bronchus, and lung cancer	20.07
12 Drug-susceptible tuberculosis	15.8
13 Diarrheal diseases	14.36
14 Asthma	13.88
15 Liver cancer	13.31
16 Falls	13.23
17 Other neonatal disorders	12.8
18 Colon and rectum cancer	12.3
19 Interstitial lung disease and pulmonary sarcoidosis	11.71
20 Neonatal encephalopathy due to birth asphyxia and trauma	11.5
22 Congenital heart anomalies	
26 Neonatal sepsis and other neonatal infections	
27 Chronic kidney disease	
28 Drowning	
29 Neonatal preterm birth	
30 Other endocrine, metabolic, blood, and immune disorders	
33 Endocrine, metabolic, blood, and immune disorders	
37 Ovarian cancer	

Leading causes of death before and after garbage code redistribution: Mexico - 2015.

Causes are connected by arrows before and after redistribution. Infectious diseases are shown in red, non-communicable causes in blue, and injuries in green.
In addition to garbage redistribution, the diagram also reflects the deaths moved during misassignment correction for Alzheimer's disease and other dementias.

Leading causes Before Redistribution	Deaths	Leading causes After Redistribution	Deaths
1 Ischemic heart disease	87915.0	1 Ischemic heart disease	97782.13
2 Diabetes mellitus type 2	40898.0	2 Diabetes mellitus type 2	62989.84
3 Cirrhosis and other chronic liver diseases	28803.0	3 Chronic kidney disease due to diabetes mellitus type 2	40056.72
4 Chronic kidney disease due to diabetes mellitus type 2	26240.0	4 Cirrhosis and other chronic liver diseases	36259.89
5 Chronic obstructive pulmonary disease	24412.0	5 Chronic obstructive pulmonary disease	28180.05
6 Physical violence by firearm	12760.0	6 Ischemic stroke	18529.56
7 Chronic kidney disease	9363.0	7 Other lower respiratory infections	15275.39
8 Chronic kidney disease due to hypertension	8484.0	8 Physical violence by firearm	15254.54
9 Hypertensive heart disease	7476.0	9 Alzheimer's disease and other dementias	13181.42
10 Intracerebral hemorrhage	7323.0	10 Chronic kidney disease due to hypertension	12246.27
11 Tracheal, bronchus, and lung cancer	7266.0	11 Intracerebral hemorrhage	12156.81
12 Protein-energy malnutrition	7014.0	12 Chronic kidney disease	10749.44
13 Prostate cancer	6463.0	13 Stomach cancer	9743.28
14 Breast cancer	6314.0	14 Pedestrian road injuries	9210.33
15 Neonatal preterm birth	6191.0	15 Hypertensive heart disease	8854.3
16 Stomach cancer	6182.0	16 Colon and rectum cancer	8405.45
17 Colon and rectum cancer	6069.0	17 Tracheal, bronchus, and lung cancer	8386.48
18 Urinary tract infections and interstitial nephritis	5931.0	18 Prostate cancer	7756.33
19 Self-harm by other specified means	5752.0	19 Breast cancer	7351.61
20 Congenital heart anomalies	4735.0	20 Falls	7281.71
21 Pedestrian road injuries		21 Self-harm by other specified means	
24 Ischemic stroke		22 Protein-energy malnutrition	
29 Alzheimer's disease and other dementias		23 Neonatal preterm birth	
35 Falls		24 Urinary tract infections and interstitial nephritis	
101 Other lower respiratory infections		27 Congenital heart anomalies	

Leading causes of death before and after garbage code redistribution: North Macedonia - 2013.

Causes are connected by arrows before and after redistribution. Infectious diseases are shown in red, non-communicable causes in blue, and injuries in green.
In addition to garbage redistribution, the diagram also reflects the deaths moved during misassignment correction for Alzheimer's disease and other dementias.

Leading causes Before Redistribution	Deaths	Leading causes After Redistribution	Deaths
1 Ischemic heart disease	1587.0	1 Ischemic stroke	3005.41
2 Tracheal, bronchus, and lung cancer	848.0	2 Ischemic heart disease	2926.33
3 Hypertensive heart disease	561.0	3 Other cardiomyopathy	2906.11
4 Diabetes mellitus type 2	508.0	4 Intracerebral hemorrhage	1084.13
5 Colon and rectum cancer	418.0	5 Tracheal, bronchus, and lung cancer	1017.4
6 Chronic obstructive pulmonary disease	349.0	6 Diabetes mellitus type 2	874.26
7 Stomach cancer	307.0	7 Hypertensive heart disease	856.24
8 Ischemic stroke	306.0	8 Colon and rectum cancer	533.9
9 Breast cancer	276.0	9 Chronic obstructive pulmonary disease	473.17
10 Intracerebral hemorrhage	244.0	10 Alzheimer's disease and other dementias	354.21
11 Pancreatic cancer	207.0	11 Stomach cancer	352.93
12 Prostate cancer	203.0	12 Breast cancer	318.62
13 Cirrhosis and other chronic liver diseases	189.0	13 Cirrhosis and other chronic liver diseases	259.25
14 Chronic kidney disease	183.0	14 Pancreatic cancer	247.89
15 Liver cancer	178.0	15 Prostate cancer	239.9
16 Brain and central nervous system cancer	169.0	16 Chronic kidney disease	237.59
17 Self-harm by other specified means	143.0	17 Brain and central nervous system cancer	195.85
18 Neonatal preterm birth	140.0	18 Liver cancer	190.85
19 Bladder cancer	112.0	19 Subarachnoid hemorrhage	184.4
20 Parkinson's disease	105.0	20 Self-harm by other specified means	170.25
26 Alzheimer's disease and other dementias		21 Neonatal preterm birth	
28 Subarachnoid hemorrhage		22 Parkinson's disease	
128 Other cardiomyopathy		23 Bladder cancer	

Leading causes of death before and after garbage code redistribution: Malta - 2015.

Causes are connected by arrows before and after redistribution. Infectious diseases are shown in red, non-communicable causes in blue, and injuries in green.
In addition to garbage redistribution, the diagram also reflects the deaths moved during misassignment correction for Alzheimer's disease and other dementias.

Leading causes Before Redistribution	Deaths
1 Ischemic heart disease	732.0
2 Tracheal, bronchus, and lung cancer	189.0
3 Alzheimer's disease and other dementias	148.0
4 Colon and rectum cancer	122.0
5 Chronic obstructive pulmonary disease	92.0
6 Pancreatic cancer	83.0
7 Breast cancer	81.0
8 Parkinson's disease	48.0
9 Interstitial lung disease and pulmonary sarcoidosis	47.0
10 Urinary tract infections and interstitial nephritis	41.0
11 Prostate cancer	39.0
12 Falls	35.0
13 Stomach cancer	34.0
14 Ovarian cancer	34.0
15 Hypertensive heart disease	33.0
16 Intracerebral hemorrhage	32.0
17 Self-harm by other specified means	31.0
18 Bladder cancer	26.0
19 Kidney cancer	26.0
20 Non-rheumatic calcific aortic valve disease	25.0
25 Ischemic stroke	
32 Diabetes mellitus type 2	
38 Atrial fibrillation and flutter	
112 Other lower respiratory infections	

Leading causes After Redistribution	Deaths
1 Ischemic heart disease	809.88
2 Alzheimer's disease and other dementias	239.81
3 Tracheal, bronchus, and lung cancer	199.06
4 Ischemic stroke	196.15
5 Colon and rectum cancer	138.35
6 Diabetes mellitus type 2	136.12
7 Other lower respiratory infections	107.6
8 Chronic obstructive pulmonary disease	101.06
9 Breast cancer	91.73
10 Pancreatic cancer	89.31
11 Intracerebral hemorrhage	64.19
12 Parkinson's disease	52.82
13 Atrial fibrillation and flutter	50.3
14 Interstitial lung disease and pulmonary sarcoidosis	48.63
15 Prostate cancer	44.05
16 Hypertensive heart disease	42.84
17 Stomach cancer	40.86
18 Falls	37.77
19 Ovarian cancer	37.41
20 Urinary tract infections and interstitial nephritis	36.25
22 Self-harm by other specified means	
24 Non-rheumatic calcific aortic valve disease	
25 Bladder cancer	
26 Kidney cancer	

Leading causes of death before and after garbage code redistribution: Montenegro - 2005.

Causes are connected by arrows before and after redistribution. Infectious diseases are shown in red, non-communicable causes in blue, and injuries in green.

In addition to garbage redistribution, the diagram also reflects the deaths moved during misassignment correction for Alzheimer's disease and other dementias.

Leading causes Before Redistribution	Deaths
1 Intracerebral hemorrhage	559.0
2 Ischemic heart disease	445.0
3 Tracheal, bronchus, and lung cancer	309.0
4 Chronic kidney disease	97.0
5 Breast cancer	75.0
6 Ischemic stroke	73.0
7 Self-harm by other specified means	71.0
8 Colon and rectum cancer	57.0
9 Liver cancer	54.0
10 Pancreatic cancer	52.0
11 Brain and central nervous system cancer	49.0
12 Self-harm by firearm	46.0
13 Prostate cancer	41.0
14 Stomach cancer	41.0
15 Aortic aneurysm	36.0
16 Atrial fibrillation and flutter	35.0
17 Larynx cancer	34.0
18 Vascular intestinal disorders	27.0
19 Physical violence by firearm	22.0
20 Other lower respiratory infections	21.0
31 Alzheimer's disease and other dementias	
86 Diabetes mellitus type 2	
90 Cirrhosis and other chronic liver diseases	
91 Other cardiomyopathy	
92 Alcoholic cardiomyopathy	

Leading causes After Redistribution	Deaths
1 Other cardiomyopathy	1064.94
2 Ischemic heart disease	719.77
3 Intracerebral hemorrhage	688.59
4 Tracheal, bronchus, and lung cancer	422.05
5 Ischemic stroke	144.2
6 Chronic kidney disease	125.91
7 Colon and rectum cancer	108.7
8 Diabetes mellitus type 2	108.67
9 Breast cancer	108.27
10 Alzheimer's disease and other dementias	92.93
11 Self-harm by other specified means	84.91
12 Pancreatic cancer	79.5
13 Alcoholic cardiomyopathy	74.23
14 Brain and central nervous system cancer	68.53
15 Stomach cancer	67.39
16 Liver cancer	66.9
17 Prostate cancer	63.64
18 Self-harm by firearm	52.87
19 Cirrhosis and other chronic liver diseases	49.5
20 Other lower respiratory infections	44.96
21 Aortic aneurysm	
22 Larynx cancer	
23 Atrial fibrillation and flutter	
25 Vascular intestinal disorders	
31 Physical violence by firearm	

Leading causes of death before and after garbage code redistribution: Mongolia - 2010.

Causes are connected by arrows before and after redistribution. Infectious diseases are shown in red, non-communicable causes in blue, and injuries in green.
In addition to garbage redistribution, the diagram also reflects the deaths moved during misassignment correction for Alzheimer's disease and other dementias.

Leading causes Before Redistribution	Deaths	Leading causes After Redistribution	Deaths
1 Ischemic heart disease	2710.0	1 Ischemic heart disease	3007.49
2 Intracerebral hemorrhage	2299.0	2 Intracerebral hemorrhage	2524.5
3 Liver cancer	1570.0	3 Liver cancer	1589.46
4 Cirrhosis and other chronic liver diseases	1014.0	4 Cirrhosis and other chronic liver diseases	1056.4
5 Stomach cancer	585.0	5 Stomach cancer	602.68
6 Alcohol use disorders	504.0	6 Alcohol use disorders	507.03
7 Self-harm by other specified means	433.0	7 Other lower respiratory infections	488.33
8 Tracheal, bronchus, and lung cancer	343.0	8 Self-harm by other specified means	457.1
9 Neonatal encephalopathy due to birth asphyxia and trauma	342.0	9 Neonatal encephalopathy due to birth asphyxia and trauma	367.72
10 Drug-susceptible tuberculosis	325.0	10 Tracheal, bronchus, and lung cancer	357.48
11 Esophageal cancer	282.0	11 Drug-susceptible tuberculosis	337.66
12 Environmental heat and cold exposure	229.0	12 Ischemic stroke	293.88
13 Motor vehicle road injuries	225.0	13 Esophageal cancer	288.62
14 Neonatal preterm birth	220.0	14 Neonatal preterm birth	235.46
15 Physical violence by other means	186.0	15 Motor vehicle road injuries	234.94
16 Falls	173.0	16 Environmental heat and cold exposure	234.11
17 Subarachnoid hemorrhage	148.0	17 Physical violence by other means	201.92
18 Chronic kidney disease	135.0	18 Chronic obstructive pulmonary disease	188.37
19 Drowning	130.0	19 Falls	187.05
20 Peptic ulcer disease	126.0	20 Subarachnoid hemorrhage	163.36
22 Chronic obstructive pulmonary disease		21 Chronic kidney disease	
26 Ischemic stroke		22 Drowning	
31 Other lower respiratory infections		24 Peptic ulcer disease	

Leading causes of death before and after garbage code redistribution: Northern Mariana Islands - 2015.

Causes are connected by arrows before and after redistribution. Infectious diseases are shown in red, non-communicable causes in blue, and injuries in green.

In addition to garbage redistribution, the diagram also reflects the deaths moved during misassignment correction for Alzheimer's disease and other dementias.

Leading causes Before Redistribution	Deaths
1 Ischemic heart disease	36.0
2 Self-harm by other specified means	10.0
3 Tracheal, bronchus, and lung cancer	9.0
4 Breast cancer	8.0
5 Colon and rectum cancer	8.0
6 Intracerebral hemorrhage	5.0
7 Chronic obstructive pulmonary disease	4.0
8 Chronic kidney disease due to hypertension	3.0
9 Chronic kidney disease	3.0
10 Neonatal preterm birth	3.0
11 Prostate cancer	3.0
12 Lip and oral cavity cancer	2.0
13 Liver cancer	2.0
14 Cervical cancer	2.0
15 Alcohol use disorders	2.0
16 Drowning	2.0
17 Pulmonary aspiration and foreign body in airway	2.0
18 Peptic ulcer disease	2.0
19 Alzheimer's disease and other dementias	2.0
20 Hypertensive heart disease	2.0
23 Stomach cancer	
34 Other lower respiratory infections	
43 Chronic kidney disease due to diabetes mellitus type 2	
45 Diabetes mellitus type 2	
46 Chronic kidney disease due to diabetes mellitus type 1	
47 Ischemic stroke	

Leading causes After Redistribution	Deaths
1 Ischemic heart disease	39.24
2 Diabetes mellitus type 2	17.24
3 Self-harm by other specified means	10.68
4 Tracheal, bronchus, and lung cancer	10.26
5 Intracerebral hemorrhage	9.73
6 Colon and rectum cancer	8.64
7 Breast cancer	8.6
8 Chronic kidney disease due to diabetes mellitus type 2	7.18
9 Chronic obstructive pulmonary disease	6.69
10 Other lower respiratory infections	6.53
11 Chronic kidney disease due to diabetes mellitus type 1	5.13
12 Ischemic stroke	3.81
13 Chronic kidney disease	3.21
14 Prostate cancer	3.18
15 Chronic kidney disease due to hypertension	3.08
16 Neonatal preterm birth	3.0
17 Cervical cancer	2.93
18 Alzheimer's disease and other dementias	2.66
19 Lip and oral cavity cancer	2.61
20 Stomach cancer	2.49
21 Hypertensive heart disease	
23 Peptic ulcer disease	
24 Drowning	
25 Pulmonary aspiration and foreign body in airway	
26 Liver cancer	
27 Alcohol use disorders	

Leading causes of death before and after garbage code redistribution: Mauritius - 2015.

Causes are connected by arrows before and after redistribution. Infectious diseases are shown in red, non-communicable causes in blue, and injuries in green.
In addition to garbage redistribution, the diagram also reflects the deaths moved during misassignment correction for Alzheimer's disease and other dementias.

Leading causes Before Redistribution	Deaths
1 Ischemic heart disease	1199.0
2 Hypertensive heart disease	299.0
3 Breast cancer	177.0
4 Tracheal, bronchus, and lung cancer	164.0
5 Intracerebral hemorrhage	142.0
6 Colon and rectum cancer	137.0
7 Cirrhosis and other chronic liver diseases	127.0
8 Chronic kidney disease due to hypertension	119.0
9 Self-harm by other specified means	103.0
10 Stomach cancer	89.0
11 Asthma	86.0
12 Chronic obstructive pulmonary disease	84.0
13 HIV/AIDS resulting in other diseases	82.0
14 Diabetes mellitus type 2	70.0
15 Prostate cancer	65.0
16 Falls	60.0
17 Interstitial lung disease and pulmonary sarcoidosis	57.0
18 Pancreatic cancer	53.0
19 Adverse effects of medical treatment	53.0
20 Alcohol use disorders	51.0
24 Chronic kidney disease due to diabetes mellitus type 2	
35 Alzheimer's disease and other dementias	
40 Ischemic stroke	
62 Other lower respiratory infections	
107 Chronic kidney disease due to diabetes mellitus type 1	

Leading causes After Redistribution	Deaths
1 Diabetes mellitus type 2	1648.61
2 Ischemic heart disease	1383.47
3 Chronic kidney disease due to diabetes mellitus type 2	682.95
4 Ischemic stroke	436.34
5 Hypertensive heart disease	368.39
6 Intracerebral hemorrhage	358.15
7 Alzheimer's disease and other dementias	264.2
8 Chronic kidney disease due to hypertension	234.97
9 Chronic obstructive pulmonary disease	205.76
10 Cirrhosis and other chronic liver diseases	200.74
11 Other lower respiratory infections	197.77
12 Breast cancer	195.74
13 Tracheal, bronchus, and lung cancer	184.49
14 Colon and rectum cancer	181.42
15 Stomach cancer	130.51
16 Interstitial lung disease and pulmonary sarcoidosis	117.47
17 Self-harm by other specified means	107.6
18 Asthma	92.31
19 HIV/AIDS resulting in other diseases	90.29
20 Chronic kidney disease due to diabetes mellitus type 1	85.3
21 Prostate cancer	
24 Falls	
25 Pancreatic cancer	
27 Alcohol use disorders	
29 Adverse effects of medical treatment	

Leading causes of death before and after garbage code redistribution: Malaysia - 2014.

Causes are connected by arrows before and after redistribution. Infectious diseases are shown in red, non-communicable causes in blue, and injuries in green.
In addition to garbage redistribution, the diagram also reflects the deaths moved during misassignment correction for Alzheimer's disease and other dementias.

Leading causes Before Redistribution	Deaths
1 Ischemic heart disease	10427.0
2 Intracerebral hemorrhage	2495.0
3 Tracheal, bronchus, and lung cancer	1740.0
4 Ischemic stroke	1215.0
5 Other urinary diseases	1194.0
6 Breast cancer	1061.0
7 Cirrhosis and other chronic liver diseases	975.0
8 Colon and rectum cancer	922.0
9 Liver cancer	909.0
10 Chronic obstructive pulmonary disease	884.0
11 Chronic kidney disease	789.0
12 Drug-susceptible tuberculosis	735.0
13 Leukemia	565.0
14 Decubitus ulcer	503.0
15 Chronic kidney disease due to glomerulonephritis	491.0
16 Neonatal preterm birth	489.0
17 HIV/AIDS resulting in other diseases	436.0
18 Drowning	410.0
19 Congenital heart anomalies	390.0
20 Stomach cancer	350.0
75 Other lower respiratory infections	
104 Self-harm by other specified means	
108 Alzheimer's disease and other dementias	
124 Motorcyclist road injuries	
147 Diabetes mellitus type 2	
154 Pedestrian road injuries	
163 Motor vehicle road injuries	

Leading causes After Redistribution	Deaths
1 Ischemic heart disease	12434.4
2 Other lower respiratory infections	9196.77
3 Intracerebral hemorrhage	4865.89
4 Ischemic stroke	3017.59
5 Cirrhosis and other chronic liver diseases	2328.11
6 Tracheal, bronchus, and lung cancer	2291.78
7 Motor vehicle road injuries	2229.25
8 Chronic obstructive pulmonary disease	1830.37
9 Breast cancer	1463.06
10 Colon and rectum cancer	1377.52
11 Drug-susceptible tuberculosis	1304.55
12 Chronic kidney disease	1285.46
13 Other urinary diseases	1197.38
14 Alzheimer's disease and other dementias	1188.62
15 Diabetes mellitus type 2	1173.62
16 Motorcyclist road injuries	1111.19
17 Pedestrian road injuries	1081.71
18 Liver cancer	1078.08
19 Self-harm by other specified means	851.71
20 Chronic kidney disease due to glomerulonephritis	796.62
22 HIV/AIDS resulting in other diseases	
24 Leukemia	
25 Stomach cancer	
27 Drowning	
31 Neonatal preterm birth	
32 Decubitus ulcer	
33 Congenital heart anomalies	

Leading causes of death before and after garbage code redistribution: Nicaragua - 2015.

Causes are connected by arrows before and after redistribution. Infectious diseases are shown in red, non-communicable causes in blue, and injuries in green.
In addition to garbage redistribution, the diagram also reflects the deaths moved during misassignment correction for Alzheimer's disease and other dementias.

Leading causes Before Redistribution	Deaths
1 Ischemic heart disease	3117.0
2 Chronic kidney disease	1251.0
3 Diabetes mellitus type 2	1044.0
4 Cirrhosis and other chronic liver diseases	932.0
5 Chronic obstructive pulmonary disease	728.0
6 Neonatal preterm birth	518.0
7 Chronic kidney disease due to diabetes mellitus type 2	495.0
8 Hypertensive heart disease	404.0
9 Alcohol use disorders	310.0
10 Self-harm by other specified means	292.0
11 Stomach cancer	280.0
12 Intracerebral hemorrhage	267.0
13 Neonatal sepsis and other neonatal infections	263.0
14 Cervical cancer	242.0
15 Prostate cancer	222.0
16 Congenital heart anomalies	214.0
17 Colon and rectum cancer	204.0
18 Neonatal encephalopathy due to birth asphyxia and trauma	202.0
19 Breast cancer	201.0
20 Chronic kidney disease due to hypertension	200.0
59 Ischemic stroke	
64 Alzheimer's disease and other dementias	
100 Other lower respiratory infections	

Leading causes After Redistribution	Deaths
1 Ischemic heart disease	3565.61
2 Diabetes mellitus type 2	1340.81
3 Chronic kidney disease	1329.26
4 Cirrhosis and other chronic liver diseases	1320.85
5 Chronic obstructive pulmonary disease	801.21
6 Other lower respiratory infections	696.72
7 Chronic kidney disease due to diabetes mellitus type 2	656.02
8 Ischemic stroke	625.82
9 Intracerebral hemorrhage	550.96
10 Neonatal preterm birth	542.26
11 Alzheimer's disease and other dementias	503.09
12 Hypertensive heart disease	481.42
13 Stomach cancer	408.4
14 Alcohol use disorders	324.51
15 Self-harm by other specified means	322.93
16 Chronic kidney disease due to hypertension	315.01
17 Cervical cancer	291.69
18 Neonatal sepsis and other neonatal infections	284.84
19 Colon and rectum cancer	277.17
20 Prostate cancer	272.45
22 Breast cancer	
23 Congenital heart anomalies	
28 Neonatal encephalopathy due to birth asphyxia and trauma	

Leading causes of death before and after garbage code redistribution: Netherlands - 2015.

Causes are connected by arrows before and after redistribution. Infectious diseases are shown in red, non-communicable causes in blue, and injuries in green.
In addition to garbage redistribution, the diagram also reflects the deaths moved during misassignment correction for Alzheimer's disease and other dementias.

Leading causes Before Redistribution	Deaths
1 Alzheimer's disease and other dementias	14324.0
2 Tracheal, bronchus, and lung cancer	10507.0
3 Ischemic heart disease	8981.0
4 Chronic obstructive pulmonary disease	6892.0
5 Colon and rectum cancer	5231.0
6 Breast cancer	3297.0
7 Falls	2839.0
8 Pancreatic cancer	2696.0
9 Prostate cancer	2640.0
10 Self-harm by other specified means	1822.0
11 Esophageal cancer	1813.0
12 Intracerebral hemorrhage	1763.0
13 Ischemic stroke	1728.0
14 Atrial fibrillation and flutter	1593.0
15 Parkinson's disease	1556.0
16 Stomach cancer	1305.0
17 Bladder cancer	1222.0
18 Urinary tract infections and interstitial nephritis	1135.0
19 Chronic kidney disease	1111.0
20 Non-rheumatic calcific aortic valve disease	1108.0
34 Diabetes mellitus type 2	
52 Influenza	

Leading causes After Redistribution	Deaths
1 Ischemic heart disease	14227.5
2 Tracheal, bronchus, and lung cancer	12283.93
3 Alzheimer's disease and other dementias	10733.39
4 Chronic obstructive pulmonary disease	9558.02
5 Ischemic stroke	8132.41
6 Colon and rectum cancer	7024.37
7 Falls	4216.56
8 Breast cancer	4021.72
9 Pancreatic cancer	3469.12
10 Prostate cancer	3330.95
11 Intracerebral hemorrhage	3060.82
12 Diabetes mellitus type 2	2760.83
13 Esophageal cancer	2317.42
14 Influenza	2263.61
15 Stomach cancer	2257.34
16 Non-rheumatic calcific aortic valve disease	2184.15
17 Atrial fibrillation and flutter	2064.99
18 Self-harm by other specified means	2046.31
19 Chronic kidney disease	1849.53
20 Parkinson's disease	1728.36
21 Urinary tract infections and interstitial nephritis	
22 Bladder cancer	

Leading causes of death before and after garbage code redistribution: Norway - 2015.

Causes are connected by arrows before and after redistribution. Infectious diseases are shown in red, non-communicable causes in blue, and injuries in green.
In addition to garbage redistribution, the diagram also reflects the deaths moved during misassignment correction for Alzheimer’s disease and other dementias.

Leading causes Before Redistribution	Deaths
1 Ischemic heart disease	4213.0
2 Alzheimer's disease and other dementias	3291.0
3 Tracheal, bronchus, and lung cancer	2197.0
4 Chronic obstructive pulmonary disease	2109.0
5 Colon and rectum cancer	1606.0
6 Prostate cancer	1046.0
7 Falls	815.0
8 Pancreatic cancer	761.0
9 Atrial fibrillation and flutter	719.0
10 Breast cancer	592.0
11 Intracerebral hemorrhage	545.0
12 Self-harm by other specified means	504.0
13 Ischemic stroke	503.0
14 Non-rheumatic calcific aortic valve disease	486.0
15 Aortic aneurysm	419.0
16 Parkinson's disease	410.0
17 Malignant skin melanoma	331.0
18 Stomach cancer	327.0
19 Urinary tract infections and interstitial nephritis	321.0
20 Brain and central nervous system cancer	307.0
34 Diabetes mellitus type 2	
101 Other lower respiratory infections	

Leading causes After Redistribution	Deaths
1 Ischemic heart disease	5687.62
2 Alzheimer's disease and other dementias	3080.51
3 Chronic obstructive pulmonary disease	2587.29
4 Tracheal, bronchus, and lung cancer	2472.18
5 Ischemic stroke	2092.75
6 Colon and rectum cancer	1979.27
7 Falls	1260.86
8 Prostate cancer	1234.33
9 Other lower respiratory infections	982.58
10 Pancreatic cancer	892.59
11 Intracerebral hemorrhage	784.89
12 Atrial fibrillation and flutter	760.01
13 Non-rheumatic calcific aortic valve disease	749.43
14 Breast cancer	701.54
15 Self-harm by other specified means	566.71
16 Diabetes mellitus type 2	538.66
17 Parkinson's disease	536.82
18 Aortic aneurysm	501.79
19 Stomach cancer	442.07
20 Urinary tract infections and interstitial nephritis	423.6
24 Malignant skin melanoma	
26 Brain and central nervous system cancer	

Leading causes of death before and after garbage code redistribution: New Zealand - 2015.

Causes are connected by arrows before and after redistribution. Infectious diseases are shown in red, non-communicable causes in blue, and injuries in green.

In addition to garbage redistribution, the diagram also reflects the deaths moved during misassignment correction for Alzheimer's disease and other dementias.

Leading causes Before Redistribution	Deaths	Leading causes After Redistribution	Deaths
1 Ischemic heart disease	5017.0	1 Ischemic heart disease	5399.01
2 Alzheimer's disease and other dementias	2502.0	2 Alzheimer's disease and other dementias	2145.07
3 Tracheal, bronchus, and lung cancer	1809.0	3 Tracheal, bronchus, and lung cancer	1918.25
4 Chronic obstructive pulmonary disease	1608.0	4 Chronic obstructive pulmonary disease	1802.56
5 Colon and rectum cancer	1271.0	5 Ischemic stroke	1670.34
6 Breast cancer	674.0	6 Colon and rectum cancer	1533.14
7 Prostate cancer	647.0	7 Prostate cancer	745.15
8 Falls	545.0	8 Breast cancer	740.54
9 Pancreatic cancer	506.0	9 Falls	609.39
10 Self-harm by other specified means	482.0	10 Atrial fibrillation and flutter	603.24
11 Ischemic stroke	473.0	11 Intracerebral hemorrhage	571.37
12 Atrial fibrillation and flutter	441.0	12 Pancreatic cancer	550.19
13 Diabetes mellitus type 2	418.0	13 Self-harm by other specified means	509.9
14 Intracerebral hemorrhage	386.0	14 Diabetes mellitus type 2	468.91
15 Malignant skin melanoma	378.0	15 Non-rheumatic calcific aortic valve disease	406.08
16 Non-rheumatic calcific aortic valve disease	368.0	16 Malignant skin melanoma	401.51
17 Chronic kidney disease due to diabetes mellitus type 2	359.0	17 Chronic kidney disease due to diabetes mellitus type 2	388.26
18 Aortic aneurysm	339.0	18 Parkinson's disease	381.87
19 Liver cancer	270.0	19 Aortic aneurysm	355.74
20 Stomach cancer	261.0	20 Influenza	351.87
21 Parkinson's disease		21 Stomach cancer	
65 Influenza		24 Liver cancer	

Leading causes of death before and after garbage code redistribution: Oman - 2014.

Causes are connected by arrows before and after redistribution. Infectious diseases are shown in red, non-communicable causes in blue, and injuries in green.
In addition to garbage redistribution, the diagram also reflects the deaths moved during misassignment correction for Alzheimer’s disease and other dementias.

Leading causes Before Redistribution	Deaths
1 Ischemic heart disease	353.0
2 Diabetes mellitus type 2	198.0
3 Neonatal preterm birth	189.0
4 Motor vehicle road injuries	130.0
5 Ischemic stroke	77.0
6 Chronic kidney disease	74.0
7 Intracerebral hemorrhage	68.0
8 Cirrhosis and other chronic liver diseases	59.0
9 Urinary tract infections and interstitial nephritis	52.0
10 Pedestrian road injuries	50.0
11 Other endocrine, metabolic, blood, and immune disorders	50.0
12 Hypertensive heart disease	47.0
13 Decubitus ulcer	46.0
14 Other congenital birth defects	45.0
15 Congenital heart anomalies	40.0
16 Atrial fibrillation and flutter	37.0
17 Stomach cancer	32.0
18 Other neonatal disorders	32.0
19 Tracheal, bronchus, and lung cancer	30.0
20 Breast cancer	30.0
21 Colon and rectum cancer	
23 Other cardiovascular and circulatory diseases (internal)	
27 Chronic obstructive pulmonary disease	
28 Falls	
43 Alzheimer's disease and other dementias	
44 Other lower respiratory infections	
90 Chronic kidney disease due to hypertension	
134 Physical violence by other means	

Leading causes After Redistribution	Deaths
1 Ischemic heart disease	1326.42
2 Diabetes mellitus type 2	534.49
3 Motor vehicle road injuries	459.57
4 Ischemic stroke	448.45
5 Other lower respiratory infections	282.73
6 Intracerebral hemorrhage	282.33
7 Neonatal preterm birth	246.74
8 Cirrhosis and other chronic liver diseases	218.97
9 Pedestrian road injuries	208.46
10 Hypertensive heart disease	200.48
11 Chronic kidney disease	182.98
12 Chronic obstructive pulmonary disease	152.03
13 Falls	100.8
14 Stomach cancer	99.72
15 Tracheal, bronchus, and lung cancer	94.16
16 Alzheimer's disease and other dementias	90.44
17 Chronic kidney disease due to hypertension	87.42
18 Colon and rectum cancer	86.81
19 Other cardiovascular and circulatory diseases (internal)	86.25
20 Physical violence by other means	81.95
21 Other endocrine, metabolic, blood, and immune disorders	
22 Congenital heart anomalies	
23 Urinary tract infections and interstitial nephritis	
25 Breast cancer	
29 Other congenital birth defects	
31 Decubitus ulcer	
34 Other neonatal disorders	
42 Atrial fibrillation and flutter	

Leading causes of death before and after garbage code redistribution: Panama - 2015.

Causes are connected by arrows before and after redistribution. Infectious diseases are shown in red, non-communicable causes in blue, and injuries in green.

In addition to garbage redistribution, the diagram also reflects the deaths moved during misassignment correction for Alzheimer's disease and other dementias.

Leading causes Before Redistribution	Deaths
1 Ischemic heart disease	1491.0
2 HIV/AIDS resulting in other diseases	466.0
3 Chronic obstructive pulmonary disease	458.0
4 Chronic kidney disease	393.0
5 Diabetes mellitus type 2	344.0
6 Physical violence by firearm	340.0
7 Prostate cancer	335.0
8 Cirrhosis and other chronic liver diseases	326.0
9 Stomach cancer	324.0
10 Tracheal, bronchus, and lung cancer	318.0
11 Hypertensive heart disease	291.0
12 Colon and rectum cancer	278.0
13 Alzheimer's disease and other dementias	258.0
14 Drug-susceptible tuberculosis	218.0
15 Breast cancer	217.0
16 Neonatal preterm birth	197.0
17 Congenital heart anomalies	190.0
18 Pedestrian road injuries	189.0
19 Motor vehicle road injuries	178.0
20 Cervical cancer	161.0
21 Intracerebral hemorrhage	
25 Chronic kidney disease due to hypertension	
32 Chronic kidney disease due to diabetes mellitus type 2	
40 Ischemic stroke	
61 Other cardiomyopathy	
68 Other lower respiratory infections	

Leading causes After Redistribution	Deaths
1 Ischemic heart disease	1983.15
2 Diabetes mellitus type 2	1138.47
3 Ischemic stroke	980.7
4 Alzheimer's disease and other dementias	697.89
5 Intracerebral hemorrhage	575.16
6 Chronic obstructive pulmonary disease	569.17
7 HIV/AIDS resulting in other diseases	519.54
8 Chronic kidney disease	485.57
9 Other lower respiratory infections	481.52
10 Stomach cancer	464.93
11 Prostate cancer	429.34
12 Cirrhosis and other chronic liver diseases	400.27
13 Tracheal, bronchus, and lung cancer	394.0
14 Hypertensive heart disease	393.83
15 Physical violence by firearm	389.12
16 Colon and rectum cancer	386.42
17 Chronic kidney disease due to diabetes mellitus type 2	293.45
18 Breast cancer	276.97
19 Chronic kidney disease due to hypertension	245.93
20 Other cardiomyopathy	244.67
21 Congenital heart anomalies	
22 Pedestrian road injuries	
23 Drug-susceptible tuberculosis	
24 Neonatal preterm birth	
25 Motor vehicle road injuries	
26 Cervical cancer	

Leading causes of death before and after garbage code redistribution: Peru - 2015.

Causes are connected by arrows before and after redistribution. Infectious diseases are shown in red, non-communicable causes in blue, and injuries in green.

In addition to garbage redistribution, the diagram also reflects the deaths moved during misassignment correction for Alzheimer's disease and other dementias.

Leading causes Before Redistribution	Deaths
1 Interstitial lung disease and pulmonary sarcoidosis	3954.0
2 Cirrhosis and other chronic liver diseases	3592.0
3 Ischemic heart disease	3358.0
4 Stomach cancer	2888.0
5 Chronic kidney disease	2236.0
6 Chronic obstructive pulmonary disease	1748.0
7 Tracheal, bronchus, and lung cancer	1741.0
8 Urinary tract infections and interstitial nephritis	1532.0
9 Prostate cancer	1498.0
10 Pulmonary aspiration and foreign body in airway	1490.0
11 Drug-susceptible tuberculosis	1375.0
12 Colon and rectum cancer	1306.0
13 Hypertensive heart disease	1027.0
14 Breast cancer	1023.0
15 Cervical cancer	990.0
16 Protein-energy malnutrition	900.0
17 Diabetes mellitus type 2	857.0
18 Pancreatic cancer	826.0
19 Brain and central nervous system cancer	822.0
20 Intracerebral hemorrhage	801.0
30 Ischemic stroke	
32 Pedestrian road injuries	
45 Other lower respiratory infections	
55 Falls	
116 Alzheimer's disease and other dementias	

Leading causes After Redistribution	Deaths
1 Other lower respiratory infections	11438.89
2 Ischemic heart disease	5890.58
3 Drug-susceptible tuberculosis	4383.7
4 Cirrhosis and other chronic liver diseases	4196.43
5 Interstitial lung disease and pulmonary sarcoidosis	4103.5
6 Diabetes mellitus type 2	3968.37
7 Stomach cancer	3827.34
8 Chronic kidney disease	2879.2
9 Alzheimer's disease and other dementias	2710.03
10 Chronic obstructive pulmonary disease	2384.67
11 Ischemic stroke	2150.42
12 Intracerebral hemorrhage	2064.21
13 Pedestrian road injuries	2053.26
14 Hypertensive heart disease	1903.54
15 Tracheal, bronchus, and lung cancer	1903.46
16 Prostate cancer	1754.34
17 Colon and rectum cancer	1685.43
18 Pulmonary aspiration and foreign body in airway	1678.95
19 Urinary tract infections and interstitial nephritis	1504.62
20 Falls	1401.88
21 Cervical cancer	
24 Breast cancer	
25 Pancreatic cancer	
30 Brain and central nervous system cancer	
35 Protein-energy malnutrition	

Leading causes of death before and after garbage code redistribution: Philippines - 2015.

Causes are connected by arrows before and after redistribution. Infectious diseases are shown in red, non-communicable causes in blue, and injuries in green.
In addition to garbage redistribution, the diagram also reflects the deaths moved during misassignment correction for Alzheimer's disease and other dementias.

Leading causes Before Redistribution	Deaths	Leading causes After Redistribution	Deaths
1 Ischemic heart disease	68472.0	1 Ischemic heart disease	83202.39
2 Drug-susceptible tuberculosis	26081.0	2 Intracerebral hemorrhage	43730.22
3 Hypertensive heart disease	23805.0	3 Other lower respiratory infections	36781.82
4 Intracerebral hemorrhage	18711.0	4 Hypertensive heart disease	28378.92
5 Chronic obstructive pulmonary disease	14436.0	5 Drug-susceptible tuberculosis	28298.03
6 Diabetes mellitus type 2	13575.0	6 Diabetes mellitus type 2	26055.88
7 Tracheal, bronchus, and lung cancer	9701.0	7 Ischemic stroke	22780.65
8 Asthma	8906.0	8 Chronic obstructive pulmonary disease	17683.7
9 Cirrhosis and other chronic liver diseases	8369.0	9 Cirrhosis and other chronic liver diseases	12002.36
10 Breast cancer	7902.0	10 Tracheal, bronchus, and lung cancer	11296.05
11 Physical violence by firearm	7173.0	11 Chronic kidney disease due to diabetes mellitus type 2	11243.77
12 Chronic kidney disease	7101.0	12 Asthma	9687.75
13 Colon and rectum cancer	6833.0	13 Colon and rectum cancer	9554.2
14 Ischemic stroke	6443.0	14 Breast cancer	9336.36
15 Peptic ulcer disease	5704.0	15 Alzheimer's disease and other dementias	8789.11
16 Chronic kidney disease due to diabetes mellitus type 2	4130.0	16 Chronic kidney disease	8495.79
17 Diarrheal diseases	3929.82	17 Physical violence by firearm	7682.41
18 Chronic kidney disease due to hypertension	3911.0	18 Chronic kidney disease due to hypertension	7458.89
19 Neonatal preterm birth	3853.0	19 Pneumococcal pneumonia	6962.71
20 Physical violence by sharp object	3540.0	20 Peptic ulcer disease	6193.9
74 Alzheimer's disease and other dementias		21 Diarrheal diseases	
118 Other lower respiratory infections		25 Neonatal preterm birth	
198 Pneumococcal pneumonia		32 Physical violence by sharp object	

Leading causes of death before and after garbage code redistribution: Palau - 2013.

Causes are connected by arrows before and after redistribution. Infectious diseases are shown in red, non-communicable causes in blue, and injuries in green.
In addition to garbage redistribution, the diagram also reflects the deaths moved during misassignment correction for Alzheimer’s disease and other dementias.

Leading causes Before Redistribution	Deaths
1 Ischemic heart disease	20.0
2 Chronic kidney disease	9.0
3 Cirrhosis and other chronic liver diseases	6.0
4 Tracheal, bronchus, and lung cancer	5.0
5 Chronic obstructive pulmonary disease	3.0
6 Intracerebral hemorrhage	3.0
7 Ischemic stroke	3.0
8 Breast cancer	2.0
9 Lip and oral cavity cancer	2.0
10 Cervical cancer	2.0
11 Neonatal preterm birth	2.0
12 Other lower respiratory infections	2.0
13 Neonatal encephalopathy due to birth asphyxia and trauma	1.0
14 Prostate cancer	1.0
15 Alzheimer's disease and other dementias	1.0
16 Asthma	1.0
17 Stomach cancer	1.0
18 Liver cancer	1.0
19 Pancreatic cancer	1.0
20 Other malignant neoplasms (internal)	1.0
27 HIV/AIDS resulting in other diseases	
28 Diabetes mellitus type 2	
29 Falls	
30 Diarrheal diseases	
31 Influenza	

Leading causes After Redistribution	Deaths
1 Ischemic heart disease	35.54
2 Other lower respiratory infections	16.84
3 Intracerebral hemorrhage	12.64
4 Chronic kidney disease	10.72
5 Cirrhosis and other chronic liver diseases	10.54
6 Chronic obstructive pulmonary disease	9.37
7 Ischemic stroke	7.59
8 Tracheal, bronchus, and lung cancer	7.46
9 Diabetes mellitus type 2	5.07
10 Diarrheal diseases	3.52
11 Breast cancer	3.26
12 Asthma	3.21
13 HIV/AIDS resulting in other diseases	3.17
14 Influenza	3.1
15 Stomach cancer	3.06
16 Lip and oral cavity cancer	2.65
17 Alzheimer's disease and other dementias	2.64
18 Cervical cancer	2.56
19 Falls	2.22
20 Neonatal preterm birth	2.19
24 Prostate cancer	
27 Other malignant neoplasms (internal)	
29 Pancreatic cancer	
30 Liver cancer	
37 Neonatal encephalopathy due to birth asphyxia and trauma	

Leading causes of death before and after garbage code redistribution: Papua New Guinea - 1980.

Causes are connected by arrows before and after redistribution. Infectious diseases are shown in red, non-communicable causes in blue, and injuries in green.
In addition to garbage redistribution, the diagram also reflects the deaths moved during misassignment correction for Alzheimer's disease and other dementias.

Leading causes Before Redistribution	Deaths
1 Lower respiratory infections	898.0
2 Malaria	251.0
3 Meningitis	237.9
4 Neonatal disorders	220.0
5 Tuberculosis	211.0
6 Diarrheal diseases	161.66
7 Chronic respiratory diseases	79.0
8 Other intestinal infectious diseases	74.0
9 Protein-energy malnutrition	62.0
10 Cirrhosis and other chronic liver diseases	44.67
11 Pertussis	40.0
12 Other direct maternal disorders	39.0
13 Road injuries	30.0
14 Other cardiovascular and circulatory diseases (internal)	28.95
15 Chronic kidney disease	25.98
16 Paralytic ileus and intestinal obstruction	23.0
17 Pancreatitis	21.41
18 Measles	21.0
19 Other hemoglobinopathies and hemolytic anemias	19.58
20 Maternal disorders	13.0

27 Congenital heart anomalies
45 Ischemic heart disease
48 Rheumatic heart disease
122 Chronic obstructive pulmonary disease

Leading causes After Redistribution	Deaths
1 Lower respiratory infections	953.66
2 Malaria	266.51
3 Meningitis	250.28
4 Tuberculosis	242.36
5 Neonatal disorders	231.74
6 Diarrheal diseases	181.28
7 Chronic respiratory diseases	86.76
8 Other intestinal infectious diseases	76.71
9 Protein-energy malnutrition	76.28
10 Ischemic heart disease	75.31
11 Cirrhosis and other chronic liver diseases	69.25
12 Other cardiovascular and circulatory diseases (internal)	50.08
13 Congenital heart anomalies	47.02
14 Other direct maternal disorders	44.76
15 Pertussis	42.79
16 Chronic kidney disease	39.24
17 Road injuries	34.99
18 Rheumatic heart disease	30.18
19 Chronic obstructive pulmonary disease	29.16
20 Paralytic ileus and intestinal obstruction	28.82

21 Pancreatitis
23 Measles
25 Other hemoglobinopathies and hemolytic anemias
33 Maternal disorders

Leading causes of death before and after garbage code redistribution: Poland - 2015.

Causes are connected by arrows before and after redistribution. Infectious diseases are shown in red, non-communicable causes in blue, and injuries in green.
In addition to garbage redistribution, the diagram also reflects the deaths moved during misassignment correction for Alzheimer’s disease and other dementias.

Leading causes Before Redistribution	Deaths
1 Ischemic heart disease	39359.0
2 Tracheal, bronchus, and lung cancer	24990.0
3 Ischemic stroke	15244.0
4 Colon and rectum cancer	12525.0
5 Chronic obstructive pulmonary disease	6724.0
6 Breast cancer	6397.0
7 Diabetes mellitus type 2	5915.0
8 Intracerebral hemorrhage	5587.0
9 Cirrhosis and other chronic liver diseases	5511.0
10 Stomach cancer	5376.0
11 Self-harm by other specified means	5001.0
12 Pancreatic cancer	4928.0
13 Prostate cancer	4889.0
14 Falls	4507.0
15 Bladder cancer	3905.0
16 Alcohol use disorders	3689.0
17 Hypertensive heart disease	3293.0
18 Brain and central nervous system cancer	3054.0
19 Kidney cancer	2889.0
20 Ovarian cancer	2814.0
23 Alzheimer's disease and other dementias	
26 Atrial fibrillation and flutter	
38 Other lower respiratory infections	
56 Other cardiomyopathy	

Leading causes After Redistribution	Deaths
1 Ischemic heart disease	84636.49
2 Ischemic stroke	34819.53
3 Tracheal, bronchus, and lung cancer	30398.12
4 Colon and rectum cancer	17029.79
5 Alzheimer's disease and other dementias	14944.74
6 Other lower respiratory infections	11404.2
7 Other cardiomyopathy	10260.22
8 Chronic obstructive pulmonary disease	9520.85
9 Intracerebral hemorrhage	9026.14
10 Hypertensive heart disease	8967.34
11 Diabetes mellitus type 2	8660.02
12 Cirrhosis and other chronic liver diseases	8144.25
13 Breast cancer	7761.7
14 Stomach cancer	6692.48
15 Self-harm by other specified means	6392.85
16 Pancreatic cancer	6360.23
17 Prostate cancer	6066.37
18 Falls	5418.63
19 Atrial fibrillation and flutter	4983.77
20 Bladder cancer	4602.74
21 Alcohol use disorders	
22 Brain and central nervous system cancer	
23 Kidney cancer	
24 Ovarian cancer	

Leading causes of death before and after garbage code redistribution: Puerto Rico - 2015.

Causes are connected by arrows before and after redistribution. Infectious diseases are shown in red, non-communicable causes in blue, and injuries in green.
In addition to garbage redistribution, the diagram also reflects the deaths moved during misassignment correction for Alzheimer's disease and other dementias.

Leading causes Before Redistribution	Deaths
1 Ischemic heart disease	3215.0
2 Alzheimer's disease and other dementias	2453.0
3 Chronic obstructive pulmonary disease	909.0
4 Colon and rectum cancer	658.0
5 Tracheal, bronchus, and lung cancer	600.0
6 Cirrhosis and other chronic liver diseases	586.0
7 Hypertensive heart disease	554.0
8 Diabetes mellitus type 2	544.0
9 Physical violence by firearm	539.0
10 Prostate cancer	483.0
11 Breast cancer	443.0
12 Pancreatic cancer	321.0
13 Chronic kidney disease	307.0
14 Intracerebral hemorrhage	292.0
15 Falls	254.0
16 Stomach cancer	219.0
17 Endocrine, metabolic, blood, and immune disorders	217.0
18 Urinary tract infections and interstitial nephritis	215.0
19 HIV/AIDS resulting in other diseases	196.0
20 Self-harm by other specified means	188.0
22 Chronic kidney disease due to diabetes mellitus type 2	
23 Chronic kidney disease due to hypertension	
36 Ischemic stroke	
85 Other lower respiratory infections	

Leading causes After Redistribution	Deaths
1 Ischemic heart disease	3975.0
2 Diabetes mellitus type 2	2748.18
3 Alzheimer's disease and other dementias	1299.04
4 Chronic obstructive pulmonary disease	1228.29
5 Colon and rectum cancer	955.27
6 Ischemic stroke	861.09
7 Cirrhosis and other chronic liver diseases	837.89
8 Hypertensive heart disease	759.89
9 Tracheal, bronchus, and lung cancer	753.87
10 Chronic kidney disease due to diabetes mellitus type 2	698.8
11 Prostate cancer	678.59
12 Physical violence by firearm	639.85
13 Breast cancer	596.41
14 Intracerebral hemorrhage	572.33
15 Other lower respiratory infections	532.28
16 Chronic kidney disease	470.6
17 Chronic kidney disease due to hypertension	434.09
18 Pancreatic cancer	426.76
19 Falls	380.47
20 Urinary tract infections and interstitial nephritis	365.9
21 Stomach cancer	
25 Endocrine, metabolic, blood, and immune disorders	
26 HIV/AIDS resulting in other diseases	
28 Self-harm by other specified means	

Leading causes of death before and after garbage code redistribution: Portugal - 2015.

Causes are connected by arrows before and after redistribution. Infectious diseases are shown in red, non-communicable causes in blue, and injuries in green.
In addition to garbage redistribution, the diagram also reflects the deaths moved during misassignment correction for Alzheimer's disease and other dementias.

Leading causes Before Redistribution	Deaths
1 Ischemic heart disease	7328.0
2 Alzheimer's disease and other dementias	4932.0
3 Tracheal, bronchus, and lung cancer	4025.0
4 Colon and rectum cancer	3849.0
5 Chronic obstructive pulmonary disease	2535.0
6 Stomach cancer	2340.0
7 Prostate cancer	1723.0
8 Breast cancer	1709.0
9 Diabetes mellitus type 2	1644.0
10 Urinary tract infections and interstitial nephritis	1618.0
11 Intracerebral hemorrhage	1586.0
12 Pancreatic cancer	1427.0
13 Cirrhosis and other chronic liver diseases	1215.0
14 Hypertensive heart disease	1124.0
15 Atrial fibrillation and flutter	1022.0
16 Bladder cancer	1012.0
17 Self-harm by other specified means	968.0
18 Liver cancer	924.0
19 Brain and central nervous system cancer	887.0
20 Chronic kidney disease	820.0
21 Parkinson's disease	
22 Falls	
25 Other lower respiratory infections	
30 Chronic kidney disease due to diabetes mellitus type 2	
31 Ischemic stroke	

Leading causes After Redistribution	Deaths
1 Ischemic heart disease	10352.09
2 Ischemic stroke	9563.82
3 Alzheimer's disease and other dementias	7676.03
4 Chronic obstructive pulmonary disease	5446.13
5 Other lower respiratory infections	5334.53
6 Colon and rectum cancer	4764.13
7 Tracheal, bronchus, and lung cancer	4656.53
8 Diabetes mellitus type 2	3929.29
9 Intracerebral hemorrhage	3634.38
10 Stomach cancer	2994.94
11 Prostate cancer	2118.7
12 Breast cancer	2023.31
13 Hypertensive heart disease	1950.74
14 Urinary tract infections and interstitial nephritis	1830.73
15 Pancreatic cancer	1726.02
16 Cirrhosis and other chronic liver diseases	1654.27
17 Falls	1335.23
18 Chronic kidney disease due to diabetes mellitus type 2	1314.78
19 Self-harm by other specified means	1307.7
20 Parkinson's disease	1302.5
22 Bladder cancer	
23 Chronic kidney disease	
25 Liver cancer	
27 Atrial fibrillation and flutter	
28 Brain and central nervous system cancer	

Leading causes of death before and after garbage code redistribution: Paraguay - 2015.

Causes are connected by arrows before and after redistribution. Infectious diseases are shown in red, non-communicable causes in blue, and injuries in green.

In addition to garbage redistribution, the diagram also reflects the deaths moved during misassignment correction for Alzheimer's disease and other dementias.

Leading causes Before Redistribution	Deaths
1 Ischemic heart disease	2493.0
2 Diabetes mellitus type 2	1371.0
3 Motorcyclist road injuries	579.0
4 Chronic obstructive pulmonary disease	570.0
5 Tracheal, bronchus, and lung cancer	527.0
6 Hypertensive heart disease	500.0
7 Cirrhosis and other chronic liver diseases	454.0
8 Intracerebral hemorrhage	449.0
9 Chronic kidney disease	435.0
10 Neonatal preterm birth	389.0
11 Colon and rectum cancer	357.0
12 Physical violence by firearm	356.0
13 Prostate cancer	355.0
14 Breast cancer	347.0
15 Alcohol use disorders	340.0
16 Self-harm by other specified means	318.0
17 Cervical cancer	295.0
18 Protein-energy malnutrition	292.0
19 Pedestrian road injuries	273.0
20 Stomach cancer	256.0
54 Alzheimer's disease and other dementias	
60 Other lower respiratory infections	
112 Ischemic stroke	

Leading causes After Redistribution	Deaths
1 Ischemic heart disease	3357.0
2 Diabetes mellitus type 2	2537.68
3 Ischemic stroke	1340.84
4 Alzheimer's disease and other dementias	1103.53
5 Intracerebral hemorrhage	972.28
6 Chronic obstructive pulmonary disease	900.93
7 Other lower respiratory infections	759.95
8 Hypertensive heart disease	733.98
9 Tracheal, bronchus, and lung cancer	682.17
10 Motorcyclist road injuries	634.44
11 Cirrhosis and other chronic liver diseases	604.53
12 Chronic kidney disease	579.18
13 Colon and rectum cancer	469.42
14 Prostate cancer	453.52
15 Cervical cancer	412.52
16 Neonatal preterm birth	412.49
17 Pedestrian road injuries	411.71
18 Breast cancer	410.73
19 Physical violence by firearm	398.69
20 Alcohol use disorders	372.66
21 Stomach cancer	
22 Self-harm by other specified means	
27 Protein-energy malnutrition	

Leading causes of death before and after garbage code redistribution: Palestine - 2015.

Causes are connected by arrows before and after redistribution. Infectious diseases are shown in red, non-communicable causes in blue, and injuries in green.
In addition to garbage redistribution, the diagram also reflects the deaths moved during misassignment correction for Alzheimer's disease and other dementias.

Leading causes Before Redistribution	Deaths	Leading causes After Redistribution	Deaths
1 Ischemic heart disease	1283.0	1 Ischemic heart disease	2356.58
2 Diabetes mellitus type 2	788.0	2 Ischemic stroke	1253.73
3 Ischemic stroke	739.0	3 Diabetes mellitus type 2	1038.11
4 Hypertensive heart disease	364.0	4 Hypertensive heart disease	621.75
5 Neonatal preterm birth	358.0	5 Other lower respiratory infections	458.21
6 Tracheal, bronchus, and lung cancer	297.0	6 Neonatal preterm birth	428.08
7 Other neonatal disorders	258.0	7 Tracheal, bronchus, and lung cancer	367.91
8 Other lower respiratory infections	257.0	8 Chronic kidney disease	361.6
9 Colon and rectum cancer	229.0	9 Intracerebral hemorrhage	349.63
10 Chronic kidney disease	225.0	10 Other neonatal disorders	299.22
11 Breast cancer	196.0	11 Colon and rectum cancer	294.0
12 Congenital heart anomalies	195.0	12 Cirrhosis and other chronic liver diseases	264.6
13 Brain and central nervous system cancer	155.0	13 Congenital heart anomalies	261.35
14 Intracerebral hemorrhage	135.0	14 Alzheimer's disease and other dementias	254.03
15 Physical violence by firearm	120.0	15 Breast cancer	241.17
16 Neonatal encephalopathy due to birth asphyxia and trauma	112.0	16 Chronic obstructive pulmonary disease	209.65
17 Cirrhosis and other chronic liver diseases	108.0	17 Brain and central nervous system cancer	180.6
18 Chronic obstructive pulmonary disease	107.0	18 Falls	147.11
19 Leukemia	100.0	19 Motor vehicle road injuries	146.36
20 Falls	96.0	20 Neonatal encephalopathy due to birth asphyxia and trauma	141.81
22 Motor vehicle road injuries		22 Physical violence by firearm	
54 Alzheimer's disease and other dementias		23 Leukemia	

Leading causes of death before and after garbage code redistribution: Qatar - 2015.

Causes are connected by arrows before and after redistribution. Infectious diseases are shown in red, non-communicable causes in blue, and injuries in green.
In addition to garbage redistribution, the diagram also reflects the deaths moved during misassignment correction for Alzheimer's disease and other dementias.

Leading causes Before Redistribution	Deaths
1 Ischemic heart disease	117.0
2 Self-harm by other specified means	66.0
3 Neonatal preterm birth	64.0
4 Other lower respiratory infections	48.0
5 Breast cancer	41.0
6 Liver cancer	37.0
7 Tracheal, bronchus, and lung cancer	35.0
8 Chronic kidney disease	35.0
9 Falls	33.0
10 Cirrhosis and other chronic liver diseases	33.0
11 Congenital heart anomalies	31.0
12 Other congenital birth defects	28.0
13 Colon and rectum cancer	27.0
14 Intracerebral hemorrhage	21.0
15 Other exposure to mechanical forces	20.0
16 Stomach cancer	16.0
17 Electrocution	15.0
18 Drowning	15.0
19 Leukemia	13.0
20 Brain and central nervous system cancer	13.0

44 Chronic obstructive pulmonary disease
62 Motor vehicle road injuries
66 Ischemic stroke
91 Pedestrian road injuries
92 Diabetes mellitus type 2

Leading causes After Redistribution	Deaths
1 Ischemic heart disease	282.52
2 Motor vehicle road injuries	205.26
3 Diabetes mellitus type 2	148.94
4 Self-harm by other specified means	92.67
5 Other lower respiratory infections	82.66
6 Pedestrian road injuries	73.35
7 Neonatal preterm birth	71.11
8 Intracerebral hemorrhage	64.94
9 Cirrhosis and other chronic liver diseases	62.42
10 Tracheal, bronchus, and lung cancer	54.62
11 Chronic kidney disease	51.66
12 Falls	50.76
13 Liver cancer	50.52
14 Breast cancer	49.26
15 Chronic obstructive pulmonary disease	48.68
16 Ischemic stroke	45.81
17 Colon and rectum cancer	39.23
18 Congenital heart anomalies	36.16
19 Other congenital birth defects	30.93
20 Other exposure to mechanical forces	30.68

21 Stomach cancer
24 Electrocution
25 Leukemia
26 Brain and central nervous system cancer
27 Drowning

Leading causes of death before and after garbage code redistribution: Romania - 2015.

Causes are connected by arrows before and after redistribution. Infectious diseases are shown in red, non-communicable causes in blue, and injuries in green.
In addition to garbage redistribution, the diagram also reflects the deaths moved during misassignment correction for Alzheimer's disease and other dementias.

Leading causes Before Redistribution	Deaths
1 Ischemic heart disease	52709.0
2 Hypertensive heart disease	12415.0
3 Tracheal, bronchus, and lung cancer	10380.0
4 Cirrhosis and other chronic liver diseases	9120.0
5 Colon and rectum cancer	6172.0
6 Chronic obstructive pulmonary disease	6004.0
7 Ischemic stroke	4961.0
8 Intracerebral hemorrhage	4087.0
9 Breast cancer	3501.0
10 Stomach cancer	3357.0
11 Pancreatic cancer	2864.0
12 Prostate cancer	2208.0
13 Self-harm by other specified means	2205.0
14 Chronic kidney disease	2084.0
15 Alzheimer's disease and other dementias	2013.0
16 Brain and central nervous system cancer	1657.0
17 Cervical cancer	1644.0
18 Bladder cancer	1559.0
19 Falls	1291.0
20 Other malignant neoplasms (internal)	1267.0
21 Diabetes mellitus type 2	
22 Subarachnoid hemorrhage	
37 Parkinson's disease	
55 Other cardiomyopathy	
93 Other lower respiratory infections	

Leading causes After Redistribution	Deaths
1 Ischemic heart disease	64616.18
2 Ischemic stroke	39433.88
3 Hypertensive heart disease	16086.94
4 Intracerebral hemorrhage	13769.19
5 Tracheal, bronchus, and lung cancer	11180.38
6 Cirrhosis and other chronic liver diseases	9975.49
7 Alzheimer's disease and other dementias	7558.8
8 Colon and rectum cancer	7495.22
9 Chronic obstructive pulmonary disease	7080.88
10 Other cardiomyopathy	6318.13
11 Other lower respiratory infections	5355.83
12 Stomach cancer	4159.32
13 Breast cancer	3978.89
14 Pancreatic cancer	3404.0
15 Prostate cancer	2602.67
16 Self-harm by other specified means	2408.99
17 Diabetes mellitus type 2	2324.15
18 Subarachnoid hemorrhage	2312.14
19 Chronic kidney disease	2236.21
20 Parkinson's disease	2091.41
21 Cervical cancer	
22 Bladder cancer	
23 Brain and central nervous system cancer	
24 Falls	
25 Other malignant neoplasms (internal)	

Leading causes of death before and after garbage code redistribution: Russian Federation - 2015.

Causes are connected by arrows before and after redistribution. Infectious diseases are shown in red, non-communicable causes in blue, and injuries in green.
In addition to garbage redistribution, the diagram also reflects the deaths moved during misassignment correction for Alzheimer's disease and other dementias.

Leading causes Before Redistribution	Deaths
1 Ischemic heart disease	478029.22
2 Ischemic stroke	149322.51
3 Cirrhosis and other chronic liver diseases	50743.05
4 Tracheal, bronchus, and lung cancer	50539.07
5 Intracerebral hemorrhage	40500.02
6 Colon and rectum cancer	39609.7
7 Chronic obstructive pulmonary disease	33172.07
8 Stomach cancer	30015.35
9 Alzheimer's disease and other dementias	28338.88
10 Alcohol use disorders	25372.81
11 Self-harm by other specified means	23938.25
12 Breast cancer	22771.52
13 Alcoholic cardiomyopathy	19307.51
14 Diabetes mellitus type 2	19161.79
15 Pancreatic cancer	17172.09
16 Hypertensive heart disease	15187.23
17 Drug-susceptible tuberculosis	13069.51
18 Pancreatitis	12662.55
19 Motor vehicle road injuries	12128.78
20 Prostate cancer	11859.66
33 Other cardiomyopathy	
46 Physical violence by other means	
61 Other lower respiratory infections	

Leading causes After Redistribution	Deaths
1 Ischemic heart disease	560810.59
2 Ischemic stroke	251339.86
3 Intracerebral hemorrhage	61309.81
4 Tracheal, bronchus, and lung cancer	56329.7
5 Cirrhosis and other chronic liver diseases	56042.1
6 Colon and rectum cancer	46407.3
7 Alzheimer's disease and other dementias	44755.83
8 Self-harm by other specified means	43915.95
9 Chronic obstructive pulmonary disease	38576.03
10 Alcoholic cardiomyopathy	35306.19
11 Stomach cancer	35192.99
12 Other lower respiratory infections	31190.21
13 Alcohol use disorders	27339.45
14 Breast cancer	25874.62
15 Diabetes mellitus type 2	25061.5
16 Other cardiomyopathy	22963.85
17 Pancreatic cancer	19822.43
18 Hypertensive heart disease	18554.89
19 Physical violence by other means	15570.86
20 Drug-susceptible tuberculosis	14091.91
21 Pancreatitis	
22 Prostate cancer	
23 Motor vehicle road injuries	

Leading causes of death before and after garbage code redistribution: Singapore - 2015.

Causes are connected by arrows before and after redistribution. Infectious diseases are shown in red, non-communicable causes in blue, and injuries in green.

In addition to garbage redistribution, the diagram also reflects the deaths moved during misassignment correction for Alzheimer's disease and other dementias.

Leading causes Before Redistribution	Deaths
1 Ischemic heart disease	3312.0
2 Tracheal, bronchus, and lung cancer	1313.0
3 Colon and rectum cancer	881.0
4 Hypertensive heart disease	723.0
5 Liver cancer	519.0
6 Breast cancer	450.0
7 Urinary tract infections and interstitial nephritis	443.0
8 Chronic kidney disease	410.0
9 Self-harm by other specified means	408.0
10 Pancreatic cancer	378.0
11 Intracerebral hemorrhage	364.0
12 Chronic obstructive pulmonary disease	349.0
13 Stomach cancer	319.0
14 Prostate cancer	179.0
15 Falls	156.0
16 Cirrhosis and other chronic liver diseases	154.0
17 Ovarian cancer	133.0
18 Nasopharynx cancer	132.0
19 Kidney cancer	123.0
20 Esophageal cancer	111.0

21 Other non-Hodgkin lymphoma
32 Parkinson's disease
35 Ischemic stroke
97 Other lower respiratory infections
108 Alzheimer's disease and other dementias
113 Influenza
142 Pneumococcal pneumonia

Leading causes After Redistribution	Deaths
1 Ischemic heart disease	3334.06
2 Other lower respiratory infections	2388.26
3 Tracheal, bronchus, and lung cancer	1366.39
4 Colon and rectum cancer	960.96
5 Alzheimer's disease and other dementias	741.13
6 Hypertensive heart disease	733.88
7 Ischemic stroke	629.29
8 Liver cancer	541.45
9 Intracerebral hemorrhage	524.11
10 Breast cancer	471.67
11 Self-harm by other specified means	447.87
12 Pneumococcal pneumonia	432.45
13 Chronic obstructive pulmonary disease	425.91
14 Chronic kidney disease	412.01
15 Pancreatic cancer	399.43
16 Urinary tract infections and interstitial nephritis	383.9
17 Stomach cancer	362.53
18 Influenza	299.29
19 Parkinson's disease	206.23
20 Other non-Hodgkin lymphoma	198.86

21 Prostate cancer
23 Falls
24 Cirrhosis and other chronic liver diseases
25 Ovarian cancer
26 Nasopharynx cancer
27 Kidney cancer
28 Esophageal cancer

Leading causes of death before and after garbage code redistribution: El Salvador - 2015.

Causes are connected by arrows before and after redistribution. Infectious diseases are shown in red, non-communicable causes in blue, and injuries in green.

In addition to garbage redistribution, the diagram also reflects the deaths moved during misassignment correction for Alzheimer's disease and other dementias.

Leading causes Before Redistribution	Deaths
1 Physical violence by firearm	4601.0
2 Ischemic heart disease	2536.0
3 Chronic kidney disease	2003.0
4 Cirrhosis and other chronic liver diseases	817.0
5 Alcohol use disorders	634.0
6 Diabetes mellitus type 2	556.0
7 Physical violence by other means	441.0
8 Stomach cancer	437.0
9 Physical violence by sharp object	408.0
10 Self-harm by other specified means	337.0
11 Chronic obstructive pulmonary disease	335.0
12 Falls	239.0
13 Neonatal preterm birth	204.0
14 Colon and rectum cancer	203.0
15 Cervical cancer	194.0
16 HIV/AIDS resulting in other diseases	192.0
17 Tracheal, bronchus, and lung cancer	191.0
18 Prostate cancer	176.0
19 Breast cancer	170.0
20 Urinary tract infections and interstitial nephritis	166.0
21 Intracerebral hemorrhage	
42 Alzheimer's disease and other dementias	
51 Ischemic stroke	
53 Chronic kidney disease due to hypertension	
57 Other lower respiratory infections	
153 Pedestrian road injuries	

Leading causes After Redistribution	Deaths
1 Ischemic heart disease	5591.68
2 Physical violence by firearm	4931.88
3 Chronic kidney disease	3276.75
4 Diabetes mellitus type 2	2134.52
5 Other lower respiratory infections	1978.51
6 Cirrhosis and other chronic liver diseases	1591.66
7 Alzheimer's disease and other dementias	1133.66
8 Chronic obstructive pulmonary disease	1133.36
9 Ischemic stroke	1063.03
10 Stomach cancer	973.88
11 Intracerebral hemorrhage	735.86
12 Alcohol use disorders	717.78
13 Pedestrian road injuries	590.51
14 Physical violence by other means	584.7
15 Colon and rectum cancer	507.68
16 Chronic kidney disease due to hypertension	487.53
17 Physical violence by sharp object	446.41
18 Tracheal, bronchus, and lung cancer	441.96
19 Prostate cancer	436.59
20 Cervical cancer	418.1
21 Self-harm by other specified means	
23 Urinary tract infections and interstitial nephritis	
24 Falls	
25 Breast cancer	
27 HIV/AIDS resulting in other diseases	
33 Neonatal preterm birth	

Leading causes of death before and after garbage code redistribution: San Marino - 2015.

Causes are connected by arrows before and after redistribution. Infectious diseases are shown in red, non-communicable causes in blue, and injuries in green.
In addition to garbage redistribution, the diagram also reflects the deaths moved during misassignment correction for Alzheimer's disease and other dementias.

Leading causes Before Redistribution	Deaths	Leading causes After Redistribution	Deaths
1 Stroke	12.0	1 Ischemic heart disease	17.35
2 Alzheimer's disease and other dementias	10.21	2 Stroke	11.62
3 Tracheal, bronchus, and lung cancer	9.16	3 Diabetes mellitus type 2	10.45
4 Chronic respiratory diseases	9.0	4 Alzheimer's disease and other dementias	10.36
5 Colon and rectum cancer	8.1	5 Tracheal, bronchus, and lung cancer	10.3
6 Ischemic heart disease	8.0	6 Colon and rectum cancer	9.24
7 Self-harm	6.0	7 Chronic respiratory diseases	9.19
8 Falls	5.0	8 Falls	6.34
9 Bladder cancer	4.07	9 Self-harm	6.15
10 Pancreatic cancer	4.01	10 Leukemia	4.81
11 Leukemia	4.0	11 Pancreatic cancer	4.5
12 Stomach cancer	3.04	12 Bladder cancer	4.41
13 Breast cancer	3.02	13 Hypertensive heart disease	4.12
14 Cirrhosis and other chronic liver diseases	3.02	14 Other cardiomyopathy	4.09
15 Non-Hodgkin lymphoma	3.02	15 Stomach cancer	3.84
16 Transport injuries	3.0	16 Cirrhosis and other chronic liver diseases	3.44
17 Parkinson's disease	2.77	17 Breast cancer	3.4
18 Ovarian cancer	2.04	18 Non-Hodgkin lymphoma	3.32
19 Prostate cancer	2.02	19 Parkinson's disease	3.22
20 Liver cancer	2.0	20 Transport injuries	3.05
26 Hypertensive heart disease		21 Prostate cancer	
49 Other cardiomyopathy		23 Ovarian cancer	
177 Diabetes mellitus type 2		24 Liver cancer	

Leading causes of death before and after garbage code redistribution: Serbia - 2015.

Causes are connected by arrows before and after redistribution. Infectious diseases are shown in red, non-communicable causes in blue, and injuries in green.
In addition to garbage redistribution, the diagram also reflects the deaths moved during misassignment correction for Alzheimer's disease and other dementias.

Leading causes Before Redistribution	Deaths
1 Ischemic heart disease	9567.0
2 Ischemic stroke	8307.0
3 Tracheal, bronchus, and lung cancer	5346.0
4 Colon and rectum cancer	2603.0
5 Chronic obstructive pulmonary disease	2390.0
6 Chronic kidney disease	1791.0
7 Alzheimer's disease and other dementias	1732.0
8 Breast cancer	1729.0
9 Diabetes mellitus type 2	1585.0
10 Hypertensive heart disease	1362.0
11 Intracerebral hemorrhage	1330.0
12 Pancreatic cancer	1137.0
13 Prostate cancer	1074.0
14 Stomach cancer	912.0
15 Self-harm by other specified means	889.0
16 Cirrhosis and other chronic liver diseases	763.0
17 Bladder cancer	723.0
18 Brain and central nervous system cancer	691.0
19 Atrial fibrillation and flutter	575.0
20 Peptic ulcer disease	535.0
44 Other cardiomyopathy	
59 Other lower respiratory infections	
114 Alcoholic cardiomyopathy	

Leading causes After Redistribution	Deaths
1 Ischemic heart disease	17446.68
2 Ischemic stroke	13785.42
3 Other cardiomyopathy	13574.32
4 Tracheal, bronchus, and lung cancer	6382.12
5 Colon and rectum cancer	3499.89
6 Hypertensive heart disease	3201.39
7 Diabetes mellitus type 2	3164.38
8 Chronic obstructive pulmonary disease	3118.23
9 Intracerebral hemorrhage	2986.23
10 Chronic kidney disease	2158.54
11 Alzheimer's disease and other dementias	2151.7
12 Breast cancer	2076.53
13 Pancreatic cancer	1444.2
14 Other lower respiratory infections	1404.26
15 Prostate cancer	1372.41
16 Stomach cancer	1297.81
17 Cirrhosis and other chronic liver diseases	1110.12
18 Self-harm by other specified means	1091.31
19 Alcoholic cardiomyopathy	1004.48
20 Brain and central nervous system cancer	943.99
21 Bladder cancer	
24 Peptic ulcer disease	
26 Atrial fibrillation and flutter	

Leading causes of death before and after garbage code redistribution: Sao Tome and Principe - 1985.

Causes are connected by arrows before and after redistribution. Infectious diseases are shown in red, non-communicable causes in blue, and injuries in green.

In addition to garbage redistribution, the diagram also reflects the deaths moved during misassignment correction for Alzheimer's disease and other dementias.

Leading causes Before Redistribution	Deaths	Leading causes After Redistribution	Deaths
1 Malaria	350.0	1 Lower respiratory infections	403.3
2 Lower respiratory infections	281.0	2 Malaria	381.17
3 Diarrheal diseases	205.8	3 Diarrheal diseases	246.6
4 Measles	190.0	4 Measles	202.91
5 Neonatal disorders	104.0	5 Chronic respiratory diseases	159.48
6 Chronic respiratory diseases	85.0	6 Cirrhosis and other chronic liver diseases	153.49
7 Protein-energy malnutrition	79.0	7 Intracerebral hemorrhage	126.39
8 Cirrhosis and other chronic liver diseases	74.01	8 Neonatal disorders	125.03
9 Tetanus	47.0	9 Ischemic heart disease	124.14
10 Chronic kidney disease due to hypertension	36.74	10 Protein-energy malnutrition	106.97
11 Road injuries	34.0	11 Ischemic stroke	95.7
12 Tuberculosis	31.0	12 Tuberculosis	90.92
13 Ischemic stroke	27.98	13 Chronic kidney disease due to hypertension	80.27
14 Meningitis	25.0	14 Tetanus	54.99
15 Idiopathic epilepsy	25.0	15 Road injuries	42.14
16 Ischemic heart disease	23.0	16 Other malignant neoplasms (internal)	37.96
17 Intracerebral hemorrhage	21.96	17 Other cardiovascular and circulatory diseases (internal)	37.23
18 Other hemoglobinopathies and hemolytic anemias	19.94	18 Meningitis	31.04
19 Congenital birth defects	19.0	19 Stomach cancer	30.97
20 Falls	16.02	20 Falls	28.91
24 Stomach cancer		22 Idiopathic epilepsy	
27 Other malignant neoplasms (internal)		24 Congenital birth defects	
29 Other cardiovascular and circulatory diseases (internal)		25 Other hemoglobinopathies and hemolytic anemias	

Leading causes of death before and after garbage code redistribution: Suriname - 2014.

Causes are connected by arrows before and after redistribution. Infectious diseases are shown in red, non-communicable causes in blue, and injuries in green.

In addition to garbage redistribution, the diagram also reflects the deaths moved during misassignment correction for Alzheimer's disease and other dementias.

Leading causes Before Redistribution	Deaths
1 Ischemic heart disease	247.0
2 Self-harm by other specified means	121.0
3 HIV/AIDS resulting in other diseases	92.0
4 Intracerebral hemorrhage	81.0
5 Tracheal, bronchus, and lung cancer	64.0
6 Chronic kidney disease	52.0
7 Neonatal preterm birth	52.0
8 Cirrhosis and other chronic liver diseases	47.0
9 Motor vehicle road injuries	46.0
10 Prostate cancer	46.0
11 Breast cancer	38.0
12 Colon and rectum cancer	38.0
13 Urinary tract infections and interstitial nephritis	37.0
14 Cervical cancer	34.0
15 Hypertensive heart disease	33.0
16 Chronic obstructive pulmonary disease	33.0
17 Decubitus ulcer	27.0
18 Neonatal encephalopathy due to birth asphyxia and trauma	25.0
19 Drowning	24.0
20 Chronic kidney disease due to hypertension	21.0
46 Ischemic stroke	
73 Other lower respiratory infections	
91 Alzheimer's disease and other dementias	
137 Diabetes mellitus type 2	
138 Chronic kidney disease due to diabetes mellitus type 2	

Leading causes After Redistribution	Deaths
1 Ischemic heart disease	389.88
2 Intracerebral hemorrhage	225.63
3 Diabetes mellitus type 2	165.32
4 Ischemic stroke	152.19
5 Self-harm by other specified means	125.36
6 Other lower respiratory infections	104.36
7 HIV/AIDS resulting in other diseases	102.62
8 Tracheal, bronchus, and lung cancer	86.04
9 Prostate cancer	76.54
10 Chronic kidney disease	75.9
11 Colon and rectum cancer	73.87
12 Hypertensive heart disease	70.06
13 Cirrhosis and other chronic liver diseases	69.5
14 Chronic obstructive pulmonary disease	59.98
15 Alzheimer's disease and other dementias	56.09
16 Neonatal preterm birth	54.12
17 Motor vehicle road injuries	51.74
18 Urinary tract infections and interstitial nephritis	49.53
19 Breast cancer	48.1
20 Chronic kidney disease due to diabetes mellitus type 2	47.91
22 Cervical cancer	
23 Chronic kidney disease due to hypertension	
25 Drowning	
26 Neonatal encephalopathy due to birth asphyxia and trauma	
27 Decubitus ulcer	

Leading causes of death before and after garbage code redistribution: Slovakia - 2014.

Causes are connected by arrows before and after redistribution. Infectious diseases are shown in red, non-communicable causes in blue, and injuries in green.
In addition to garbage redistribution, the diagram also reflects the deaths moved during misassignment correction for Alzheimer's disease and other dementias.

Leading causes Before Redistribution	Deaths
1 Ischemic heart disease	13338.0
2 Ischemic stroke	2889.0
3 Tracheal, bronchus, and lung cancer	2177.0
4 Colon and rectum cancer	1991.0
5 Alzheimer's disease and other dementias	1380.0
6 Cirrhosis and other chronic liver diseases	1252.0
7 Hypertensive heart disease	1145.0
8 Breast cancer	910.0
9 Intracerebral hemorrhage	887.0
10 Pancreatic cancer	822.0
11 Chronic obstructive pulmonary disease	817.0
12 Prostate cancer	738.0
13 Falls	731.0
14 Stomach cancer	656.0
15 Diabetes mellitus type 2	537.0
16 Self-harm by other specified means	497.0
17 Kidney cancer	451.0
18 Brain and central nervous system cancer	390.0
19 Bladder cancer	374.0
20 Gallbladder and biliary tract cancer	350.0
35 Other lower respiratory infections	
135 Atrial fibrillation and flutter	

Leading causes After Redistribution	Deaths
1 Ischemic heart disease	14413.15
2 Ischemic stroke	3789.27
3 Tracheal, bronchus, and lung cancer	2389.36
4 Colon and rectum cancer	2307.74
5 Cirrhosis and other chronic liver diseases	1568.58
6 Alzheimer's disease and other dementias	1542.94
7 Other lower respiratory infections	1540.39
8 Hypertensive heart disease	1364.42
9 Intracerebral hemorrhage	1159.78
10 Falls	1031.56
11 Breast cancer	996.9
12 Pancreatic cancer	924.8
13 Chronic obstructive pulmonary disease	918.03
14 Prostate cancer	829.83
15 Diabetes mellitus type 2	781.19
16 Stomach cancer	775.49
17 Self-harm by other specified means	735.34
18 Kidney cancer	487.57
19 Brain and central nervous system cancer	418.81
20 Atrial fibrillation and flutter	415.65
21 Bladder cancer	
23 Gallbladder and biliary tract cancer	

Leading causes of death before and after garbage code redistribution: Slovenia - 2015.

Causes are connected by arrows before and after redistribution. Infectious diseases are shown in red, non-communicable causes in blue, and injuries in green.
In addition to garbage redistribution, the diagram also reflects the deaths moved during misassignment correction for Alzheimer's disease and other dementias.

Leading causes Before Redistribution	Deaths
1 Ischemic heart disease	2076.0
2 Tracheal, bronchus, and lung cancer	1192.0
3 Colon and rectum cancer	778.0
4 Ischemic stroke	666.0
5 Hypertensive heart disease	660.0
6 Falls	491.0
7 Breast cancer	430.0
8 Cirrhosis and other chronic liver diseases	422.0
9 Prostate cancer	404.0
10 Chronic obstructive pulmonary disease	387.0
11 Self-harm by other specified means	386.0
12 Pancreatic cancer	367.0
13 Non-rheumatic calcific aortic valve disease	350.0
14 Stomach cancer	339.0
15 Alcohol use disorders	281.0
16 Diabetes mellitus type 2	270.0
17 Intracerebral hemorrhage	270.0
18 Liver cancer	249.0
19 Leukemia	192.0
20 Brain and central nervous system cancer	186.0
24 Alzheimer's disease and other dementias	
26 Other lower respiratory infections	
139 Other cardiomyopathy	

Leading causes After Redistribution	Deaths
1 Ischemic heart disease	2630.78
2 Ischemic stroke	1423.88
3 Tracheal, bronchus, and lung cancer	1299.58
4 Alzheimer's disease and other dementias	1024.1
5 Hypertensive heart disease	1005.14
6 Colon and rectum cancer	875.31
7 Other cardiomyopathy	574.74
8 Non-rheumatic calcific aortic valve disease	522.66
9 Other lower respiratory infections	519.01
10 Falls	510.01
11 Breast cancer	464.37
12 Chronic obstructive pulmonary disease	460.2
13 Cirrhosis and other chronic liver diseases	453.46
14 Prostate cancer	447.42
15 Self-harm by other specified means	419.1
16 Intracerebral hemorrhage	399.47
17 Pancreatic cancer	396.29
18 Stomach cancer	368.02
19 Diabetes mellitus type 2	323.99
20 Alcohol use disorders	289.79
21 Liver cancer	
24 Leukemia	
25 Brain and central nervous system cancer	

Leading causes of death before and after garbage code redistribution: Sweden - 2015.

Causes are connected by arrows before and after redistribution. Infectious diseases are shown in red, non-communicable causes in blue, and injuries in green.
In addition to garbage redistribution, the diagram also reflects the deaths moved during misassignment correction for Alzheimer’s disease and other dementias.

Leading causes Before Redistribution	Deaths	Leading causes After Redistribution	Deaths
1 Ischemic heart disease	12121.0	1 Ischemic heart disease	16332.04
2 Alzheimer's disease and other dementias	8578.0	2 Alzheimer's disease and other dementias	6818.68
3 Tracheal, bronchus, and lung cancer	3716.0	3 Ischemic stroke	5211.0
4 Chronic obstructive pulmonary disease	2850.0	4 Tracheal, bronchus, and lung cancer	4272.82
5 Colon and rectum cancer	2847.0	5 Colon and rectum cancer	3672.95
6 Atrial fibrillation and flutter	2610.0	6 Chronic obstructive pulmonary disease	3553.55
7 Prostate cancer	2358.0	7 Prostate cancer	2861.27
8 Pancreatic cancer	1813.0	8 Atrial fibrillation and flutter	2603.06
9 Ischemic stroke	1457.0	9 Pancreatic cancer	2195.93
10 Breast cancer	1432.0	10 Falls	2120.41
11 Hypertensive heart disease	1298.0	11 Influenza	2049.78
12 Intracerebral hemorrhage	1156.0	12 Hypertensive heart disease	2002.74
13 Falls	1056.0	13 Intracerebral hemorrhage	1875.23
14 Self-harm by other specified means	1052.0	14 Diabetes mellitus type 2	1757.68
15 Aortic aneurysm	815.0	15 Breast cancer	1731.45
16 Non-rheumatic calcific aortic valve disease	779.0	16 Self-harm by other specified means	1301.27
17 Bladder cancer	694.0	17 Parkinson's disease	1215.98
18 Cirrhosis and other chronic liver diseases	686.0	18 Non-rheumatic calcific aortic valve disease	1199.74
19 Parkinson's disease	649.0	19 Aortic aneurysm	973.77
20 Kidney cancer	606.0	20 Cirrhosis and other chronic liver diseases	901.3
29 Diabetes mellitus type 2		23 Bladder cancer	
37 Influenza		25 Kidney cancer	

Leading causes of death before and after garbage code redistribution: Seychelles - 2015.

Causes are connected by arrows before and after redistribution. Infectious diseases are shown in red, non-communicable causes in blue, and injuries in green.
In addition to garbage redistribution, the diagram also reflects the deaths moved during misassignment correction for Alzheimer's disease and other dementias.

Leading causes Before Redistribution	Deaths
1 Lower respiratory infections	68.29
2 Stroke	58.0
3 Ischemic heart disease	31.0
4 Hypertensive heart disease	23.52
5 Cirrhosis and other chronic liver diseases	22.28
6 Prostate cancer	22.0
7 Colon and rectum cancer	19.04
8 Drowning	19.0
9 Chronic kidney disease	14.43
10 Transport injuries	13.0
11 Tracheal, bronchus, and lung cancer	11.05
12 Larynx cancer	10.0
13 Chronic respiratory diseases	10.0
14 Breast cancer	9.01
15 Neonatal disorders	8.0
16 Urinary tract infections and interstitial nephritis	7.26
17 Self-harm	7.0
18 Lip and oral cavity cancer	6.68
19 Chronic kidney disease due to hypertension	6.53
20 Liver cancer	6.0
22 Falls	
38 Alzheimer's disease and other dementias	
113 Ischemic stroke	
177 Diabetes mellitus type 2	
178 Intracerebral hemorrhage	

Leading causes After Redistribution	Deaths
1 Lower respiratory infections	72.58
2 Ischemic heart disease	64.56
3 Stroke	57.65
4 Hypertensive heart disease	31.8
5 Cirrhosis and other chronic liver diseases	26.64
6 Prostate cancer	23.76
7 Colon and rectum cancer	22.61
8 Ischemic stroke	20.68
9 Drowning	19.69
10 Chronic kidney disease	18.52
11 Intracerebral hemorrhage	16.24
12 Alzheimer's disease and other dementias	15.25
13 Diabetes mellitus type 2	14.33
14 Transport injuries	13.57
15 Tracheal, bronchus, and lung cancer	13.36
16 Chronic kidney disease due to hypertension	12.7
17 Breast cancer	10.96
18 Larynx cancer	10.34
19 Chronic respiratory diseases	10.21
20 Falls	8.31
21 Neonatal disorders	
22 Lip and oral cavity cancer	
23 Urinary tract infections and interstitial nephritis	
24 Self-harm	
27 Liver cancer	

Leading causes of death before and after garbage code redistribution: Syrian Arab Republic - 2009.

Causes are connected by arrows before and after redistribution. Infectious diseases are shown in red, non-communicable causes in blue, and injuries in green.

In addition to garbage redistribution, the diagram also reflects the deaths moved during misassignment correction for Alzheimer’s disease and other dementias.

Leading causes Before Redistribution	Deaths	Leading causes After Redistribution	Deaths
1 Ischemic heart disease	14216.0	1 Ischemic heart disease	20189.69
2 Lower respiratory infections	5865.31	2 Lower respiratory infections	6767.85
3 Stroke	4382.0	3 Stroke	4782.78
4 Chronic respiratory diseases	2632.0	4 Chronic respiratory diseases	2935.49
5 Neonatal disorders	2585.0	5 Neonatal disorders	2590.54
6 Congenital birth defects	1783.0	6 Congenital birth defects	1822.61
7 Transport injuries	1386.0	7 Transport injuries	1537.12
8 Tracheal, bronchus, and lung cancer	862.63	8 Other cardiomyopathy	1343.34
9 Chronic kidney disease	774.37	9 Chronic kidney disease	1304.03
10 Cirrhosis and other chronic liver diseases	647.68	10 Hypertensive heart disease	1181.58
11 Leukemia	544.61	11 Alzheimer's disease and other dementias	1174.16
12 Brain and central nervous system cancer	504.89	12 Tracheal, bronchus, and lung cancer	1051.49
13 Atrial fibrillation and flutter	470.06	13 Cirrhosis and other chronic liver diseases	1002.15
14 Colon and rectum cancer	440.05	14 Diabetes mellitus type 2	940.12
15 Stomach cancer	394.09	15 Leukemia	671.62
16 Interstitial lung disease and pulmonary sarcoidosis	378.95	16 Ischemic stroke	642.86
17 Liver cancer	361.78	17 Brain and central nervous system cancer	600.85
18 Breast cancer	302.49	18 Chronic obstructive pulmonary disease	598.9
19 Pancreatic cancer	287.64	19 Colon and rectum cancer	554.54
20 Urinary tract infections and interstitial nephritis	265.04	20 Interstitial lung disease and pulmonary sarcoidosis	541.11
22 Hypertensive heart disease		21 Stomach cancer	
29 Other cardiomyopathy		22 Atrial fibrillation and flutter	
34 Alzheimer's disease and other dementias		23 Liver cancer	
98 Ischemic stroke		25 Breast cancer	
164 Chronic obstructive pulmonary disease		27 Pancreatic cancer	
165 Diabetes mellitus type 2		31 Urinary tract infections and interstitial nephritis	

Leading causes of death before and after garbage code redistribution: Thailand - 2015.

Causes are connected by arrows before and after redistribution. Infectious diseases are shown in red, non-communicable causes in blue, and injuries in green.
In addition to garbage redistribution, the diagram also reflects the deaths moved during misassignment correction for Alzheimer's disease and other dementias.

Leading causes Before Redistribution	Deaths	Leading causes After Redistribution	Deaths
1 Ischemic heart disease	19417.0	1 Other lower respiratory infections	42732.48
2 Liver cancer	16375.0	2 Ischemic heart disease	33825.08
3 Tracheal, bronchus, and lung cancer	13073.0	3 Intracerebral hemorrhage	24500.54
4 Intracerebral hemorrhage	11644.0	4 Liver cancer	21213.79
5 Cirrhosis and other chronic liver diseases	10547.0	5 Diabetes mellitus type 2	20105.78
6 Ischemic stroke	8071.0	6 Ischemic stroke	20013.25
7 Chronic kidney disease	7529.0	7 Cirrhosis and other chronic liver diseases	19783.35
8 Alzheimer's disease and other dementias	6747.0	8 Tracheal, bronchus, and lung cancer	19654.55
9 Chronic obstructive pulmonary disease	6343.0	9 Alzheimer's disease and other dementias	16577.77
10 Drug-susceptible tuberculosis	6084.0	10 Chronic kidney disease	13888.16
11 Motorcyclist road injuries	5863.0	11 Chronic obstructive pulmonary disease	12861.35
12 HIV/AIDS resulting in other diseases	5456.0	12 Drug-susceptible tuberculosis	11081.48
13 Subarachnoid hemorrhage	4470.0	13 Colon and rectum cancer	8973.24
14 Colon and rectum cancer	4108.0	14 Motorcyclist road injuries	8872.87
15 Self-harm by other specified means	4039.0	15 HIV/AIDS resulting in other diseases	7710.16
16 Breast cancer	3766.0	16 Self-harm by other specified means	6692.29
17 Gallbladder and biliary tract cancer	3676.0	17 Subarachnoid hemorrhage	6364.95
18 Other urinary diseases	3340.0	18 Motor vehicle road injuries	6150.47
19 Drowning	3250.0	19 Breast cancer	6083.55
20 Diabetes mellitus type 2	2544.0	20 Diarrheal diseases	6050.27
26 Motor vehicle road injuries		22 Gallbladder and biliary tract cancer	
27 Diarrheal diseases		23 Other urinary diseases	
31 Other lower respiratory infections		25 Drowning	

Leading causes of death before and after garbage code redistribution: Tajikistan - 2005.

Causes are connected by arrows before and after redistribution. Infectious diseases are shown in red, non-communicable causes in blue, and injuries in green.

In addition to garbage redistribution, the diagram also reflects the deaths moved during misassignment correction for Alzheimer's disease and other dementias.

Leading causes Before Redistribution	Deaths
1 Ischemic heart disease	5706.0
2 Stroke	2645.0
3 Lower respiratory infections	1507.0
4 Cirrhosis and other chronic liver diseases	1109.0
5 Chronic respiratory diseases	818.0
6 Neonatal disorders	634.0
7 Tuberculosis	622.0
8 Other congenital birth defects	493.0
9 Diarrheal diseases	468.0
10 Stomach cancer	447.0
11 Road injuries	309.0
12 Esophageal cancer	297.0
13 Idiopathic epilepsy	282.0
14 Drowning	252.0
15 Rheumatic heart disease	203.0
16 Neoplasms	189.0
17 Self-harm	187.0
18 Tracheal, bronchus, and lung cancer	171.0
19 Meningitis	169.0
20 Urinary tract infections and interstitial nephritis	160.0
45 Alzheimer's disease and other dementias	
49 Diabetes mellitus type 2	

Leading causes After Redistribution	Deaths
1 Ischemic heart disease	8977.96
2 Stroke	5514.45
3 Lower respiratory infections	1720.26
4 Cirrhosis and other chronic liver diseases	1322.38
5 Chronic respiratory diseases	1242.18
6 Tuberculosis	673.85
7 Neonatal disorders	647.72
8 Stomach cancer	586.46
9 Diabetes mellitus type 2	562.37
10 Diarrheal diseases	509.66
11 Other congenital birth defects	508.01
12 Drowning	461.19
13 Road injuries	374.72
14 Esophageal cancer	340.62
15 Idiopathic epilepsy	317.55
16 Alzheimer's disease and other dementias	312.54
17 Tracheal, bronchus, and lung cancer	309.44
18 Rheumatic heart disease	253.17
19 Self-harm	219.02
20 Meningitis	218.78
22 Neoplasms	
25 Urinary tract infections and interstitial nephritis	

Leading causes of death before and after garbage code redistribution: Turkmenistan - 2015.

Causes are connected by arrows before and after redistribution. Infectious diseases are shown in red, non-communicable causes in blue, and injuries in green.
In addition to garbage redistribution, the diagram also reflects the deaths moved during misassignment correction for Alzheimer's disease and other dementias.

Leading causes Before Redistribution	Deaths	Leading causes After Redistribution	Deaths
1 Ischemic heart disease	7232.0	1 Ischemic heart disease	8952.73
2 Stroke	4093.0	2 Stroke	4614.51
3 Cirrhosis and other chronic liver diseases	2108.24	3 Cirrhosis and other chronic liver diseases	2539.76
4 Neonatal disorders	1555.0	4 Hypertensive heart disease	1828.27
5 Hypertensive heart disease	1543.88	5 Neonatal disorders	1581.35
6 Lower respiratory infections	908.26	6 Lower respiratory infections	1168.7
7 Congenital birth defects	794.0	7 Congenital birth defects	860.77
8 Tuberculosis	428.0	8 Diabetes mellitus type 2	646.99
9 Stomach cancer	288.29	9 Tuberculosis	561.54
10 Tracheal, bronchus, and lung cancer	263.44	10 Other lower respiratory infections	456.3
11 Esophageal cancer	248.05	11 Alcoholic cardiomyopathy	377.66
12 Breast cancer	244.08	12 Stomach cancer	350.28
13 Chronic kidney disease due to hypertension	242.97	13 Alzheimer's disease and other dementias	334.63
14 Upper respiratory infections	200.73	14 Tracheal, bronchus, and lung cancer	329.69
15 Chronic respiratory diseases	190.0	15 Upper respiratory infections	317.27
16 Alcoholic cardiomyopathy	183.26	16 Chronic kidney disease due to hypertension	304.84
17 Liver cancer	179.08	17 Breast cancer	290.22
18 Other malignant neoplasms (internal)	160.0	18 Esophageal cancer	288.57
19 Urinary tract infections and interstitial nephritis	153.49	19 Chronic respiratory diseases	229.57
20 Brain and central nervous system cancer	138.0	20 Self-harm	210.82
22 Self-harm		21 Other malignant neoplasms (internal)	
83 Alzheimer's disease and other dementias		22 Liver cancer	
144 Other lower respiratory infections		24 Urinary tract infections and interstitial nephritis	
176 Diabetes mellitus type 2		26 Brain and central nervous system cancer	

Leading causes of death before and after garbage code redistribution: Tonga - 2003.

Causes are connected by arrows before and after redistribution. Infectious diseases are shown in red, non-communicable causes in blue, and injuries in green.
In addition to garbage redistribution, the diagram also reflects the deaths moved during misassignment correction for Alzheimer's disease and other dementias.

Leading causes Before Redistribution	Deaths	Leading causes After Redistribution	Deaths
1 Endocrine, metabolic, blood, and immune disorders	132.0	1 Ischemic heart disease	211.45
2 Diabetes mellitus	113.0	2 Endocrine, metabolic, blood, and immune disorders	197.1
3 Ischemic heart disease	97.0	3 Diabetes mellitus	168.52
4 Stroke	64.0	4 Stroke	121.23
5 Chronic respiratory diseases	52.0	5 Chronic respiratory diseases	100.45
6 Lower respiratory infections	46.0	6 Lower respiratory infections	89.93
7 Digestive diseases	37.0	7 Digestive diseases	68.25
8 Tracheal, bronchus, and lung cancer	31.0	8 Tracheal, bronchus, and lung cancer	48.14
9 Injuries	25.0	9 Liver cancer	47.97
10 Liver cancer	23.0	10 Injuries	35.7
11 Transport injuries	22.0	11 Transport injuries	34.01
12 Cirrhosis and other chronic liver diseases	19.0	12 Breast cancer	30.75
13 Breast cancer	19.0	13 Stomach cancer	28.6
14 Meningitis	16.0	14 Cirrhosis and other chronic liver diseases	27.29
15 Neonatal disorders	15.0	15 Meningitis	24.99
16 Pulmonary aspiration and foreign body in airway	14.0	16 Pulmonary aspiration and foreign body in airway	22.92
17 Drowning	14.0	17 Neonatal disorders	22.59
18 Stomach cancer	13.0	18 Acute glomerulonephritis	21.17
19 Acute hepatitis	12.0	19 Prostate cancer	21.05
20 Neonatal preterm birth	10.0	20 Drowning	19.14
21 Prostate cancer		23 Acute hepatitis	
58 Acute glomerulonephritis		28 Neonatal preterm birth	

Leading causes of death before and after garbage code redistribution: Trinidad and Tobago - 2012.

Causes are connected by arrows before and after redistribution. Infectious diseases are shown in red, non-communicable causes in blue, and injuries in green.
In addition to garbage redistribution, the diagram also reflects the deaths moved during misassignment correction for Alzheimer’s disease and other dementias.

Leading causes Before Redistribution	Deaths
1 Ischemic heart disease	1357.0
2 Prostate cancer	290.0
3 Physical violence by firearm	276.0
4 HIV/AIDS resulting in other diseases	214.0
5 Breast cancer	176.0
6 Colon and rectum cancer	163.0
7 Chronic kidney disease due to hypertension	154.0
8 Self-harm by other specified means	148.0
9 Tracheal, bronchus, and lung cancer	141.0
10 Hypertensive heart disease	136.0
11 Chronic obstructive pulmonary disease	118.0
12 Alzheimer's disease and other dementias	104.0
13 Intracerebral hemorrhage	102.0
14 Cirrhosis and other chronic liver diseases	93.0
15 Chronic kidney disease	91.0
16 Motor vehicle road injuries	78.0
17 Cervical cancer	75.0
18 Ischemic stroke	74.0
19 Physical violence by sharp object	72.0
20 Other cardiovascular and circulatory diseases (internal)	67.0
33 Diabetes mellitus type 2	
82 Chronic kidney disease due to diabetes mellitus type 2	
148 Other lower respiratory infections	

Leading causes After Redistribution	Deaths
1 Ischemic heart disease	1528.67
2 Diabetes mellitus type 2	1283.2
3 Ischemic stroke	617.34
4 Intracerebral hemorrhage	341.17
5 Prostate cancer	330.03
6 Physical violence by firearm	283.32
7 Chronic kidney disease due to diabetes mellitus type 2	234.56
8 HIV/AIDS resulting in other diseases	225.68
9 Alzheimer's disease and other dementias	218.63
10 Breast cancer	201.68
11 Colon and rectum cancer	201.66
12 Chronic kidney disease due to hypertension	197.11
13 Hypertensive heart disease	172.09
14 Self-harm by other specified means	162.9
15 Tracheal, bronchus, and lung cancer	158.35
16 Chronic obstructive pulmonary disease	144.09
17 Cirrhosis and other chronic liver diseases	133.82
18 Other lower respiratory infections	126.09
19 Motor vehicle road injuries	122.15
20 Chronic kidney disease	104.95
21 Cervical cancer	
23 Other cardiovascular and circulatory diseases (internal)	
26 Physical violence by sharp object	

Leading causes of death before and after garbage code redistribution: Tunisia - 2013.

Causes are connected by arrows before and after redistribution. Infectious diseases are shown in red, non-communicable causes in blue, and injuries in green.

In addition to garbage redistribution, the diagram also reflects the deaths moved during misassignment correction for Alzheimer's disease and other dementias.

Leading causes Before Redistribution	Deaths	Leading causes After Redistribution	Deaths
1 Ischemic heart disease	1316.0	1 Ischemic heart disease	2999.6
2 Tracheal, bronchus, and lung cancer	822.0	2 Ischemic stroke	1904.47
3 Neonatal preterm birth	572.0	3 Diabetes mellitus type 2	1821.99
4 Ischemic stroke	489.0	4 Tracheal, bronchus, and lung cancer	1105.87
5 Intracerebral hemorrhage	405.0	5 Intracerebral hemorrhage	933.59
6 Chronic obstructive pulmonary disease	296.0	6 Neonatal preterm birth	823.81
7 Colon and rectum cancer	285.0	7 Alzheimer's disease and other dementias	723.46
8 Chronic kidney disease	269.0	8 Chronic obstructive pulmonary disease	555.33
9 Breast cancer	259.0	9 Colon and rectum cancer	481.06
10 Diabetes mellitus type 2	239.0	10 Chronic kidney disease	472.01
11 Prostate cancer	223.0	11 Cirrhosis and other chronic liver diseases	431.41
12 Cirrhosis and other chronic liver diseases	209.0	12 Other lower respiratory infections	397.18
13 Alzheimer's disease and other dementias	207.0	13 Breast cancer	395.08
14 Other congenital birth defects	185.0	14 Hypertensive heart disease	375.27
15 Congenital heart anomalies	162.0	15 Motor vehicle road injuries	359.78
16 Stomach cancer	152.0	16 Prostate cancer	350.88
17 Neonatal encephalopathy due to birth asphyxia and trauma	151.0	17 Stomach cancer	310.9
18 Pancreatic cancer	146.0	18 Falls	269.24
19 Asthma	144.0	19 Pancreatic cancer	226.34
20 Bladder cancer	135.0	20 Pedestrian road injuries	220.03
31 Hypertensive heart disease		21 Congenital heart anomalies	
35 Other lower respiratory infections		22 Other congenital birth defects	
82 Falls		23 Asthma	
151 Motor vehicle road injuries		24 Bladder cancer	
163 Pedestrian road injuries		25 Neonatal encephalopathy due to birth asphyxia and trauma	

Leading causes of death before and after garbage code redistribution: Turkey - 2015.

Causes are connected by arrows before and after redistribution. Infectious diseases are shown in red, non-communicable causes in blue, and injuries in green.
In addition to garbage redistribution, the diagram also reflects the deaths moved during misassignment correction for Alzheimer's disease and other dementias.

Leading causes Before Redistribution	Deaths
1 Ischemic heart disease	64668.0
2 Chronic obstructive pulmonary disease	24064.0
3 Tracheal, bronchus, and lung cancer	23150.0
4 Alzheimer's disease and other dementias	12817.0
5 Hypertensive heart disease	10042.0
6 Colon and rectum cancer	7020.0
7 Stomach cancer	6718.0
8 Chronic kidney disease	6701.0
9 Intracerebral hemorrhage	5385.0
10 Ischemic stroke	4810.0
11 Neonatal preterm birth	4627.0
12 Pancreatic cancer	4577.0
13 Falls	4122.0
14 Breast cancer	3989.0
15 Brain and central nervous system cancer	3615.0
16 Prostate cancer	3566.0
17 Diabetes mellitus type 2	3311.0
18 Cirrhosis and other chronic liver diseases	3180.0
19 Congenital heart anomalies	2449.0
20 Subarachnoid hemorrhage	2385.0

22 Chronic kidney disease due to hypertension
29 Other lower respiratory infections
39 Motor vehicle road injuries

Leading causes After Redistribution	Deaths
1 Ischemic heart disease	86978.19
2 Ischemic stroke	28138.78
3 Chronic obstructive pulmonary disease	27222.97
4 Tracheal, bronchus, and lung cancer	24530.98
5 Diabetes mellitus type 2	15815.61
6 Hypertensive heart disease	15659.56
7 Alzheimer's disease and other dementias	15598.33
8 Intracerebral hemorrhage	13337.34
9 Other lower respiratory infections	12528.09
10 Chronic kidney disease	9019.77
11 Colon and rectum cancer	8484.44
12 Stomach cancer	8312.74
13 Cirrhosis and other chronic liver diseases	5803.93
14 Pancreatic cancer	5150.71
15 Neonatal preterm birth	5016.21
16 Falls	4914.57
17 Breast cancer	4660.92
18 Chronic kidney disease due to hypertension	4450.69
19 Prostate cancer	4221.29
20 Motor vehicle road injuries	4065.38

21 Brain and central nervous system cancer
22 Congenital heart anomalies
24 Subarachnoid hemorrhage

Leading causes of death before and after garbage code redistribution: Taiwan (Province of China) - 2015.

Causes are connected by arrows before and after redistribution. Infectious diseases are shown in red, non-communicable causes in blue, and injuries in green.

In addition to garbage redistribution, the diagram also reflects the deaths moved during misassignment correction for Alzheimer's disease and other dementias.

Leading causes Before Redistribution	Deaths
1 Ischemic heart disease	9964.0
2 Tracheal, bronchus, and lung cancer	9452.0
3 Colon and rectum cancer	5742.0
4 Chronic obstructive pulmonary disease	5503.0
5 Cirrhosis and other chronic liver diseases	4713.0
6 Self-harm by other specified means	3613.0
7 Intracerebral hemorrhage	2948.0
8 Hypertensive heart disease	2607.0
9 Urinary tract infections and interstitial nephritis	2588.0
10 Stomach cancer	2343.0
11 Chronic kidney disease	2180.0
12 Breast cancer	2153.0
13 Pancreatic cancer	1952.0
14 Lip and oral cavity cancer	1911.0
15 Esophageal cancer	1806.0
16 Ischemic stroke	1626.0
17 Alzheimer's disease and other dementias	1543.0
18 Falls	1384.0
19 Liver cancer	1292.0
20 Prostate cancer	1242.0
50 Diabetes mellitus type 2	
69 Influenza	
112 Other lower respiratory infections	

Leading causes After Redistribution	Deaths
1 Ischemic heart disease	13679.85
2 Tracheal, bronchus, and lung cancer	10665.75
3 Colon and rectum cancer	9469.2
4 Diabetes mellitus type 2	8970.2
5 Influenza	7797.83
6 Chronic obstructive pulmonary disease	6912.77
7 Alzheimer's disease and other dementias	6511.01
8 Intracerebral hemorrhage	6125.58
9 Cirrhosis and other chronic liver diseases	5977.91
10 Ischemic stroke	5729.71
11 Stomach cancer	4158.69
12 Hypertensive heart disease	4011.63
13 Self-harm by other specified means	3930.94
14 Esophageal cancer	3167.73
15 Pancreatic cancer	2993.1
16 Other lower respiratory infections	2983.43
17 Urinary tract infections and interstitial nephritis	2938.58
18 Chronic kidney disease	2834.67
19 Breast cancer	2689.7
20 Lip and oral cavity cancer	2144.22
21 Falls	
23 Liver cancer	
24 Prostate cancer	

Leading causes of death before and after garbage code redistribution: Ukraine - 2015.

Causes are connected by arrows before and after redistribution. Infectious diseases are shown in red, non-communicable causes in blue, and injuries in green.
In addition to garbage redistribution, the diagram also reflects the deaths moved during misassignment correction for Alzheimer's disease and other dementias.

Leading causes Before Redistribution	Deaths	Leading causes After Redistribution	Deaths
1 Ischemic heart disease	295319.32	1 Ischemic heart disease	314953.6
2 Ischemic stroke	50731.86	2 Ischemic stroke	65509.08
3 Cirrhosis and other chronic liver diseases	15390.8	3 Cirrhosis and other chronic liver diseases	16364.27
4 Tracheal, bronchus, and lung cancer	13185.69	4 Intracerebral hemorrhage	15232.75
5 Colon and rectum cancer	11631.22	5 Tracheal, bronchus, and lung cancer	14238.79
6 Intracerebral hemorrhage	11615.25	6 Alzheimer's disease and other dementias	13835.94
7 Chronic obstructive pulmonary disease	7730.98	7 Colon and rectum cancer	12737.58
8 Self-harm by other specified means	7679.32	8 Self-harm by other specified means	10011.42
9 Stomach cancer	7556.06	9 Alcoholic cardiomyopathy	8503.57
10 Breast cancer	7363.31	10 Stomach cancer	8392.62
11 Drug-susceptible tuberculosis	5214.21	11 Chronic obstructive pulmonary disease	8005.48
12 Alcohol use disorders	4880.13	12 Breast cancer	7862.07
13 Pancreatic cancer	4594.3	13 Drug-susceptible tuberculosis	5422.43
14 Alcoholic cardiomyopathy	3926.1	14 Other cardiomyopathy	5210.96
15 Prostate cancer	3853.6	15 Alcohol use disorders	5131.23
16 Subarachnoid hemorrhage	3238.95	16 Pancreatic cancer	5037.8
17 Lower extremity peripheral arterial disease	2795.2	17 Other lower respiratory infections	4913.99
18 Kidney cancer	2463.4	18 Prostate cancer	4237.28
19 HIV/AIDS resulting in other diseases	2416.49	19 Subarachnoid hemorrhage	3732.26
20 Vascular intestinal disorders	2408.99	20 Lower extremity peripheral arterial disease	3099.52
46 Alzheimer's disease and other dementias		24 Vascular intestinal disorders	
51 Other cardiomyopathy		25 Kidney cancer	
66 Other lower respiratory infections		26 HIV/AIDS resulting in other diseases	

Leading causes of death before and after garbage code redistribution: Uruguay - 2015.

Causes are connected by arrows before and after redistribution. Infectious diseases are shown in red, non-communicable causes in blue, and injuries in green.
In addition to garbage redistribution, the diagram also reflects the deaths moved during misassignment correction for Alzheimer’s disease and other dementias.

Leading causes Before Redistribution	Deaths	Leading causes After Redistribution	Deaths
1 Ischemic heart disease	2374.0	1 Ischemic heart disease	3677.33
2 Tracheal, bronchus, and lung cancer	1321.0	2 Ischemic stroke	2352.12
3 Alzheimer's disease and other dementias	1184.0	3 Chronic obstructive pulmonary disease	1891.07
4 Chronic obstructive pulmonary disease	1156.0	4 Tracheal, bronchus, and lung cancer	1669.63
5 Colon and rectum cancer	995.0	5 Alzheimer's disease and other dementias	1430.66
6 Breast cancer	668.0	6 Colon and rectum cancer	1427.47
7 Ischemic stroke	626.0	7 Breast cancer	875.87
8 Pancreatic cancer	575.0	8 Diabetes mellitus type 2	866.58
9 Prostate cancer	553.0	9 Pneumococcal pneumonia	826.77
10 Self-harm by other specified means	473.0	10 Intracerebral hemorrhage	793.88
11 Urinary tract infections and interstitial nephritis	456.0	11 Prostate cancer	740.7
12 Hypertensive heart disease	417.0	12 Pancreatic cancer	730.83
13 Stomach cancer	398.0	13 Hypertensive heart disease	722.74
14 Intracerebral hemorrhage	364.0	14 Stomach cancer	597.91
15 Kidney cancer	245.0	15 Urinary tract infections and interstitial nephritis	577.37
16 Parkinson's disease	242.0	16 Self-harm by other specified means	524.71
17 Esophageal cancer	232.0	17 Other lower respiratory infections	494.13
18 Non-rheumatic calcific aortic valve disease	228.0	18 Non-rheumatic calcific aortic valve disease	379.18
19 Subarachnoid hemorrhage	216.0	19 Falls	355.42
20 Aortic aneurysm	199.0	20 Chronic kidney disease due to hypertension	350.96
31 Diabetes mellitus type 2		22 Subarachnoid hemorrhage	
36 Chronic kidney disease due to hypertension		23 Parkinson's disease	
76 Falls		24 Esophageal cancer	
100 Pneumococcal pneumonia		25 Kidney cancer	
112 Other lower respiratory infections		28 Aortic aneurysm	

Leading causes of death before and after garbage code redistribution: United States of America - 2015.

Causes are connected by arrows before and after redistribution. Infectious diseases are shown in red, non-communicable causes in blue, and injuries in green.

In addition to garbage redistribution, the diagram also reflects the deaths moved during misassignment correction for Alzheimer's disease and other dementias.

Leading causes Before Redistribution	Deaths
1 Ischemic heart disease	366790.0
2 Alzheimer's disease and other dementias	245661.0
3 Tracheal, bronchus, and lung cancer	154584.0
4 Chronic obstructive pulmonary disease	149964.0
5 Colon and rectum cancer	53419.0
6 Cirrhosis and other chronic liver diseases	51687.0
7 Breast cancer	42072.0
8 Hypertensive heart disease	41694.0
9 Pancreatic cancer	41649.0
10 Falls	33382.0
11 Chronic kidney disease	30376.0
12 Prostate cancer	28912.0
13 Parkinson's disease	27795.0
14 Intracerebral hemorrhage	26034.0
15 Motor vehicle road injuries	25417.0
16 Atrial fibrillation and flutter	23859.0
17 Self-harm by other specified means	22042.0
18 Self-harm by firearm	22017.0
19 Endocrine, metabolic, blood, and immune disorders	19548.0
20 Interstitial lung disease and pulmonary sarcoidosis	19427.0
22 Chronic kidney disease due to hypertension	
26 Diabetes mellitus type 2	
28 Ischemic stroke	
59 Influenza	
141 Opioid use disorders	

Leading causes After Redistribution	Deaths
1 Ischemic heart disease	467947.89
2 Alzheimer's disease and other dementias	194246.74
3 Chronic obstructive pulmonary disease	182064.1
4 Tracheal, bronchus, and lung cancer	178197.19
5 Ischemic stroke	99290.43
6 Colon and rectum cancer	71346.76
7 Cirrhosis and other chronic liver diseases	63926.74
8 Diabetes mellitus type 2	61778.77
9 Hypertensive heart disease	56087.69
10 Intracerebral hemorrhage	55905.64
11 Breast cancer	50543.89
12 Pancreatic cancer	49968.01
13 Falls	43273.5
14 Chronic kidney disease	40449.45
15 Prostate cancer	38013.12
16 Opioid use disorders	36522.83
17 Influenza	34509.97
18 Chronic kidney disease due to hypertension	32471.16
19 Parkinson's disease	30331.54
20 Atrial fibrillation and flutter	29866.56
21 Motor vehicle road injuries	
25 Self-harm by other specified means	
28 Self-harm by firearm	
29 Endocrine, metabolic, blood, and immune disorders	
30 Interstitial lung disease and pulmonary sarcoidosis	

Leading causes of death before and after garbage code redistribution: Uzbekistan - 2015.

Causes are connected by arrows before and after redistribution. Infectious diseases are shown in red, non-communicable causes in blue, and injuries in green.

In addition to garbage redistribution, the diagram also reflects the deaths moved during misassignment correction for Alzheimer's disease and other dementias.

Leading causes Before Redistribution	Deaths
1 Ischemic heart disease	40898.0
2 Hypertensive heart disease	20197.0
3 Cirrhosis and other chronic liver diseases	7442.0
4 Intracerebral hemorrhage	4017.0
5 Ischemic stroke	2461.0
6 Other lower respiratory infections	2315.0
7 Self-harm by other specified means	2310.0
8 Neonatal preterm birth	2039.0
9 Drug-susceptible tuberculosis	1779.0
10 Stomach cancer	1754.0
11 Pedestrian road injuries	1412.0
12 Neonatal encephalopathy due to birth asphyxia and trauma	1351.0
13 Breast cancer	1321.0
14 Tracheal, bronchus, and lung cancer	1314.0
15 Drowning	1071.0
16 Motor vehicle road injuries	1058.0
17 Liver cancer	924.0
18 Brain and central nervous system cancer	890.0
19 Chronic obstructive pulmonary disease	789.0
20 Esophageal cancer	766.0
62 Diabetes mellitus type 2	
127 Alzheimer's disease and other dementias	

Leading causes After Redistribution	Deaths
1 Ischemic heart disease	50061.67
2 Hypertensive heart disease	21110.28
3 Cirrhosis and other chronic liver diseases	8260.49
4 Intracerebral hemorrhage	7464.09
5 Ischemic stroke	7179.95
6 Other lower respiratory infections	6708.51
7 Diabetes mellitus type 2	4849.27
8 Self-harm by other specified means	2767.07
9 Neonatal preterm birth	2142.01
10 Drug-susceptible tuberculosis	2005.82
11 Stomach cancer	1915.72
12 Pedestrian road injuries	1735.85
13 Tracheal, bronchus, and lung cancer	1464.26
14 Breast cancer	1421.25
15 Neonatal encephalopathy due to birth asphyxia and trauma	1420.46
16 Motor vehicle road injuries	1356.52
17 Chronic obstructive pulmonary disease	1237.81
18 Drowning	1187.2
19 Alzheimer's disease and other dementias	1043.5
20 Brain and central nervous system cancer	1039.3
22 Liver cancer	
27 Esophageal cancer	

Leading causes of death before and after garbage code redistribution: Saint Vincent and the Grenadines - 2015.

Causes are connected by arrows before and after redistribution. Infectious diseases are shown in red, non-communicable causes in blue, and injuries in green.

In addition to garbage redistribution, the diagram also reflects the deaths moved during misassignment correction for Alzheimer’s disease and other dementias.

Leading causes Before Redistribution	Deaths
1 Ischemic heart disease	91.0
2 Prostate cancer	51.0
3 HIV/AIDS resulting in other diseases	21.0
4 Hypertensive heart disease	21.0
5 Intracerebral hemorrhage	18.0
6 Breast cancer	15.0
7 Other cardiovascular and circulatory diseases (internal)	13.0
8 Decubitus ulcer	12.0
9 Physical violence by firearm	12.0
10 Colon and rectum cancer	12.0
11 Stomach cancer	11.0
12 Tracheal, bronchus, and lung cancer	11.0
13 Diabetes mellitus type 2	10.0
14 Physical violence by sharp object	9.0
15 Cervical cancer	7.0
16 Cirrhosis and other chronic liver diseases	7.0
17 Chronic kidney disease due to hypertension	7.0
18 Drowning	6.0
19 Chronic obstructive pulmonary disease	6.0
20 Ovarian cancer	5.0

49 Alzheimer's disease and other dementias
62 Motor vehicle road injuries
65 Ischemic stroke
98 Other lower respiratory infections

Leading causes After Redistribution	Deaths
1 Ischemic heart disease	113.93
2 Diabetes mellitus type 2	64.55
3 Prostate cancer	55.76
4 Ischemic stroke	47.98
5 Intracerebral hemorrhage	41.68
6 Hypertensive heart disease	30.65
7 Other lower respiratory infections	28.02
8 HIV/AIDS resulting in other diseases	26.93
9 Breast cancer	18.92
10 Colon and rectum cancer	17.01
11 Alzheimer's disease and other dementias	16.73
12 Other cardiovascular and circulatory diseases (internal)	15.53
13 Stomach cancer	15.48
14 Motor vehicle road injuries	13.96
15 Chronic kidney disease due to hypertension	13.55
16 Tracheal, bronchus, and lung cancer	13.39
17 Physical violence by firearm	13.21
18 Cervical cancer	12.32
19 Decubitus ulcer	11.22
20 Physical violence by sharp object	10.19

21 Cirrhosis and other chronic liver diseases
23 Chronic obstructive pulmonary disease
25 Drowning
33 Ovarian cancer

Leading causes of death before and after garbage code redistribution: Venezuela (Bolivarian Republic of) - 2014.

Causes are connected by arrows before and after redistribution. Infectious diseases are shown in red, non-communicable causes in blue, and injuries in green.

In addition to garbage redistribution, the diagram also reflects the deaths moved during misassignment correction for Alzheimer’s disease and other dementias.

Leading causes Before Redistribution	Deaths
1 Ischemic heart disease	26887.0
2 Physical violence by firearm	8310.0
3 Diabetes mellitus type 2	7297.0
4 Chronic obstructive pulmonary disease	4563.0
5 Tracheal, bronchus, and lung cancer	4065.0
6 Hypertensive heart disease	4057.0
7 Cirrhosis and other chronic liver diseases	3069.0
8 Chronic kidney disease due to diabetes mellitus type 2	3043.0
9 Intracerebral hemorrhage	2900.0
10 Neonatal preterm birth	2656.0
11 Prostate cancer	2589.0
12 Breast cancer	2230.0
13 HIV/AIDS resulting in other diseases	2050.0
14 Chronic kidney disease	1961.0
15 Colon and rectum cancer	1958.0
16 Stomach cancer	1884.0
17 Chronic kidney disease due to hypertension	1851.0
18 Cervical cancer	1563.0
19 Diarrheal diseases	1395.0
20 Neonatal sepsis and other neonatal infections	1261.0
22 Ischemic stroke	
38 Alzheimer's disease and other dementias	
39 Pedestrian road injuries	
97 Other lower respiratory infections	

Leading causes After Redistribution	Deaths
1 Ischemic heart disease	28267.54
2 Physical violence by firearm	13394.41
3 Diabetes mellitus type 2	9098.28
4 Ischemic stroke	5126.44
5 Intracerebral hemorrhage	5009.07
6 Chronic obstructive pulmonary disease	4763.04
7 Alzheimer's disease and other dementias	4465.27
8 Hypertensive heart disease	4460.57
9 Tracheal, bronchus, and lung cancer	4389.84
10 Other lower respiratory infections	4353.95
11 Chronic kidney disease due to diabetes mellitus type 2	4066.66
12 Cirrhosis and other chronic liver diseases	3659.12
13 Pedestrian road injuries	3098.37
14 Prostate cancer	2902.25
15 Neonatal preterm birth	2738.0
16 Breast cancer	2458.42
17 Stomach cancer	2377.69
18 Colon and rectum cancer	2349.9
19 HIV/AIDS resulting in other diseases	2150.33
20 Chronic kidney disease due to hypertension	2131.87
21 Chronic kidney disease	
22 Cervical cancer	
24 Diarrheal diseases	
25 Neonatal sepsis and other neonatal infections	

Leading causes of death before and after garbage code redistribution: United States Virgin Islands - 2015.

Causes are connected by arrows before and after redistribution. Infectious diseases are shown in red, non-communicable causes in blue, and injuries in green.

In addition to garbage redistribution, the diagram also reflects the deaths moved during misassignment correction for Alzheimer’s disease and other dementias.

Leading causes Before Redistribution	Deaths
1 Ischemic heart disease	65.0
2 Alzheimer's disease and other dementias	48.0
3 Physical violence by firearm	35.0
4 Prostate cancer	22.0
5 Tracheal, bronchus, and lung cancer	21.0
6 Hypertensive heart disease	20.0
7 Colon and rectum cancer	14.0
8 Breast cancer	13.0
9 Cirrhosis and other chronic liver diseases	11.0
10 Chronic obstructive pulmonary disease	8.0
11 Chronic kidney disease due to hypertension	7.0
12 Atrial fibrillation and flutter	7.0
13 HIV/AIDS resulting in other diseases	7.0
14 Intracerebral hemorrhage	7.0
15 Esophageal cancer	6.0
16 Chronic kidney disease	6.0
17 Drowning	6.0
18 Diabetes mellitus type 2	5.0
19 Alcohol use disorders	5.0
20 Ischemic stroke	5.0
22 Chronic kidney disease due to diabetes mellitus type 2	
23 Other non-Hodgkin lymphoma	
76 Other lower respiratory infections	

Leading causes After Redistribution	Deaths
1 Ischemic heart disease	103.59
2 Physical violence by firearm	36.24
3 Ischemic stroke	35.68
4 Diabetes mellitus type 2	32.99
5 Prostate cancer	31.38
6 Hypertensive heart disease	30.17
7 Tracheal, bronchus, and lung cancer	28.22
8 Intracerebral hemorrhage	27.09
9 Colon and rectum cancer	21.25
10 Breast cancer	18.14
11 Cirrhosis and other chronic liver diseases	16.6
12 Alzheimer's disease and other dementias	13.82
13 Chronic obstructive pulmonary disease	12.76
14 Chronic kidney disease due to hypertension	11.67
15 Chronic kidney disease due to diabetes mellitus type 2	10.83
16 Atrial fibrillation and flutter	9.69
17 HIV/AIDS resulting in other diseases	9.06
18 Other lower respiratory infections	8.71
19 Chronic kidney disease	7.62
20 Other non-Hodgkin lymphoma	7.35
21 Esophageal cancer	
24 Drowning	
25 Alcohol use disorders	

Leading causes of death before and after garbage code redistribution: South Africa - 2015.

Causes are connected by arrows before and after redistribution. Infectious diseases are shown in red, non-communicable causes in blue, and injuries in green.

In addition to garbage redistribution, the diagram also reflects the deaths moved during misassignment correction for Alzheimer’s disease and other dementias.

Leading causes Before Redistribution	Deaths
1 Drug-susceptible tuberculosis	33530.0
2 HIV/AIDS resulting in other diseases	22482.0
3 Other unspecified infectious diseases	16396.9
4 Ischemic heart disease	12662.0
5 Diarrheal diseases	10082.0
6 Chronic obstructive pulmonary disease	8654.0
7 Hypertensive heart disease	7167.0
8 Physical violence by sharp object	6547.0
9 Tracheal, bronchus, and lung cancer	5728.0
10 Unintentional firearm injuries	5714.0
11 Asthma	3802.0
12 Neonatal preterm birth	3689.0
13 Cervical cancer	3688.0
14 Idiopathic epilepsy	3598.0
15 Breast cancer	3478.0
16 Prostate cancer	3091.0
17 Meningitis	3035.0
18 Esophageal cancer	2868.0
19 Cirrhosis and other chronic liver diseases	2737.0
20 Colon and rectum cancer	2678.0

21 Intracerebral hemorrhage
23 Chronic kidney disease due to hypertension
26 Alzheimer's disease and other dementias
29 Diabetes mellitus type 2
58 Motor vehicle road injuries
65 Ischemic stroke
67 Other lower respiratory infections
71 Influenza
74 Self-harm by other specified means
196 HIV/AIDS

Leading causes After Redistribution	Deaths
1 HIV/AIDS	48653.27
2 Drug-susceptible tuberculosis	38080.84
3 HIV/AIDS resulting in other diseases	29954.94
4 Diabetes mellitus type 2	27899.54
5 Ischemic heart disease	23173.51
6 Other unspecified infectious diseases	18707.03
7 Intracerebral hemorrhage	15533.29
8 Ischemic stroke	15412.85
9 Influenza	14917.2
10 Diarrheal diseases	14486.88
11 Hypertensive heart disease	13686.09
12 Chronic obstructive pulmonary disease	13205.95
13 Other lower respiratory infections	10267.01
14 Physical violence by sharp object	7698.79
15 Tracheal, bronchus, and lung cancer	7412.54
16 Unintentional firearm injuries	6268.57
17 Motor vehicle road injuries	6023.25
18 Chronic kidney disease due to hypertension	5573.99
19 Self-harm by other specified means	5289.56
20 Alzheimer's disease and other dementias	5117.31

22 Cirrhosis and other chronic liver diseases
24 Breast cancer
25 Asthma
26 Cervical cancer
27 Prostate cancer
28 Meningitis
29 Neonatal preterm birth
30 Colon and rectum cancer
32 Esophageal cancer
49 Idiopathic epilepsy

This figure also captures additional corrections applied prior to redistribution, namely adjustments made for the misdiagnosis of Parkinson's, atrial fibrillation, and Alzheimer's disease and other dementias not discussed in detail in this paper (Appendix Figure 1). Additionally, only real underlying causes are included in this figure. For that reason, one will not see "Garbage Code" listed in the deaths prior to redistribution.
